# Association of traffic air pollution and rhinitis quality of life in Peruvian children with asthma

**DOI:** 10.1371/journal.pone.0193910

**Published:** 2018-03-21

**Authors:** Sonali Bose, Karina Romero, Kevin J. Psoter, Frank C. Curriero, Chen Chen, Caroline M. Johnson, Deepak Kaji, Patrick N. Breysse, D’Ann L. Williams, Murugappan Ramanathan, William Checkley, Nadia N. Hansel

**Affiliations:** 1 Johns Hopkins Department of Medicine—Baltimore, MD, United States of America; 2 Icahn School of Medicine at Mount Sinai, the GASP investigators, New York, NY, United States of America; 3 Johns Hopkins Bloomberg School of Public Health—Baltimore, MD, United States of America; 4 Johns Hopkins School of Medicine, Department of Pediatrics—Baltimore, MD, United States of America; 5 Department of Global Health, University of Washington, Seattle, WA, United States of America; 6 Johns Hopkins School of Medicine, Department of Otolaryngology—Baltimore, MD, United States of America; University of Torino, ITALY

## Abstract

**Background:**

Air pollution exposure may contribute to rhinoconjunctivitis morbidity in children with underlying airways disease. Prior studies have not assessed rhinoconjunctivitis-related quality of life (QOL) in children with asthma chronically exposed to air pollution.

**Methods:**

Children ages 9–19 years with asthma from peri-urban Peru, self-reporting rhinoconjunctivitis symptoms (n = 484), were administered the Rhinoconjunctivitis QOL Questionnaire (RQLQ) at repeated intervals over one year, with scores dichotomized into bothered (>0) and not bothered (= 0). Individual weekly exposures to particulate matter<2.5μm (PM_2.5_) and its black carbon (BC) component were estimated by inverse distance weighted methods. Generalized estimating equations, adjusting for covariates, estimated associations of PM_2.5_ and BC with QOL.

**Results:**

Participants were on average 13 years old, 55% female, and majority were atopic (77%). Mean (SD) PM_2.5_ and BC concentrations were 21(3.2) μg/m3 and 4.4(1.5) μg/m3, respectively. In adjusted multi-pollutant models, each 10μg/m3 increase in PM_2.5_ was associated with increased odds of worse rhinoconjunctivitis QOL (OR;[95% CI]: 1.83;[1.33,2.52]). A 10% increase in the BC proportion was associated with higher rhinitis burden (OR;[95% CI]: 1.80;[1.22,2.66]), while increases in the non-BC component of PM did not significantly impact rhinoconjunctivitis QOL. Associations were similar regardless of atopy.

**Conclusion:**

Higher PM2.5 and BC exposure is associated with worse rhinitis QOL among asthmatic children.

## Introduction

Asthma and allergic respiratory diseases are the leading chronic illnesses among children and have become a rising global health burden in the last several decades [1,2]. Specifically, rhinoconjunctivitis, which is commonly co-morbid with asthma independent of atopic status [3,4], disproportionately affects low-income countries [5] and causes significant physical and psychosocial morbidity that can adversely impact quality of life (QOL) [6,7]. Recent attention to the growing prevalence of rhinitis symptoms and its impact on respiratory morbidity has led to efforts to identify environmental factors contributing to uncontrolled upper respiratory disease among vulnerable children [8,9].

Rapid urbanization in the developing world has been implicated in the increased prevalence of respiratory and allergic disease in populations specifically living in urban areas with poor air quality [10], supported by an increased risk of rhinitis found among children living in urban areas with higher air pollution exposure compared to non-urban children, especially in areas of high vehicular traffic [11–13]. In addition, recent studies investigating the influence of traffic-related air pollutants (TRAP) upon rhinitis have shown that individual exposure to higher concentrations of ambient pollutants such as particulate matter less than 2.5 microns in diameter (PM_2.5_), are significantly associated with increased prevalence of allergic rhinitis (AR) [14–16], as well as AR-related hospitalizations [17], suggesting that TRAP may contribute to both the development and morbidity of rhinitis. More specifically, Kim et al. reported an association between black carbon exposure (a component of PM and indicator of vehicular traffic-related combustion) and greater need for allergic rhinitis treatment among elementary school children [18], highlighting the significance of PM composition in identifying environmental risk factors for rhinitis-related morbidity.

However, despite these recent reports, studies have not been consistent in determining adverse effects of particulate air pollution upon rhinitis [19–23]. While the majority of this research pertains to industrialized nations whereby findings cannot be generalized to developing regions of the world, such conflicting reports in the literature warrant further research employing quantitative measures of exposure to TRAP including black carbon, as well as multidimensional validated questionnaires in order to systematically evaluate the adverse health effects of air pollution exposure, especially among children with pre-existing asthma. Moreover, no prior study has examined the impact of air pollution upon QOL related to uncontrolled upper respiratory symptoms. We have previously reported a high prevalence rate of AR (23%) among children living in urban Lima, Peru [24], yet the role of TRAP in contributing to the individual burden of upper airway disease is unclear.

Accordingly, the aim of this study was to determine the association of ambient PM_2.5_ and black carbon concentrations upon rhinitis QOL among children and adolescents with asthma living in peri-urban regions of Peru.

## Methods

### Study setting and design

We conducted a longitudinal study of children with a diagnosis of asthma between the ages of 9 and 19 years living in two adjacent peri-urban residential communities in Lima: Pampas de San Juan de Miraflores (Pampas) and Villa el Salvador (Villa), nested within a larger parent study, Genetic Asthma Susceptibility and Pollution in Peru (GASP). Both of these resource-poor areas have experienced rapid growth over the past several decades, and residents live in open-air homes located on paved or unpaved roads, with varied proximity to highly trafficked main commuter routes using diverse modes of transportation and an old vehicle fleet. Only children with a pre-existing physician diagnosis of asthma, and who either reported taking medications for asthma or reported active symptoms over the past year, were followed longitudinally (n = 484). Children were excluded if they had a history of other chronic respiratory conditions, such as tuberculosis. Enrollment occurred between 2011 and 2014, and children with active asthma were followed for a total of 9 months in Pampas and 6 months in Villa in the parent study. Written informed consent was obtained from parents or guardians for participants younger than 18 years of age. The study was approved by the Institutional Review Boards of Johns Hopkins School of Medicine (Baltimore, MD) and of Asociacion Benefica (A.B). PRISMA (Lima, Peru).

### Demographic and clinical characterization

Baseline questionnaires collecting information regarding demographics, personal and family histories, and asthma status were administered to all children in the presence of caregivers. Current diagnosis of rhinitis was defined by a positive answer to the following standardized International Study of Asthma and Allergies in Childhood (ISAAC) core question: In the past 12 months, has your child had a problem with sneezing, or a runny, or blocked nose when he/she DID NOT have a cold or the flu?” [5] Children were assessed for rhinitis QOL monthly for the entire 6-month data collection period in Villa and monthly for the last 3 months of the 9-month study period in Pampas. Rhinitis-related QOL was assessed using validated Spanish language versions of the 7-point Juniper Rhinoconjunctivitis Quality of Life Questionnaire (RQLQ) for children (ages 9–11) and adolescents (12–19 years) that evaluate symptoms over the previous week—Pediatric Rhinoconjunctivitis Quality of Life Questionnaire (PRQLQ) and its adolescent counterpart (ARQLQ), respectively [25,26]. Overall PRQLQ scores are the un-weighted average of 23 questions across five individual domain components (nasal symptoms, ocular symptoms, practical problems, activity limitation, and other symptoms) while adolescents are scored and averaged for 25 questions across 6 domains (nasal symptoms, ocular symptoms, practical problems, activity limitation, emotional function, and non-hay fever symptoms). Domain-specific scores are the arithmetic means of the questions in each domain. Asthma severity was classified as mild, moderate, or severe, according to the National Asthma Education and Prevention Program (NAEPP) guidelines [27]. Children were assessed for asthma control monthly using the validated Spanish language versions of the pediatric Asthma Control Test (ACT) for children aged 9–11 years and the ACT for adolescents aged 12–19 years to evaluate asthma symptoms over the previous 4 weeks [28,29]. Asthma control was classified as “well controlled” at a score ≥20 and “uncontrolled” at a score <20.

Baseline anthropometry including height and weight were measured, and lung function was assessed using a flow-based portable spirometer (SpiroPro, Jaeger/Cardinal Health, Hoechberg, Germany) according to American Thoracic Society (ATS) guidelines [30]. Bronchodilator response was measured 15–20 minutes after 4 puffs of a short-acting bronchodilator were administered according to ATS guidelines with ≥12% and ≥200cc change in either FEV_1_ or FVC noted as a positive bronchodilator response [30]. Atopy, or allergic sensitization, was defined as a positive response to total serum IgE of a fluorescent enzyme immunoassay (ImmunoCAP250; Phadia, Kalamazoo, Michigan, USA) to one of three phadiotopes: 1.Mold Multi-allergen MX2 (M1, Penicillium notatum; M2, Cladosporium herbarum (Hormodendrum); M3, Aspergillus fumigates; M5, Candida albicans; M6, Alternaria alternate/tenius; M8, Helminthospoirum halodes); 2. House Multiallergen HX2 (H2, House dust Hollister Stier; D1, Dermatophagoides pteronyssinus; D2, Dermatophagoides farina; I6, Blatella germanica, Cockroach), and 3. Animal Epidermal Multi-allergen EX2 (E1, Cat dander and epithelium; E5, Dog dander; E6, Guinea pig epithelium; E87, Rat; E88, Mouse).

### Environmental assessment

Fifteen outdoor locations from each community (n = 30 total) were chosen to monitor continuous, week-long ambient concentrations of PM_2.5_ (particulate matter with aerodynamic < 2.5 μm), black carbon (BC), temperature, and humidity at varying distances from the main roads within each site. Monitoring equipment was located on the roofs of houses in a secure and weather-protected environment. PM was collected gravimetrically with a Personal DataRam (pDR model 1200, Thermo Scientific Boston, MA, USA) and a BGI 400 Personal Air Sampling Pump (BGI Incorporated, Waltham, MA) set to a flow-rate of 4L/min, with flow-rate consistency verified before and after sampling using a BIOS Defender 530 calibrator (BIOS International, Butler, NJ, USA). Each PDR was connected via a cyclone to a cassette with a 37 mm Teflon filter (Pall Corporation, Port Washington, NY), which were pre- and post-weighed at the Johns Hopkins Bloomberg School of Public Health in a temperature and humidity controlled weighing room using a Mettler-Toledo MT5 microbalance (Mettler-Toledo, Inc., Columbus, OH) following EPA standard protocol. BC was assessed from the same 37 mm Teflon PM_2.5_ filters collected using the pDR 1200 and analyzed using the Magee OT-21 SootScan^TM^ Model Transmissometer (Magee Scientific Corporation, Berkely, CA, USA). Temperature and humidity data captured by the Hobo Data Loggers (Onset Corp, Poasset, MA) every five minutes throughout each weekly sample were used to correct PM_2.5_ and BC measurements.

Individual-level pollutant and meteorological exposures were determined based on inverse distance weighted (IDW) methods. Briefly, participants and monitors were spatially referenced within each region. The average (mean) measurements for each monitor was determined for the 28-day period prior to outcome (RQLQ) assessment of each participant, and the final individual-level exposure was taken as the IDW mean average based on the 15 monitors within each region. The use of IDW in this study provides a more informative approach than just assigning exposure based on the closest one or few monitors by accounting for the spatial variation in exposure with the limited data available.

### Statistical analysis

Demographic and clinical characteristics of children with asthma and rhinoconjunctivitis symptoms at baseline were tabulated overall and compared between study sites using Student *t* tests with unequal variances and Chi squared tests for continuous and categorical variables, respectively. Socioeconomic status (SES) was determined for households in the study regions using principal component analysis comprised of 15 household variables, including 12 individual household assets, 1 household size variable, and 2 variables related to years of parental education, with lower SES values reflecting higher levels of poverty.

The outcome of interest was RQLQ which was categorized as not bothered (score = 0) or bothered (score>0). To evaluate the effects of PM_2.5_ and BC on rhinoconjunctivitis QOL amongst children with asthma, multivariable logistic regression, based on generalized estimating equations with an unstructured working correlation to account for the repeated measures of subjects during follow-up was used. Single and multi-pollutant models were evaluated that considered PM_2.5_ and BC exposure on odds of being bothered by rhinoconjunctivitis. All analyses were adjusted for the average (IDW mean) temperature and relative humidity in the 30-day period, as well as the following individual level baseline covariates: age (years), sex (male vs. female), SES, Cole Body Mass Index (BMI) classification (normal, overweight, obese), site of recruitment, and forced expiratory volume in the first second (FEV_1_) percent predicted based on the Global Lung Function Initiative (GLI) reference values for mixed ethnic populations [31].

For multipollutant models, we evaluated the proportion of BC within PM_2.5_ (change in BC as a component of the total PM_2.5_). Inference from these models then takes the form of a change in odds of being bothered by rhinoconjunctivitis associated with an increase of BC while holding PM_2.5_ fixed. Finally, the association of BC and PM_2.5_ within each individual domain of the RQLQ was evaluated using the previously described models. Results are presented as odds ratios (OR) with corresponding 95% confidence intervals (CI) estimated by robust standard errors. Effect size estimates are presented for a 10μg/m^3^ increase in PM_2.5_ and a 10% increase in proportion of BC exposure. Interactions between both PM and BC with study site (Pampas vs. Villa) and atopic status were individually evaluated to determine whether the effect of pollutants on rhinitis QOL was differential between site or among those who were atopic compared to non-atopic individuals. Analyses were performed using STATA Version 13.0 (StataCorp LP, College Station, TX) and the R statistical environment (Version 3.3.0).

## Results

### Demographics

Children with active asthma (n = 484) were followed longitudinally across both sites. Overall, children had a mean age of 13 years and 55% were female. The majority of children were atopic (77%). One-third of the children had mild, 44% had moderate, and 22% had severe persistent asthma, with no significant difference in this distribution between sites (p = 0.64). Despite 66% of subjects having moderate to severe disease by NAEPP guidelines, only 12% had uncontrolled asthma at initial visit. Twenty-six percent reported taking controller medication for asthma (including inhaled or oral corticosteroids). Baseline FEV_1_ was not significantly different in Pampas vs. Villa, in contrast to site-related differences in SES and BMI (Table 1). Fifteen percent of children were found to have reversibility upon repeat post-bronchodilator spirometric testing.

**Table 1:** Baseline demographic and clinical characteristics of asthmatic children overall and by site.

	Overall	Pampas	Villa	*P* value
	(n = 462)	(n = 239)	(n = 223)	
*Demographic characteristics*				
Age (years), mean (SD)	13.2 (2.56)	13.6 (2.61)	12.9 (2.45)	0.012
Male, n (%)	207 (44.8)	96 (40.2)	111 (49.8)	0.038
SES^1^, mean (SD)	0.31 (1.68)	-0.32 (1.64)	0.97 (1.45)	<0.001
Number of follow up visits, mean (SD)	3.6 (1.05)	2.8 (0.45)	4.4 (0.87)	<0.001
*Clinical characteristics*				
Cole BMI, n (%)				0.023
Normal	218 (47.2)	126 (52.7)	92 (41.3)	
Overweight	150 (32.5)	74 (31.0)	76 (34.1)	
Obese	94 (20.4)	39 (16.3)	55 (24.7)	
FEV_1_ (percent predicted), mean (SD)	105.5 (14.2)	105.1 (14.4)	105.9 (14.0)	0.551
FVC (percent predicted), mean (SD)	116.11 (15.22)	116.59 (15.01)	115.60 (15.46)	0.487
FEV_1_/FVC (percent predicted), mean (SD)	97.39(7.76)	96.58 (8.17)	98.26 (7.20)	0.019
Atopy present, n (%)	324 (77.0)	170 (79.1)	154 (74.8)	0.294
Uncontrolled asthma^2^, n (%)	51 (11.7)	8 (3.6)	43 (20.3)	<0.001
Asthma severity				0.639
Mild intermittent	62 (15.3)	37 (16.7)	25 (13.5)	
Mild persistent	74 (18.2)	43 (19.5)	31 (16.8)	
Moderate persistent	180 (44.3)	95 (43.0)	85 (46.0)	
Severe persistent	90 (22.2)	46 (20.8)	44 (23.8)	
*Environmental characteristics*				
Temp (°C), mean (SD)	21.1 (2.46)	21.4 (0.38)	25.5 (0.62)	<0.001
Temp (°C), (25-75^th^ percentiles)	21.4 (19.5, 25.7)	21.5 (21.2, 21.7)	25.5 (25.1, 25.8)	
Humidity (°C), mean (SD)	74.5 (4.47)	25.3 (1.06)	77.0 (3.45)	<0.001
Humidity (°C), (25-75^th^ percentiles)	74.4 (71.9, 77.3)	25.2 (24.5, 25.9)	76.5 (74.6, 78.7)	
PM_2.5_, mean (SD)	21.1 (3.19)	23.7 (2.16)	18.4 (1.30)	<0.001
PM_2.5_, (25-75^th^ percentiles)	21.5 (18.2, 23.4)	23.2 (22.0, 24.8)	18.2 (17.7, 18.8)	
BC, mean (SD)	4.4 (1.52)	3.0 (0.48)	5.9 (0.46)	<0.001
BC, (25-75^th^ percentiles)	3.9 (3.0, 5.9)	3.0 (2.7, 3.4)	5.9 (1.7, 2.0)	

Abbreviations: SD: standard deviation; SES: socioeconomic status; BMI: body mass index; FEV_1_: forced expiratory volume in one second; FVC: forced vital capacity in one second.

*P* values based on Student t tests with unequal variances and chi squared tests for continuous and categorical variables, respectively.

^1^ Principal component analysis based on 15 household variables including variables for household size and individual assets and parental education.

^2^ Asthma control test (ACT) scores at initial visit: a score ≥20 was classified as controlled.

Of 484 children, 463 had rhinoconjunctivitis QOL assessed during at least one follow-up visit, and 462 children had complete environmental exposure data to enter the analytic sample, comprising 1654 individual measures of RQLQ. In Pampas, 92–95% of the 239 children, and in Villa, 79–95% of 223 children, completed the RQLQ assessment at each of the follow up visits over the 3- and 6-month periods, respectively. Overall, children completed the RQLQ an average of 3 times during the study and 311 of the 462 children (67%) reported being bothered by rhinoconjunctivitis at least once during the study period.

### Environmental assessment

The overall mean (SD) PM_2.5_ concentration was 21.1 (3.19) μg/m^3^ and mean (SD) BC concentration was 4.4 (1.52) μg/m^3^. PM_2.5_ was significantly higher in Pampas compared to Villa (23.7 (2.16) vs. 18.4 (1.30) μg/m^3^, p<0.001), though the opposite was true for BC (3.0 (0.48) vs. 5.9 (0.46) μg/m^3^, P<0.001, Pampas vs. Villa respectively). Mean (SD) temperature and humidity in Pampas were 21.1°C (2.44) and 72.2% (4.02), respectively, and in Villa were 21.0°C (2.48) and 77.0% (3.45), respectively.

### Association of pollutants with rhinoconjunctivitis QOL

In single-pollutant unadjusted models, there was no significant relationship between a 10 μg/m^3^ change in PM_2.5_ and the odds of being impaired by rhinoconjunctivitis symptoms. (OR; [95% CI]: 1.10 [0.91, 1.33]). In contrast, a 1 μg/m^3^ increase in BC exposure was significantly associated with an increase in odds of adverse QOL associated with symptomatic rhinoconjunctivitis. (OR; [95% CI]: 1.13 [1.07, 1.21]). In adjusted models, both PM_2.5_ and BC were significantly associated with increased odds of adverse rhinoconjunctivitis QOL (OR; [95% CI]: 1.42; [1.09, 1.85]) per 10 μg/m^3^ increase in PM_2.5_) and (1.34; [1.18, 1.53] per 1 μg/m^3^ increase in BC), respectively (Table 2).

**Table 2:** Unadjusted and adjusted^1^ single and multipollutant logistic regression analyses evaluating the association of PM_2.5_ and black carbon with rhinoconjunctivitis quality of life in young children and adolescents residing in Pampas and Villa, Peru.

	Unadjusted OR (95% CI)	Adjusted^1^ OR (95% CI)
Single pollutant		
PM_2.5_^2^	1.10 (0.91, 1.33)	1.42 (1.09, 1.85)
BC^3^	1.13 (1.07, 1.21)	1.34 (1.18, 1.53)
Multipollutant: proportion of BC		
PM_2.5_^2^	1.26 (0.99, 1.60)	1.83 (1.33, 2.52)
BC^4^	1.32 (1.15, 1.52)	1.80 (1.22, 2.66)
Multipollutant: proportion of nonBC		
PM_2.5_^2^	1.26 (0.99, 1.60)	1.83 (1.33, 2.52)
nonBC^4^	0.76 (0.66, 0.87)	0.56 (0.38, 0.82)

Abbreviations: OR: odds ratio; CI: confidence interval; PM_2.5_: particulate matter <2.5μg in aerodynamic diameter; BC: black carbon.

Note: Unadjusted models consider account for pediatric/adolescent survey.

^1^All models adjusted for the following covariates: pediatric/adolescent survey, age, gender, socioeconomic status, Cole BMI classification (normal, overweight, obese), site (Pampas, Villa), baseline FEV_1_ percent predicted, temperature (°C) and humidity (%).

^2^ Reflects a 10μg/m^3^ increase in PM_2.5_ exposure.

^3^Reflects a 1μg/m^3^ increase in BC exposure.

^4^Reflects a 10% increase in proportion of BC or nonBC component of PM_2.5._

In adjusted multi-pollutant models accounting for the proportion of BC content within PM and holding PM levels constant, we found that the association between higher PM_2.5_ and rhinoconjunctivitis morbidity was preserved (OR; [95% CI]: 1.83; [1.33, 2.52]). Specifically, a 10% increase in the proportion of BC content was also significantly associated with increased odds of being bothered by rhinoconjunctivitis (OR; [95% CI]: 1.80; [1.22, 2.66]). In contrast, an increase in the non-BC proportion of PM did not significantly impact rhinoconjunctivitis QOL (Table 2). No interaction with study site was observed for either PM_2.5_ or BC (S1 Table).

### Interaction between atopic status and air pollution exposure

Four hundred and twenty-one children (87%) had available allergic sensitization data, and all interaction p-values between pollutants and atopy were >0.10 suggesting a similar effect of pollutant exposure on RQLQ in atopic and in non-atopic children. When stratified by atopic status, both atopic and nonatopic children demonstrated qualitatively similar associations between pollutant exposure and rhinoconjunctivitis QOL, though for nonatopic children, given the limited sample size, this did not reach statistical significance (Table 3).

**Table 3:** Adjusted^1^ single and multipollutant logistic regression analyses evaluating the association of PM_2.5_ and black carbon with rhinoconjunctivitis quality of life in young children and adolescents residing in Pampas and Villa, Peru, stratified by atopic status.

	Overall (n = 421)	Atopic (n = 324)	Non-atopic (n = 97)	
	Adjusted^1^ OR (95% CI)	Adjusted^1^ OR (95% CI)	Adjusted^1^ OR (95% CI)	p-value^2^
Single pollutant				
PM_2.5_^3^	1.34 (1.01, 1.77)	1.44 (1.08, 1.92)	1.05 (0.54, 2.02)	0.501
BC^4^	1.33 (1.16, 1.52)	1.37 (1.18, 1.58)	1.21 (0.88, 1.65)	0.920
Multipollutant: proportion of BC				
PM_2.5_^3^	1.77 (1.27, 2.48)	1.84 (1.29, 2.61)	1.64 (0.72, 3.73)	0.675
BC^5^	1.90 (1.27, 2.85)	1.76 (1.14, 2.73)	2.32 (0.91, 5.91)	0.624

Abbreviations: OR: odds ratio; CI: confidence interval; PM_2.5_: particulate matter <2.5μg in aerodynamic diameter; BC: black carbon.

Note: Unadjusted models consider account for pediatric/adolescent survey.

^1^All models adjusted for the following covariates: pediatric/adolescent survey, age, gender, socioeconomic status, Cole BMI classification (normal, overweight, obese), site (Pampas, Villa), baseline FEV_1_ percent predicted, temperature (°C) and humidity (%).

^2^ P-values represent models that include interaction terms of pollutant and atopic status.

^3^Reflects a 10μg/m^3^ increase in PM_2.5_ exposure.

^4^Reflects a 1μg/m^3^ increase in BC exposure.

^5^Reflects a 10% increase in proportion of BC.

### Rhinitis QOL domains

**Fig 1:**
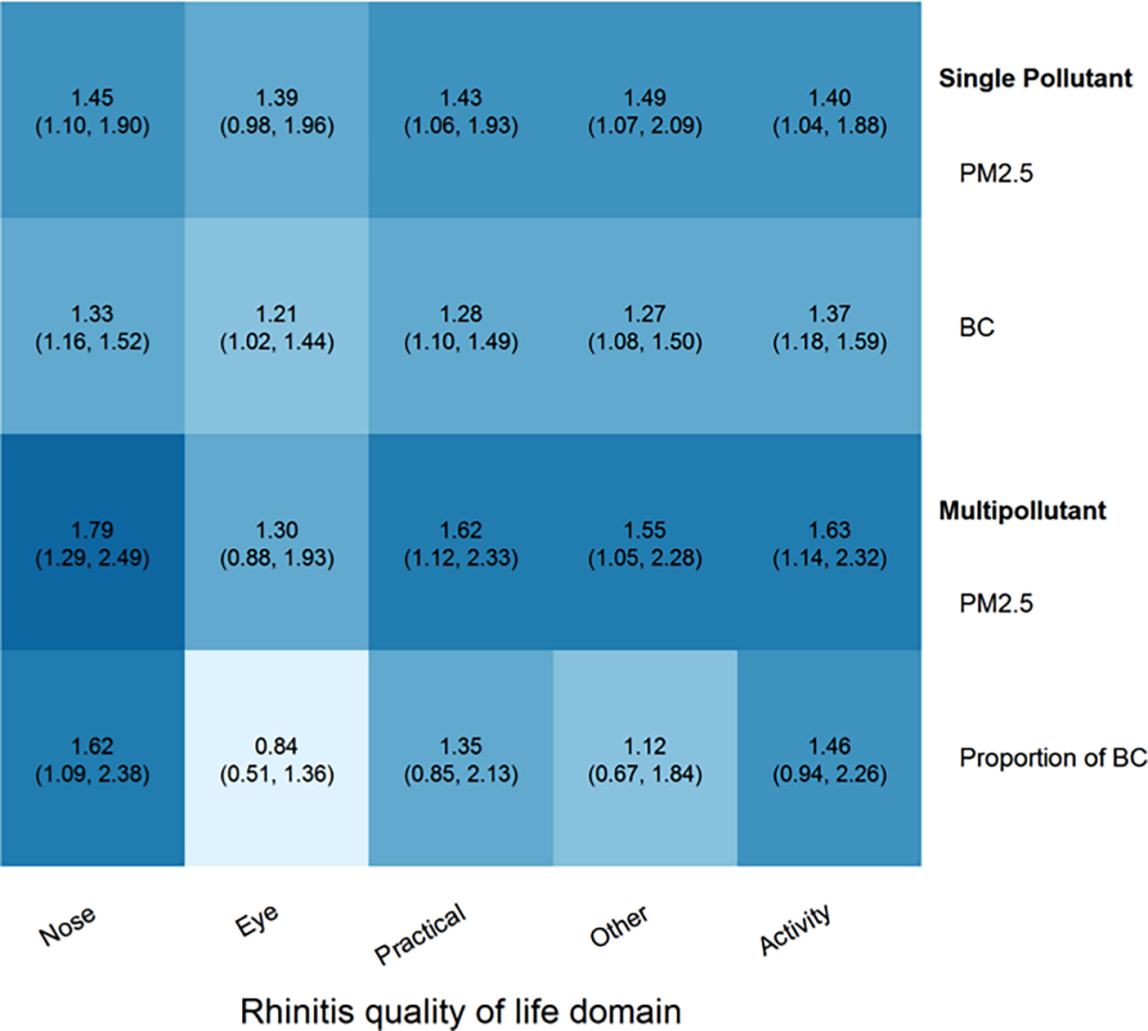
Single and multi-pollutant associations of PM_2.5_ and black carbon with rhinoconjunctivitis quality of life, by domain. All models were adjusted for the following covariates: pediatric/adolescent survey, age, sex, socioeconomic status, Cole BMI classification (normal, overweight, obese), site (Pampas, Villa), baseline FEV_1_ percent predicted, temperature (°C) and humidity (%). Increased intensity of color reflects higher odds ratios.

To understand if the association between pollution and QOL was being driven by any one particular domain of the RQLQ, the associations of exposure to PM_2.5_ and BC with each of the individual domains in RQLQ were analyzed. We found that among the domains common to young children and adolescents, the odds of being troubled specifically by nasal symptoms in multi-pollutant models was 1.79 times higher (95% CI: 1.29, 2.49) for each 10 μg/m^3^ increase in PM_2.5_ exposure, and 1.62 (95% CI: 1.09, 2.38) times higher for a 10% increase in proportion of BC component. (Fig 1)

## Discussion

In our study of children and adolescents with asthma living in peri-urban Peru, we found that ambient particulate air pollution was significantly associated with a higher burden of rhinoconjunctivitis symptoms. In multi-pollutant models, a 10 μg/m^3^ increase in airborne PM_2.5_ was associated with an 83% increased odds of adverse rhinoconjunctivitis QOL. Additionally, a 10% increase in proportion of the BC component of PM_2.5_, an indicator of traffic-related combustion, was associated with an 80% increased odds of being bothered by rhinoconjunctivitis. Relative increases in the concentration of non-black carbon PM components did not have the same deleterious effect upon QOL, further supporting a unique role of black carbon in the observed association between air pollution and rhinoconjunctivitis symptoms. Furthermore, the adverse effects of air pollution were appreciated in both allergic and non-allergic children alike, illustrating the potentially nonselective effects of PM and BC exposure on respiratory health. To our knowledge, this is the first study to implicate PM, especially the black carbon component, in poorer rhinitis QOL, suggesting that traffic-related air pollution may play a significant role in the burden of rhinitic disease among children with asthma, regardless of atopic status.

A significant proportion (two-thirds) of our cohort was found to have rhinoconjunctivitis symptoms during the study period. While worldwide estimates of rhinitis vary greatly by geography depending on definitions used and the availability of allergic testing [32,33], prior population-based studies in the US, for example, have reported that approximately 50% of children with asthma have comorbid allergic rhinitis [34]. In comparison, our findings report a potentially higher burden of rhinoconjunctivitis that may reflect residence within regions that have experienced unparalleled urbanization and growth within recent decades. In other Latin American countries such as Bolivia, for example, children living in urban compared to rural areas were several times more likely to have severe rhinoconjunctivitis (OR; [95%CI]: 2.8; [1.2–6.6]) [35], and we have previously shown similar urban-rural disparities within Peru, where rates of rhinitis were significantly higher in peri-urban adolescents in Lima compared to their rural counterparts (23% vs. 12%, respectively, p<0.001) [36].

Global studies of respiratory disease such as the International Study of Asthma and Allergies in Childhood (ISAAC) have found that many developing regions of the world are subject to health effects specifically from vehicular sources of ambient air pollution [37], which tend to accompany the surge of industrialization in low- and middle-income nations [38]. Our results further support adverse consequences from vehicular traffic in developing regions, as our findings regarding black carbon exposure, whose major source is from traffic combustion, suggests a link between TRAP and upper respiratory morbidity. Prior studies have relied heavily on exposure proxies such as self-reports or spatial measures to heavily trafficked roads to estimate exposure to mobile sources of air pollution. For example, an early cross-sectional study of 3,703 German school children demonstrated that adolescents that self-categorized their exposure to truck traffic as either “frequent” or “constant” compared to “never,” had significantly higher symptoms of allergic rhinitis (OR; [95 CI%]: 1.71; [1.36–2.15] and 1.96; [1.4–2.76], respectively) [39]. Self-report of residential truck traffic was also used to estimate exposure in participants of the third phase of ISAAC, which found that higher exposures (“almost the whole day” versus “never”) were associated with increased rhinoconjunctivitis symptoms in both children and adolescents (OR; [95%CI]: 1.33; [1.20–1.48] and 1.39; [1.27–1.52] respectively) [37]. Similarly, Zuraimi et al. used self-reported traffic densities to estimate traffic exposures in a cross-sectional study of 2,994 randomly selected children, and found a dose-response relationship of increasingly heavy traffic densities (low, medium, heavy) to increased prevalence ratios (PR) of rhinoconjunctivitis symptoms (PR;[CI]: 1.92; [1.17–3.16]) [40].

Our study therefore builds upon prior self-reported exposure assessments by using fine spatial mapping of measured pollutants, specifically PM_2.5_ and BC, providing quantitative exposure assessments of PM_2.5_ and black carbon to strengthen the association between TRAP and symptomatic rhinitis. More recent studies from Asia [11,12,15–18] have provided more quantitative estimates of particulate air pollution exposure, demonstrating that higher concentrations of PM_2.5_ and black carbon contribute to rhinitis-related morbidity. In the present report, we have extended this investigation to Latin America, and in Peru in particular, where the burden of asthma and allergic rhinitis are high, to more fully characterize the influence of air pollutant on rhinoconjunctivitis quality of life.

A particular strength of our study is the use of validated, age-specific questionnaires of RQLQ to account for multiple domains within the burden of rhinoconjunctivitis disease among children and adolescents, illustrating not only its physical manifestations but also the associated psychological and social distress that interferes with individual daily activities and productivity, increasingly recognized important outcomes [41]. Importantly, children with rhinoconjunctivitis compared to those with rhinitis alone have been reported to have poorer quality of life, with the presence of conjunctivitis symptoms doubling the risk of the disease impacting daily activities [42]. These negative effects are especially pronounced in the young, where critical growth periods of cognitive, emotional, and social development are vulnerable to dysfunction such as learning impairment, sleep and mood disturbances, and peer and familial tension, and whose caregivers may also be impacted by the disease [43]. The burden of rhinoconjunctivitis also stems from increased health care utilization, as illustrated by a study that demonstrated 13–31 μg/m^3^ increases in ambient sulfur dioxide over London were associated with a subsequent 24.5% increase in consultations for allergic rhinitis over the days following exposure, particularly in children (p<0.00001) [44]. Appreciating the detrimental effects of rhinoconjunctivitis on QOL in such populations may therefore have implications for further management of the disease, especially in young children.

Our study has a few limitations. First, the relationship of PM and black carbon to rhinoconjunctivitis symptoms in children with asthma we demonstrate here cannot be generalized to non-asthmatic children who have rhinoconjunctivitis alone. However, the finding that there was no interaction between pollutant concentrations and atopic status based on IgE sensitization suggest that the link between air pollution and rhinoconjunctivitis symptoms is possibly independent of allergic mechanisms. In support of this finding, recent animal data has demonstrated evidence that mice experimentally exposed chronically to PM_2.5_ were found to have increased non-allergic eosinophilic sinonasal inflammation and epithelial barrier dysfunction in the nasal airway, compared with control mice [45]. Nevertheless, further work is needed to identify factors that influence individual susceptibilities to non-allergen airborne exposures with regards to patient-reported rhinoconjunctivitis symptoms. Finally, our study assessed PM_2.5_ and BC simultaneously to QOL for up to 6 months per participant, therefore, it is unclear what the impact of long-term environmental exposures might be upon rhinitis-related morbidity due to progression or chronicity of disease.

In conclusion, ambient pollution exposure to airborne PM_2.5_, specifically its BC component, significantly increased the odds of worse rhinoconjunctivitis-related QOL among both atopic and non-atopic peri-urban children and adolescents with asthma. To our knowledge, this is the first study to demonstrate the impact of black carbon upon rhinoconjunctivitis symptoms within the milieu of a rapidly industrializing environment. These findings raise concern and highlight the need for increased recognition of rhinitis symptoms in children with asthma, dedicated management of upper airway disease in these populations, and targeted strategies to improve air quality for children. Further efforts aimed at reducing such environmental exposures are critical to protect vulnerable populations.

## Supporting information

S1 Table Unadjusted and adjusted^1^ single and multipollutant logistic regression analyses evaluating the association of PM_2.5_ and black carbon with rhinoconjunctivitis quality of life in young children and adolescents residing in Pampas and Villa, Peru. (DOCX)

S1 File Limited and de-identified dataset. (HTM)

## Abbreviations

(PM): particulate matter
(BC): black carbon
(QOL): quality of life
