# Supplementary material for: Association of traffic air pollution and rhinitis quality of life in Peruvian children with asthma
Source: PLoS One. 2018 Mar 21;13(3):e0193910. doi: 10.1371/journal.pone.0193910 (PMC5862476; doi:10.1371/journal.pone.0193910)
Supplement: S1 Table — (DOCX) [file pone.0193910.s001.docx]

S1 Table: Unadjusted and adjusted^1^ single and multipollutant logistic regression analyses evaluating the association of PM_2.5_ and black carbon with rhinoconjunctivitis quality of life in young children and adolescents residing in Pampas and Villa, Peru

|  | Unadjusted OR  (95% CI) | Adjusted^1^ OR  (95% CI) |
| --- | --- | --- |
| ***Pampas*** |  |  |
| Single pollutant |  |  |
| PM_2.5_^2^ | 1.10 (0.65, 1.86) | 2.00 (0.97, 4.11) |
| BC^3^ | 1.21 (0.79, 1.86) | 1.79 (1.01, 3.16) |
| Multipollutant: proportion of BC |  |  |
| PM_2.5_^2^ | 0.92 (0.52, 1.63) | 1.82 (0.85, 3.87) |
| BC^4^ | 28.35 (1.18, 683.86) | 5.16 (0.18, 149.97) |
| Multipollutant: proportion of nonBC |  |  |
| PM_2.5_^2^ | 0.92 (0.52, 1.63) | 1.82 (0.85, 3.87) |
| nonBC^4^ | 0.04 (0.01, 0.85) | 0.19 (0.01, 5.64) |
| ***Villa*** |  |  |
| Single pollutant |  |  |
| PM_2.5_^2^ | 1.13 (0.89, 1.42) | 1.24 (0.89, 1.74) |
| BC^3^ | 1.18 (1.07, 1.30) | 1.37 (1.16, 1.61) |
| Multipollutant: proportion of BC |  |  |
| PM_2.5_^2^ | 1.58 (1.17, 2.14) | 2.35 (1.41, 3.90) |
| BC^4^ | 2.07 (1.47, 2.91) | 2.39 (1.40, 4.09) |
| Multipollutant: proportion of nonBC |  |  |
| PM_2.5_^2^ | 1.58 (1.17, 2.14) | 2.35 (1.41, 3.90) |
| nonBC^4^ | 0.48 (0.34, 0.68) | 0.42 (0.24, 0.72) |

Abbreviations: OR: odds ratio; CI: confidence interval; PM_2.5_: particulate matter <2.5μg in aerodynamic diameter; BC: black carbon

Note: Unadjusted models account for pediatric/adolescent survey
