# Supplementary material for: Association of traffic air pollution and rhinitis quality of life in Peruvian children with asthma
Source: PLoS One. 2018 Mar 21;13(3):e0193910. doi: 10.1371/journal.pone.0193910 (PMC5862476; doi:10.1371/journal.pone.0193910)
Supplement: S1 File — (HTM) [file pone.0193910.s002.htm]

| id | outcome | particulate\_matter | black\_carbon | proportion\_black\_carbon | proportion\_non\_black\_carbon | age\_category | age | sex | socioeconomic\_status | body\_mass\_index | fevppc | temperature | relative\_humidity | site | visit | number\_visits | atopy\_baseline | noserqlq\_categorical | eyerqlq\_categorical | probrqlq\_categorical | otrqlq\_categorical | actrqlq\_categorical | erqlq\_categorical | prefvcpp | prefvcz | preratiopp | preratioz | beclotake | fluttake | prednew | shsmoke | abqcurrsmoke | medication\_use | bd\_reversability | asthma\_severity | asthma\_control\_score | asthma\_control\_categorical | paqlq\_score | paqlq\_dichotomous | |
| 2019 |  | 21.991 | 2.705 | 12.300487 | 87.699516 | 1 | 12.804928 | 1 | -2.6271136 | 2 | 92.162918 | 22.314444 | 69.123 | 0 | 0 | 3 | 1 |  | | | | | | 113.8152236 | 1.263591662 | 86.59033291 | -1.898413359 | 2 | 2 |  | 0 | 0 | 0 | 1 |  | | | | | |
| 2019 | 0 | 16.966 | 1.72 | 10.137923 | 89.862076 | 1 | 14.187543 | 1 | -2.6271136 | 2 | 92.162918 | 25.116667 | 72.160004 | 0 | 19 | 3 | 1 | 0 | 0 | 0 | 0 | 0 | 0 |  | NA |  | NA | 2 | 2 | 2 | 0 | 0 | 0 |  | | 24 | 0 | 7 | 0 |  |
| 2019 | 0 | 19.801001 | 2.1099999 | 10.656027 | 89.343971 | 1 | 14.245038 | 1 | -2.6271136 | 2 | 92.162918 | 24.779997 | 74.370003 | 0 | 20 | 3 | 1 | 0 | 0 | 0 | 0 | 0 | 0 | 102.3446847 | 0.21444539 | 101.2243743 | 0.190477779 | 2 | 2 | 2 | 0 | 0 | 0 | 0 |  | 24 | 0 |  | | |
| 2019 | 0 | 21.952 | 2.3699999 | 10.796283 | 89.20372 | 1 | 14.283367 | 1 | -2.6271136 | 2 | 92.162918 | 23.718889 | 72.650002 | 0 | 21 | 3 | 1 | 0 | 0 | 0 | 0 | 0 | 0 |  | NA |  | NA | 2 | 2 | 2 | 0 | 0 | 0 |  | | 24 | 0 | 7 | 0 |  |
| 2033 |  | 23.342 | 3.402 | 14.574587 | 85.425415 | 1 | 13.106091 | 0 | -2.7576251 | 2 | 100.23751 | 21.610001 | 73.15 | 0 | 0 | 1 | 1 |  | | | | | | 124.6941769 | 2.128212902 | 81.29097641 | -2.484823683 | 2 | 2 |  | 0 | 0 | 0 | 0 | 1 |  | | | | |
| 2033 | 0 | 17.229 | 1.75 | 10.157293 | 89.842705 | 1 | 14.428473 | 0 | -2.7576251 | 2 | 100.23751 | 24.265556 | 74.160004 | 0 | 19 | 1 | 1 | 0 | 0 | 0 | 0 | 0 | 0 |  | NA |  | NA | 2 | 2 | 2 | 0 | 0 | 0 |  | 1 | 24 | 0 | 7 | 0 |  |
| 2101 |  | 22.013 | 2.768 | 12.574388 | 87.425613 | 1 | 12.246407 | 1 | -3.9488213 | 2 | 109.8959 | 18.610556 | 73.298 | 0 | 0 | 3 | 1 |  | | | | | | 119.4343643 | 1.775406937 | 95.58004624 | -0.683945154 | 2 | 2 |  | 0 | 0 | 0 | 0 | 3 |  | | | | |
| 2101 | 0 | 16.245001 | 1.7 | 10.464758 | 89.53524 | 1 | 13.604381 | 1 | -3.9488213 | 2 | 109.8959 | 26.813889 | 64.370003 | 0 | 19 | 3 | 1 | 0 | 0 | 0 | 0 | 0 | 0 |  | NA |  | NA | 2 | 2 | 2 | 0 | 0 | 0 |  | 3 | 24 | 0 | 7 | 0 |  |
| 2101 | 0 | 19.622 | 2.1300001 | 10.855163 | 89.144836 | 1 | 13.653662 | 1 | -3.9488213 | 2 | 109.8959 | 26.456667 | 66.790001 | 0 | 20 | 3 | 1 | 0 | 0 | 0 | 0 | 0 | 0 | 90.03172433 | -0.915408728 | 97.50705929 | -0.379162178 | 2 | 2 | 1 | 0 | 0 | 0 | 0 | 3 | 20 | 0 |  | | |
| 2101 | 0 | 20.416 | 2.27 | 11.118731 | 88.881271 | 1 | 13.678303 | 1 | -3.9488213 | 2 | 109.8959 | 25.895 | 65 | 0 | 21 | 3 | 1 | 0 | 0 | 0 | 0 | 0 | 0 |  | NA |  | NA | 2 | 2 | 1 | 0 | 0 | 0 |  | 3 | 24 | 0 | 7 | 0 |  |
| 2106 |  | 22.514 | 2.827 | 12.556631 | 87.443367 | 1 | 12.566735 | 1 | -2.9990819 | 3 | 98.458115 | 20.53389 | 74.114 | 0 | 0 | 3 | 1 |  | | | | | | 116.2697643 | 1.487427587 | 90.6627661 | -1.376206544 | 2 | 2 |  | 0 | 0 | 1 | 0 | 1 |  | | | | |
| 2106 | 0 | 16.580999 | 1.9 | 11.458899 | 88.5411 | 1 | 13.883641 | 1 | -2.9990819 | 3 | 98.458115 | 25.179443 | 71.400002 | 0 | 19 | 3 | 1 | 0 | 0 | 0 | 0 | 0 | 0 |  | NA |  | NA | 2 | 2 | 2 | 0 | 0 | 1 |  | 1 | 24 | 0 | 7 | 0 |  |
| 2106 | 0 | 20.797001 | 2.1900001 | 10.530365 | 89.469635 | 1 | 13.932922 | 1 | -2.9990819 | 3 | 98.458115 | 24.734446 | 74.830002 | 0 | 20 | 3 | 1 | 0 | 0 | 0 | 0 | 0 | 0 | 96.35795096 | -0.33386524 | 105.6674898 | 0.921675441 | 2 | 2 | 2 | 0 | 0 | 1 | 1 | 1 | 24 | 0 |  | | |
| 2106 | 0 | 22.051001 | 2.4200001 | 10.974559 | 89.025444 | 1 | 13.97399 | 1 | -2.9990819 | 3 | 98.458115 | 24.027224 | 72.839996 | 0 | 21 | 3 | 1 | 0 | 0 | 0 | 0 | 0 | 0 |  | NA |  | NA | 2 | 2 | 2 | 0 | 0 | 1 |  | 1 | 25 | 0 | 7 | 0 |  |
| 2110 |  | 23.234 | 3.187 | 13.716967 | 86.283035 | 0 | 10.370978 | 0 | -0.80598843 | 3 | 117.14352 | 21.219444 | 72.12 | 0 | 0 | 3 | 1 |  | | | | | | 126.4134733 | 2.301059182 | 98.97267807 | -0.182229742 | 2 | 2 |  | 0 | 0 | 0 | 0 | 3 |  | | | | |
| 2110 | 0 | 18.188999 | 1.86 | 10.225962 | 89.77404 | 0 | 11.701574 | 0 | -0.80598843 | 3 | 117.14352 | 25.065554 | 71.099998 | 0 | 19 | 3 | 1 | 0 | 0 | 0 | 0 | 0 |  | | NA |  | NA | 2 | 2 | 2 | 0 | 0 | 0 |  | 3 | 27 | 0 | 7 | 0 |  |
| 2110 | 0 | 21.767 | 2.4300001 | 11.163689 | 88.836311 | 0 | 11.748117 | 0 | -0.80598843 | 3 | 117.14352 | 24.844444 | 73.110001 | 0 | 20 | 3 | 1 | 0 | 0 | 0 | 0 | 0 |  | 118.6699325 | 1.630830701 | 98.10638281 | -0.327749121 | 2 | 2 | 2 | 0 | 0 | 0 | 0 | 3 | 27 | 0 |  | | |
| 2110 | 0 | 25.604 | 2.8099999 | 10.974847 | 89.025154 | 0 | 11.786448 | 0 | -0.80598843 | 3 | 117.14352 | 23.698332 | 71.839996 | 0 | 21 | 3 | 1 | 0 | 0 | 0 | 0 | 0 |  | | NA |  | NA | 2 | 2 | 2 | 0 | 0 | 0 |  | 3 | 27 | 0 | 7 | 0 |  |
| 2132 |  | 21.564 | 2.693 | 12.488406 | 87.511597 | 1 | 13.284052 | 0 | 1.2833946 | 2 | 103.04534 | 22.445555 | 74.969 | 0 | 0 | 3 | 1 |  | | | | | | 102.3910075 | 0.209551265 | 104.9318119 | 0.92939966 | 2 | 2 |  | 0 | 0 | 0 | 0 | 4 |  | | | | |
| 2132 | 0 | 18.733 | 2.0799999 | 11.1034 | 88.896599 | 1 | 14.67488 | 0 | 1.2833946 | 2 | 103.04534 | 24.156113 | 76.440002 | 0 | 19 | 3 | 1 | 0 | 0 | 0 | 0 | 0 | 0 |  | NA |  | NA | 2 | 2 | 2 | 0 | 0 | 0 |  | 4 | 24 | 0 | 7 | 0 |  |
| 2132 | 0 | 21.445 | 2.6600001 | 12.403824 | 87.596176 | 1 | 14.71321 | 0 | 1.2833946 | 2 | 103.04534 | 24.08889 | 78.510002 | 0 | 20 | 3 | 1 | 0 | 0 | 0 | 0 | 0 | 0 |  | NA |  | NA | 2 | 2 | 2 | 0 | 0 | 0 |  | 4 | 25 | 0 |  | | |
| 2132 | 0 | 23.621 | 2.75 | 11.642182 | 88.357819 | 1 | 14.748802 | 0 | 1.2833946 | 2 | 103.04534 | 22.798334 | 76.980003 | 0 | 21 | 3 | 1 | 0 | 0 | 0 | 0 | 0 | 0 |  | NA |  | NA | 2 | 2 | 2 | 0 | 0 | 0 |  | 4 | 24 | 0 | 7 | 0 |  |
| 2140 |  | 24.175 | 3.331 | 13.778697 | 86.221306 | 0 | 11.994524 | 1 | -0.11307801 | 4 | 118.22483 | 15.956667 | 78.662 | 0 | 0 | 1 | 0 |  | | | | | | 124.1505858 | 2.203177171 | 101.7065782 | 0.279922895 | 2 | 2 |  | 1 | 0 | 0 | 0 | 3 |  | | | | |
| 2140 | 0 | 16.231001 | 1.7 | 10.473784 | 89.526215 | 1 | 13.251198 | 1 | -0.11307801 | 4 | 118.22483 | 26.821667 | 64.330002 | 0 | 19 | 1 | 0 | 0 | 0 | 0 | 0 | 0 | 0 |  | NA |  | NA | 2 | 2 | 2 | 1 | 0 | 0 |  | 3 | 24 | 0 | 7 | 0 |  |
| 2144 |  | 24.537 | 3.225 | 13.143416 | 86.856583 | 0 | 11.605749 | 1 | -1.5018775 | 3 | 110.18252 | 19.142221 | 75.81 | 0 | 0 | 2 | 1 |  | | | | | | 120.6987804 | 1.88802029 | 97.65366415 | -0.37225994 |  | | | | | 0 | 0 | 3 |  | | | | |
| 2144 | 1 | 17.757999 | 1.97 | 11.093593 | 88.90641 | 1 | 12.829569 | 1 | -1.5018775 | 3 | 110.18252 | 25.164444 | 71.910004 | 0 | 19 | 2 | 1 | 1 | 1 | 1 | 1 | 1 | 1 |  | NA |  | NA |  | | 2 |  | | 0 |  | 3 | 24 | 0 | 7 | 0 |  |
| 2144 | 1 | 20.827999 | 2.26 | 10.850779 | 89.149223 | 1 | 12.870637 | 1 | -1.5018775 | 3 | 110.18252 | 24.975555 | 73.190002 | 0 | 20 | 2 | 1 | 1 | 1 | 1 | 1 | 1 | 1 | 113.1574724 | 1.203557035 | 93.08826188 | -1.036775424 |  | | 2 |  | | 0 | 0 | 3 | 19 | 1 |  | | |
| 2146 |  | 24.322 | 3.557 | 14.624619 | 85.375381 | 0 | 9.4127312 | 1 | -3.1156132 | 2 | 104.18021 | 15.160556 | 77.411 | 0 | 0 | 3 | 1 |  | | | | | | 113.3890231 | 1.195284146 | 98.30439857 | -0.268425812 | 2 | 2 |  | 0 | 0 | 0 | 0 | 4 |  | | | | |
| 2146 | 1 | 18.02 | 1.8200001 | 10.09989 | 89.900108 | 0 | 10.685832 | 1 | -3.1156132 | 2 | 104.18021 | 25.549444 | 68.449997 | 0 | 19 | 3 | 1 | 0 | 1 | 1 | 1 | 0 |  | | NA |  | NA | 2 | 2 | 2 | 0 | 0 | 0 |  | 4 | 23 | 0 | 7 | 0 |  |
| 2146 | 0 | 21.983 | 2.3800001 | 10.826549 | 89.173454 | 0 | 10.724161 | 1 | -3.1156132 | 2 | 104.18021 | 25.449444 | 69.93 | 0 | 20 | 3 | 1 | 0 | 0 | 0 | 0 | 0 |  | 113.3268076 | 1.213638584 | 94.65556304 | -0.825670071 | 2 | 2 | 2 | 0 | 0 | 0 | 0 | 4 | 26 | 0 |  | | |
| 2146 | 0 | 24.490999 | 2.5899999 | 10.575314 | 89.42469 | 0 | 10.762491 | 1 | -3.1156132 | 2 | 104.18021 | 23.608891 | 70.440002 | 0 | 21 | 3 | 1 | 0 | 0 | 0 | 0 | 0 |  | | NA |  | NA | 2 | 2 | 2 | 0 | 0 | 0 |  | 4 | 27 | 0 | 7 | 0 |  |
| 2147 |  | 24.487 | 3.601 | 14.705762 | 85.294235 | 0 | 9.5112934 | 1 | -0.4597266 | 4 | 139.02347 | 13.926111 | 79.505 | 0 | 0 | 3 | 1 |  | | | | | | 151.1315246 | 4.536492693 | 98.40481061 | -0.253073716 | 2 | 2 |  | 0 | 0 | 0 | 0 | 4 |  | | | | |
| 2147 | 0 | 17.686001 | 1.8 | 10.177541 | 89.822456 | 0 | 10.778918 | 1 | -0.4597266 | 4 | 139.02347 | 25.131111 | 69.720001 | 0 | 19 | 3 | 1 | 0 | 0 | 0 | 0 | 0 |  | | NA |  | NA | 2 | 2 | 2 | 0 | 0 | 0 |  | 4 | 25 | 0 | 7 | 0 |  |
| 2147 | 0 | 22.259001 | 2.3499999 | 10.557527 | 89.442474 | 0 | 10.817248 | 1 | -0.4597266 | 4 | 139.02347 | 24.938889 | 71.290001 | 0 | 20 | 3 | 1 | 0 |  | | | | | 135.3617188 | 3.211106463 | 99.38057645 | -0.100045426 | 2 | 2 | 2 | 0 | 0 | 0 | 0 | 4 | 27 | 0 |  | | |
| 2147 | 0 | 24.121 | 2.4100001 | 9.9912939 | 90.008705 | 0 | 10.855578 | 1 | -0.4597266 | 4 | 139.02347 | 23.126665 | 71.910004 | 0 | 21 | 3 | 1 | 0 | 0 | 0 | 0 | 0 |  | | NA |  | NA | 2 | 2 | 2 | 0 | 0 | 0 |  | 4 | 26 | 0 | 7 | 0 |  |
| 2208 |  | 24.646 | 3.358 | 13.624929 | 86.375069 | 0 | 9.8398361 | 0 | -1.5454687 | 2 | 82.953079 | 21.641111 | 71.349 | 0 | 0 | 3 | 1 |  | | | | | | 89.03557955 | -0.983365922 | 99.37891702 | -0.111283479 | 2 | 2 |  | 1 | 0 | 0 | 1 | 3 |  | | | | |
| 2208 | 0 | 17.768 | 1.9 | 10.693381 | 89.306618 | 0 | 11.033539 | 0 | -1.5454687 | 2 | 82.953079 | 25.253889 | 71.239998 | 0 | 19 | 3 | 1 | 0 | 0 | 0 | 0 | 0 |  | | NA |  | NA | 2 | 2 | 2 | 1 | 0 | 0 |  | 3 | 26 | 0 | 7 | 0 |  |
| 2208 | 1 | 20.941 | 2.3900001 | 11.413018 | 88.586983 | 0 | 11.069131 | 0 | -1.5454687 | 2 | 82.953079 | 24.890001 | 74.209999 | 0 | 20 | 3 | 1 | 1 | 0 | 1 | 1 | 0 |  | 87.80369333 | -1.093804586 | 100.0335213 | 0.005973111 | 2 | 2 | 2 | 1 | 0 | 0 | 0 | 3 | 24 | 0 |  | | |
| 2208 | 0 | 22.893 | 2.6199999 | 11.444546 | 88.55545 | 0 | 11.123888 | 0 | -1.5454687 | 2 | 82.953079 | 24.182222 | 72.849998 | 0 | 21 | 3 | 1 | 0 | 0 | 0 | 0 | 0 |  | | NA |  | NA | 2 | 2 | 2 | 1 | 0 | 0 |  | 3 | 27 | 0 | 7 | 0 |  |
| 2210 |  | 25.15 | 3.983 | 15.836978 | 84.163025 | 1 | 12.744696 | 1 | 0.3116996 | 2 | 92.618332 | 18.691668 | 70.127 | 0 | 0 | 3 | 0 |  | | | | | | 102.2044397 | 0.202213439 | 97.02450251 | -0.463155534 | 2 | 2 |  | 0 | 0 | 0 | 1 | 3 |  | | | | |
| 2210 | 0 | 18.058001 | 1.47 | 8.1404362 | 91.859566 | 1 | 13.982204 | 1 | 0.3116996 | 2 | 92.618332 | 26.472778 | 65.089996 | 0 | 19 | 3 | 0 | 0 | 0 | 0 | 0 | 0 | 0 |  | NA |  | NA | 2 | 2 | 2 | 0 | 0 | 0 |  | 3 | 24 | 0 | 7 | 0 |  |
| 2210 | 0 | 18.304001 | 2 | 10.926573 | 89.073425 | 1 | 14.017796 | 1 | 0.3116996 | 2 | 92.618332 | 26.596666 | 66.470001 | 0 | 20 | 3 | 0 | 0 | 0 | 0 | 0 | 0 | 0 | 102.1910409 | 0.200631181 | 104.20176 | 0.678402458 | 2 | 2 | 2 | 0 | 0 | 0 | 0 | 3 | 24 | 0 |  | | |
| 2210 | 0 | 22.254 | 2.6099999 | 11.728229 | 88.271774 | 1 | 14.056126 | 1 | 0.3116996 | 2 | 92.618332 | 25.498335 | 64.510002 | 0 | 21 | 3 | 0 | 0 | 0 | 0 | 0 | 0 | 0 |  | NA |  | NA | 2 | 2 | 2 | 0 | 0 | 0 |  | 3 | 24 | 0 | 7 | 0 |  |
| 2267 |  | 25.691 | 2.084 | 8.1117897 | 91.888206 | 1 | 17.634497 | 1 | -2.591012 | 2 | 117.88763 | 23.105 | 72.258 | 0 | 0 | 3 | 1 |  | | | | | | 140.3847087 | 3.647234267 | 89.84954798 | -1.375274441 | 2 | 2 |  | 0 | 0 | 0 | 1 | 1 |  | | | | |
| 2267 | 0 | 19.756001 | 1.9400001 | 9.8198013 | 90.180199 | 1 | 18.781656 | 1 | -2.591012 | 2 | 117.88763 | 25.800001 | 69.620003 | 0 | 19 | 3 | 1 | 0 | 0 | 0 | 0 | 0 | 0 |  | NA |  | NA | 2 | 2 | 2 | 0 | 0 | 0 |  | 1 | 22 | 0 | 6.5 | 1 |  |
| 2267 | 0 | 23.672001 | 2.4200001 | 10.223048 | 89.776955 | 1 | 18.819986 | 1 | -2.591012 | 2 | 117.88763 | 25.524445 | 69.699997 | 0 | 20 | 3 | 1 | 0 | 0 | 0 | 0 | 0 | 0 | 128.7570882 | 2.601230505 | 79.85295592 | -2.491540254 | 2 | 2 | 2 | 0 | 0 | 0 | 1 | 1 | 20 | 0 |  | | |
| 2267 | 0 | 35.158001 | 2.8399999 | 8.0778198 | 91.92218 | 1 | 18.858316 | 1 | -2.591012 | 2 | 117.88763 | 23.925556 | 70.360001 | 0 | 21 | 3 | 1 | 0 | 0 | 0 | 0 | 0 | 0 |  | NA |  | NA | 2 | 2 | 2 | 0 | 0 | 0 |  | 1 | 24 | 0 | 7 | 0 |  |
| 2272 |  | 26.545 | 2.781 | 10.476549 | 89.523453 | 1 | 12.410678 | 0 | 0.96862894 | 4 | 96.004791 | 22.09 | 72.18 | 0 | 0 | 3 | 1 |  | | | | | | 115.4843205 | 1.350401792 | 91.55247419 | -1.315393095 | 2 | 2 |  | 0 | 0 | 0 | 0 | 2 |  | | | | |
| 2272 | 1 | 18.844 | 1.85 | 9.8174486 | 90.182549 | 1 | 13.511293 | 0 | 0.96862894 | 4 | 96.004791 | 25.756666 | 68.879997 | 0 | 19 | 3 | 1 | 1 | 1 | 1 | 1 | 1 | 1 |  | NA |  | NA | 2 | 2 | 2 | 0 | 0 | 0 |  | 2 | 24 | 0 | 7 | 0 |  |
| 2272 | 1 | 21.282 | 2.25 | 10.572314 | 89.427689 | 1 | 13.568789 | 0 | 0.96862894 | 4 | 96.004791 | 25.41222 | 71.18 | 0 | 20 | 3 | 1 | 1 | 1 | 1 | 1 | 1 | 1 | 112.1303391 | 1.048355166 | 103.0473346 | 0.552897473 | 2 | 2 | 2 | 0 | 0 | 0 | 0 | 2 | 24 | 0 |  | | |
| 2272 | 1 | 27.591 | 2.8499999 | 10.329454 | 89.670547 | 1 | 13.587954 | 0 | 0.96862894 | 4 | 96.004791 | 24.319445 | 69.580002 | 0 | 21 | 3 | 1 | 1 | 1 | 1 | 1 | 1 | 1 |  | NA |  | NA | 2 | 2 | 2 | 0 | 0 | 0 |  | 2 | 24 | 0 | 7 | 0 |  |
| 2280 |  | 28.344 | 3.749 | 13.226786 | 86.773216 | 1 | 16.44627 | 1 | 1.6736273 | 2 | 99.95652 | 21.310556 | 70.266 | 0 | 0 | 3 | 0 |  | | | | | | 100.0518539 | 0.004731906 | 106.8102004 | 1.071292912 | 2 | 2 |  | 0 | 0 | 0 | 0 | 4 |  | | | | |
| 2280 | 0 | 16.958 | 1.8 | 10.614459 | 89.385544 | 1 | 17.59343 | 1 | 1.6736273 | 2 | 99.95652 | 25.030556 | 73.279999 | 0 | 19 | 3 | 0 | 0 | 0 | 0 | 0 | 0 | 0 |  | NA |  | NA | 2 | 2 | 2 | 0 | 0 | 0 |  | 4 | 24 | 0 | 7 | 0 |  |
| 2280 | 1 | 20.278 | 2.1600001 | 10.651938 | 89.348061 | 1 | 17.63176 | 1 | 1.6736273 | 2 | 99.95652 | 24.779997 | 73.470001 | 0 | 20 | 3 | 0 | 1 | 1 | 0 | 1 | 0 | 0 | 98.18619247 | -0.165416867 | 101.1367859 | 0.168537764 | 2 | 2 | 2 | 0 | 0 | 0 | 0 | 4 | 20 | 0 |  | | |
| 2280 | 0 | 21.507999 | 2.3099999 | 10.74019 | 89.259811 | 1 | 17.67009 | 1 | 1.6736273 | 2 | 99.95652 | 23.633333 | 72.699997 | 0 | 21 | 3 | 0 | 0 | 0 | 0 | 0 | 0 | 0 |  | NA |  | NA | 2 | 2 | 2 | 0 | 0 | 0 |  | 4 | 24 | 0 | 7 | 0 |  |
| 2281 |  | 28.142 | 3.746 | 13.311066 | 86.688934 | 1 | 15.12115 | 0 | 1.4721255 | 2 | 111.07928 | 20.458334 | 71.843 | 0 | 0 | 1 | 1 |  | | | | | | 113.4679282 | 1.155407504 | 101.4121175 | 0.247312413 | 2 | 2 |  | 0 | 0 | 1 | 0 | 4 |  | | | | |
| 2281 | 0 | 16.761999 | 1.74 | 10.380624 | 89.619377 | 1 | 16.235455 | 0 | 1.4721255 | 2 | 111.07928 | 25.541666 | 70.230003 | 0 | 19 | 1 | 1 | 0 | 0 | 0 | 0 | 0 | 0 |  | NA |  | NA | 2 | 2 | 2 | 0 | 0 | 1 |  | 4 | 24 | 0 | 7 | 0 |  |
| 2287 |  | 27.162 | 3.41 | 12.554304 | 87.445694 | 1 | 13.93566 | 1 | -2.093612 | 2 | 89.654549 | 21.062778 | 72.635 | 0 | 0 | 2 | 1 |  | | | | | | 97.85299893 | -0.196848251 | 97.85762293 | -0.328642437 | 2 | 2 |  | 0 | 0 | 0 | 0 | 4 |  | | | | |
| 2287 | 0 | 18.781 | 1.95 | 10.382833 | 89.617165 | 1 | 15.063655 | 1 | -2.093612 | 2 | 89.654549 | 25.303886 | 70.860001 | 0 | 19 | 2 | 1 | 0 | 0 | 0 | 0 | 0 | 0 |  | NA |  | NA | 2 | 2 | 2 | 0 | 0 | 0 |  | 4 | 24 | 0 | 7 | 0 |  |
| 2287 | 0 | 26.392 | 2.77 | 10.495605 | 89.504395 | 1 | 15.137577 | 1 | -2.093612 | 2 | 89.654549 | 23.801113 | 71.709999 | 0 | 21 | 2 | 1 | 0 | 0 | 0 | 0 | 0 | 0 |  | NA |  | NA | 2 | 2 | 2 | 0 | 0 | 0 |  | 4 | 25 | 0 | 7 | 0 |  |
| 2289 |  | 26.864 | 3.056 | 11.375819 | 88.624184 | 1 | 17.691992 | 1 | -1.0450851 | 3 | 78.929733 | 21.678333 | 72.425 | 0 | 0 | 3 | 0 |  | | | | | | 99.28512973 | -0.065172772 | 79.91938967 | -2.503537942 | 2 | 2 |  | 0 | 0 | 0 | 1 | 1 |  | | | | |
| 2289 | 0 | 18.632999 | 1.87 | 10.035958 | 89.964043 | 1 | 18.798084 | 1 | -1.0450851 | 3 | 78.929733 | 25.658333 | 69.440002 | 0 | 19 | 3 | 0 | 0 | 0 | 0 | 0 | 0 | 0 |  | NA |  | NA | 2 | 2 | 2 | 0 | 0 | 0 |  | 1 | 24 | 0 | 7 | 0 |  |
| 2289 | 1 | 21.256001 | 2.29 | 10.773428 | 89.22657 | 1 | 18.855577 | 1 | -1.0450851 | 3 | 78.929733 | 25.218887 | 71.970001 | 0 | 20 | 3 | 0 | 1 | 0 | 0 | 0 | 1 | 0 | 93.29440604 | -0.61155889 | 99.10449642 | -0.12902343 | 2 | 2 | 2 | 0 | 0 | 0 | 0 | 1 | 21 | 0 |  | | |
| 2289 | 0 | 26.32 | 2.79 | 10.600304 | 89.399696 | 1 | 18.872005 | 1 | -1.0450851 | 3 | 78.929733 | 24.187223 | 70.330002 | 0 | 21 | 3 | 0 | 0 | 0 | 0 | 0 | 0 | 0 |  | NA |  | NA | 2 | 2 | 2 | 0 | 0 | 0 |  | 1 | 23 | 0 | 7 | 0 |  |
| 2294 |  | 29.313 | 4.262 | 14.539624 | 85.460373 | 0 | 11.934292 | 0 | 1.7815342 | 3 | 103.58427 | 24.76111 | 64.393 | 0 | 0 | 3 | 1 |  | | | | | | 109.4119702 | 0.827397125 | 101.3094626 | 0.237646223 | 2 | 2 |  | 0 | 0 | 0 | 0 | 4 |  | | | | |
| 2294 | 0 | 16.905001 | 1.79 | 10.588583 | 89.411415 | 1 | 13.032169 | 0 | 1.7815342 | 3 | 103.58427 | 25.903889 | 68.190002 | 0 | 19 | 3 | 1 | 0 | 0 | 0 | 0 | 0 | 0 |  | NA |  | NA | 2 | 2 | 2 | 0 | 0 | 0 |  | 4 | 24 | 0 | 7 | 0 |  |
| 2294 | 1 | 23.111 | 2.6900001 | 11.63948 | 88.360519 | 1 | 13.070499 | 0 | 1.7815342 | 3 | 103.58427 | 25.799446 | 68.760002 | 0 | 20 | 3 | 1 | 1 | 0 | 0 | 1 | 1 | 1 | 108.0741982 | 0.704163049 | 93.2760059 | -1.067530731 | 2 | 2 | 2 | 0 | 0 | 0 | 0 | 4 | 24 | 0 |  | | |
| 2294 | 1 | 27.885 | 3.0599999 | 10.973641 | 89.02636 | 1 | 13.103354 | 0 | 1.7815342 | 3 | 103.58427 | 24.809443 | 67.809998 | 0 | 21 | 3 | 1 | 1 | 0 | 1 | 0 | 0 | 1 |  | NA |  | NA | 2 | 2 | 2 | 0 | 0 | 0 |  | 4 | 23 | 0 | 7 | 0 |  |
| 2303 |  | 29.771 | 3.5 | 11.756407 | 88.243591 | 1 | 15.909651 | 0 | 0.6383335 | 4 | 108.61603 | 20.656666 | 72.425 | 0 | 0 | 2 | 1 |  | | | | | | 115.8742264 | 1.353286408 | 101.256959 | 0.217545227 | 2 | 2 |  | 0 | 0 | 0 | 0 |  | | | | | |
| 2303 | 0 | 19.742001 | 2.01 | 10.181338 | 89.818665 | 1 | 17.015743 | 0 | 0.6383335 | 4 | 108.61603 | 25.927221 | 67.900002 | 0 | 19 | 2 | 1 | 0 | 0 | 0 | 0 | 0 | 0 |  | NA |  | NA | 2 | 2 | 2 | 0 | 0 | 0 |  | | 24 | 0 | 7 | 0 |  |
| 2303 | 0 | 23.313999 | 2.5599999 | 10.980527 | 89.01947 | 1 | 17.089664 | 0 | 0.6383335 | 4 | 108.61603 | 25.020002 | 70.449997 | 0 | 21 | 2 | 1 | 0 | 0 | 0 | 0 | 0 | 0 |  | NA |  | NA | 2 | 2 | 2 | 0 | 0 | 0 |  | | 24 | 0 | 7 | 0 |  |
| 2305 |  | 28.317 | 3.569 | 12.603736 | 87.396263 | 1 | 12.555783 | 1 | 1.7179354 | 2 | 50.567551 | 19.625555 | 74.987 | 0 | 0 | 3 | 1 |  | | | | | | 99.70668273 | -0.026925772 | 54.30392037 | -4.729010267 | 2 | 2 |  | 0 | 0 | 0 | 0 | 2 |  | | | | |
| 2305 | 1 | 17.471001 | 1.98 | 11.333066 | 88.666931 | 1 | 13.316906 | 1 | 1.7179354 | 2 | 50.567551 | 24.977222 | 72.589996 | 0 | 19 | 3 | 1 | 1 | 1 | 1 | 1 | 1 | 1 |  | NA |  | NA | 2 | 2 | 2 | 0 | 0 | 0 |  | 2 | 24 | 0 | 6.9000001 | 1 |  |
| 2305 | 0 | 20.686001 | 2.25 | 10.876921 | 89.123077 | 1 | 13.355236 | 1 | 1.7179354 | 2 | 50.567551 | 24.792778 | 74.32 | 0 | 20 | 3 | 1 | 0 | 0 | 0 | 0 | 0 | 0 |  | NA |  | NA | 2 | 2 | 2 | 0 | 0 | 0 |  | 2 | 24 | 0 |  | | |
| 2305 | 0 | 22.496 | 2.46 | 10.935277 | 89.06472 | 1 | 13.38809 | 1 | 1.7179354 | 2 | 50.567551 | 23.832224 | 72.730003 | 0 | 21 | 3 | 1 | 0 | 0 | 0 | 0 | 0 | 0 |  | NA |  | NA | 2 | 2 | 2 | 0 | 0 | 0 |  | 2 | 23 | 0 | 6.8000002 | 1 |  |
| 2347 |  | 26.627 | 3.553 | 13.343598 | 86.656403 | 0 | 9.8945923 | 0 | -0.0941329 | 4 | 92.051598 | 23.326666 | 72.27 | 0 | 0 | 3 | 1 |  | | | | | | 106.3435272 | 0.560359524 | 92.30945446 | -1.225751083 | 2 | 2 |  | 1 |  | 0 | 0 | 3 |  | | | | |
| 2347 | 1 | 18.233 | 1.91 | 10.475512 | 89.52449 | 0 | 10.861054 | 0 | -0.0941329 | 4 | 92.051598 | 25.131111 | 71.730003 | 0 | 19 | 3 | 1 | 1 | 1 | 1 | 1 | 1 |  | | NA |  | NA | 2 | 2 | 2 | 1 |  | 0 |  | 3 | 26 | 0 | 7 | 0 |  |
| 2347 | 1 | 21.07 | 2.3599999 | 11.200759 | 88.79924 | 0 | 10.899384 | 0 | -0.0941329 | 4 | 92.051598 | 24.59111 | 75.139999 | 0 | 20 | 3 | 1 | 1 | 1 | 1 | 1 | 1 |  | 122.085849 | 1.928736983 | 98.0237639 | -0.343129425 | 2 | 2 | 2 | 1 |  | 0 | 0 | 3 | 26 | 0 |  | | |
| 2347 | 1 | 22.987 | 2.5899999 | 11.267238 | 88.732765 | 0 | 10.937714 | 0 | -0.0941329 | 4 | 92.051598 | 24.237776 | 73 | 0 | 21 | 3 | 1 | 1 | 0 | 0 | 0 | 0 |  | | NA |  | NA | 2 | 2 | 2 | 1 |  | 0 |  | 3 | 22 | 0 | 6.0999999 | 1 |  |
| 2348 |  | 26.479 | 3.584 | 13.535254 | 86.464745 | 0 | 11.01985 | 1 | -1.8247317 | 2 | 116.28963 | 21.639999 | 71.143 | 0 | 0 | 3 | 1 |  | | | | | | 146.4379505 | 4.200842018 | 85.11456921 | -2.095092409 | 2 | 2 |  | 0 | 0 | 0 | 1 | 2 |  | | | | |
| 2348 | 1 | 18.459 | 1.83 | 9.9138632 | 90.086136 | 1 | 12.021903 | 1 | -1.8247317 | 2 | 116.28963 | 25.701111 | 68.489998 | 0 | 19 | 3 | 1 | 1 | 0 | 1 | 0 | 1 |  | | NA |  | NA | 2 | 2 | 2 | 0 | 0 | 0 |  | 2 | 25 | 0 | 7 | 0 |  |
| 2348 | 0 | 20.325001 | 2.2 | 10.824108 | 89.175896 | 1 | 12.041068 | 1 | -1.8247317 | 2 | 116.28963 | 25.319998 | 71.800003 | 0 | 20 | 3 | 1 | 0 | 0 | 0 | 0 | 0 | 0 | 131.7416927 | 2.891353514 | 91.27768315 | -1.299155591 | 2 | 2 | 2 | 0 | 0 | 0 | 0 | 2 | 27 | 0 |  | | |
| 2348 | 0 | 24.341999 | 2.5799999 | 10.598965 | 89.401031 | 1 | 12.098562 | 1 | -1.8247317 | 2 | 116.28963 | 24.775557 | 69.839996 | 0 | 21 | 3 | 1 | 0 | 0 | 0 | 0 | 0 | 0 |  | NA |  | NA | 2 | 2 | 2 | 0 | 0 | 0 |  | 2 | 25 | 0 | 7 | 0 |  |
| 2351 |  | 25.659 | 3.727 | 14.525118 | 85.474884 | 1 | 16.673512 | 0 | -2.4526858 | 2 | 107.55823 | 19.796667 | 73.414 | 0 | 0 | 2 | 0 |  | | | | | | 108.5498952 | 0.73047134 | 106.1496935 | 1.124681039 | 2 | 2 |  | 0 | 0 | 0 | 0 | 3 |  | | | | |
| 2351 | 1 | 17.438 | 1.74 | 9.9782085 | 90.02179 | 1 | 17.63176 | 0 | -2.4526858 | 2 | 107.55823 | 25.45389 | 68.470001 | 0 | 19 | 2 | 0 | 1 | 0 | 0 | 0 | 1 | 0 |  | NA |  | NA | 2 | 2 | 2 | 0 | 0 | 0 |  | 3 | 24 | 0 | 7 | 0 |  |
| 2351 | 0 | 24.577999 | 2.6600001 | 10.822688 | 89.177315 | 1 | 17.70842 | 0 | -2.4526858 | 2 | 107.55823 | 23.901669 | 70.940002 | 0 | 21 | 2 | 0 | 0 | 0 | 0 | 0 | 0 | 0 |  | NA |  | NA | 2 | 2 | 2 | 0 | 0 | 0 |  | 3 | 24 | 0 | 7 | 0 |  |
| 2387 |  | 24.819 | 3.413 | 13.751561 | 86.248436 | 0 | 11.266256 | 1 | -0.90703815 | 3 | 111.92879 | 22.955 | 71.157 | 0 | 0 | 2 | 0 |  | | | | | | 105.6122487 | 0.513061898 | 113.1696712 | 2.412473298 | 2 | 2 |  | 0 | 0 | 0 | 0 | 3 |  | | | | |
| 2387 | 0 | 18.910999 | 1.91 | 10.099942 | 89.900055 | 1 | 12.197125 | 1 | -0.90703815 | 3 | 111.92879 | 25.709444 | 68.309998 | 0 | 19 | 2 | 0 | 0 | 0 | 0 | 0 | 0 | 0 |  | NA |  | NA | 2 | 2 | 2 | 0 | 0 | 0 |  | 3 | 24 | 0 | 7 | 0 |  |
| 2387 | 1 | 24.576 | 2.8699999 | 11.67806 | 88.321938 | 1 | 12.273785 | 1 | -0.90703815 | 3 | 111.92879 | 24.370554 | 69.410004 | 0 | 21 | 2 | 0 | 1 | 1 | 0 | 1 | 1 | 0 |  | NA |  | NA | 2 | 2 | 2 | 0 | 0 | 0 |  | 3 | 24 | 0 | 7 | 0 |  |
| 2389 |  | 25.143 | 3.538 | 14.071511 | 85.92849 | 1 | 12.019165 | 1 | -0.79377419 | 2 | 130.40999 | 22.15 | 71.652 | 0 | 0 | 3 | 0 |  | | | | | | 132.6793551 | 2.975780313 | 105.0945936 | 0.860938384 | 2 | 2 |  | 0 | 0 | 0 | 0 | 4 |  | | | | |
| 2389 | 0 | 17.285 | 1.71 | 9.892971 | 90.107033 | 1 | 12.936345 | 1 | -0.79377419 | 2 | 130.40999 | 25.644447 | 68.480003 | 0 | 19 | 3 | 0 | 0 | 0 | 0 | 0 | 0 | 0 |  | NA |  | NA | 2 | 2 | 2 | 0 | 0 | 0 |  | 4 | 24 | 0 | 7 | 0 |  |
| 2389 | 1 | 20.921 | 2.27 | 10.850342 | 89.149658 | 1 | 12.99384 | 1 | -0.79377419 | 2 | 130.40999 | 25.230556 | 71 | 0 | 20 | 3 | 0 | 1 | 0 | 0 | 1 | 1 | 0 | 124.2621401 | 2.213450143 | 110.8434963 | 1.891182978 | 2 | 2 | 2 | 0 | 0 | 0 | 0 | 4 | 24 | 0 |  | | |
| 2389 | 0 | 25.282 | 2.8299999 | 11.193734 | 88.806267 | 1 | 13.01848 | 1 | -0.79377419 | 2 | 130.40999 | 24.156113 | 69.870003 | 0 | 21 | 3 | 0 | 0 | 0 | 0 | 0 | 0 | 0 |  | NA |  | NA | 2 | 2 | 2 | 0 | 0 | 0 |  | 4 | 25 | 0 | 7 | 0 |  |
| 2403 |  | 25.981 | 3.504 | 13.486779 | 86.513222 | 1 | 15.247091 | 1 | 1.5357243 | 2 | 116.07139 | 23.332777 | 75.627 | 0 | 0 | 3 | 1 |  | | | | | | 115.5119294 | 1.412541076 | 107.2691436 | 1.172396577 | 1 | 2 |  | 0 | 0 | 1 | 0 |  | | | | | |
| 2403 | 0 | 18.837999 | 2.0899999 | 11.094596 | 88.905403 | 1 | 16.167009 | 1 | 1.5357243 | 2 | 116.07139 | 24.331665 | 75.690002 | 0 | 19 | 3 | 1 | 0 | 0 | 0 | 0 | 0 | 0 |  | NA |  | NA | 1 | 2 | 2 | 0 | 0 | 1 |  | | 24 | 0 | 7 | 0 |  |
| 2403 | 0 | 21.417 | 2.6099999 | 12.186581 | 87.813423 | 1 | 16.2026 | 1 | 1.5357243 | 2 | 116.07139 | 24.301664 | 77.349998 | 0 | 20 | 3 | 1 | 0 | 0 | 0 | 0 | 0 | 0 | 112.1222308 | 1.102802437 | 104.343155 | 0.669587097 | 1 | 2 | 2 | 0 | 0 | 1 | 0 |  | 24 | 0 |  | | |
| 2403 | 0 | 23.892 | 2.75 | 11.510129 | 88.489868 | 1 | 16.24367 | 1 | 1.5357243 | 2 | 116.07139 | 22.967224 | 76.110001 | 0 | 21 | 3 | 1 | 0 | 0 | 0 | 0 | 0 | 0 |  | NA |  | NA | 1 | 2 | 2 | 0 | 0 | 1 |  | | 21 | 0 | 5.3000002 | 1 |  |
| 2452 |  | 21.767 | 2.06 | 9.4638672 | 90.536133 | 1 | 14.959617 | 1 | 0.39040497 | 4 | 82.202934 | 20.610001 | 78.482 | 0 | 0 | 2 | 1 |  | | | | | | 90.88885015 | -0.835350152 | 97.124763 | -0.429634205 | 2 | 2 |  | 1 | 0 | 0 | 1 | 4 |  | | | | |
| 2452 | 1 | 16.039 | 1.67 | 10.412121 | 89.587883 | 1 | 15.690623 | 1 | 0.39040497 | 4 | 82.202934 | 24.666668 | 74.110001 | 0 | 19 | 2 | 1 | 0 | 1 | 1 | 0 | 0 | 0 |  | NA |  | NA | 2 | 2 | 2 | 1 | 0 | 0 |  | 4 | 24 | 0 | 7 | 0 |  |
| 2452 | 1 | 19.518999 | 2.0899999 | 10.707516 | 89.29248 | 1 | 15.73169 | 1 | 0.39040497 | 4 | 82.202934 | 24.528889 | 76.68 | 0 | 20 | 2 | 1 | 1 | 0 | 0 | 0 | 1 | 0 | 116.5483743 | 1.504931466 | 92.77066078 | -1.024345741 | 2 | 2 | 2 | 1 | 0 | 0 | 1 | 4 | 13 | 1 |  | | |
| 2461 |  | 21.304 | 2.27 | 10.655276 | 89.344727 | 0 | 10.277892 | 1 | 2.3747432 | 4 | 85.477287 | 21.669445 | 75.146 | 0 | 0 | 3 | 0 |  | | | | | | 110.1807275 | 0.922000374 | 82.88504251 | -2.336458811 | 2 | 2 |  | 1 | 0 | 1 | 1 | 1 |  | | | | |
| 2461 | 0 | 18.311001 | 1.85 | 10.103216 | 89.896782 | 0 | 11.036277 | 1 | 2.3747432 | 4 | 85.477287 | 26.033335 | 66.790001 | 0 | 19 | 3 | 0 | 0 | 0 | 0 | 0 | 0 |  | | NA |  | NA | 2 | 2 | 2 | 1 | 0 | 1 |  | 1 | 27 | 0 | 7 | 0 |  |
| 2461 | 0 | 21.861 | 2.4200001 | 11.069942 | 88.930061 | 0 | 11.074607 | 1 | 2.3747432 | 4 | 85.477287 | 26.03389 | 68.239998 | 0 | 20 | 3 | 0 | 0 | 0 | 0 | 0 | 0 |  | 106.2190728 | 0.568526524 | 89.25052417 | -1.576795103 | 2 | 2 | 2 | 1 | 0 | 1 | 0 | 1 | 26 | 0 |  | | |
| 2461 | 1 | 24.767 | 2.78 | 11.224613 | 88.775383 | 0 | 11.112936 | 1 | 2.3747432 | 4 | 85.477287 | 24.042223 | 68.940002 | 0 | 21 | 3 | 0 | 1 | 0 | 1 | 1 | 1 |  | | NA |  | NA | 2 | 2 | 2 | 1 | 0 | 1 |  | 1 | 23 | 0 | 6.8000002 | 1 |  |
| 2486 |  | 25.483 | 3.962 | 15.54762 | 84.452377 | 1 | 12.183436 | 1 | -0.66808552 | 2 | 107.13612 | 13.669444 | 77.693 | 0 | 0 | 3 | 1 |  | | | | | | 119.3411186 | 1.766779359 | 95.79546156 | -0.652336371 | 2 | 2 |  | 1 |  | 1 | 0 | 2 |  | | | | |
| 2486 | 0 | 16.740999 | 1.66 | 9.9157763 | 90.084221 | 1 | 13.377139 | 1 | -0.66808552 | 2 | 107.13612 | 26.49222 | 64.870003 | 0 | 19 | 3 | 1 | 0 | 0 | 0 | 0 | 0 | 0 |  | NA |  | NA | 2 | 2 | 2 | 1 |  | 1 |  | 2 | 24 | 0 | 7 | 0 |  |
| 2486 | 1 | 20.829 | 2.1199999 | 10.178117 | 89.821884 | 1 | 13.418207 | 1 | -0.66808552 | 2 | 107.13612 | 26.006109 | 67.239998 | 0 | 20 | 3 | 1 | 1 | 0 | 0 | 0 | 0 | 0 |  | NA |  | NA | 2 | 2 | 2 | 1 |  | 1 |  | 2 | 17 | 1 |  | | |
| 2486 | 1 | 23.818001 | 2.6800001 | 11.251994 | 88.748009 | 1 | 13.456536 | 1 | -0.66808552 | 2 | 107.13612 | 25.832779 | 66.209999 | 0 | 21 | 3 | 1 | 1 | 0 | 0 | 0 | 0 | 0 |  | NA |  | NA | 2 | 2 | 2 | 1 |  | 1 |  | 2 | 13 | 1 | 4.6999998 | 1 |  |
| 2487 |  | 24.964 | 3.748 | 15.013619 | 84.986382 | 0 | 10.116359 | 1 | -0.1577317 | 4 | 110.06575 | 15.117222 | 76.791 | 0 | 0 | 3 | 1 |  | | | | | | 115.6647241 | 1.413816837 | 101.8272613 | 0.301674569 | 2 | 2 |  | 0 | 0 | 0 | 1 |  | | | | | |
| 2487 | 0 | 18.275999 | 1.84 | 10.067849 | 89.932152 | 0 | 11.359343 | 1 | -0.1577317 | 4 | 110.06575 | 25.996111 | 66.889999 | 0 | 19 | 3 | 1 | 0 | 0 | 0 | 0 | 0 |  | | NA |  | NA | 2 | 2 | 2 | 0 | 0 | 0 |  | | 27 | 0 | 7 | 0 |  |
| 2487 | 0 | 21.891001 | 2.4100001 | 11.00909 | 88.990906 | 0 | 11.397673 | 1 | -0.1577317 | 4 | 110.06575 | 25.987223 | 68.349998 | 0 | 20 | 3 | 1 | 0 | 0 | 0 | 0 | 0 |  | 91.30037633 | -0.79942144 | 103.881076 | 0.65177787 | 2 | 2 | 2 | 0 | 0 | 0 | 1 |  | 26 | 0 |  | | |
| 2487 | 0 | 24.735001 | 2.76 | 11.158278 | 88.841721 | 0 | 11.436003 | 1 | -0.1577317 | 4 | 110.06575 | 23.99 | 69.080002 | 0 | 21 | 3 | 1 | 0 | 0 | 0 | 0 | 0 |  | | NA |  | NA | 2 | 2 | 2 | 0 | 0 | 0 |  | | 26 | 0 | 7 | 0 |  |
| 2489 |  | 24.714 | 3.367 | 13.623857 | 86.376144 | 1 | 12.295688 | 0 | -1.1737348 | 2 | 98.471054 | 18.912777 | 73.802 | 0 | 0 | 2 |  | | | | | | | 103.3179268 | 0.292514586 | 101.9155152 | 0.349471104 | 2 | 2 |  | 0 | 0 | 0 | 0 |  | | | | | |
| 2489 | 0 | 17.693001 | 1.7 | 9.6083193 | 90.391678 | 1 | 13.511293 | 0 | -1.1737348 | 2 | 98.471054 | 25.823334 | 68.160004 | 0 | 19 | 2 |  | 0 | 0 | 0 | 0 | 0 | 0 |  | NA |  | NA | 2 | 2 | 2 | 0 | 0 | 0 |  | | 24 | 0 | 7 | 0 |  |
| 2489 | 0 | 20.174999 | 2.1700001 | 10.755887 | 89.24411 | 1 | 13.552361 | 0 | -1.1737348 | 2 | 98.471054 | 25.094999 | 71.260002 | 0 | 20 | 2 |  | 0 | 0 | 0 | 0 | 0 | 0 | 83.80808618 | -1.43531709 | 98.84737958 | -0.196925006 | 2 | 2 | 2 | 0 | 0 | 0 | 1 |  | 24 | 0 |  | | |
| 2490 |  | 25.119 | 3.25 | 12.938414 | 87.061584 | 1 | 16.509241 | 0 | -0.23286186 | 3 | 128.01762 | 21.752222 | 71.698 | 0 | 0 | 3 | 0 |  | | | | | | 134.8943705 | 2.923588713 | 101.5599386 | 0.269085374 | 2 | 2 |  | 1 | 0 | 0 | 0 |  | | | | | |
| 2490 | 0 | 19.089001 | 2.05 | 10.739168 | 89.260834 | 1 | 17.733059 | 0 | -0.23286186 | 3 | 128.01762 | 25.417221 | 70.160004 | 0 | 19 | 3 | 0 | 0 | 0 | 0 | 0 | 0 | 0 |  | NA |  | NA | 2 | 2 | 2 | 1 | 0 | 0 |  | | 24 | 0 | 7 | 0 |  |
| 2490 | 0 | 21.879 | 2.45 | 11.197952 | 88.802048 | 1 | 17.763176 | 0 | -0.23286186 | 3 | 128.01762 | 25.145 | 73.239998 | 0 | 20 | 3 | 0 | 0 | 0 | 0 | 0 | 0 | 0 | 133.1127004 | 2.762050731 | 96.47054615 | -0.56075809 | 2 | 2 | 2 | 1 | 0 | 0 | 0 |  | 24 | 0 |  | | |
| 2490 | 0 | 22.882999 | 2.6099999 | 11.405848 | 88.594154 | 1 | 17.79603 | 0 | -0.23286186 | 3 | 128.01762 | 24.779997 | 71.209999 | 0 | 21 | 3 | 0 | 0 | 0 | 0 | 0 | 0 | 0 |  | NA |  | NA | 2 | 2 | 2 | 1 | 0 | 0 |  | | 24 | 0 | 7 | 0 |  |
| 2492 |  | 25.163 | 3.402 | 13.519851 | 86.480148 | 1 | 12.279261 | 0 | 1.5357243 | 2 | 120.94055 | 20.633888 | 75.627 | 0 | 0 | 2 | 1 |  | | | | | | 129.8971006 | 2.581050891 | 99.85252702 | -0.026083563 | 2 | 2 |  | 1 | 0 | 0 | 0 | 4 |  | | | | |
| 2492 | 0 | 18.162001 | 1.96 | 10.791762 | 89.208237 | 1 | 13.50308 | 0 | 1.5357243 | 2 | 120.94055 | 24.794443 | 73.400002 | 0 | 19 | 2 | 1 | 0 | 0 | 0 | 0 | 0 | 0 |  | NA |  | NA | 2 | 2 | 2 | 1 | 0 | 0 |  | 4 | 24 | 0 | 7 | 0 |  |
| 2492 | 1 | 21.176001 | 2.4300001 | 11.475255 | 88.524742 | 1 | 13.54141 | 0 | 1.5357243 | 2 | 120.94055 | 24.172224 | 77.050003 | 0 | 20 | 2 | 1 | 1 | 0 | 0 | 0 | 1 | 0 | 113.155803 | 1.136098 | 98.94045301 | -0.181303534 | 2 | 2 | 2 | 1 | 0 | 0 | 0 | 4 | 24 | 0 |  | | |
| 2495 |  | 25.27 | 3.664 | 14.499407 | 85.500595 | 1 | 12.944558 | 0 | -1.0104688 | 3 | 112.55094 | 15.293889 | 78.043 | 0 | 0 | 2 |  | | | | | | | 112.7077729 | 1.106734123 | 106.9800354 | 1.361606603 | 2 | 2 |  | 0 | 0 | 0 | 0 | 4 |  | | | | |
| 2495 | 0 | 17.309999 | 1.71 | 9.8786831 | 90.121315 | 1 | 14.146475 | 0 | -1.0104688 | 3 | 112.55094 | 25.567225 | 68.279999 | 0 | 19 | 2 |  | 0 | 0 | 0 | 0 | 0 | 0 |  | NA |  | NA | 2 | 2 | 2 | 0 | 0 | 0 |  | 4 | 24 | 0 | 7 | 0 |  |
| 2495 | 0 | 23.426001 | 2.54 | 10.842653 | 89.157349 | 1 | 14.223135 | 0 | -1.0104688 | 3 | 112.55094 | 24.722221 | 70.629997 | 0 | 21 | 2 |  | 0 | 0 | 0 | 0 | 0 | 0 |  | NA |  | NA | 2 | 2 | 2 | 0 | 0 | 0 |  | 4 | 24 | 0 | 7 | 0 |  |
| 2497 |  | 26.818 | 3.028 | 11.290924 | 88.709076 | 1 | 15.939767 | 0 | -1.4420309 | 2 | 90.818832 | 20.049999 | 73.666 | 0 | 0 | 3 | 1 |  | | | | | | 118.5002823 | 1.573649216 | 82.37998015 | -2.349379676 | 2 | 2 |  | 0 | 0 | 1 | 1 | 1 |  | | | | |
| 2497 | 1 | 22.142 | 2.1800001 | 9.8455429 | 90.154457 | 1 | 17.152636 | 0 | -1.4420309 | 2 | 90.818832 | 26.386112 | 65.889999 | 0 | 19 | 3 | 1 | 1 | 1 | 1 | 1 | 1 | 0 |  | NA |  | NA | 2 | 2 | 2 | 0 | 0 | 1 |  | 1 | 24 | 0 | 7 | 0 |  |
| 2497 | 1 | 23.808001 | 2.29 | 9.6186152 | 90.381386 | 1 | 17.190966 | 0 | -1.4420309 | 2 | 90.818832 | 25.716112 | 69.199997 | 0 | 20 | 3 | 1 | 1 | 1 | 1 | 1 | 1 | 1 | 97.51395608 | -0.213886266 | 76.0876782 | -2.904399954 | 2 | 2 | 2 | 0 | 0 | 1 | 1 | 1 | 21 | 0 |  | | |
| 2497 | 1 | 24.399 | 2.5799999 | 10.574203 | 89.425797 | 1 | 17.232033 | 0 | -1.4420309 | 2 | 90.818832 | 25.344446 | 69.25 | 0 | 21 | 3 | 1 | 1 | 1 | 1 | 1 | 1 | 1 |  | NA |  | NA | 2 | 2 | 2 | 0 | 0 | 1 |  | 1 | 18 | 1 | 4.6999998 | 1 |  |
| 2498 |  | 26.526 | 3.335 | 12.57257 | 87.427429 | 0 | 10.614648 | 0 | 1.6736273 | 2 | 99.626198 | 20.081667 | 73.873 | 0 | 0 | 3 | 1 |  | | | | | | 105.0673877 | 0.449160028 | 101.6338927 | 0.301927457 | 2 | 2 |  | 0 | 0 | 1 | 0 |  | | | | | |
| 2498 | 0 | 19.190001 | 2 | 10.422094 | 89.577904 | 0 | 11.813827 | 0 | 1.6736273 | 2 | 99.626198 | 25.528332 | 69.389999 | 0 | 19 | 3 | 1 | 0 | 0 | 0 | 0 | 0 |  | | NA |  | NA | 2 | 2 | 2 | 0 | 0 | 1 |  | | 27 | 0 | 7 | 0 |  |
| 2498 | 1 | 22.683001 | 2.4000001 | 10.580611 | 89.419388 | 0 | 11.843943 | 0 | 1.6736273 | 2 | 99.626198 | 25.261667 | 72.620003 | 0 | 20 | 3 | 1 | 1 | 0 | 0 | 0 | 0 |  | 89.61749718 | -0.929269515 | 105.5661929 | 1.078866525 | 2 | 2 | 2 | 0 | 0 | 1 | 0 |  | 26 | 0 |  | | |
| 2498 | 0 | 23.503 | 2.6099999 | 11.104965 | 88.895035 | 0 | 11.885011 | 0 | 1.6736273 | 2 | 99.626198 | 24.637777 | 70.970001 | 0 | 21 | 3 | 1 | 0 | 0 | 0 | 0 | 0 |  | | NA |  | NA | 2 | 2 | 2 | 0 | 0 | 1 |  | | 27 | 0 | 7 | 0 |  |
| 2499 |  | 25.362 | 3.237 | 12.763189 | 87.236809 | 0 | 10.869267 | 0 | 1.9194371 | 2 | 105.91796 | 18.795 | 77.394 | 0 | 0 | 3 | 1 |  | | | | | | 106.7205685 | 0.594737828 | 105.7525054 | 1.131877363 | 2 | 2 |  | 0 | 0 | 1 | 0 | 4 |  | | | | |
| 2499 | 1 | 16.162001 | 1.89 | 11.694097 | 88.305901 | 1 | 12.084873 | 0 | 1.9194371 | 2 | 105.91796 | 24.893888 | 72.519997 | 0 | 19 | 3 | 1 | 1 | 1 | 1 | 0 | 1 | 1 |  | NA |  | NA | 2 | 2 | 2 | 0 | 0 | 1 |  | 4 | 24 | 0 | 7 | 0 |  |
| 2499 | 1 | 20.378 | 2.1700001 | 10.648739 | 89.351257 | 1 | 12.136892 | 0 | 1.9194371 | 2 | 105.91796 | 24.496113 | 75.93 | 0 | 20 | 3 | 1 | 0 | 1 | 1 | 0 | 1 | 0 | 97.1095466 | -0.256487279 | 109.3253625 | 1.905703491 | 2 | 2 | 2 | 0 | 0 | 1 | 0 | 4 | 24 | 0 |  | | |
| 2499 | 0 | 21.834 | 2.4100001 | 11.037831 | 88.962166 | 1 | 12.175222 | 0 | 1.9194371 | 2 | 105.91796 | 23.688334 | 73.660004 | 0 | 21 | 3 | 1 | 0 | 0 | 0 | 0 | 0 | 0 |  | NA |  | NA | 2 | 2 | 2 | 0 | 0 | 1 |  | 4 | 25 | 0 | 7 | 0 |  |
| 2500 |  | 24.191 | 1.881 | 7.775619 | 92.22438 | 1 | 16.465435 | 0 | -2.5146148 | 3 | 122.24088 | 22.445555 | 73.366 | 0 | 0 | 3 | 1 |  | | | | | | 129.3540887 | 2.469815335 | 101.1841751 | 0.203354785 | 2 | 2 |  | 0 | 0 | 0 | 0 | 3 |  | | | | |
| 2500 | 0 | 19.559999 | 1.91 | 9.7648268 | 90.235176 | 1 | 17.675564 | 0 | -2.5146148 | 3 | 122.24088 | 25.876112 | 68.790001 | 0 | 19 | 3 | 1 | 0 | 0 | 0 | 0 | 0 | 0 |  | NA |  | NA | 2 | 2 | 2 | 0 | 0 | 0 |  | 3 | 24 | 0 | 7 | 0 |  |
| 2500 | 1 | 22.164 | 2.25 | 10.151597 | 89.848404 | 1 | 17.724846 | 0 | -2.5146148 | 3 | 122.24088 | 25.72278 | 70.080002 | 0 | 20 | 3 | 1 | 1 | 1 | 1 | 1 | 0 | 1 | 132.205573 | 2.688751384 | 94.3927941 | -0.867495786 | 2 | 2 | 2 | 0 | 0 | 0 | 0 | 3 | 20 | 0 |  | | |
| 2500 | 0 | 34.051998 | 2.8699999 | 8.4282866 | 91.571716 | 1 | 17.752224 | 0 | -2.5146148 | 3 | 122.24088 | 24.121111 | 69.830002 | 0 | 21 | 3 | 1 | 0 | 0 | 0 | 0 | 0 | 0 |  | NA |  | NA | 2 | 2 | 2 | 0 | 0 | 0 |  | 3 | 25 | 0 | 7 | 0 |  |
| 2508 |  | 27.39 | 3.444 | 12.573932 | 87.426071 | 0 | 10.625599 | 1 | -1.5018775 | 4 | 101.11298 | 23.174999 | 72.732 | 0 | 0 | 3 | 1 |  | | | | | | 124.4294417 | 2.213274221 | 85.28009303 | -2.068518442 |  | | | | | 0 | 1 | 1 |  | | | | |
| 2508 | 0 | 21.663 | 2.21 | 10.201727 | 89.798271 | 0 | 11.813827 | 1 | -1.5018775 | 4 | 101.11298 | 25.974443 | 68.410004 | 0 | 19 | 3 | 1 | 0 | 0 | 0 | 0 | 0 |  | | NA |  | NA |  | | 2 |  | | 0 |  | 1 | 26 | 0 | 7 | 0 |  |
| 2508 | 1 | 22.834999 | 2.4400001 | 10.685352 | 89.314644 | 0 | 11.852156 | 1 | -1.5018775 | 4 | 101.11298 | 26.00889 | 70.709999 | 0 | 20 | 3 | 1 | 1 | 1 | 1 | 1 | 1 |  | 144.9953382 | 4.085688401 | 77.9974456 | -2.891444057 |  | | 2 |  | | 0 | 1 | 1 | 24 | 0 |  | | |
| 2508 | 0 | 25.01 | 2.7 | 10.795682 | 89.204315 | 0 | 11.890486 | 1 | -1.5018775 | 4 | 101.11298 | 25.366669 | 68.900002 | 0 | 21 | 3 | 1 | 0 | 0 | 0 | 0 | 0 | 0 |  | NA |  | NA |  | | 2 |  | | 0 |  | 1 | 27 | 0 | 7 | 0 |  |
| 2509 |  | 26.472 | 3.392 | 12.813539 | 87.186462 | 0 | 9.327858 | 1 | 0.88414335 | 4 | 113.90351 | 19.121666 | 75.234 | 0 | 0 | 3 |  | | | | | | | 133.9522841 | 3.01207397 | 90.92883574 | -1.326828841 | 2 | 2 |  | 0 | 0 | 0 | 0 | 3 |  | | | | |
| 2509 | 1 | 18.393 | 1.97 | 10.710596 | 89.289406 | 0 | 10.428473 | 1 | 0.88414335 | 4 | 113.90351 | 25.592224 | 69.809998 | 0 | 19 | 3 |  | 1 | 1 | 1 | 1 | 1 |  | | NA |  | NA | 2 | 2 | 2 | 0 | 0 | 0 |  | 3 | 27 | 0 | 7 | 0 |  |
| 2509 | 1 | 21.686001 | 2.3699999 | 10.928709 | 89.071289 | 0 | 10.466804 | 1 | 0.88414335 | 4 | 113.90351 | 25.398335 | 71.330002 | 0 | 20 | 3 |  | 1 | 1 | 1 | 1 | 1 |  | 116.1209159 | 1.461060529 | 83.56443354 | -2.265479228 | 2 | 2 | 2 | 0 | 0 | 0 | 0 | 3 | 19 | 1 |  | | |
| 2509 | 0 | 23.976 | 2.5699999 | 10.719052 | 89.280945 | 0 | 10.505134 | 1 | 0.88414335 | 4 | 113.90351 | 24.296667 | 70.110001 | 0 | 21 | 3 |  | 0 | 0 | 0 | 0 | 0 |  | | NA |  | NA | 2 | 2 | 2 | 0 | 0 | 0 |  | 3 | 27 | 0 | 7 | 0 |  |
| 2514 |  | 28.271 | 4.28 | 15.139189 | 84.860809 | 1 | 17.065023 | 1 | 1.3507731 | 2 | 65.761681 | 23.543888 | 67.43 | 0 | 0 | 3 | 1 |  | | | | | | 84.81277411 | -1.390801328 | 83.19264471 | -2.167346838 | 2 | 2 |  | 1 | 0 | 0 | 0 | 1 |  | | | | |
| 2514 | 0 | 16.375999 | 1.6799999 | 10.258916 | 89.741081 | 1 | 18.162903 | 1 | 1.3507731 | 2 | 65.761681 | 25.457777 | 69.879997 | 0 | 19 | 3 | 1 | 0 | 0 | 0 | 0 | 0 | 0 |  | NA |  | NA | 2 | 2 | 2 | 1 | 0 | 0 |  | 1 | 24 | 0 | 7 | 0 |  |
| 2514 | 1 | 18.497999 | 2.01 | 10.86604 | 89.133957 | 1 | 18.206707 | 1 | 1.3507731 | 2 | 65.761681 | 24.922777 | 72.540001 | 0 | 20 | 3 | 1 | 1 | 1 | 0 | 1 | 1 | 0 | 82.85401047 | -1.569269101 | 74.73286077 | -2.996079514 | 2 | 2 | 2 | 1 | 0 | 0 | 1 | 1 | 20 | 0 |  | | |
| 2514 | 1 | 25.285 | 2.8299999 | 11.192406 | 88.807594 | 1 | 18.247776 | 1 | 1.3507731 | 2 | 65.761681 | 24.598888 | 70.769997 | 0 | 21 | 3 | 1 | 1 | 1 | 1 | 1 | 1 | 1 |  | NA |  | NA | 2 | 2 | 2 | 1 | 0 | 0 |  | 1 | 20 | 0 | 5.6999998 | 1 |  |
| 2516 |  | 26.986 | 4.279 | 15.85637 | 84.143631 | 1 | 13.738535 | 0 | -0.009923273 | 2 | 113.22247 | 21.452778 | 71.778 | 0 | 0 | 3 |  | | | | | | | 120.8024973 | 1.790108697 | 100.2233914 | 0.039100032 | 2 | 2 |  | 0 | 0 | 0 | 0 |  | | | | | |
| 2516 | 0 | 16.474001 | 1.66 | 10.076484 | 89.923515 | 1 | 14.830937 | 0 | -0.009923273 | 2 | 113.22247 | 24.780558 | 72.519997 | 0 | 19 | 3 |  | 0 | 0 | 0 | 0 | 0 | 0 |  | NA |  | NA | 2 | 2 | 2 | 0 | 0 | 0 |  | | 24 | 0 | 7 | 0 |  |
| 2516 | 1 | 19.406 | 2.0799999 | 10.718334 | 89.28167 | 1 | 14.866529 | 0 | -0.009923273 | 2 | 113.22247 | 23.893335 | 76.110001 | 0 | 20 | 3 |  | 1 | 0 | 0 | 0 | 1 | 0 | 113.83852 | 1.188067355 | 101.7959501 | 0.316888931 | 2 | 2 | 2 | 0 | 0 | 0 | 0 |  | 23 | 0 |  | | |
| 2516 | 1 | 23.482 | 2.5899999 | 11.029724 | 88.970276 | 1 | 14.90486 | 0 | -0.009923273 | 2 | 113.22247 | 24.146666 | 74.099998 | 0 | 21 | 3 |  | 1 | 0 | 0 | 1 | 1 | 0 |  | NA |  | NA | 2 | 2 | 2 | 0 | 0 | 0 |  | | 24 | 0 | 7 | 0 |  |
| 2519 |  | 28.629 | 3.605 | 12.592127 | 87.407875 | 0 | 10.160164 | 0 | 0.82386881 | 3 | 99.090927 | 23.733334 | 66.75 | 0 | 0 | 3 |  | | | | | | | 111.1589054 | 0.983148082 | 94.14549364 | -0.961052059 | 1 | 1 |  | 0 | 0 | 1 | 0 | 3 |  | | | | |
| 2519 | 0 | 15.641 | 1.67 | 10.677067 | 89.322937 | 0 | 11.154004 | 0 | 0.82386881 | 3 | 99.090927 | 24.680002 | 74.239998 | 0 | 19 | 3 |  | 0 | 0 | 0 | 0 | 0 |  | | NA |  | NA | 1 | 1 | 2 | 0 | 0 | 1 |  | 3 | 27 | 0 | 7 | 0 |  |
| 2519 | 0 | 17.773001 | 1.9 | 10.690372 | 89.309631 | 0 | 11.192334 | 0 | 0.82386881 | 3 | 99.090927 | 23.891665 | 78.489998 | 0 | 20 | 3 |  | 0 | 0 | 0 | 0 | 0 |  | 87.05106085 | -1.164369349 | 106.2919916 | 1.239894216 | 1 | 1 | 2 | 0 | 0 | 1 | 1 | 3 | 27 | 0 |  | | |
| 2519 | 0 | 20.006001 | 2.1099999 | 10.546835 | 89.453163 | 0 | 11.230664 | 0 | 0.82386881 | 3 | 99.090927 | 23.925556 | 75.57 | 0 | 21 | 3 |  | 0 | 0 | 0 | 0 | 0 |  | | NA |  | NA | 1 | 1 | 2 | 0 | 0 | 1 |  | 3 | 25 | 0 | 6.9000001 | 1 |  |
| 2520 |  | 28.937 | 4.272 | 14.763106 | 85.236893 | 1 | 13.229295 | 0 | 0.49391067 | 3 | 123.42162 | 17.946112 | 73.711 | 0 | 0 | 3 | 1 |  | | | | | | 132.4926274 | 2.781853463 | 99.75932279 | -0.042078221 | 2 | 2 |  | 0 | 0 | 0 | 0 | 3 |  | | | | |
| 2520 | 0 | 18.283001 | 1.71 | 9.3529501 | 90.647049 | 1 | 14.318959 | 0 | 0.49391067 | 3 | 123.42162 | 25.797775 | 69.610001 | 0 | 19 | 3 | 1 | 0 | 0 | 0 | 0 | 0 | 0 |  | NA |  | NA | 2 | 2 | 2 | 0 | 0 | 0 |  | 3 | 24 | 0 | 7 | 0 |  |
| 2520 | 1 | 19.288 | 1.99 | 10.317296 | 89.682701 | 1 | 14.357289 | 0 | 0.49391067 | 3 | 123.42162 | 25.683891 | 71.629997 | 0 | 20 | 3 | 1 | 1 | 0 | 1 | 0 | 1 | 0 | 137.6544619 | 3.185601353 | 100.0514029 | 0.008905207 | 2 | 2 | 2 | 0 | 0 | 0 | 0 | 3 | 24 | 0 |  | | |
| 2520 | 0 | 21.618 | 2.3199999 | 10.731797 | 89.268204 | 1 | 14.392881 | 0 | 0.49391067 | 3 | 123.42162 | 24.993332 | 69.830002 | 0 | 21 | 3 | 1 | 0 | 0 | 0 | 0 | 0 | 0 |  | NA |  | NA | 2 | 2 | 2 | 0 | 0 | 0 |  | 3 | 24 | 0 | 7 | 0 |  |
| 2524 |  | 28.321 | 3.862 | 13.636524 | 86.36348 | 1 | 12.027378 | 0 | -1.5018775 | 4 | 94.546974 | 20.254444 | 72.331 | 0 | 0 | 3 | 1 |  | | | | | | 108.8548359 | 0.778350555 | 90.82875445 | -1.415570472 |  | | | | | 0 | 1 | 2 |  | | | | |
| 2524 | 1 | 17.455 | 1.87 | 10.713263 | 89.286736 | 1 | 13.092402 | 0 | -1.5018775 | 4 | 94.546974 | 25.902224 | 69.07 | 0 | 19 | 3 | 1 | 1 | 1 | 1 | 0 | 1 | 1 |  | NA |  | NA |  | | 2 |  | | 0 |  | 2 | 24 | 0 | 7 | 0 |  |
| 2524 | 0 | 20.618 | 2.26 | 10.961296 | 89.038704 | 1 | 13.130733 | 0 | -1.5018775 | 4 | 94.546974 | 25.608889 | 70.010002 | 0 | 20 | 3 | 1 | 0 | 0 | 0 | 0 | 0 | 0 | 104.1029792 | 0.358036542 | 97.60227515 | -0.404236786 |  | | 2 |  | | 0 | 0 | 2 | 24 | 0 |  | | |
| 2524 | 0 | 23.219999 | 2.53 | 10.89578 | 89.104218 | 1 | 13.149898 | 0 | -1.5018775 | 4 | 94.546974 | 24.478333 | 69.110001 | 0 | 21 | 3 | 1 | 0 | 0 | 0 | 0 | 0 | 0 |  | NA |  | NA |  | | 2 |  | | 0 |  | 2 | 24 | 0 | 7 | 0 |  |
| 2526 |  | 27.094 | 3.246 | 11.980513 | 88.019485 | 0 | 10.825462 | 1 | 0.9477421 | 4 | 81.889526 | 22.705 | 71.205 | 0 | 0 | 3 | 1 |  | | | | | | 77.18191147 | -2.09647685 | 113.7036189 | 2.5313895 |  | | | 0 | 0 | 0 | 0 | 3 |  | | | | |
| 2526 | 1 | 18.566999 | 1.86 | 10.017774 | 89.982224 | 0 | 11.674195 | 1 | 0.9477421 | 4 | 81.889526 | 25.569445 | 69.669998 | 0 | 19 | 3 | 1 | 1 | 0 | 0 | 0 | 0 |  | | NA |  | NA |  | | 2 | 0 | 0 | 0 |  | 3 | 27 | 0 | 7 | 0 |  |
| 2526 | 1 | 20.952999 | 2.24 | 10.690594 | 89.309402 | 0 | 11.712525 | 1 | 0.9477421 | 4 | 81.889526 | 25.151669 | 72.669998 | 0 | 20 | 3 | 1 | 1 | 0 | 0 | 0 | 0 |  | 75.93577815 | -2.223960167 | 111.5537859 | 2.056189464 |  | | 2 | 0 | 0 | 0 | 0 | 3 | 27 | 0 |  | | |
| 2526 | 0 | 24.15 | 2.6199999 | 10.848861 | 89.151138 | 0 | 11.750855 | 1 | 0.9477421 | 4 | 81.889526 | 24.674444 | 70.839996 | 0 | 21 | 3 | 1 | 0 | 0 | 0 | 0 | 0 |  | | NA |  | NA |  | | 2 | 0 | 0 | 0 |  | 3 | 27 | 0 | 7 | 0 |  |
| 2529 |  | 28.638 | 3.945 | 13.775403 | 86.224594 | 1 | 17.38809 | 1 | 0.23588657 | 2 | 110.91911 | 22.38611 | 73.935 | 0 | 0 | 3 | 1 |  | | | | | | 116.1981131 | 1.470982241 | 99.51022313 | -0.071879875 | 2 | 2 |  | 0 | 0 | 1 | 0 | 4 |  | | | | |
| 2529 | 0 | 18.856001 | 2.1099999 | 11.190071 | 88.809929 | 1 | 18.447639 | 1 | 0.23588657 | 2 | 110.91911 | 24.171112 | 76.5 | 0 | 19 | 3 | 1 | 0 | 0 | 0 | 0 | 0 | 0 |  | NA |  | NA | 2 | 2 | 2 | 0 | 0 | 1 |  | 4 | 24 | 0 | 7 | 0 |  |
| 2529 | 1 | 21.201 | 2.6099999 | 12.31074 | 87.689262 | 1 | 18.485968 | 1 | 0.23588657 | 2 | 110.91911 | 24.069443 | 78.07 | 0 | 20 | 3 | 1 | 1 | 0 | 0 | 0 | 1 | 0 | 100.56718 | 0.051646528 | 111.1444486 | 1.764026171 | 2 | 2 | 2 | 0 | 0 | 1 | 0 | 4 | 25 | 0 |  | | |
| 2529 | 0 | 24.107 | 2.8 | 11.614883 | 88.385117 | 1 | 18.516085 | 1 | 0.23588657 | 2 | 110.91911 | 22.786667 | 76.900002 | 0 | 21 | 3 | 1 | 0 | 0 | 0 | 0 | 0 | 0 |  | NA |  | NA | 2 | 2 | 2 | 0 | 0 | 1 |  | 4 | 25 | 0 | 7 | 0 |  |
| 2530 |  | 29.286 | 3.638 | 12.422318 | 87.577682 | 1 | 17.900068 | 0 | -0.41591239 | 3 | 105.80234 | 21.858889 | 71.328 | 0 | 0 | 3 | 0 |  | | | | | | 112.5879321 | 1.066069083 | 100.5241253 | 0.087638959 | 2 | 2 |  | 0 |  | 0 | 0 | 3 |  | | | | |
| 2530 | 0 | 19.069 | 2 | 10.488227 | 89.511772 | 1 | 18.951403 | 0 | -0.41591239 | 3 | 105.80234 | 25.496111 | 69.589996 | 0 | 19 | 3 | 0 | 0 | 0 | 0 | 0 | 0 | 0 |  | NA |  | NA | 2 | 2 | 2 | 0 |  | 0 |  | 3 | 24 | 0 | 7 | 0 |  |
| 2530 | 0 | 22.575001 | 2.3900001 | 10.586932 | 89.413071 | 1 | 18.98152 | 0 | -0.41591239 | 3 | 105.80234 | 25.231112 | 72.839996 | 0 | 20 | 3 | 0 | 0 | 0 | 0 | 0 | 0 | 0 | 107.0317498 | 0.595086897 | 93.41684675 | -0.9894321 | 2 | 2 | 2 | 0 |  | 0 | 0 | 3 | 24 | 0 |  | | |
| 2530 | 1 | 23.327 | 2.5899999 | 11.103013 | 88.896988 | 1 | 19.022587 | 0 | -0.41591239 | 3 | 105.80234 | 24.627779 | 71.129997 | 0 | 21 | 3 | 0 | 1 | 1 | 1 | 1 | 0 |  | | NA |  | NA | 2 | 2 | 2 | 0 |  | 0 |  | 3 | 25 | 0 | 7 | 0 |  |
| 2533 |  | 26.897 | 4.286 | 15.934863 | 84.06514 | 1 | 13.09514 | 1 | -0.70106024 | 2 | 99.534782 | 22.333889 | 70.919 | 0 | 0 | 3 | 1 |  | | | | | | 106.7378608 | 0.617172939 | 99.58396706 | -0.065820883 | 2 | 2 |  | 0 | 0 | 0 | 0 | 3 |  | | | | |
| 2533 | 0 | 17.41 | 1.78 | 10.22401 | 89.775993 | 1 | 14.154689 | 1 | -0.70106024 | 2 | 99.534782 | 24.368887 | 73.809998 | 0 | 19 | 3 | 1 | 0 | 0 | 0 | 0 | 0 | 0 |  | NA |  | NA | 2 | 2 | 2 | 0 | 0 | 0 |  | 3 | 24 | 0 | 7 | 0 |  |
| 2533 | 0 | 21.743999 | 2.4200001 | 11.129508 | 88.870491 | 1 | 14.201232 | 1 | -0.70106024 | 2 | 99.534782 | 24.142223 | 75.470001 | 0 | 20 | 3 | 1 | 0 | 0 | 0 | 0 | 0 | 0 | 97.35070347 | -0.242816958 | 109.6003525 | 1.613327945 | 2 | 2 | 2 | 0 | 0 | 0 | 0 | 3 | 24 | 0 |  | | |
| 2533 | 0 | 25.740999 | 2.8499999 | 11.071831 | 88.928169 | 1 | 14.236824 | 1 | -0.70106024 | 2 | 99.534782 | 23.066666 | 74.209999 | 0 | 21 | 3 | 1 | 0 | 0 | 0 | 0 | 0 | 0 |  | NA |  | NA | 2 | 2 | 2 | 0 | 0 | 0 |  | 3 | 25 | 0 | 7 | 0 |  |
| 2534 |  | 28.341 | 3.854 | 13.598673 | 86.401329 | 1 | 12.605065 | 0 | 1.9830359 | 2 | 97.069534 | 20.411667 | 72.121 | 0 | 0 | 3 | 1 |  | | | | | | 108.5876701 | 0.752170657 | 95.23855003 | -0.782966032 | 2 | 2 |  | 0 | 0 | 0 | 0 | 2 |  | | | | |
| 2534 | 0 | 17.033001 | 1.77 | 10.391592 | 89.608406 | 1 | 13.653662 | 0 | 1.9830359 | 2 | 97.069534 | 25.904999 | 68.400002 | 0 | 19 | 3 | 1 | 0 | 0 | 0 | 0 | 0 | 0 |  | NA |  | NA | 2 | 2 | 2 | 0 | 0 | 0 |  | 2 | 24 | 0 | 7 | 0 |  |
| 2534 | 1 | 19.914 | 2.1600001 | 10.846641 | 89.153358 | 1 | 13.691992 | 0 | 1.9830359 | 2 | 97.069534 | 25.656666 | 71.169998 | 0 | 20 | 3 | 1 | 1 | 0 | 1 | 1 | 1 | 0 | 108.4260358 | 0.732299813 | 103.6093813 | 0.663348078 | 2 | 2 | 1 | 0 | 0 | 0 | 0 | 2 | 9 | 1 |  | | |
| 2534 | 0 | 22.07 | 2.4100001 | 10.919801 | 89.0802 | 1 | 13.730322 | 0 | 1.9830359 | 2 | 97.069534 | 24.874445 | 69.370003 | 0 | 21 | 3 | 1 | 0 | 0 | 0 | 0 | 0 | 0 |  | NA |  | NA | 2 | 2 | 1 | 0 | 0 | 0 |  | 2 | 14 | 1 | 6.0999999 | 1 |  |
| 2535 |  | 28.996 | 3.654 | 12.601738 | 87.398262 | 1 | 16.36961 | 1 | 1.9194371 | 3 | 108.65406 | 22.402779 | 70.783 | 0 | 0 | 3 | 1 |  | | | | | | 103.8620681 | 0.352119166 | 111.888209 | 1.946679363 | 2 | 2 |  | 0 | 0 | 0 | 0 | 3 |  | | | | |
| 2535 | 0 | 19.347 | 2.0799999 | 10.75102 | 89.248978 | 1 | 17.409992 | 1 | 1.9194371 | 3 | 108.65406 | 25.431667 | 70.68 | 0 | 19 | 3 | 1 | 0 | 0 | 0 | 0 | 0 | 0 |  | NA |  | NA | 2 | 2 | 2 | 0 | 0 | 0 |  | 3 | 24 | 0 | 7 | 0 |  |
| 2535 | 0 | 22.962 | 2.55 | 11.105305 | 88.894699 | 1 | 17.448322 | 1 | 1.9194371 | 3 | 108.65406 | 25.072777 | 71.199997 | 0 | 20 | 3 | 1 | 0 | 0 | 0 | 0 | 0 | 0 | 94.71165969 | -0.482587188 | 111.6895095 | 1.870990013 | 2 | 2 | 2 | 0 | 0 | 0 | 0 | 3 | 24 | 0 |  | | |
| 2535 | 0 | 25.108 | 2.73 | 10.873029 | 89.126968 | 1 | 17.486652 | 1 | 1.9194371 | 3 | 108.65406 | 23.972223 | 70.800003 | 0 | 21 | 3 | 1 | 0 | 0 | 0 | 0 | 0 | 0 |  | NA |  | NA | 2 | 2 | 2 | 0 | 0 | 0 |  | 3 | 24 | 0 | 7 | 0 |  |
| 2536 |  | 28.576 | 3.687 | 12.902435 | 87.097565 | 0 | 10.012321 | 0 | 1.5357243 | 3 | 109.35685 | 23.991112 | 69.991 | 0 | 0 | 3 | 1 |  | | | | | | 118.3602112 | 1.607286095 | 98.45041066 | -0.273175526 | 2 | 2 |  | 0 | 0 | 0 | 0 | 4 |  | | | | |
| 2536 | 0 | 20.013 | 2.0699999 | 10.343276 | 89.656723 | 0 | 11.025325 | 0 | 1.5357243 | 3 | 109.35685 | 25.744442 | 69.099998 | 0 | 19 | 3 | 1 | 0 | 0 | 0 | 0 | 0 |  | | NA |  | NA | 2 | 2 | 2 | 0 | 0 | 0 |  | 4 | 27 | 0 | 7 | 0 |  |
| 2536 | 0 | 22.221001 | 2.3900001 | 10.755591 | 89.244408 | 0 | 11.058179 | 0 | 1.5357243 | 3 | 109.35685 | 25.405558 | 72.419998 | 0 | 20 | 3 | 1 | 0 | 0 | 0 | 0 | 0 |  | 103.2763212 | 0.289734949 | 102.6115189 | 0.484186408 | 2 | 2 | 2 | 0 | 0 | 0 | 0 | 4 | 27 | 0 |  | | |
| 2536 | 0 | 23.938 | 2.6300001 | 10.986716 | 89.013283 | 0 | 11.110198 | 0 | 1.5357243 | 3 | 109.35685 | 25.093332 | 70.080002 | 0 | 21 | 3 | 1 | 0 | 0 | 0 | 0 | 0 |  | | NA |  | NA | 2 | 2 | 2 | 0 | 0 | 0 |  | 4 | 27 | 0 | 7 | 0 |  |
| 2537 |  | 28.098 | 3.784 | 13.467151 | 86.532852 | 1 | 12.43258 | 1 | 1.6736273 | 2 | 81.432762 | 21.706112 | 70.911 | 0 | 0 | 3 |  | | | | | | | 78.86859593 | -1.951209759 | 107.6811667 | 1.318931305 | 2 | 2 |  | 0 | 0 | 0 | 1 | 4 |  | | | | |
| 2537 | 0 | 17.132999 | 1.75 | 10.214207 | 89.785789 | 1 | 13.434634 | 1 | 1.6736273 | 2 | 81.432762 | 25.716667 | 69.379997 | 0 | 19 | 3 |  | 0 | 0 | 0 | 0 | 0 | 0 |  | NA |  | NA | 2 | 2 | 2 | 0 | 0 | 0 |  | 4 | 24 | 0 | 7 | 0 |  |
| 2537 | 1 | 19.657 | 2.0899999 | 10.632344 | 89.367653 | 1 | 13.475701 | 1 | 1.6736273 | 2 | 81.432762 | 25.351114 | 72.25 | 0 | 20 | 3 |  | 1 | 0 | 1 | 1 | 1 | 0 | 71.58151158 | -2.625774698 | 101.5826816 | 0.250789884 | 2 | 2 | 2 | 0 | 0 | 0 | 0 | 4 | 19 | 1 |  | | |
| 2537 | 1 | 21.677 | 2.3599999 | 10.887115 | 89.112885 | 1 | 13.514031 | 1 | 1.6736273 | 2 | 81.432762 | 24.78611 | 70.360001 | 0 | 21 | 3 |  | 1 | 0 | 1 | 0 | 0 | 0 |  | NA |  | NA | 2 | 2 | 2 | 0 | 0 | 0 |  | 4 | 24 | 0 | 7 | 0 |  |
| 2538 |  | 26.843 | 3.94 | 14.677942 | 85.32206 | 1 | 14.959617 | 1 | -1.9238331 | 2 | 110.09638 | 21.35 | 70.946 | 0 | 0 | 3 | 1 |  | | | | | | 124.3857223 | 2.21730116 | 95.08598383 | -0.721960559 | 2 | 2 |  | 0 | 0 | 0 | 0 | 2 |  | | | | |
| 2538 | 0 | 17.582001 | 1.74 | 9.8964844 | 90.103516 | 1 | 15.953457 | 1 | -1.9238331 | 2 | 110.09638 | 25.555 | 68.800003 | 0 | 19 | 3 | 1 | 0 | 0 | 0 | 0 | 0 | 0 |  | NA |  | NA | 2 | 2 | 2 | 0 | 0 | 0 |  | 2 | 24 | 0 | 7 | 0 |  |
| 2538 | 0 | 20.589001 | 2.23 | 10.831026 | 89.168976 | 1 | 15.991786 | 1 | -1.9238331 | 2 | 110.09638 | 25.187222 | 71.839996 | 0 | 20 | 3 | 1 | 0 | 0 | 0 | 0 | 0 | 0 | 113.8978469 | 1.264571166 | 83.89392304 | -2.116673925 | 2 | 2 | 2 | 0 | 0 | 0 | 0 | 2 | 25 | 0 |  | | |
| 2538 | 0 | 24.174999 | 2.71 | 11.209929 | 88.79007 | 1 | 16.043806 | 1 | -1.9238331 | 2 | 110.09638 | 24.411667 | 70.300003 | 0 | 21 | 3 | 1 | 0 | 0 | 0 | 0 | 0 | 0 |  | NA |  | NA | 2 | 2 | 2 | 0 | 0 | 0 |  | 2 | 24 | 0 | 7 | 0 |  |
| 2539 |  | 26.375 | 3.948 | 14.96872 | 85.031281 | 0 | 9.0130053 | 1 | -2.7576251 | 2 | 130.10291 | 19.140556 | 72.145 | 0 | 0 | 3 | 1 |  | | | | | | 139.6642555 | 3.491920569 | 102.039645 | 0.33452765 | 2 | 2 |  | 0 | 0 | 0 | 0 | 4 |  | | | | |
| 2539 | 0 | 18.190001 | 1.83 | 10.060472 | 89.939529 | 0 | 10.006845 | 1 | -2.7576251 | 2 | 130.10291 | 26.074999 | 66.519997 | 0 | 19 | 3 | 1 | 0 | 0 | 0 | 0 | 0 |  | | NA |  | NA | 2 | 2 | 2 | 0 | 0 | 0 |  | 4 | 23 | 0 | 7 | 0 |  |
| 2539 | 1 | 21.014999 | 2.21 | 10.516298 | 89.483704 | 0 | 10.02601 | 1 | -2.7576251 | 2 | 130.10291 | 25.956667 | 69.410004 | 0 | 20 | 3 | 1 | 1 | 0 | 1 | 1 | 0 |  | 128.8335652 | 2.59984341 | 100.2688094 | 0.04374309 | 2 | 2 | 2 | 0 | 0 | 0 | 0 | 4 | 19 | 1 |  | | |
| 2539 | 0 | 23.781 | 2.6500001 | 11.143351 | 88.856651 | 0 | 10.080767 | 1 | -2.7576251 | 2 | 130.10291 | 24.842779 | 68.190002 | 0 | 21 | 3 | 1 | 0 | 0 | 0 | 0 | 0 |  | | NA |  | NA | 2 | 2 | 2 | 0 | 0 | 0 |  | 4 | 23 | 0 | 7 | 0 |  |
| 2540 |  | 26.332 | 3.621 | 13.751329 | 86.248672 | 1 | 16.596851 | 1 | 1.4721255 | 2 | 108.05199 | 22.416666 | 70.653 | 0 | 0 | 3 | 0 |  | | | | | | 103.0219329 | 0.275507232 | 111.9068051 | 1.942883361 | 2 | 2 |  | 0 | 0 | 0 | 0 | 3 |  | | | | |
| 2540 | 0 | 18.681 | 1.9 | 10.170762 | 89.829239 | 1 | 17.590691 | 1 | 1.4721255 | 2 | 108.05199 | 25.566109 | 69.400002 | 0 | 19 | 3 | 0 | 0 | 0 | 0 | 0 | 0 | 0 |  | NA |  | NA | 2 | 2 | 2 | 0 | 0 | 0 |  | 3 | 24 | 0 | 7 | 0 |  |
| 2540 | 0 | 21.724001 | 2.4400001 | 11.231817 | 88.768181 | 1 | 17.63176 | 1 | 1.4721255 | 2 | 108.05199 | 25.435555 | 70.540001 | 0 | 20 | 3 | 0 | 0 | 0 | 0 | 0 | 0 | 0 | 95.0348057 | -0.453196223 | 104.5897919 | 0.698855174 | 2 | 2 | 2 | 0 | 0 | 0 | 0 | 3 | 25 | 0 |  | | |
| 2540 | 0 | 25.538 | 2.76 | 10.807425 | 89.192574 | 1 | 17.67009 | 1 | 1.4721255 | 2 | 108.05199 | 23.942223 | 70.290001 | 0 | 21 | 3 | 0 | 0 | 0 | 0 | 0 | 0 | 0 |  | NA |  | NA | 2 | 2 | 2 | 0 | 0 | 0 |  | 3 | 24 | 0 | 7 | 0 |  |
| 2541 |  | 24.374 | 3.241 | 13.296956 | 86.703041 | 1 | 12.090349 | 1 | -3.2767196 | 2 | 106.31058 | 22.421667 | 72.099 | 0 | 0 | 1 | 1 |  | | | | | | 101.6938892 | 0.155379556 | 111.9905587 | 2.14933506 | 2 | 2 |  | 0 | 0 | 0 | 0 | 3 |  | | | | |
| 2541 | 0 | 20.937 | 2.1500001 | 10.268903 | 89.731094 | 1 | 13.092402 | 1 | -3.2767196 | 2 | 106.31058 | 25.207779 | 69.620003 | 0 | 21 | 1 | 1 | 0 | 0 | 0 | 0 | 0 | 0 |  | NA |  | NA | 2 | 2 | 2 | 0 | 0 | 0 |  | 3 | 25 | 0 | 7 | 0 |  |
| 2544 |  | 25.845 | 3.159 | 12.222867 | 87.77713 | 1 | 12.673512 | 0 | 1.7815342 | 3 | 108.58445 | 23.85111 | 71.932 | 0 | 0 | 3 | 1 |  | | | | | | 121.5119056 | 1.863921002 | 95.31712906 | -0.770513851 | 2 | 2 |  | 0 | 0 | 1 | 0 | 3 |  | | | | |
| 2544 | 1 | 16.971001 | 1.74 | 10.252784 | 89.747215 | 1 | 13.574265 | 0 | 1.7815342 | 3 | 108.58445 | 25.184444 | 71.910004 | 0 | 19 | 3 | 1 | 1 | 1 | 1 | 1 | 1 | 1 |  | NA |  | NA | 2 | 2 | 1 | 0 | 0 | 1 |  | 3 | 24 | 0 | 6.8000002 | 1 |  |
| 2544 | 0 | 19.17 | 2.04 | 10.641627 | 89.358376 | 1 | 13.609857 | 0 | 1.7815342 | 3 | 108.58445 | 24.639442 | 75.489998 | 0 | 20 | 3 | 1 | 0 | 0 | 0 | 0 | 0 | 0 | 97.47882631 | -0.22103331 | 94.00751735 | -0.957228286 | 2 | 2 | 1 | 0 | 0 | 1 | 1 | 3 | 14 | 1 |  | | |
| 2544 | 1 | 20.851 | 2.23 | 10.694931 | 89.305069 | 1 | 13.648186 | 0 | 1.7815342 | 3 | 108.58445 | 24.527224 | 72.889999 | 0 | 21 | 3 | 1 | 1 | 1 | 1 | 0 | 1 | 1 |  | NA |  | NA | 2 | 2 | 2 | 0 | 0 | 1 |  | 3 | 24 | 0 | 7 | 0 |  |
| 2545 |  | 24.677 | 2.988 | 12.108441 | 87.891556 | 0 | 11.652293 | 1 | 2.1023452 | 4 | 111.60221 | 23.267778 | 72.765 | 0 | 0 | 2 | 0 |  | | | | | | 129.6439103 | 2.698946977 | 91.93766039 | -1.212291863 | 2 | 2 |  | 1 | 0 | 0 | 1 | 2 |  | | | | |
| 2545 | 0 | 16.889 | 1.75 | 10.361773 | 89.638229 | 1 | 12.522929 | 1 | 2.1023452 | 4 | 111.60221 | 25.637224 | 69.669998 | 0 | 19 | 2 | 0 | 0 | 0 | 0 | 0 | 0 | 0 |  | NA |  | NA | 2 | 2 | 2 | 1 | 0 | 0 |  | 2 | 24 | 0 | 7 | 0 |  |
| 2545 | 0 | 21.534 | 2.3399999 | 10.866536 | 89.133461 | 1 | 12.602327 | 1 | 2.1023452 | 4 | 111.60221 | 24.699444 | 70.720001 | 0 | 21 | 2 | 0 | 0 | 0 | 0 | 0 | 0 | 0 |  | NA |  | NA | 2 | 2 | 2 | 1 | 0 | 0 |  | 2 | 24 | 0 | 7 | 0 |  |
| 2548 |  | 25.298 | 3.37 | 13.321211 | 86.678787 | 0 | 9.0568104 | 0 | 1.4721255 | 3 | 131.72522 | 21.998888 | 71.655 | 0 | 0 | 3 | 1 |  | | | | | | 132.1242723 | 2.756575745 | 108.5755241 | 1.803335484 | 2 | 2 |  | 0 | 0 | 0 | 0 | 2 |  | | | | |
| 2548 | 0 | 16.607 | 1.74 | 10.477509 | 89.522491 | 0 | 9.9739904 | 0 | 1.4721255 | 3 | 131.72522 | 26.534445 | 65.519997 | 0 | 19 | 3 | 1 | 0 | 0 | 0 | 0 | 0 |  | | NA |  | NA | 2 | 2 | 2 | 0 | 0 | 0 |  | 2 | 27 | 0 | 7 | 0 |  |
| 2548 | 0 | 19.674999 | 2.1199999 | 10.775095 | 89.224907 | 0 | 10.009583 | 0 | 1.4721255 | 3 | 131.72522 | 26.135 | 68.089996 | 0 | 20 | 3 | 1 | 0 | 0 | 0 | 0 | 0 |  | 119.4473609 | 1.703705535 | 101.2363695 | 0.227069189 | 2 | 2 | 2 | 0 | 0 | 0 | 0 | 2 | 27 | 0 |  | | |
| 2548 | 1 | 20.785 | 2.3 | 11.065672 | 88.934326 | 0 | 10.047913 | 0 | 1.4721255 | 3 | 131.72522 | 25.86611 | 66.849998 | 0 | 21 | 3 | 1 | 1 | 0 | 0 | 0 | 0 |  | | NA |  | NA | 2 | 2 | 2 | 0 | 0 | 0 |  | 2 | 26 | 0 | 7 | 0 |  |
| 2549 |  | 25.548 | 3.504 | 13.71536 | 86.284637 | 1 | 17.59343 | 0 | 1.7179354 | 4 | 83.877289 | 23.325001 | 72.013 | 0 | 0 | 2 | 0 |  | | | | | | 94.04802569 | -0.512854464 | 95.57722343 | -0.695919249 | 2 | 2 |  | 0 | 0 | 0 | 0 | 3 |  | | | | |
| 2549 | 0 | 17.264 | 1.75 | 10.136701 | 89.863297 | 1 | 18.505133 | 0 | 1.7179354 | 4 | 83.877289 | 25.245556 | 70.760002 | 0 | 19 | 2 | 0 | 0 | 0 | 0 | 0 | 0 | 0 |  | NA |  | NA | 2 | 2 | 2 | 0 | 0 | 0 |  | 3 | 24 | 0 | 7 | 0 |  |
| 2549 | 0 | 20.093 | 2.1300001 | 10.600707 | 89.399292 | 1 | 18.546202 | 0 | 1.7179354 | 4 | 83.877289 | 24.427778 | 74.190002 | 0 | 20 | 2 | 0 | 0 | 0 | 0 | 0 | 0 | 0 | 102.3240055 | 0.197888426 | 95.53837626 | -0.693249957 | 2 | 2 | 2 | 0 | 0 | 0 | 0 | 3 | 24 | 0 |  | | |
| 2551 |  | 24.789 | 3.416 | 13.780306 | 86.219696 | 1 | 12.930869 | 1 | 0.080996305 | 3 | 98.722015 | 22.975555 | 70.274 | 0 | 0 | 3 | 1 |  | | | | | | 110.166211 | 0.930563526 | 96.03676187 | -0.609660224 | 2 | 2 |  | 0 | 0 | 0 | 0 | 3 |  | | | | |
| 2551 | 0 | 18.690001 | 1.77 | 9.4703045 | 90.529694 | 1 | 13.848049 | 1 | 0.080996305 | 3 | 98.722015 | 25.906109 | 67.339996 | 0 | 19 | 3 | 1 | 0 | 0 | 0 | 0 | 0 | 0 |  | NA |  | NA | 2 | 2 | 2 | 0 | 0 | 0 |  | 3 | 24 | 0 | 7 | 0 |  |
| 2551 | 0 | 20.273001 | 2.28 | 11.246485 | 88.753517 | 1 | 13.886379 | 1 | 0.080996305 | 3 | 98.722015 | 25.818333 | 69.43 | 0 | 20 | 3 | 1 |  | | 0 | 0 | 0 |  | 106.0694307 | 0.555046183 | 94.83868296 | -0.767859522 | 2 | 2 | 2 | 0 | 0 | 0 | 0 | 3 | 24 | 0 |  | | |
| 2551 | 1 | 23.952999 | 2.8399999 | 11.856552 | 88.143448 | 1 | 13.908282 | 1 | 0.080996305 | 3 | 98.722015 | 24.881113 | 67.669998 | 0 | 21 | 3 | 1 | 1 | 0 | 0 | 0 | 0 | 0 |  | NA |  | NA | 2 | 2 | 2 | 0 | 0 | 0 |  | 3 | 25 | 0 | 7 | 0 |  |
| 2552 |  | 26.504 | 3.412 | 12.873528 | 87.126472 | 1 | 14.748802 | 0 | -0.79377419 | 3 | 105.2164 | 22.802221 | 71.975 | 0 | 0 | 3 |  | | | | | | | 117.0390769 | 1.460878796 | 96.30007499 | -0.605678636 | 2 | 2 |  | 1 | 0 | 0 | 0 | 4 |  | | | | |
| 2552 | 0 | 17.716 | 1.73 | 9.7651844 | 90.234818 | 1 | 15.646817 | 0 | -0.79377419 | 3 | 105.2164 | 25.668335 | 70.080002 | 0 | 19 | 3 |  | 0 | 0 | 0 | 0 | 0 | 0 |  | NA |  | NA | 2 | 2 | 2 | 1 | 0 | 0 |  | 4 | 24 | 0 | 6.9000001 | 1 |  |
| 2552 | 1 | 19.386999 | 2.02 | 10.419353 | 89.580643 | 1 | 15.687885 | 0 | -0.79377419 | 3 | 105.2164 | 25.244446 | 72.779999 | 0 | 20 | 3 |  | 1 | 0 | 0 | 0 | 1 | 0 | 101.5144544 | 0.13042491 | 103.1643944 | 0.559402346 | 2 | 2 | 2 | 1 | 0 | 0 | 0 | 4 | 24 | 0 |  | | |
| 2552 | 0 | 21.361 | 2.29 | 10.720472 | 89.279526 | 1 | 15.726215 | 0 | -0.79377419 | 3 | 105.2164 | 24.85722 | 70.790001 | 0 | 21 | 3 |  | 0 | 0 | 0 | 0 | 0 | 0 |  | NA |  | NA | 2 | 2 | 2 | 1 | 0 | 0 |  | 4 | 24 | 0 | 7 | 0 |  |
| 2554 |  | 27.584 | 3.259 | 11.81482 | 88.185181 | 0 | 10.872005 | 1 | 2.3747432 | 2 | 98.971069 | 23.454445 | 71.757 | 0 | 0 | 3 | 1 |  | | | | | | 107.0462071 | 0.642307808 | 100.3693253 | 0.060261504 | 2 | 2 |  | 1 | 0 | 1 | 0 | 3 |  | | | | |
| 2554 | 0 | 19.155001 | 1.9400001 | 10.127904 | 89.872093 | 0 | 11.767282 | 1 | 2.3747432 | 2 | 98.971069 | 25.844442 | 68.139999 | 0 | 19 | 3 | 1 | 0 | 0 | 0 | 0 | 0 |  | | NA |  | NA | 2 | 2 | 2 | 1 | 0 | 1 |  | 3 | 27 | 0 | 7 | 0 |  |
| 2554 | 1 | 22.540001 | 2.3099999 | 10.248446 | 89.751556 | 0 | 11.808351 | 1 | 2.3747432 | 2 | 98.971069 | 25.537224 | 71.040001 | 0 | 20 | 3 | 1 | 1 | 1 | 1 | 1 | 0 |  | 110.6739102 | 0.976580165 | 102.9620491 | 0.491973803 | 2 | 2 | 2 | 1 | 0 | 1 | 0 | 3 | 25 | 0 |  | | |
| 2554 | 1 | 23.549 | 2.5799999 | 10.955879 | 89.044121 | 0 | 11.849419 | 1 | 2.3747432 | 2 | 98.971069 | 24.788891 | 70.040001 | 0 | 21 | 3 | 1 | 1 | 1 | 1 | 1 | 1 |  | | NA |  | NA | 2 | 2 | 2 | 1 | 0 | 1 |  | 3 | 18 | 1 | 6 | 1 |  |
| 2556 |  | 26.083 | 3.046 | 11.678104 | 88.321892 | 1 | 16.188911 | 1 | 0.048028298 | 2 | 112.4444 | 23.948334 | 72.587 | 0 | 0 | 3 | 1 |  | | | | | | 111.6191041 | 1.057562748 | 107.6374356 | 1.214152911 | 2 | 2 |  | 0 | 0 | 1 | 0 | 3 |  | | | | |
| 2556 | 0 | 20.764999 | 2.1199999 | 10.209487 | 89.790512 | 1 | 17.034908 | 1 | 0.048028298 | 2 | 112.4444 | 25.958887 | 68.43 | 0 | 19 | 3 | 1 | 0 | 0 | 0 | 0 | 0 | 0 |  | NA |  | NA | 2 | 2 | 2 | 0 | 0 | 1 |  | 3 | 24 | 0 | 7 | 0 |  |
| 2556 | 0 | 22.587 | 2.45 | 10.846948 | 89.153053 | 1 | 17.092403 | 1 | 0.048028298 | 2 | 112.4444 | 25.769445 | 70.779999 | 0 | 20 | 3 | 1 | 0 | 0 | 0 | 0 | 0 | 0 | 101.4279651 | 0.130175752 | 109.9652593 | 1.591043207 | 2 | 2 | 2 | 0 | 0 | 1 | 0 | 3 | 24 | 0 |  | | |
| 2556 | 0 | 25.584 | 2.78 | 10.866166 | 89.133835 | 1 | 17.130732 | 1 | 0.048028298 | 2 | 112.4444 | 24.602221 | 69.809998 | 0 | 21 | 3 | 1 | 0 | 0 | 0 | 0 | 0 | 0 |  | NA |  | NA | 2 | 2 | 2 | 0 | 0 | 1 |  | 3 | 24 | 0 | 7 | 0 |  |
| 2558 |  | 22.254 | 2.695 | 12.110183 | 87.889816 | 0 | 11.170431 | 1 | -1.7033793 | 2 | 90.3088 | 23.314444 | 74.165 | 0 | 0 | 3 | 1 |  | | | | | | 92.91180421 | -0.649784998 | 104.0129283 | 0.676999087 | 1 | 2 |  | 0 |  | 1 | 0 | 3 |  | | | | |
| 2558 | 0 | 18.660999 | 1.86 | 9.9673119 | 90.032684 | 1 | 12.021903 | 1 | -1.7033793 | 2 | 90.3088 | 25.629446 | 69.419998 | 0 | 19 | 3 | 1 | 0 | 0 | 0 | 0 | 0 | 0 |  | NA |  | NA | 1 | 2 | 2 | 0 |  | 1 |  | 3 | 24 | 0 | 7 | 0 |  |
| 2558 | 0 | 20.945999 | 2.22 | 10.598682 | 89.401314 | 1 | 12.060233 | 1 | -1.7033793 | 2 | 90.3088 | 25.236113 | 72.339996 | 0 | 20 | 3 | 1 | 0 | 0 | 0 | 0 | 0 | 0 | 91.88615474 | -0.746287395 | 80.00577832 | -2.662853363 | 1 | 2 | 2 | 0 |  | 1 | 1 | 3 | 16 | 1 |  | | |
| 2558 | 1 | 24.377001 | 2.6199999 | 10.747835 | 89.252167 | 1 | 12.098562 | 1 | -1.7033793 | 2 | 90.3088 | 24.736113 | 70.559998 | 0 | 21 | 3 | 1 | 1 | 1 | 1 | 1 | 1 | 1 |  | NA |  | NA | 1 | 2 | 2 | 0 |  | 1 |  | 3 | 24 | 0 | 7 | 0 |  |
| 2559 |  | 19.971 | 2.209 | 11.061039 | 88.938965 | 1 | 14.464066 | 0 | -1.2399462 | 2 | 105.49629 | 21.131111 | 77.16 | 0 | 0 | 2 | 1 |  | | | | | | 105.3233928 | 0.461629402 | 107.4527096 | 1.432174993 | 2 | 2 |  | 0 | 0 | 0 | 0 | 3 |  | | | | |
| 2559 | 0 | 21.233999 | 2.1800001 | 10.266555 | 89.733444 | 1 | 15.244353 | 0 | -1.2399462 | 2 | 105.49629 | 25.633888 | 70.970001 | 0 | 20 | 2 | 1 | 0 | 0 | 0 | 0 | 0 | 0 | 104.4761869 | 0.386577294 | 106.826588 | 1.285663787 | 2 | 2 | 2 | 0 | 0 | 0 | 0 | 3 | 24 | 0 |  | | |
| 2559 | 0 | 29.291 | 2.7 | 9.2178488 | 90.78215 | 1 | 15.301848 | 0 | -1.2399462 | 2 | 105.49629 | 24.462221 | 70.169998 | 0 | 21 | 2 | 1 | 0 | 0 | 0 | 0 | 0 | 0 |  | NA |  | NA | 2 | 2 | 2 | 0 | 0 | 0 |  | 3 | 25 | 0 | 7 | 0 |  |
| 2561 |  | 21.974 | 2.916 | 13.270228 | 86.729774 | 0 | 11.318275 | 1 | -0.40354156 | 2 | 107.31879 | 23.021667 | 74.623 | 0 | 0 | 3 | 1 |  | | | | | | 118.348658 | 1.672660706 | 97.0349095 | -0.468433219 | 2 | 2 |  | 0 | 0 | 1 | 0 | 4 |  | | | | |
| 2561 | 0 | 18.900999 | 2.01 | 10.634358 | 89.365639 | 1 | 12.156057 | 1 | -0.40354156 | 2 | 107.31879 | 25.388332 | 69.410004 | 0 | 19 | 3 | 1 | 0 | 0 | 0 | 0 | 0 | 0 |  | NA |  | NA | 2 | 2 | 2 | 0 | 0 | 1 |  | 4 | 24 | 0 | 7 | 0 |  |
| 2561 | 0 | 22.302999 | 2.5999999 | 11.657624 | 88.342377 | 1 | 12.191649 | 1 | -0.40354156 | 2 | 107.31879 | 24.404446 | 74 | 0 | 20 | 3 | 1 | 0 | 0 | 0 | 0 | 0 | 0 | 105.2742125 | 0.483463695 | 88.54521765 | -1.66117101 | 2 | 2 | 2 | 0 | 0 | 1 | 1 | 4 | 21 | 0 |  | | |
| 2561 | 0 | 24.229 | 2.9100001 | 12.010401 | 87.989601 | 1 | 12.22998 | 1 | -0.40354156 | 2 | 107.31879 | 24.665001 | 71.339996 | 0 | 21 | 3 | 1 | 0 | 0 | 0 | 0 | 0 | 0 |  | NA |  | NA | 2 | 2 | 2 | 0 | 0 | 1 |  | 4 | 24 | 0 | 7 | 0 |  |
| 2562 |  | 19.162 | 1.678 | 8.7569151 | 91.243088 | 1 | 12.843258 | 1 | -1.5018775 | 4 | 111.31256 | 18.776112 | 79.293 | 0 | 0 | 2 |  | | | | | | | 120.6218349 | 1.883105234 | 98.33971699 | -0.261005566 |  | | | | | 0 | 0 | 4 |  | | | | |
| 2562 | 0 | 16.92 | 1.76 | 10.401892 | 89.598106 | 1 | 13.453798 | 1 | -1.5018775 | 4 | 111.31256 | 24.913334 | 73.360001 | 0 | 19 | 2 |  | 0 | 0 | 0 | 0 | 0 | 0 |  | NA |  | NA |  | | 2 |  | | 0 |  | 4 | 24 | 0 | 7 | 0 |  |
| 2562 | 0 | 21.516001 | 2.3199999 | 10.782672 | 89.217331 | 1 | 13.524982 | 1 | -1.5018775 | 4 | 111.31256 | 23.733334 | 73.18 | 0 | 21 | 2 |  | 0 | 0 | 0 | 0 | 0 | 0 |  | NA |  | NA |  | | 2 |  | | 0 |  | 4 | 24 | 0 | 7 | 0 |  |
| 2564 |  | 21.518 | 2.392 | 11.116275 | 88.883728 | 0 | 10.477755 | 1 | 1.7179354 | 3 | 110.23439 | 21.532223 | 76.443 | 0 | 0 | 3 | 1 |  | | | | | | 123.6656768 | 2.141417043 | 95.54689682 | -0.692600475 | 2 | 2 |  | 0 | 0 | 1 | 0 | 3 |  | | | | |
| 2564 | 0 | 17.212 | 1.71 | 9.9349289 | 90.065071 | 0 | 11.238877 | 1 | 1.7179354 | 3 | 110.23439 | 25.453335 | 68.32 | 0 | 19 | 3 | 1 | 0 | 0 | 0 | 0 | 0 |  | | NA |  | NA | 2 | 2 | 2 | 0 | 0 | 1 |  | 3 | 26 | 0 | 7 | 0 |  |
| 2564 | 1 | 20.306 | 2.0999999 | 10.34177 | 89.658226 | 0 | 11.282683 | 1 | 1.7179354 | 3 | 110.23439 | 24.71722 | 72.57 | 0 | 20 | 3 | 1 | 1 | 0 | 0 | 0 | 0 |  | 122.7976924 | 2.079475179 | 92.2467722 | -1.168478635 | 2 | 2 | 2 | 0 | 0 | 1 | 0 | 3 | 26 | 0 |  | | |
| 2564 | 0 | 24.568001 | 2.6500001 | 10.786388 | 89.213608 | 0 | 11.321013 | 1 | 1.7179354 | 3 | 110.23439 | 23.811666 | 71.129997 | 0 | 21 | 3 | 1 | 0 | 0 | 0 | 0 | 0 |  | | NA |  | NA | 2 | 2 | 2 | 0 | 0 | 1 |  | 3 | 27 | 0 | 7 | 0 |  |
| 2566 |  | 21.416 | 2.362 | 11.029137 | 88.970863 | 1 | 14.970568 | 1 | -3.8274689 | 2 | 117.23508 | 21.438889 | 76.633 | 0 | 0 | 3 |  | | | | | | | 120.8350935 | 1.895941187 | 103.8337057 | 0.604943018 |  | | | 0 | 0 | 0 | 0 | 3 |  | | | | |
| 2566 | 0 | 17.157 | 1.6900001 | 9.8502073 | 90.149796 | 1 | 15.720739 | 1 | -3.8274689 | 2 | 117.23508 | 25.436113 | 68.389999 | 0 | 19 | 3 |  | 0 | 0 | 0 | 0 | 0 | 0 |  | NA |  | NA |  | | 2 | 0 | 0 | 0 |  | 3 | 24 | 0 | 7 | 0 |  |
| 2566 | 0 | 19.451 | 2.0599999 | 10.590714 | 89.409286 | 1 | 15.756331 | 1 | -3.8274689 | 2 | 117.23508 | 24.412779 | 72.980003 | 0 | 20 | 3 |  | 0 | 0 | 0 | 0 | 0 | 0 | 123.3203708 | 2.11792531 | 97.86079652 | -0.316583702 |  | | 2 | 0 | 0 | 0 | 0 | 3 | 24 | 0 |  | | |
| 2566 | 0 | 23.525 | 2.53 | 10.754517 | 89.245483 | 1 | 15.794662 | 1 | -3.8274689 | 2 | 117.23508 | 24.514444 | 71.029999 | 0 | 21 | 3 |  | 0 | 0 | 0 | 0 | 0 | 0 |  | NA |  | NA |  | | 2 | 0 | 0 | 0 |  | 3 | 24 | 0 | 7 | 0 |  |
| 2568 |  | 18.688 | 1.767 | 9.455265 | 90.544731 | 0 | 11.282683 | 1 | 0.88414335 | 2 | 87.028336 | 17.691668 | 82.077 | 0 | 0 | 3 | 1 |  | | | | | | 94.58672981 | -0.496325459 | 98.68484392 | -0.211047146 | 2 | 2 |  | 0 | 0 | 1 | 0 | 4 |  | | | | |
| 2568 | 0 | 18.834 | 2.01 | 10.672189 | 89.327812 | 0 | 11.849419 | 1 | 0.88414335 | 2 | 87.028336 | 24.962778 | 72.589996 | 0 | 19 | 3 | 1 | 0 | 0 | 0 | 0 | 0 |  | | NA |  | NA | 2 | 2 | 2 | 0 | 0 | 1 |  | 4 | 26 | 0 | 7 | 0 |  |
| 2568 | 1 | 21.129999 | 2.4200001 | 11.452911 | 88.547089 | 0 | 11.868583 | 1 | 0.88414335 | 2 | 87.028336 | 25.073891 | 74.730003 | 0 | 20 | 3 | 1 | 1 | 1 | 1 | 1 | 1 |  | 115.8645983 | 1.450391249 | 94.38220817 | -0.86038436 | 2 | 2 | 2 | 0 | 0 | 1 | 0 | 4 | 27 | 0 |  | | |
| 2568 | 1 | 24.264999 | 2.6700001 | 11.003504 | 88.996498 | 0 | 11.926078 | 1 | 0.88414335 | 2 | 87.028336 | 24.022776 | 73.209999 | 0 | 21 | 3 | 1 | 1 | 0 | 0 | 0 | 0 |  | | NA |  | NA | 2 | 2 | 2 | 0 | 0 | 1 |  | 4 | 27 | 0 | 7 | 0 |  |
| 2569 |  | 22.013 | 2.436 | 11.066188 | 88.933815 | 0 | 9.6755648 | 1 | -0.75279576 | 4 | 118.7271 | 21.415556 | 77.881 | 0 | 0 | 3 | 1 |  | | | | | | 126.0050451 | 2.326031918 | 101.7213678 | 0.282982039 | 2 | 2 |  | 0 | 0 | 1 | 0 | 3 |  | | | | |
| 2569 | 0 | 18.275999 | 1.9 | 10.396149 | 89.603851 | 0 | 10.436687 | 1 | -0.75279576 | 4 | 118.7271 | 25.240555 | 71.190002 | 0 | 19 | 3 | 1 | 0 | 0 | 0 | 0 | 0 |  | | NA |  | NA | 2 | 2 | 2 | 0 | 0 | 1 |  | 3 | 27 | 0 | 7 | 0 |  |
| 2569 | 1 | 21.035 | 2.3299999 | 11.076777 | 88.923225 | 0 | 10.475018 | 1 | -0.75279576 | 4 | 118.7271 | 24.727224 | 74.519997 | 0 | 20 | 3 | 1 | 1 | 1 | 1 | 1 | 0 |  | 119.9923504 | 1.815833472 | 97.69344524 | -0.366824656 | 2 | 2 | 2 | 0 | 0 | 1 | 0 | 3 | 26 | 0 |  | | |
| 2569 | 1 | 23.212 | 2.5999999 | 11.201102 | 88.798897 | 0 | 10.513347 | 1 | -0.75279576 | 4 | 118.7271 | 24.346664 | 72.449997 | 0 | 21 | 3 | 1 | 1 | 0 | 1 | 0 | 0 |  | | NA |  | NA | 2 | 2 | 2 | 0 | 0 | 1 |  | 3 | 23 | 0 | 6.8000002 | 1 |  |
| 2570 |  | 24.774 | 2.416 | 9.7521591 | 90.247841 | 0 | 10.828199 | 0 | -2.9581742 | 2 | 94.131035 | 20.916666 | 75.062 | 0 | 0 | 3 | 1 |  | | | | | | 113.0206997 | 1.146372888 | 89.14442878 | -1.639846117 | 2 | 2 |  | 0 | 0 | 1 | 0 | 3 |  | | | | |
| 2570 | 0 | 21.648001 | 2.28 | 10.53215 | 89.46785 | 0 | 11.550992 | 0 | -2.9581742 | 2 | 94.131035 | 25.761667 | 69.449997 | 0 | 19 | 3 | 1 | 0 | 0 | 0 | 0 | 0 |  | | NA |  | NA | 2 | 2 | 2 | 0 | 0 | 1 |  | 3 | 26 | 0 | 7 | 0 |  |
| 2570 | 0 | 22.566 | 2.3 | 10.192325 | 89.807678 | 0 | 11.56742 | 0 | -2.9581742 | 2 | 94.131035 | 26.293335 | 71.190002 | 0 | 20 | 3 | 1 | 0 | 0 | 0 | 0 | 0 |  | 98.23556839 | -0.156233641 | 90.44234949 | -1.464101389 | 2 | 2 | 2 | 0 | 0 | 1 | 1 | 3 | 26 | 0 |  | | |
| 2570 | 0 | 24.479 | 2.6600001 | 10.866457 | 89.133545 | 0 | 11.619439 | 0 | -2.9581742 | 2 | 94.131035 | 25.45389 | 69.120003 | 0 | 21 | 3 | 1 | 0 | 0 | 0 | 0 | 0 |  | | NA |  | NA | 2 | 2 | 2 | 0 | 0 | 1 |  | 3 | 27 | 0 | 7 | 0 |  |
| 2572 |  | 21.306 | 2.218 | 10.410213 | 89.58979 | 1 | 14.258727 | 1 | -1.1277679 | 2 | 127.89391 | 21.932777 | 73.236 | 0 | 0 | 2 | 1 |  | | | | | | 136.2977865 | 3.296218932 | 100.0669461 | 0.010394473 | 2 | 2 |  | 0 | 0 | 1 | 0 | 3 |  | | | | |
| 2572 | 0 | 16.405001 | 1.71 | 10.423651 | 89.576347 | 1 | 14.986995 | 1 | -1.1277679 | 2 | 127.89391 | 26.644999 | 65.07 | 0 | 19 | 2 | 1 | 0 | 0 | 0 | 0 | 0 | 0 |  | NA |  | NA | 2 | 2 | 2 | 0 | 0 | 1 |  | 3 | 24 | 0 | 7 | 0 |  |
| 2572 | 0 | 19.59 | 2.1199999 | 10.821847 | 89.178154 | 1 | 15.030801 | 1 | -1.1277679 | 2 | 127.89391 | 26.296112 | 67.540001 | 0 | 20 | 2 | 1 | 0 | 0 | 0 | 0 | 0 | 0 | 104.9176822 | 0.4488402 | 95.32066551 | -0.682928634 | 2 | 2 | 2 | 0 | 0 | 1 | 0 | 3 | 25 | 0 |  | | |
| 2573 |  | 22.363 | 2.168 | 9.6945848 | 90.305412 | 1 | 14.050651 | 1 | -0.35855055 | 3 | 122.54913 | 21.176111 | 77.773 | 0 | 0 | 3 | 1 |  | | | | | | 129.2621893 | 2.662343078 | 101.4002256 | 0.220765695 | 2 | 2 |  | 1 | 0 | 0 | 0 | 3 |  | | | | |
| 2573 | 0 | 16.441999 | 1.6900001 | 10.278556 | 89.721443 | 1 | 14.792608 | 1 | -0.35855055 | 3 | 122.54913 | 24.983334 | 72.790001 | 0 | 19 | 3 | 1 | 0 | 0 | 0 | 0 | 0 | 0 |  | NA |  | NA | 2 | 2 | 2 | 1 | 0 | 0 |  | 3 | 24 | 0 | 7 | 0 |  |
| 2573 | 1 | 18.834 | 2 | 10.619093 | 89.380905 | 1 | 14.833675 | 1 | -0.35855055 | 3 | 122.54913 | 24.612778 | 76.019997 | 0 | 20 | 3 | 1 | 1 | 1 | 1 | 1 | 0 | 1 | 134.0262518 | 3.087680874 | 99.85712826 | -0.021858467 | 2 | 2 | 2 | 1 | 0 | 0 | 0 | 3 | 25 | 0 |  | | |
| 2573 | 0 | 20.871 | 2.23 | 10.684682 | 89.315315 | 1 | 14.869267 | 1 | -0.35855055 | 3 | 122.54913 | 23.995001 | 73.610001 | 0 | 21 | 3 | 1 | 0 | 0 | 0 | 0 | 0 | 0 |  | NA |  | NA | 2 | 2 | 2 | 1 | 0 | 0 |  | 3 | 24 | 0 | 7 | 0 |  |
| 2574 |  | 22.342 | 2.173 | 9.7260761 | 90.273926 | 0 | 9.0787134 | 1 | -3.5694447 | 2 | 83.043816 | 21.178888 | 77.911 | 0 | 0 | 3 | 1 |  | | | | | | 93.64618292 | -0.566233877 | 94.63205391 | -0.814038869 | 2 | 2 |  | 0 | 0 | 1 | 1 | 3 |  | | | | |
| 2574 | 1 | 16.660999 | 1.75 | 10.503572 | 89.496429 | 0 | 9.8425732 | 1 | -3.5694447 | 2 | 83.043816 | 24.787775 | 73.989998 | 0 | 19 | 3 | 1 | 1 | 1 | 1 | 1 | 1 |  | | NA |  | NA | 2 | 2 | 1 | 0 | 0 | 1 |  | 3 | 16 | 1 | 5.4000001 | 1 |  |
| 2574 | 1 | 19.736 | 2.0999999 | 10.640453 | 89.359543 | 0 | 9.8781652 | 1 | -3.5694447 | 2 | 83.043816 | 24.711668 | 75.209999 | 0 | 20 | 3 | 1 | 1 | 1 | 1 | 1 | 1 |  | 100.7147945 | 0.064737391 | 92.10571547 | -1.182145299 | 2 | 2 | 1 | 0 | 0 | 1 | 1 | 3 | 27 | 0 |  | | |
| 2574 | 1 | 21.125 | 2.27 | 10.745563 | 89.25444 | 0 | 9.9137573 | 1 | -3.5694447 | 2 | 83.043816 | 23.574999 | 73.790001 | 0 | 21 | 3 | 1 | 1 | 1 | 0 | 1 | 0 |  | | NA |  | NA | 2 | 2 | 2 | 0 | 0 | 1 |  | 3 | 27 | 0 | 6.5 | 1 |  |
| 2575 |  | 19.715 | 2.106 | 10.682221 | 89.31778 | 0 | 11.422314 | 1 | 1.5965829 | 4 | 128.64214 | 19.661667 | 82.822 | 0 | 0 | 2 | 0 |  | | | | | | 136.5396265 | 3.319201686 | 101.2761588 | 0.209621093 | 2 | 2 |  | 0 | 0 | 0 | 0 | 2 |  | | | | |
| 2575 | 1 | 16.938 | 1.76 | 10.390837 | 89.609161 | 1 | 12.188911 | 1 | 1.5965829 | 4 | 128.64214 | 23.545557 | 77.419998 | 0 | 19 | 2 | 0 | 1 | 1 | 1 | 1 | 1 | 1 |  | NA |  | NA | 2 | 2 | 2 | 0 | 0 | 0 |  | 2 | 24 | 0 | 7 | 0 |  |
| 2575 | 0 | 25.468 | 2.8399999 | 11.151248 | 88.848755 | 1 | 12.265572 | 1 | 1.5965829 | 4 | 128.64214 | 22.296112 | 77.099998 | 0 | 21 | 2 | 0 | 0 | 0 | 0 | 0 | 0 | 0 |  | NA |  | NA | 2 | 2 | 2 | 0 | 0 | 0 |  | 2 | 20 | 0 | 7 | 0 |  |
| 2576 |  | 21.547 | 2.393 | 11.105954 | 88.894043 | 1 | 19.06913 | 1 | -0.68017346 | 4 | 115.78797 | 21.508333 | 77.355 | 0 | 0 | 3 | 1 |  | | | | | | 128.7848319 | 2.603337182 | 94.38017085 | -0.781541218 | 2 | 2 |  | 1 | 0 | 0 | 0 | 3 |  | | | | |
| 2576 | 0 | 19.028999 | 2 | 10.510274 | 89.489723 | 1 | 19.857632 | 1 | -0.68017346 | 4 | 115.78797 | 25.267223 | 71.620003 | 0 | 19 | 3 | 1 | 0 | 0 | 0 | 0 | 0 | 0 |  | NA |  | NA | 2 | 2 | 2 | 1 | 0 | 0 |  | 3 | 24 | 0 | 7 | 0 |  |
| 2576 | 1 | 22.295 | 2.47 | 11.078717 | 88.92128 | 1 | 19.895962 | 1 | -0.68017346 | 4 | 115.78797 | 24.971664 | 71.830002 | 0 | 20 | 3 | 1 | 1 | 0 | 1 | 1 | 1 | 0 | 122.1750351 | 2.005600116 | 93.57094818 | -0.884212884 | 2 | 2 | 2 | 1 | 0 | 0 | 0 | 3 | 24 | 0 |  | | |
| 2576 | 0 | 28.007999 | 2.8399999 | 10.13996 | 89.860039 | 1 | 19.920603 | 1 | -0.68017346 | 4 | 115.78797 | 23.546669 | 72.190002 | 0 | 21 | 3 | 1 | 0 | 0 | 0 | 0 | 0 | 0 |  | NA |  | NA | 2 | 2 | 2 | 1 | 0 | 0 |  | 3 | 25 | 0 | 7 | 0 |  |
| 2577 |  | 19.782 | 2.247 | 11.358811 | 88.64119 | 1 | 19.392197 | 0 | -1.3094851 | 2 | 112.63046 | 21.313889 | 77.515 | 0 | 0 | 3 | 1 |  | | | | | | 118.9072293 | 1.584320354 | 101.2785446 | 0.21181491 |  | | | 1 | 0 | 0 | 0 | 4 |  | | | | |
| 2577 | 0 | 16.202 | 1.73 | 10.677694 | 89.322304 | 1 | 20.145105 | 0 | -1.3094851 | 2 | 112.63046 | 25.687222 | 70.010002 | 0 | 19 | 3 | 1 | 0 | 0 | 0 | 0 | 0 | 0 |  | NA |  | NA |  | | 2 | 1 | 0 | 0 |  | 4 | 24 | 0 | 7 | 0 |  |
| 2577 | 1 | 19.539 | 2.1900001 | 11.208353 | 88.791649 | 1 | 20.183435 | 0 | -1.3094851 | 2 | 112.63046 | 26.043333 | 70.769997 | 0 | 20 | 3 | 1 | 1 | 1 | 1 | 1 | 1 | 1 | 121.4618773 | 1.790464968 | 98.70971787 | -0.205159122 |  | | 2 | 1 | 0 | 0 | 0 | 4 | 22 | 0 |  | | |
| 2577 | 0 | 19.129999 | 2.1500001 | 11.238893 | 88.761108 | 1 | 20.219028 | 0 | -1.3094851 | 2 | 112.63046 | 24.794443 | 71.139999 | 0 | 21 | 3 | 1 | 0 | 0 | 0 | 0 | 0 | 0 |  | NA |  | NA |  | | 2 | 1 | 0 | 0 |  | 4 | 25 | 0 | 7 | 0 |  |
| 2578 |  | 20.529 | 2.37 | 11.544644 | 88.455353 | 1 | 19.219713 | 0 | 0.72728837 | 2 | 92.326218 | 21.257778 | 77.709 | 0 | 0 | 3 | 1 |  | | | | | | 99.32214972 | -0.057765305 | 99.31085114 | -0.111644917 | 2 | 2 |  | 1 | 1 | 1 | 0 | 3 |  | | | | |
| 2578 | 1 | 18.01 | 2 | 11.104941 | 88.895058 | 1 | 19.997263 | 0 | 0.72728837 | 2 | 92.326218 | 25.435555 | 70.959999 | 0 | 19 | 3 | 1 | 1 | 0 | 0 | 0 | 1 | 0 |  | NA |  | NA | 2 | 2 | 2 | 1 | 1 | 1 |  | 3 | 24 | 0 | 7 | 0 |  |
| 2578 | 0 | 21.09 | 2.4300001 | 11.522049 | 88.477951 | 1 | 20.035591 | 0 | 0.72728837 | 2 | 92.326218 | 25.028891 | 71.949997 | 0 | 20 | 3 | 1 | 0 | 0 | 0 | 0 | 0 | 0 | 86.6872107 | -1.14375121 | 107.5167186 | 1.325240356 | 2 | 2 | 2 | 1 | 1 | 1 | 0 | 3 | 25 | 0 |  | | |
| 2578 | 1 | 24.476999 | 2.8299999 | 11.561874 | 88.438126 | 1 | 20.073921 | 0 | 0.72728837 | 2 | 92.326218 | 23.691109 | 72.029999 | 0 | 21 | 3 | 1 | 0 | 0 | 0 | 0 | 1 | 0 |  | NA |  | NA | 2 | 2 | 2 | 1 | 1 | 1 |  | 3 | 24 | 0 | 7 | 0 |  |
| 2582 |  | 19.769 | 2.227 | 11.265112 | 88.734886 | 1 | 18.250513 | 1 | -2.2758231 | 2 | 119.45335 | 21.774445 | 76.361 | 0 | 0 | 3 |  | | | | | | | 128.4193009 | 2.571848009 | 99.46681947 | -0.077580148 | 2 | 2 |  | 0 | 0 | 1 | 0 | 3 |  | | | | |
| 2582 | 0 | 19.132 | 1.84 | 9.6173954 | 90.382607 | 1 | 18.973307 | 1 | -2.2758231 | 2 | 119.45335 | 25.896111 | 68.519997 | 0 | 19 | 3 |  | 0 | 0 | 0 | 0 | 0 | 0 |  | NA |  | NA | 2 | 2 | 2 | 0 | 0 | 1 |  | 3 | 24 | 0 | 7 | 0 |  |
| 2582 | 0 | 20.587 | 2.0599999 | 10.006314 | 89.993683 | 1 | 19.011637 | 1 | -2.2758231 | 2 | 119.45335 | 25.447222 | 70.800003 | 0 | 20 | 3 |  | 0 | 0 | 0 | 0 | 0 | 0 |  | NA |  | NA | 2 | 2 | 2 | 0 | 0 | 1 |  | 3 | 24 | 0 |  | | |
| 2582 | 0 | 24.149 | 2.47 | 10.228167 | 89.771835 | 1 | 19.049965 | 1 | -2.2758231 | 2 | 119.45335 | 25.391111 | 69.589996 | 0 | 21 | 3 |  | 0 | 0 | 0 | 0 | 0 | 0 |  | NA |  | NA | 2 | 2 | 2 | 0 | 0 | 1 |  | 3 | 24 | 0 | 7 | 0 |  |
| 2583 |  | 22.118 | 2.437 | 11.018175 | 88.981827 | 0 | 10.2423 | 1 | -0.5563001 | 4 | 100.73763 | 21.145 | 78.568 | 0 | 0 | 1 | 1 |  | | | | | | 108.8388057 | 0.800396376 | 99.67264287 | -0.052951245 |  | | | 0 | 0 | 0 | 0 | 3 |  | | | | |
| 2583 | 0 | 18.201 | 1.9299999 | 10.603812 | 89.396187 | 0 | 10.997947 | 1 | -0.5563001 | 4 | 100.73763 | 25.055555 | 72.099998 | 0 | 19 | 1 | 1 | 0 | 0 | 0 | 0 | 0 |  | | NA |  | NA |  | | 2 | 0 | 0 | 0 |  | 3 | 27 | 0 | 7 | 0 |  |
| 2584 |  | 21.105 | 2.347 | 11.120587 | 88.87941 | 1 | 17.330595 | 1 | -2.0030298 | 2 | 93.821365 | 21.58 | 76.999 | 0 | 0 | 3 | 1 |  | | | | | | 90.99885561 | -0.822704711 | 110.2646387 | 1.637528734 | 2 | 2 |  | 0 | 0 | 1 | 0 | 4 |  | | | | |
| 2584 | 1 | 18.605 | 1.86 | 9.9973125 | 90.002686 | 1 | 18.091719 | 1 | -2.0030298 | 2 | 93.821365 | 25.622225 | 69.519997 | 0 | 19 | 3 | 1 | 0 | 1 | 0 | 0 | 1 | 1 |  | NA |  | NA | 2 | 2 | 2 | 0 | 0 | 1 |  | 4 | 24 | 0 | 7 | 0 |  |
| 2584 | 1 | 21.250999 | 2.3 | 10.823021 | 89.176979 | 1 | 18.149214 | 1 | -2.0030298 | 2 | 93.821365 | 25.197224 | 72.059998 | 0 | 20 | 3 | 1 | 1 | 0 | 0 | 0 | 1 | 0 | 86.49503924 | -1.234581412 | 106.7768411 | 1.039394858 | 2 | 2 | 2 | 0 | 0 | 1 | 0 | 4 | 24 | 0 |  | | |
| 2584 | 1 | 26.187 | 2.79 | 10.654141 | 89.345856 | 1 | 18.16564 | 1 | -2.0030298 | 2 | 93.821365 | 24.169443 | 70.410004 | 0 | 21 | 3 | 1 | 1 | 0 | 1 | 0 | 0 | 1 |  | NA |  | NA | 2 | 2 | 2 | 0 | 0 | 1 |  | 4 | 25 | 0 | 7 | 0 |  |
| 2588 |  | 21.65 | 2.125 | 9.8152428 | 90.184761 | 0 | 9.4839153 | 1 | -2.591012 | 4 | 114.89134 | 19.305555 | 78.301 | 0 | 0 | 3 | 1 |  | | | | | | 113.5752832 | 1.213550523 | 106.0623468 | 1.039873474 | 2 | 2 |  | 0 | 0 | 0 | 0 | 3 |  | | | | |
| 2588 | 0 | 19.847 | 2.1500001 | 10.832872 | 89.16713 | 0 | 10.138262 | 1 | -2.591012 | 4 | 114.89134 | 25.431667 | 70.510002 | 0 | 19 | 3 | 1 | 0 | 0 | 0 | 0 | 0 |  | | NA |  | NA | 2 | 2 | 2 | 0 | 0 | 0 |  | 3 | 27 | 0 | 7 | 0 |  |
| 2588 | 0 | 22.118 | 2.47 | 11.167375 | 88.832626 | 0 | 10.173854 | 1 | -2.591012 | 4 | 114.89134 | 25.55611 | 71.639999 | 0 | 20 | 3 | 1 | 0 | 0 | 0 | 0 | 0 |  | 88.83252477 | -1.023094138 | 95.94982748 | -0.633504122 | 2 | 2 | 2 | 0 | 0 | 0 | 1 | 3 | 23 | 0 |  | | |
| 2588 | 1 | 24.926001 | 2.8 | 11.23325 | 88.766747 | 0 | 10.212183 | 1 | -2.591012 | 4 | 114.89134 | 24.136665 | 70.989998 | 0 | 21 | 3 | 1 | 1 | 1 | 1 | 1 | 1 |  | | NA |  | NA | 2 | 2 | 2 | 0 | 0 | 0 |  | 3 | 26 | 0 | 6.9000001 | 1 |  |
| 2589 |  | 20.872 | 2.064 | 9.8888464 | 90.111153 | 1 | 16.142368 | 0 | 0.33339408 | 2 | 81.570923 | 18.781666 | 81.005 | 0 | 0 | 3 | 1 |  | | | | | | 90.42318331 | -0.833614629 | 94.91342026 | -0.805419986 | 2 | 2 |  | 0 | 0 | 0 | 0 | 3 |  | | | | |
| 2589 | 0 | 17.976999 | 1.97 | 10.958447 | 89.04155 | 1 | 16.774813 | 0 | 0.33339408 | 2 | 81.570923 | 24.628889 | 74.300003 | 0 | 19 | 3 | 1 | 0 | 0 | 0 | 0 | 0 | 0 |  | NA |  | NA | 2 | 2 | 2 | 0 | 0 | 0 |  | 3 | 24 | 0 | 7 | 0 |  |
| 2589 | 0 | 21.167 | 2.47 | 11.669107 | 88.330894 | 1 | 16.810404 | 0 | 0.33339408 | 2 | 81.570923 | 23.990555 | 78.019997 | 0 | 20 | 3 | 1 | 0 | 0 | 0 | 0 | 0 | 0 | 93.29740327 | -0.578025141 | 76.92112828 | -2.830803368 | 2 | 2 | 2 | 0 | 0 | 0 | 0 | 3 | 24 | 0 |  | | |
| 2589 | 1 | 21.084 | 2.4400001 | 11.572757 | 88.427246 | 1 | 16.848734 | 0 | 0.33339408 | 2 | 81.570923 | 23.861664 | 75.559998 | 0 | 21 | 3 | 1 | 1 | 1 | 0 | 1 | 1 | 0 |  | NA |  | NA | 2 | 2 | 2 | 0 | 0 | 0 |  | 3 | 24 | 0 | 7 | 0 |  |
| 2602 |  | 21.708 | 2.661 | 12.258154 | 87.741844 | 0 | 10.524299 | 1 | -0.24929914 | 2 | 114.72319 | 21.646667 | 68.806 | 0 | 0 | 3 | 1 |  | | | | | | 110.8647888 | 0.9865214 | 110.6805087 | 1.921155839 | 2 | 2 |  | 0 | 0 | 0 | 0 | 4 |  | | | | |
| 2602 | 0 | 18.985001 | 1.87 | 9.8498812 | 90.150116 | 0 | 11.939767 | 1 | -0.24929914 | 2 | 114.72319 | 25.791113 | 67.910004 | 0 | 19 | 3 | 1 | 0 | 0 | 0 | 0 | 0 |  | | NA |  | NA | 2 | 2 | 2 | 0 | 0 | 0 |  | 4 | 26 | 0 | 7 | 0 |  |
| 2602 | 0 | 21.146 | 2.4200001 | 11.444245 | 88.555756 | 0 | 11.986311 | 1 | -0.24929914 | 2 | 114.72319 | 25.49778 | 70.18 | 0 | 20 | 3 | 1 | 0 | 0 | 0 | 0 | 0 |  | 109.1513291 | 0.83776342 | 107.8213619 | 1.354475551 | 2 | 2 | 2 | 0 | 0 | 0 | 0 | 4 | 27 | 0 |  | | |
| 2602 | 1 | 24.624001 | 2.8900001 | 11.736517 | 88.263481 | 1 | 12.016427 | 1 | -0.24929914 | 2 | 114.72319 | 24.280001 | 68.870003 | 0 | 21 | 3 | 1 | 1 | 0 | 0 | 0 | 0 | 0 |  | NA |  | NA | 2 | 2 | 2 | 0 | 0 | 0 |  | 4 | 24 | 0 | 7 | 0 |  |
| 2603 |  | 21.793 | 2.722 | 12.49025 | 87.50975 | 1 | 12.769336 | 1 | -2.6487656 | 2 | 104.36979 | 20.941668 | 68.146 | 0 | 0 | 3 | 1 |  | | | | | | 124.1575144 | 2.204339636 | 89.77811487 | -1.490554813 | 2 | 2 |  | 0 | 0 | 1 | 1 | 1 |  | | | | |
| 2603 | 0 | 17.454 | 1.47 | 8.4221382 | 91.577866 | 1 | 14.078029 | 1 | -2.6487656 | 2 | 104.36979 | 26.303333 | 66 | 0 | 19 | 3 | 1 | 0 | 0 | 0 | 0 | 0 | 0 |  | NA |  | NA | 2 | 2 | 2 | 0 | 0 | 1 |  | 1 | 25 | 0 | 7 | 0 |  |
| 2603 | 0 | 19.805 | 2.1700001 | 10.95683 | 89.043167 | 1 | 14.116359 | 1 | -2.6487656 | 2 | 104.36979 | 25.430557 | 69.269997 | 0 | 20 | 3 | 1 | 0 | 0 | 0 | 0 | 0 | 0 |  | NA |  | NA | 2 | 2 | 2 | 0 | 0 | 1 |  | 1 | 24 | 0 |  | | |
| 2603 | 0 | 22.341999 | 2.55 | 11.413482 | 88.586517 | 1 | 14.154689 | 1 | -2.6487656 | 2 | 104.36979 | 25.837225 | 67.07 | 0 | 21 | 3 | 1 | 0 | 0 | 0 | 0 | 0 | 0 |  | NA |  | NA | 2 | 2 | 2 | 0 | 0 | 1 |  | 1 | 24 | 0 | 7 | 0 |  |
| 2604 |  | 21.736 | 2.671 | 12.288369 | 87.711632 | 1 | 15.008898 | 1 | 0.32037216 | 4 | 104.15417 | 21.459444 | 69.357 | 0 | 0 | 3 | 1 |  | | | | | | 118.0040527 | 1.639284108 | 94.55681611 | -0.795455921 | 2 | 2 |  | 0 |  | 0 | 0 | 3 |  | | | | |
| 2604 | 0 | 17.938999 | 1.73 | 9.6437931 | 90.356209 | 1 | 16.380562 | 1 | 0.32037216 | 4 | 104.15417 | 25.902779 | 67.610001 | 0 | 19 | 3 | 1 | 0 | 0 | 0 | 0 | 0 | 0 |  | NA |  | NA | 2 | 2 | 2 | 0 |  | 0 |  | 3 | 24 | 0 | 7 | 0 |  |
| 2604 | 0 | 20.827 | 2.3 | 11.043357 | 88.956642 | 1 | 16.418892 | 1 | 0.32037216 | 4 | 104.15417 | 25.013889 | 71.32 | 0 | 20 | 3 | 1 | 0 | 0 | 0 | 0 | 0 | 0 | 110.6088492 | 0.965217088 | 101.4976057 | 0.225012099 | 2 | 2 | 2 | 0 |  | 0 | 0 | 3 | 25 | 0 |  | | |
| 2604 | 0 | 23.318001 | 2.6900001 | 11.536152 | 88.463844 | 1 | 16.459959 | 1 | 0.32037216 | 4 | 104.15417 | 25.214445 | 69.160004 | 0 | 21 | 3 | 1 | 0 | 0 | 0 | 0 | 0 | 0 |  | NA |  | NA | 2 | 2 | 2 | 0 |  | 0 |  | 3 | 24 | 0 | 7 | 0 |  |
| 2605 |  | 21.611 | 2.635 | 12.192864 | 87.807137 | 0 | 11.660506 | 1 | 1.4721255 | 3 | 105.66665 | 22.469999 | 72.479 | 0 | 0 | 3 | 1 |  | | | | | | 115.3738681 | 1.404277285 | 98.03510924 | -0.312714724 | 2 | 2 |  | 0 |  | 0 | 0 | 4 |  | | | | |
| 2605 | 0 | 18.827999 | 2.04 | 10.834928 | 89.16507 | 1 | 13.081451 | 1 | 1.4721255 | 3 | 105.66665 | 24.775 | 73.519997 | 0 | 19 | 3 | 1 | 0 | 0 | 0 | 0 | 0 | 0 |  | NA |  | NA | 2 | 2 | 2 | 0 |  | 0 |  | 4 | 24 | 0 | 7 | 0 |  |
| 2605 | 1 | 21.393999 | 2.5 | 11.68552 | 88.314484 | 1 | 13.119781 | 1 | 1.4721255 | 3 | 105.66665 | 24.706667 | 74.919998 | 0 | 20 | 3 | 1 | 0 | 0 | 0 | 0 | 1 | 0 | 107.7428162 | 0.709027327 | 94.94808208 | -0.767251946 | 2 | 2 | 2 | 0 |  | 0 | 0 | 4 | 23 | 0 |  | | |
| 2605 | 0 | 25.186001 | 2.79 | 11.077582 | 88.922417 | 1 | 13.152636 | 1 | 1.4721255 | 3 | 105.66665 | 23.316113 | 74.110001 | 0 | 21 | 3 | 1 | 0 | 0 | 0 | 0 | 0 | 0 |  | NA |  | NA | 2 | 2 | 2 | 0 |  | 0 |  | 4 | 24 | 0 | 6.9000001 | 1 |  |
| 2606 |  | 22.131 | 2.731 | 12.340157 | 87.659843 | 1 | 16.845995 | 0 | -0.57701868 | 4 | 110.90308 | 23.007778 | 71.803 | 0 | 0 | 3 | 1 |  | | | | | | 124.0203834 | 2.025521931 | 95.43247101 | -0.723082192 | 2 | 2 |  | 0 | 0 | 0 | 0 | 4 |  | | | | |
| 2606 | 0 | 22.219 | 2.6900001 | 12.106756 | 87.893242 | 1 | 18.277891 | 0 | -0.57701868 | 4 | 110.90308 | 24.596113 | 73.849998 | 0 | 19 | 3 | 1 | 0 | 0 | 0 | 0 | 0 | 0 |  | NA |  | NA | 2 | 2 | 2 | 0 | 0 | 0 |  | 4 | 24 | 0 | 7 | 0 |  |
| 2606 | 0 | 26.187 | 3.29 | 12.563486 | 87.436516 | 1 | 18.316221 | 0 | -0.57701868 | 4 | 110.90308 | 23.778891 | 74.459999 | 0 | 20 | 3 | 1 | 0 | 0 | 0 | 0 | 0 | 0 | 111.2853678 | 0.955172417 | 93.62432414 | -0.970545908 | 2 | 2 | 2 | 0 | 0 | 0 | 0 | 4 | 24 | 0 |  | | |
| 2606 | 0 | 27.548 | 3.47 | 12.596196 | 87.403801 | 1 | 18.354551 | 0 | -0.57701868 | 4 | 110.90308 | 22.664444 | 74.459999 | 0 | 21 | 3 | 1 | 0 | 0 | 0 | 0 | 0 | 0 |  | NA |  | NA | 2 | 2 | 2 | 0 | 0 | 0 |  | 4 | 24 | 0 | 7 | 0 |  |
| 2607 |  | 21.552 | 2.706 | 12.555679 | 87.444321 | 1 | 15.570157 | 0 | -2.9777105 | 2 | 93.459137 | 18.747778 | 71.166 | 0 | 0 | 3 | 1 |  | | | | | | 110.8041546 | 0.926493896 | 92.09046239 | -1.211144673 | 2 | 2 |  | 0 | 0 | 0 | 0 | 2 |  | | | | |
| 2607 | 0 | 17.764 | 1.75 | 9.8513851 | 90.148613 | 1 | 16.985626 | 0 | -2.9777105 | 2 | 93.459137 | 26.208336 | 65.900002 | 0 | 19 | 3 | 1 | 0 | 0 | 0 | 0 | 0 | 0 |  | NA |  | NA | 2 | 2 | 2 | 0 | 0 | 0 |  | 2 | 24 | 0 | 7 | 0 |  |
| 2607 | 0 | 21.563 | 2.3399999 | 10.851922 | 89.148079 | 1 | 17.032169 | 0 | -2.9777105 | 2 | 93.459137 | 26.034445 | 68.050003 | 0 | 20 | 3 | 1 |  | | 0 | 0 | 0 |  | 97.50934366 | -0.214462077 | 90.96799404 | -1.344180882 | 2 | 2 | 2 | 0 | 0 | 0 | 0 | 2 | 21 | 0 |  | | |
| 2607 | 0 | 25.360001 | 2.8900001 | 11.395899 | 88.604103 | 1 | 17.048597 | 0 | -2.9777105 | 2 | 93.459137 | 24.837778 | 67.290001 | 0 | 21 | 3 | 1 | 0 | 0 | 0 | 0 | 0 | 0 |  | NA |  | NA | 2 | 2 | 2 | 0 | 0 | 0 |  | 2 | 24 | 0 | 7 | 0 |  |
| 2608 |  | 21.583 | 2.734 | 12.667377 | 87.332626 | 1 | 16.008213 | 0 | 1.5522748 | 2 | 90.047951 | 18.173334 | 71.545 | 0 | 0 | 3 | 1 |  | | | | | | 96.07698651 | -0.339929729 | 100.2654074 | 0.045266506 | 2 | 2 |  | 0 | 0 | 1 | 0 | 4 |  | | | | |
| 2608 | 1 | 17.982 | 1.8099999 | 10.06562 | 89.93438 | 1 | 17.423683 | 0 | 1.5522748 | 2 | 90.047951 | 26.282776 | 65.650002 | 0 | 19 | 3 | 1 | 1 | 1 | 1 | 1 | 1 | 1 |  | NA |  | NA | 2 | 2 | 2 | 0 | 0 | 1 |  | 4 | 24 | 0 | 7 | 0 |  |
| 2608 | 1 | 21.542999 | 2.3199999 | 10.769159 | 89.230843 | 1 | 17.459274 | 0 | 1.5522748 | 2 | 90.047951 | 26.165001 | 67.93 | 0 | 20 | 3 | 1 | 1 | 1 | 1 | 1 | 1 | 1 | 77.11730609 | -2.005892242 | 88.47795428 | -1.652259242 | 2 | 2 | 2 | 0 | 0 | 1 | 1 | 4 | 24 | 0 |  | | |
| 2608 | 1 | 24.409 | 2.76 | 11.307304 | 88.692696 | 1 | 17.497604 | 0 | 1.5522748 | 2 | 90.047951 | 24.794998 | 67.519997 | 0 | 21 | 3 | 1 | 1 | 1 | 1 | 1 | 1 | 1 |  | NA |  | NA | 2 | 2 | 2 | 0 | 0 | 1 |  | 4 | 22 | 0 | 6.8000002 | 1 |  |
| 2609 |  | 21.739 | 2.672 | 12.291274 | 87.708725 | 1 | 14.042437 | 1 | -3.8274689 | 2 | 103.33264 | 19.995556 | 71.88 | 0 | 0 | 3 | 1 |  | | | | | | 108.8743189 | 0.811223851 | 101.7045983 | 0.269481281 | 2 | 2 |  | 1 | 0 | 0 | 0 | 3 |  | | | | |
| 2609 | 0 | 18.091999 | 1.84 | 10.170241 | 89.829758 | 1 | 15.463381 | 1 | -3.8274689 | 2 | 103.33264 | 25.436668 | 69.309998 | 0 | 19 | 3 | 1 | 0 | 0 | 0 | 0 | 0 | 0 |  | NA |  | NA | 2 | 2 | 2 | 1 | 0 | 0 |  | 3 | 24 | 0 | 7 | 0 |  |
| 2609 | 1 | 21.252001 | 2.26 | 10.634293 | 89.365707 | 1 | 15.498973 | 1 | -3.8274689 | 2 | 103.33264 | 25.346111 | 71.18 | 0 | 20 | 3 | 1 | 1 | 0 | 1 | 0 | 1 | 0 | 112.5694078 | 1.144278296 | 100.7786119 | 0.117856049 | 2 | 2 | 2 | 1 | 0 | 0 | 0 | 3 | 25 | 0 |  | | |
| 2609 | 0 | 25.284 | 2.7 | 10.67869 | 89.321312 | 1 | 15.537303 | 1 | -3.8274689 | 2 | 103.33264 | 23.867777 | 70.620003 | 0 | 21 | 3 | 1 | 0 | 0 | 0 | 0 | 0 | 0 |  | NA |  | NA | 2 | 2 | 2 | 1 | 0 | 0 |  | 3 | 24 | 0 | 7 | 0 |  |
| 2610 |  | 21.383 | 2.645 | 12.369639 | 87.630363 | 0 | 11.728952 | 0 | 0.29678264 | 3 | 111.35112 | 23.639999 | 68.599 | 0 | 0 | 3 | 1 |  | | | | | | 123.3082967 | 2.028921599 | 97.64167618 | -0.405324945 | 2 | 2 |  | 0 | 0 | 0 | 0 | 4 |  | | | | |
| 2610 | 1 | 17.454 | 1.88 | 10.77117 | 89.228828 | 1 | 13.138946 | 0 | 0.29678264 | 3 | 111.35112 | 25.21278 | 71.68 | 0 | 19 | 3 | 1 | 1 | 1 | 1 | 1 | 1 | 1 |  | NA |  | NA | 2 | 2 | 2 | 0 | 0 | 0 |  | 4 | 24 | 0 | 7 | 0 |  |
| 2610 | 0 | 20.687 | 2.3599999 | 11.408131 | 88.591873 | 1 | 13.174538 | 0 | 0.29678264 | 3 | 111.35112 | 24.993332 | 74.230003 | 0 | 20 | 3 | 1 | 0 | 0 | 0 | 0 | 0 | 0 | 118.8030868 | 1.61861423 | 99.98160427 | -0.003202093 | 2 | 2 | 2 | 0 | 0 | 0 | 0 | 4 | 24 | 0 |  | | |
| 2610 | 0 | 20.830999 | 2.3800001 | 11.425281 | 88.574722 | 1 | 13.212868 | 0 | 0.29678264 | 3 | 111.35112 | 24.406666 | 72.970001 | 0 | 21 | 3 | 1 | 0 | 0 | 0 | 0 | 0 | 0 |  | NA |  | NA | 2 | 2 | 2 | 0 | 0 | 0 |  | 4 | 24 | 0 | 7 | 0 |  |
| 2612 |  | 21.804 | 2.791 | 12.800404 | 87.199593 | 1 | 12.457221 | 0 | -0.015570504 | 3 | 102.46796 | 22.123333 | 77.21 | 0 | 0 | 3 | 1 |  | | | | | | 111.350498 | 0.992892884 | 98.21642452 | -0.307195637 | 2 | 2 |  | 0 | 0 | 0 | 0 | 4 |  | | | | |
| 2612 | 0 | 18.316 | 2.0699999 | 11.301594 | 88.69841 | 1 | 13.864476 | 0 | -0.015570504 | 3 | 102.46796 | 23.950001 | 77.610001 | 0 | 19 | 3 | 1 | 0 | 0 | 0 | 0 | 0 | 0 |  | NA |  | NA | 2 | 2 | 2 | 0 | 0 | 0 |  | 4 | 24 | 0 | 7 | 0 |  |
| 2612 | 0 | 21.509001 | 2.5999999 | 12.087962 | 87.912041 | 1 | 13.902806 | 0 | -0.015570504 | 3 | 102.46796 | 23.23889 | 81.620003 | 0 | 20 | 3 | 1 | 0 | 0 | 0 | 0 | 0 | 0 | 91.61344928 | -0.738878322 | 106.745827 | 1.293466935 | 2 | 2 | 2 | 0 | 0 | 0 | 0 | 4 | 24 | 0 |  | | |
| 2612 | 0 | 22.367001 | 2.6400001 | 11.803102 | 88.196899 | 1 | 13.941136 | 0 | -0.015570504 | 3 | 102.46796 | 22.936668 | 78.800003 | 0 | 21 | 3 | 1 | 0 | 0 | 0 | 0 | 0 | 0 |  | NA |  | NA | 2 | 2 | 2 | 0 | 0 | 0 |  | 4 | 23 | 0 | 7 | 0 |  |
| 2613 |  | 20.311 | 2.434 | 11.983654 | 88.016342 | 1 | 16.720055 | 0 | 1.7815342 | 2 | 142.57253 | 25.227222 | 63.592 | 0 | 0 | 3 | 1 |  | | | | | | 168.4117649 | 5.601734045 | 90.45540961 | -1.41470023 | 2 | 2 |  | 1 | 0 | 0 |  | 1 |  | | | | |
| 2613 | 0 | 16.089001 | 1.73 | 10.752687 | 89.247314 | 1 | 18.138262 | 0 | 1.7815342 | 2 | 142.57253 | 25.689444 | 70.010002 | 0 | 19 | 3 | 1 | 0 | 0 | 0 | 0 | 0 | 0 |  | NA |  | NA | 2 | 2 | 2 | 1 | 0 | 0 |  | 1 | 24 | 0 | 7 | 0 |  |
| 2613 | 1 | 19.417 | 2.1700001 | 11.175775 | 88.824226 | 1 | 18.176592 | 0 | 1.7815342 | 2 | 142.57253 | 25.922224 | 70.93 | 0 | 20 | 3 | 1 | 1 | 0 | 1 | 0 | 1 | 0 | 126.8160824 | 2.240719434 | 82.42848979 | -2.307450506 | 2 | 2 | 2 | 1 | 0 | 0 | 1 | 1 | 23 | 0 |  | | |
| 2613 | 1 | 21.135 | 2.3900001 | 11.308257 | 88.691742 | 1 | 18.214922 | 0 | 1.7815342 | 2 | 142.57253 | 24.451111 | 71.349998 | 0 | 21 | 3 | 1 | 1 | 0 | 1 | 0 | 1 | 0 |  | NA |  | NA | 2 | 2 | 2 | 1 | 0 | 0 |  | 1 | 23 | 0 | 7 | 0 |  |
| 2614 |  | 21.715 | 2.679 | 12.337094 | 87.662903 | 1 | 14.316222 | 1 | -1.9834018 | 4 | 111.34242 | 20.252222 | 70.195 | 0 | 0 | 3 |  | | | | | | | 143.4192296 | 3.936735442 | 83.0811029 | -2.267852235 | 2 | 2 |  | 0 | 0 | 0 | 0 | 1 |  | | | | |
| 2614 | 0 | 17.438999 | 1.62 | 9.2895241 | 90.71048 | 1 | 15.709787 | 1 | -1.9834018 | 4 | 111.34242 | 26.065001 | 66.910004 | 0 | 19 | 3 |  | 0 | 0 | 0 | 0 | 0 | 0 |  | NA |  | NA | 2 | 2 | 2 | 0 | 0 | 0 |  | 1 | 24 | 0 | 7 | 0 |  |
| 2614 | 0 | 20.003 | 2.1300001 | 10.648403 | 89.351593 | 1 | 15.748117 | 1 | -1.9834018 | 4 | 111.34242 | 25.267778 | 70.18 | 0 | 20 | 3 |  | 0 | 0 | 0 | 0 | 0 | 0 | 137.7018367 | 3.413454606 | 88.50668903 | -1.574801055 | 2 | 2 | 2 | 0 | 0 | 0 | 0 | 1 | 24 | 0 |  | | |
| 2614 | 0 | 22.945999 | 2.5599999 | 11.156629 | 88.843369 | 1 | 15.789186 | 1 | -1.9834018 | 4 | 111.34242 | 25.395554 | 68.410004 | 0 | 21 | 3 |  | 0 | 0 | 0 | 0 | 0 | 0 |  | NA |  | NA | 2 | 2 | 2 | 0 | 0 | 0 |  | 1 | 24 | 0 | 7 | 0 |  |
| 2615 |  | 25.336 | 3.214 | 12.685507 | 87.314491 | 0 | 11.45243 | 1 | -1.7033793 | 3 | 124.83216 | 17.852777 | 75.97 | 0 | 0 | 2 | 1 |  | | | | | | 137.0565338 | 3.366251643 | 96.92455767 | -0.485104493 | 2 | 2 |  | 0 | 0 | 1 | 0 | 3 |  | | | | |
| 2615 | 0 | 20.263 | 2.0999999 | 10.363717 | 89.636284 | 1 | 12.678987 | 1 | -1.7033793 | 3 | 124.83216 | 25.061666 | 71.660004 | 0 | 20 | 2 | 1 | 0 | 0 | 0 | 0 | 0 | 0 | 122.6521143 | 2.067753577 | 93.21469125 | -1.021617492 | 2 | 2 | 2 | 0 | 0 | 1 | 0 | 3 | 24 | 0 |  | | |
| 2615 | 0 | 24.275 | 2.5899999 | 10.669413 | 89.330589 | 1 | 12.720055 | 1 | -1.7033793 | 3 | 124.83216 | 24.695557 | 70.059998 | 0 | 21 | 2 | 1 | 0 | 0 | 0 | 0 | 0 | 0 |  | NA |  | NA | 2 | 2 | 2 | 0 | 0 | 1 |  | 3 | 25 | 0 | 7 | 0 |  |
| 2616 |  | 22.11 | 2.558 | 11.569426 | 88.430573 | 1 | 16.898016 | 1 | -3.4616709 | 2 | 126.37405 | 22.263889 | 70.996 | 0 | 0 | 3 | 1 |  | | | | | | 138.7822979 | 3.505798078 | 95.60151599 | -0.62942082 | 2 | 2 |  | 1 |  | 0 | 0 | 3 |  | | | | |
| 2616 | 0 | 19.004999 | 1.9299999 | 10.155222 | 89.84478 | 1 | 18.294319 | 1 | -3.4616709 | 2 | 126.37405 | 25.503332 | 70.059998 | 0 | 19 | 3 | 1 | 0 | 0 | 0 | 0 | 0 | 0 |  | NA |  | NA | 2 | 2 | 2 | 1 |  | 0 |  | 3 | 25 | 0 | 7 | 0 |  |
| 2616 | 0 | 21.676001 | 2.3499999 | 10.841483 | 89.158516 | 1 | 18.335386 | 1 | -3.4616709 | 2 | 126.37405 | 25.45389 | 71.410004 | 0 | 20 | 3 | 1 | 0 | 0 | 0 | 0 | 0 | 0 |  | NA |  | NA | 2 | 2 | 2 | 1 |  | 0 |  | 3 | 24 | 0 |  | | |
| 2616 | 0 | 29.096001 | 2.8099999 | 9.6576843 | 90.342316 | 1 | 18.373716 | 1 | -3.4616709 | 2 | 126.37405 | 23.871668 | 70.989998 | 0 | 21 | 3 | 1 | 0 | 0 | 0 | 0 | 0 | 0 |  | NA |  | NA | 2 | 2 | 2 | 1 |  | 0 |  | 3 | 25 | 0 | 7 | 0 |  |
| 2617 |  | 21.71 | 2.705 | 12.459696 | 87.540306 | 1 | 17.637236 | 0 | -3.0034351 | 4 | 110.01615 | 22.861111 | 70.267 | 0 | 0 | 3 | 0 |  | | | | | | 120.707069 | 1.744330578 | 97.46215426 | -0.409109699 | 2 | 2 |  | 0 | 0 | 0 | 0 | 3 |  | | | | |
| 2617 | 0 | 18.422001 | 2.01 | 10.910867 | 89.089134 | 1 | 19.060917 | 0 | -3.0034351 | 4 | 110.01615 | 25.021112 | 72.480003 | 0 | 19 | 3 | 0 | 0 | 0 | 0 | 0 | 0 | 0 |  | NA |  | NA | 2 | 2 | 2 | 0 | 0 | 0 |  | 3 | 24 | 0 | 7 | 0 |  |
| 2617 | 0 | 21.141001 | 2.47 | 11.683458 | 88.316544 | 1 | 19.099247 | 0 | -3.0034351 | 4 | 110.01615 | 24.909998 | 73.82 | 0 | 20 | 3 | 0 | 0 | 0 | 0 | 0 | 0 | 0 | 98.84741641 | -0.098096164 | 95.96259934 | -0.623683421 | 2 | 2 | 2 | 0 | 0 | 0 | 0 | 3 | 24 | 0 |  | | |
| 2617 | 0 | 24.377001 | 2.79 | 11.445214 | 88.554787 | 1 | 19.129364 | 0 | -3.0034351 | 4 | 110.01615 | 23.51111 | 73.169998 | 0 | 21 | 3 | 0 | 0 | 0 | 0 | 0 | 0 | 0 |  | NA |  | NA | 2 | 2 | 2 | 0 | 0 | 0 |  | 3 | 24 | 0 | 7 | 0 |  |
| 2618 |  | 21.81 | 2.736 | 12.544704 | 87.455299 | 1 | 13.341547 | 0 | 1.4795393 | 3 | 115.17204 | 17.725 | 74.391 | 0 | 0 | 3 | 0 |  | | | | | | 128.1073173 | 2.412189747 | 96.75131664 | -0.542744827 | 2 | 2 |  | 1 | 0 | 0 | 0 |  | | | | | |
| 2618 | 0 | 17.67 | 1.78 | 10.073571 | 89.92643 | 1 | 14.75154 | 0 | 1.4795393 | 3 | 115.17204 | 25.315001 | 69.07 | 0 | 19 | 3 | 0 | 0 | 0 | 0 | 0 | 0 | 0 |  | NA |  | NA | 2 | 2 | 2 | 1 | 0 | 0 |  | | 24 | 0 | 7 | 0 |  |
| 2618 | 0 | 21.143 | 2.1800001 | 10.310741 | 89.689255 | 1 | 14.78987 | 0 | 1.4795393 | 3 | 115.17204 | 25.025 | 71.629997 | 0 | 20 | 3 | 0 | 0 | 0 | 0 | 0 | 0 | 0 | 121.8854615 | 1.866305653 | 98.15201701 | -0.309657854 | 2 | 2 | 2 | 1 | 0 | 0 | 0 |  | 24 | 0 |  | | |
| 2618 | 0 | 25.764999 | 2.96 | 11.488454 | 88.511543 | 1 | 14.806297 | 0 | 1.4795393 | 3 | 115.17204 | 23.889999 | 70.559998 | 0 | 21 | 3 | 0 | 0 | 0 | 0 | 0 | 0 | 0 |  | NA |  | NA | 2 | 2 | 2 | 1 | 0 | 0 |  | | 24 | 0 | 7 | 0 |  |
| 2620 |  | 21.869 | 2.697 | 12.332525 | 87.667473 | 1 | 16.878851 | 0 | -2.0030298 | 3 | 80.624084 | 19.046667 | 73.146 | 0 | 0 | 3 | 1 |  | | | | | | 102.62407 | 0.225103938 | 83.94479822 | -2.175217278 | 2 | 2 |  | 0 | 0 | 1 | 1 | 1 |  | | | | |
| 2620 | 0 | 18.002001 | 1.8099999 | 10.054438 | 89.945564 | 1 | 18.102669 | 0 | -2.0030298 | 3 | 80.624084 | 25.411665 | 69.129997 | 0 | 19 | 3 | 1 | 0 | 0 | 0 | 0 | 0 | 0 |  | NA |  | NA | 2 | 2 | 2 | 0 | 0 | 1 |  | 1 | 24 | 0 | 7 | 0 |  |
| 2620 | 1 | 21.211 | 2.22 | 10.466268 | 89.53373 | 1 | 18.140999 | 0 | -2.0030298 | 3 | 80.624084 | 25.194443 | 71.449997 | 0 | 20 | 3 | 1 | 0 | 0 | 0 | 0 | 1 | 0 | 116.5557742 | 1.392789694 | 91.88221498 | -1.202671509 | 2 | 2 | 2 | 0 | 0 | 1 | 0 | 1 | 24 | 0 |  | | |
| 2620 | 1 | 25.575001 | 2.6800001 | 10.478983 | 89.521019 | 1 | 18.176592 | 0 | -2.0030298 | 3 | 80.624084 | 23.716667 | 70.879997 | 0 | 21 | 3 | 1 | 1 | 1 | 1 | 1 | 0 | 1 |  | NA |  | NA | 2 | 2 | 2 | 0 | 0 | 1 |  | 1 | 23 | 0 | 7 | 0 |  |
| 2621 |  | 21.529 | 2.647 | 12.295044 | 87.704956 | 0 | 10.151951 | 0 | -1.5018775 | 2 | 114.47187 | 18.252222 | 73.706 | 0 | 0 | 3 | 1 |  | | | | | | 130.7076603 | 2.664839526 | 93.78523784 | -1.01421404 |  | | | | | 0 | 0 |  | | | | | |
| 2621 | 1 | 17.608 | 1.77 | 10.052249 | 89.947754 | 0 | 11.575633 | 0 | -1.5018775 | 2 | 114.47187 | 25.409445 | 68.690002 | 0 | 19 | 3 | 1 | 1 | 0 | 0 | 0 | 1 |  | | NA |  | NA |  | | 2 |  | | 0 |  | | 26 | 0 | 7 | 0 |  |
| 2621 | 0 | 21.072001 | 2.1900001 | 10.392939 | 89.607063 | 0 | 11.616701 | 0 | -1.5018775 | 2 | 114.47187 | 25.046112 | 71.550003 | 0 | 20 | 3 | 1 | 0 | 0 | 0 | 0 | 0 |  | 116.4593865 | 1.439440786 | 96.02181992 | -0.666167585 |  | | 2 |  | | 0 | 0 |  | 26 | 0 |  | | |
| 2621 | 0 | 24.733 | 2.6199999 | 10.593134 | 89.406868 | 0 | 11.655031 | 0 | -1.5018775 | 2 | 114.47187 | 23.528887 | 71.029999 | 0 | 21 | 3 | 1 | 0 | 0 | 0 | 0 | 0 |  | | NA |  | NA |  | | 2 |  | | 0 |  | | 26 | 0 | 7 | 0 |  |
| 2622 |  | 22.423 | 2.852 | 12.719083 | 87.280914 | 0 | 11.594798 | 1 | -2.4999385 | 3 | 93.884758 | 23.101667 | 68.752 | 0 | 0 | 3 | 1 |  | | | | | | 110.4001167 | 0.950906319 | 90.90373795 | -1.354814858 | 1 | 2 |  | 0 | 0 | 1 | 0 | 1 |  | | | | |
| 2622 | 1 | 18.535 | 2.01 | 10.844349 | 89.155655 | 1 | 12.898015 | 1 | -2.4999385 | 3 | 93.884758 | 25.326666 | 70.370003 | 0 | 19 | 3 | 1 | 0 | 0 | 1 | 1 | 1 | 0 |  | NA |  | NA | 1 | 2 | 2 | 0 | 0 | 1 |  | 1 | 24 | 0 | 7 | 0 |  |
| 2622 | 1 | 21.791 | 2.48 | 11.380845 | 88.619156 | 1 | 12.936345 | 1 | -2.4999385 | 3 | 93.884758 | 25.016666 | 73.309998 | 0 | 20 | 3 | 1 | 1 | 0 | 1 | 0 | 1 | 0 |  | NA |  | NA | 1 | 2 | 1 | 0 | 0 | 1 |  | 1 | 13 | 1 |  | | |
| 2622 | 0 | 23.638 | 2.75 | 11.63381 | 88.366188 | 1 | 12.974675 | 1 | -2.4999385 | 3 | 93.884758 | 24.365557 | 71.720001 | 0 | 21 | 3 | 1 | 0 | 0 | 0 | 0 | 0 | 0 |  | NA |  | NA | 1 | 2 | 1 | 0 | 0 | 1 |  | 1 | 24 | 0 | 7 | 0 |  |
| 2623 |  | 21.789 | 2.719 | 12.478774 | 87.521225 | 1 | 15.392197 | 0 | 2.3747432 | 2 | 95.011971 | 23.865 | 66.9 | 0 | 0 | 3 | 1 |  | | | | | | 121.9847152 | 1.87049061 | 82.49070931 | -2.345149927 | 2 | 2 |  | 0 | 0 | 0 | 1 | 1 |  | | | | |
| 2623 | 0 | 17.811001 | 1.9400001 | 10.892145 | 89.107857 | 1 | 16.769337 | 0 | 2.3747432 | 2 | 95.011971 | 25.501112 | 70.129997 | 0 | 19 | 3 | 1 | 0 | 0 | 0 | 0 | 0 | 0 |  | NA |  | NA | 2 | 2 | 2 | 0 | 0 | 0 |  | 1 | 24 | 0 | 7 | 0 |  |
| 2623 | 1 | 21.158001 | 2.4100001 | 11.390491 | 88.609512 | 1 | 16.807667 | 0 | 2.3747432 | 2 | 95.011971 | 25.219442 | 72.870003 | 0 | 20 | 3 | 1 | 1 | 1 | 1 | 0 | 0 | 1 | 135.0397797 | 2.927072747 | 89.82887478 | -1.489540987 | 2 | 2 | 2 | 0 | 0 | 0 | 0 | 1 | 25 | 0 |  | | |
| 2623 | 0 | 22.676001 | 2.6300001 | 11.598166 | 88.401833 | 1 | 16.848734 | 0 | 2.3747432 | 2 | 95.011971 | 24.423332 | 71.790001 | 0 | 21 | 3 | 1 | 0 | 0 | 0 | 0 | 0 | 0 |  | NA |  | NA | 2 | 2 | 2 | 0 | 0 | 0 |  | 1 | 24 | 0 | 7 | 0 |  |
| 2624 |  | 21.665 | 2.741 | 12.651742 | 87.348259 | 0 | 11.137577 | 1 | 1.5357243 | 3 | 110.75979 | 18.395555 | 71.692 | 0 | 0 | 3 | 1 |  | | | | | | 119.1174152 | 1.740731838 | 99.33744798 | -0.107019816 | 2 | 2 |  | 0 | 0 | 0 | 1 | 3 |  | | | | |
| 2624 | 0 | 18.191999 | 1.83 | 10.059367 | 89.940636 | 1 | 12.555783 | 1 | 1.5357243 | 3 | 110.75979 | 26.075554 | 66.519997 | 0 | 19 | 3 | 1 | 0 | 0 | 0 | 0 | 0 | 0 |  | NA |  | NA | 2 | 2 | 2 | 0 | 0 | 0 |  | 3 | 24 | 0 | 7 | 0 |  |
| 2624 | 1 | 21.823999 | 2.4000001 | 10.997068 | 89.00293 | 1 | 12.596851 | 1 | 1.5357243 | 3 | 110.75979 | 26.025 | 68.129997 | 0 | 20 | 3 | 1 | 1 | 0 | 0 | 1 | 1 | 0 | 87.34781539 | -1.165170936 | 105.5058012 | 0.921314293 | 2 | 2 | 2 | 0 | 0 | 0 | 0 | 3 | 21 | 0 |  | | |
| 2624 | 0 | 24.707001 | 2.76 | 11.170923 | 88.829079 | 1 | 12.635181 | 1 | 1.5357243 | 3 | 110.75979 | 24.003334 | 68.93 | 0 | 21 | 3 | 1 | 0 | 0 | 0 | 0 | 0 | 0 |  | NA |  | NA | 2 | 2 | 2 | 0 | 0 | 0 |  | 3 | 24 | 0 | 7 | 0 |  |
| 2625 |  | 21.801 | 2.696 | 12.366405 | 87.633598 | 1 | 15.091034 | 0 | -2.7092869 | 3 | 115.64304 | 22.314444 | 71.743 | 0 | 0 | 2 | 1 |  | | | | | | 126.4188629 | 2.244188582 | 98.10749142 | -0.31666038 | 2 | 2 |  | 0 | 0 | 0 | 0 | 3 |  | | | | |
| 2625 | 1 | 21.375 | 2.49 | 11.649123 | 88.350876 | 1 | 16.553045 | 0 | -2.7092869 | 3 | 115.64304 | 24.860556 | 74.059998 | 0 | 20 | 2 | 1 | 1 | 0 | 0 | 1 | 1 | 0 | 107.4152484 | 0.632849866 | 100.0649036 | 0.010894212 | 2 | 2 | 2 | 0 | 0 | 0 | 0 | 3 | 25 | 0 |  | | |
| 2625 | 0 | 25.132 | 2.79 | 11.101385 | 88.898613 | 1 | 16.591375 | 0 | -2.7092869 | 3 | 115.64304 | 23.444443 | 73.349998 | 0 | 21 | 2 | 1 | 0 | 0 | 0 | 0 | 0 | 0 |  | NA |  | NA | 2 | 2 | 2 | 0 | 0 | 0 |  | 3 | 24 | 0 | 7 | 0 |  |
| 2626 |  | 21.62 | 2.679 | 12.391304 | 87.608696 | 0 | 10.340862 | 1 | -2.3131132 | 3 | 138.29706 | 23.918888 | 66.973 | 0 | 0 | 3 | 1 |  | | | | | | 141.2793528 | 3.715310539 | 105.0013513 | 0.852394669 | 2 | 2 |  | 0 | 0 | 0 | 0 | 4 |  | | | | |
| 2626 | 0 | 17.867001 | 1.95 | 10.913975 | 89.086021 | 0 | 11.676933 | 1 | -2.3131132 | 3 | 138.29706 | 25.452225 | 70.209999 | 0 | 19 | 3 | 1 | 0 | 0 | 0 | 0 | 0 |  | | NA |  | NA | 2 | 2 | 2 | 0 | 0 | 0 |  | 4 | 27 | 0 | 7 | 0 |  |
| 2626 | 0 | 21.299 | 2.4400001 | 11.455937 | 88.54406 | 0 | 11.715263 | 1 | -2.3131132 | 3 | 138.29706 | 25.133886 | 73.099998 | 0 | 20 | 3 | 1 | 0 | 0 | 0 | 0 | 0 |  | 84.76515336 | -1.403852138 | 98.48072843 | -0.241932612 | 2 | 2 | 2 | 0 | 0 | 0 | 0 | 4 | 20 | 0 |  | | |
| 2626 | 0 | 22.834 | 2.6700001 | 11.693089 | 88.306908 | 0 | 11.756331 | 1 | -2.3131132 | 3 | 138.29706 | 24.352222 | 71.970001 | 0 | 21 | 3 | 1 | 0 | 0 | 0 | 0 | 0 |  | | NA |  | NA | 2 | 2 | 2 | 0 | 0 | 0 |  | 4 | 25 | 0 | 6.9000001 | 1 |  |
| 2627 |  | 21.949 | 2.709 | 12.342248 | 87.657753 | 1 | 17.412731 | 0 | -3.3300688 | 2 | 114.37578 | 23.66 | 67.728 | 0 | 0 | 2 | 0 |  | | | | | | 124.529067 | 2.062282931 | 98.62832935 | -0.225104559 | 2 | 2 |  | 0 | 0 | 0 | 0 | 3 |  | | | | |
| 2627 | 0 | 18.249001 | 1.9400001 | 10.630719 | 89.369278 | 1 | 18.811773 | 0 | -3.3300688 | 2 | 114.37578 | 25.449444 | 70.150002 | 0 | 19 | 2 | 0 | 0 | 0 | 0 | 0 | 0 | 0 |  | NA |  | NA | 2 | 2 | 2 | 0 | 0 | 0 |  | 3 | 24 | 0 | 7 | 0 |  |
| 2627 | 0 | 21.343 | 2.3900001 | 11.198051 | 88.801949 | 1 | 18.850103 | 0 | -3.3300688 | 2 | 114.37578 | 25.234999 | 72.800003 | 0 | 20 | 2 | 0 | 0 | 0 | 0 | 0 | 0 | 0 |  | NA |  | NA | 2 | 2 | 2 | 0 | 0 | 0 |  | 3 | 24 | 0 |  | | |
| 2631 |  | 23.187 | 2.653 | 11.441756 | 88.558243 | 1 | 17.634497 | 1 | -4.2813001 | 2 | 118.32666 | 20.976667 | 72.944 | 0 | 0 | 2 | 0 |  | | | | | | 121.9641511 | 1.991518797 | 103.5867902 | 0.541918755 | 2 | 2 |  | 0 |  | 0 | 0 | 2 |  | | | | |
| 2631 | 1 | 21.082001 | 2.22 | 10.53031 | 89.469688 | 1 | 18.970568 | 1 | -4.2813001 | 2 | 118.32666 | 25.458887 | 71.449997 | 0 | 20 | 2 | 0 | 1 | 1 | 1 | 1 | 1 | 1 | 114.6723266 | 1.330856621 | 105.3729349 | 0.811988096 | 2 | 2 | 2 | 0 |  | 0 | 0 | 2 | 9 | 1 |  | | |
| 2631 | 0 | 26.271999 | 2.7 | 10.277102 | 89.7229 | 1 | 19.008898 | 1 | -4.2813001 | 2 | 118.32666 | 24.545555 | 70.139999 | 0 | 21 | 2 | 0 | 0 | 0 | 0 | 0 | 0 | 0 |  | NA |  | NA | 2 | 2 | 1 | 0 |  | 0 |  | 2 | 16 | 1 | 3.2 | 1 |  |
| 2632 |  | 21.723 | 2.343 | 10.785803 | 89.214195 | 1 | 15.843943 | 1 | -1.1673238 | 2 | 90.033676 | 23.066668 | 70.217 | 0 | 0 | 3 | 1 |  | | | | | | 103.8731823 | 0.353387181 | 92.49183664 | -1.063292085 | 2 | 2 |  | 0 | 0 | 0 | 0 | 2 |  | | | | |
| 2632 | 0 | 19.191 | 1.88 | 9.7962589 | 90.203743 | 1 | 17.18549 | 1 | -1.1673238 | 2 | 90.033676 | 25.830002 | 68.769997 | 0 | 19 | 3 | 1 | 0 | 0 | 0 | 0 | 0 | 0 |  | NA |  | NA | 2 | 2 | 2 | 0 | 0 | 0 |  | 2 | 24 | 0 | 7 | 0 |  |
| 2632 | 1 | 21.211 | 2.1800001 | 10.277686 | 89.722313 | 1 | 17.22382 | 1 | -1.1673238 | 2 | 90.033676 | 25.626665 | 70.949997 | 0 | 20 | 3 | 1 | 1 | 1 | 1 | 1 | 1 | 1 | 101.9835143 | 0.180779784 | 76.34212276 | -2.872201291 | 2 | 2 | 2 | 0 | 0 | 0 | 1 | 2 | 19 | 1 |  | | |
| 2632 | 1 | 27.502001 | 2.6900001 | 9.7811069 | 90.218895 | 1 | 17.26215 | 1 | -1.1673238 | 2 | 90.033676 | 24.659445 | 69.82 | 0 | 21 | 3 | 1 | 1 | 1 | 1 | 1 | 1 | 1 |  | NA |  | NA | 2 | 2 | 2 | 0 | 0 | 0 |  | 2 | 19 | 1 | 6.3000002 | 1 |  |
| 2633 |  | 21.748 | 2.521 | 11.59187 | 88.408127 | 1 | 12.867899 | 1 | -1.9491892 | 2 | 120.37479 | 21.040001 | 71.535 | 0 | 0 | 3 | 0 |  | | | | | | 125.8660991 | 2.359161777 | 102.3269726 | 0.378549512 | 2 | 2 |  | 0 | 0 | 0 | 0 |  | | | | | |
| 2633 | 0 | 18.6 | 1.85 | 9.9462366 | 90.053764 | 1 | 14.275154 | 1 | -1.9491892 | 2 | 120.37479 | 25.588888 | 69.059998 | 0 | 19 | 3 | 0 | 0 | 0 | 0 | 0 | 0 | 0 |  | NA |  | NA | 2 | 2 | 2 | 0 | 0 | 0 |  | | 24 | 0 | 7 | 0 |  |
| 2633 | 0 | 21.733 | 2.3099999 | 10.628997 | 89.371002 | 1 | 14.318959 | 1 | -1.9491892 | 2 | 120.37479 | 25.422224 | 70.790001 | 0 | 20 | 3 | 0 | 0 | 0 | 0 | 0 | 0 | 0 | 102.6318512 | 0.240829618 | 111.0109774 | 1.865787867 | 2 | 2 | 2 | 0 | 0 | 0 | 0 |  | 23 | 0 |  | | |
| 2633 | 0 | 28.212 | 2.71 | 9.6058416 | 90.394157 | 1 | 14.349076 | 1 | -1.9491892 | 2 | 120.37479 | 23.82 | 70.489998 | 0 | 21 | 3 | 0 | 0 | 0 | 0 | 0 | 0 | 0 |  | NA |  | NA | 2 | 2 | 2 | 0 | 0 | 0 |  | | 23 | 0 | 7 | 0 |  |
| 2634 |  | 21.75 | 2.558 | 11.76092 | 88.239082 | 0 | 10.954141 | 1 | -3.2270544 | 3 | 126.68741 | 20.547777 | 71.893 | 0 | 0 | 2 |  | | | | | | | 142.5441788 | 3.849852311 | 92.74643513 | -1.100110418 | 2 | 2 |  | 0 | 0 | 0 | 0 | 2 |  | | | | |
| 2634 | 0 | 20.292 | 2.0999999 | 10.348906 | 89.651093 | 1 | 12.386037 | 1 | -3.2270544 | 3 | 126.68741 | 25.078888 | 71.660004 | 0 | 20 | 2 |  | 0 | 0 | 0 | 0 | 0 | 0 | 118.9415597 | 1.730036952 | 97.27105695 | -0.423513272 | 2 | 2 | 2 | 0 | 0 | 0 | 0 | 2 | 24 | 0 |  | | |
| 2634 | 0 | 24.007999 | 2.5799999 | 10.746418 | 89.253586 | 1 | 12.421629 | 1 | -3.2270544 | 3 | 126.68741 | 24.839998 | 70.050003 | 0 | 21 | 2 |  | 0 | 0 | 0 | 0 | 0 | 0 |  | NA |  | NA | 2 | 2 | 2 | 0 | 0 | 0 |  | 2 | 25 | 0 | 7 | 0 |  |
| 2635 |  | 21.71 | 2.625 | 12.091202 | 87.908798 | 1 | 12.213552 | 1 | 1.0818928 | 3 | 118.43637 | 21.360001 | 70.118 | 0 | 0 | 3 |  | | | | | | | 140.7955183 | 3.709459141 | 90.14856307 | -1.450880239 | 2 | 2 |  | 0 | 0 | 0 | 0 | 1 |  | | | | |
| 2635 | 0 | 17.735001 | 1.71 | 9.6419506 | 90.358047 | 1 | 13.601643 | 1 | 1.0818928 | 3 | 118.43637 | 25.807779 | 68.25 | 0 | 19 | 3 |  | 0 | 0 | 0 | 0 | 0 | 0 |  | NA |  | NA | 2 | 2 | 2 | 0 | 0 | 0 |  | 1 | 24 | 0 | 7 | 0 |  |
| 2635 | 1 | 20.163 | 2.1600001 | 10.712692 | 89.287308 | 1 | 13.639973 | 1 | 1.0818928 | 3 | 118.43637 | 25.052223 | 71.32 | 0 | 20 | 3 |  | 0 | 0 | 0 | 0 | 1 | 0 | 127.6118422 | 2.514725666 | 87.73697715 | -1.731480735 | 2 | 2 | 2 | 0 | 0 | 0 | 0 | 1 | 23 | 0 |  | | |
| 2635 | 0 | 23.01 | 2.5599999 | 11.125597 | 88.874405 | 1 | 13.686516 | 1 | 1.0818928 | 3 | 118.43637 | 25.12611 | 69.739998 | 0 | 21 | 3 |  | 0 | 0 | 0 | 0 | 0 | 0 |  | NA |  | NA | 2 | 2 | 2 | 0 | 0 | 0 |  | 1 | 24 | 0 | 7 | 0 |  |
| 2640 |  | 21.948 | 2.715 | 12.370148 | 87.629852 | 1 | 12.109514 | 1 | -0.92792499 | 3 | 98.017853 | 23.247778 | 69.278 | 0 | 0 | 3 | 1 |  | | | | | | 112.417159 | 1.136023467 | 93.6628657 | -0.965007868 | 2 | 2 |  | 0 | 0 | 1 | 1 | 2 |  | | | | |
| 2640 | 0 | 19.214001 | 2.1600001 | 11.241803 | 88.758194 | 1 | 13.478439 | 1 | -0.92792499 | 3 | 98.017853 | 25.232222 | 70.699997 | 0 | 19 | 3 | 1 | 0 | 0 | 0 | 0 | 0 | 0 |  | NA |  | NA | 2 | 2 | 2 | 0 | 0 | 1 |  | 2 | 24 | 0 | 7 | 0 |  |
| 2640 | 0 | 22.52 | 2.6400001 | 11.722914 | 88.277084 | 1 | 13.522245 | 1 | -0.92792499 | 3 | 98.017853 | 24.680002 | 73.970001 | 0 | 20 | 3 | 1 | 0 | 0 | 0 | 0 | 0 | 0 | 125.512091 | 2.322181796 | 101.9238526 | 0.303375558 | 2 | 2 | 2 | 0 | 0 | 1 | 0 | 2 | 23 | 0 |  | | |
| 2640 | 0 | 24.447001 | 2.95 | 12.066919 | 87.933083 | 1 | 13.544147 | 1 | -0.92792499 | 3 | 98.017853 | 24.072779 | 72.169998 | 0 | 21 | 3 | 1 | 0 | 0 | 0 | 0 | 0 | 0 |  | NA |  | NA | 2 | 2 | 2 | 0 | 0 | 1 |  | 2 | 24 | 0 | 7 | 0 |  |
| 2643 |  | 21.784 | 2.476 | 11.36614 | 88.633858 | 1 | 15.926078 | 0 | -3.3403184 | 2 | 107.64989 | 21.911112 | 70.723 | 0 | 0 | 3 | 0 |  | | | | | | 113.2395346 | 1.130967733 | 103.1044083 | 0.55077367 | 2 | 2 |  | 0 | 0 | 0 | 0 |  | | | | | |
| 2643 | 0 | 18.402 | 1.78 | 9.6728621 | 90.327141 | 1 | 17.295004 | 0 | -3.3403184 | 2 | 107.64989 | 25.806108 | 68.459999 | 0 | 19 | 3 | 0 | 0 | 0 | 0 | 0 | 0 | 0 |  | NA |  | NA | 2 | 2 | 2 | 0 | 0 | 0 |  | | 24 | 0 | 7 | 0 |  |
| 2643 | 0 | 20.441 | 2.1099999 | 10.322391 | 89.677612 | 1 | 17.336071 | 0 | -3.3403184 | 2 | 107.64989 | 25.247223 | 71.230003 | 0 | 20 | 3 | 0 | 0 | 0 | 0 | 0 | 0 | 0 | 112.5455758 | 1.064969586 | 102.8298554 | 0.490902616 | 2 | 2 | 2 | 0 | 0 | 0 | 0 |  | 25 | 0 |  | | |
| 2643 | 0 | 23.916 | 2.54 | 10.620505 | 89.379494 | 1 | 17.374401 | 0 | -3.3403184 | 2 | 107.64989 | 25.117777 | 69.730003 | 0 | 21 | 3 | 0 | 0 | 0 | 0 | 0 | 0 | 0 |  | NA |  | NA | 2 | 2 | 2 | 0 | 0 | 0 |  | | 25 | 0 | 7 | 0 |  |
| 2644 |  | 21.798 | 2.65 | 12.157079 | 87.842918 | 0 | 9.6344967 | 0 | -1.3817564 | 4 | 116.38989 | 19.879444 | 71.929 | 0 | 0 | 3 | 1 |  | | | | | | 123.9313168 | 2.080331365 | 100.190129 | 0.034571184 | 1 | 2 |  | 0 | 0 | 1 | 0 | 2 |  | | | | |
| 2644 | 1 | 17.677999 | 1.72 | 9.7296076 | 90.270393 | 0 | 11.022587 | 0 | -1.3817564 | 4 | 116.38989 | 25.717777 | 68.099998 | 0 | 19 | 3 | 1 | 1 | 0 | 1 | 0 | 1 |  | | NA |  | NA | 1 | 2 | 2 | 0 | 0 | 1 |  | 2 | 26 | 0 | 7 | 0 |  |
| 2644 | 0 | 20.141001 | 2.1199999 | 10.525792 | 89.474205 | 0 | 11.060917 | 0 | -1.3817564 | 4 | 116.38989 | 25.024445 | 71.519997 | 0 | 20 | 3 | 1 | 0 | 0 | 0 | 0 | 0 |  | 121.0162417 | 1.837638784 | 97.18631101 | -0.482641355 | 1 | 2 | 2 | 0 | 0 | 1 | 0 | 2 | 19 | 1 |  | | |
| 2644 | 0 | 23.525 | 2.5599999 | 10.88204 | 89.117958 | 0 | 11.099247 | 0 | -1.3817564 | 4 | 116.38989 | 24.941109 | 69.779999 | 0 | 21 | 3 | 1 | 0 | 0 | 0 | 0 | 0 |  | | NA |  | NA | 1 | 2 | 2 | 0 | 0 | 1 |  | 2 | 21 | 0 | 5.3000002 | 1 |  |
| 2645 |  | 21.872 | 2.716 | 12.417703 | 87.582298 | 1 | 14.94319 | 1 | 0.14494607 | 3 | 56.336159 | 21.986666 | 69.684 | 0 | 0 | 3 | 1 |  | | | | | | 69.46406188 | -2.817877109 | 84.98861343 | -2.024595939 | 2 | 2 |  | 0 | 0 | 0 | 1 | 1 |  | | | | |
| 2645 | 1 | 18.788 | 1.97 | 10.485416 | 89.514587 | 1 | 16.161533 | 1 | 0.14494607 | 3 | 56.336159 | 25.426666 | 70.139999 | 0 | 19 | 3 | 1 | 1 | 0 | 0 | 1 | 1 | 0 |  | NA |  | NA | 2 | 2 | 2 | 0 | 0 | 0 |  | 1 | 19 | 1 | 6.1999998 | 1 |  |
| 2645 | 1 | 21.465 | 2.45 | 11.41393 | 88.586067 | 1 | 16.199863 | 1 | 0.14494607 | 3 | 56.336159 | 25.323334 | 71.419998 | 0 | 20 | 3 | 1 | 1 |  | 0 | 0 | 1 | 0 | 93.20289725 | -0.621581519 | 69.2465345 | -3.545597647 | 2 | 2 | 2 | 0 | 0 | 0 | 1 | 1 | 17 | 1 |  | | |
| 2645 | 1 | 24.924999 | 2.8299999 | 11.354062 | 88.645935 | 1 | 16.238194 | 1 | 0.14494607 | 3 | 56.336159 | 23.886112 | 70.870003 | 0 | 21 | 3 | 1 | 1 | 0 | 0 | 0 | 1 | 0 |  | NA |  | NA | 2 | 2 | 2 | 0 | 0 | 0 |  | 1 | 17 | 1 | 6.4000001 | 1 |  |
| 2646 |  | 21.848 | 2.679 | 12.261992 | 87.738007 | 1 | 17.475702 | 0 | -0.33994275 | 2 | 96.135735 | 19.535 | 72.277 | 0 | 0 | 1 | 1 |  | | | | | | 116.5846711 | 1.402540729 | 88.71772611 | -1.622948017 | 2 | 2 |  | 0 | 0 | 0 | 1 | 2 |  | | | | |
| 2646 | 0 | 17.608 | 1.71 | 9.7114944 | 90.288506 | 1 | 18.757015 | 0 | -0.33994275 | 2 | 96.135735 | 25.698891 | 68.089996 | 0 | 19 | 1 | 1 | 0 | 0 | 0 | 0 | 0 | 0 |  | NA |  | NA | 2 | 2 | 2 | 0 | 0 | 0 |  | 2 | 24 | 0 | 7 | 0 |  |
| 2647 |  | 21.967 | 2.766 | 12.591615 | 87.408386 | 1 | 16.416153 | 1 | -1.2598199 | 2 | 123.18262 | 22.251112 | 72.588 | 0 | 0 | 3 | 1 |  | | | | | | 123.731958 | 2.153408029 | 107.4180731 | 1.172969473 | 2 | 2 |  | 0 | 0 | 0 | 0 | 4 |  | | | | |
| 2647 | 1 | 18.959 | 2.0899999 | 11.023787 | 88.976212 | 1 | 17.722109 | 1 | -1.2598199 | 2 | 123.18262 | 24.743889 | 74.120003 | 0 | 19 | 3 | 1 | 1 | 1 | 1 | 0 | 1 | 0 |  | NA |  | NA | 2 | 2 | 2 | 0 | 0 | 0 |  | 4 | 24 | 0 | 7 | 0 |  |
| 2647 | 0 | 21.291 | 2.47 | 11.601146 | 88.398857 | 1 | 17.760439 | 1 | -1.2598199 | 2 | 123.18262 | 24.44611 | 74.389999 | 0 | 20 | 3 | 1 | 0 | 0 | 0 | 0 | 0 | 0 |  | NA |  | NA | 2 | 2 | 2 | 0 | 0 | 0 |  | 4 | 24 | 0 |  | | |
| 2647 | 0 | 26.156 | 2.9200001 | 11.163787 | 88.836212 | 1 | 17.798767 | 1 | -1.2598199 | 2 | 123.18262 | 23.119444 | 74.559998 | 0 | 21 | 3 | 1 | 0 | 0 | 0 | 0 | 0 | 0 |  | NA |  | NA | 2 | 2 | 2 | 0 | 0 | 0 |  | 4 | 25 | 0 | 7 | 0 |  |
| 2648 |  | 21.904 | 2.731 | 12.468042 | 87.53196 | 1 | 16.117727 | 0 | -0.75965297 | 2 | 102.05428 | 21.566111 | 69.344 | 0 | 0 | 3 | 1 |  | | | | | | 118.5121463 | 1.573202685 | 92.12295665 | -1.200780385 | 2 | 2 |  | 0 | 0 | 0 | 0 | 2 |  | | | | |
| 2648 | 0 | 18.745001 | 1.9299999 | 10.296078 | 89.703918 | 1 | 17.442848 | 0 | -0.75965297 | 2 | 102.05428 | 25.610556 | 69.620003 | 0 | 19 | 3 | 1 | 0 | 0 | 0 | 0 | 0 | 0 |  | NA |  | NA | 2 | 2 | 2 | 0 | 0 | 0 |  | 2 | 24 | 0 | 7 | 0 |  |
| 2648 | 0 | 22.372999 | 2.5599999 | 11.442364 | 88.55764 | 1 | 17.481176 | 0 | -0.75965297 | 2 | 102.05428 | 25.31222 | 69.760002 | 0 | 20 | 3 | 1 | 0 | 0 | 0 | 0 | 0 | 0 | 104.0038967 | 0.341695301 | 90.97374045 | -1.334326049 | 2 | 2 | 2 | 0 | 0 | 0 | 0 | 2 | 24 | 0 |  | | |
| 2648 | 0 | 24.798 | 2.79 | 11.250907 | 88.749092 | 1 | 17.519506 | 0 | -0.75965297 | 2 | 102.05428 | 23.939445 | 69.919998 | 0 | 21 | 3 | 1 | 0 | 0 | 0 | 0 | 0 | 0 |  | NA |  | NA | 2 | 2 | 2 | 0 | 0 | 0 |  | 2 | 24 | 0 | 7 | 0 |  |
| 2649 |  | 22.029 | 2.816 | 12.78315 | 87.21685 | 1 | 17.87269 | 1 | -0.0941329 | 2 | 116.91798 | 22.310556 | 73.303 | 0 | 0 | 2 | 1 |  | | | | | | 122.8258859 | 2.068834452 | 101.6767957 | 0.249028307 | 2 | 2 |  | 1 |  | 1 | 0 |  | | | | | |
| 2649 | 0 | 18.753 | 2.0699999 | 11.038234 | 88.961769 | 1 | 19.151266 | 1 | -0.0941329 | 2 | 116.91798 | 24.529444 | 74.730003 | 0 | 19 | 2 | 1 | 0 | 0 | 0 | 0 | 0 | 0 |  | NA |  | NA | 2 | 2 | 2 | 1 |  | 1 |  | | 24 | 0 | 7 | 0 |  |
| 2649 | 1 | 21.226999 | 2.55 | 12.013003 | 87.987 | 1 | 19.189596 | 1 | -0.0941329 | 2 | 116.91798 | 24.436111 | 76.220001 | 0 | 20 | 2 | 1 | 1 | 0 | 1 | 1 | 1 | 1 | 112.2509658 | 1.111591546 | 100.9444256 | 0.137845899 | 2 | 2 | 2 | 1 |  | 1 | 0 |  | 25 | 0 |  | | |
| 2650 |  | 21.923 | 2.785 | 12.703553 | 87.296448 | 1 | 13.522245 | 0 | -3.0945086 | 2 | 95.445023 | 16.960556 | 75.439 | 0 | 0 | 3 | 1 |  | | | | | | 115.978694 | 1.382322509 | 87.81625024 | -1.781040051 | 2 | 2 |  | 0 | 0 | 0 | 0 | 2 |  | | | | |
| 2650 | 0 | 17.125 | 1.6900001 | 9.8686142 | 90.131386 | 1 | 14.899384 | 0 | -3.0945086 | 2 | 95.445023 | 25.408335 | 68.440002 | 0 | 19 | 3 | 1 | 0 | 0 | 0 | 0 | 0 | 0 |  | NA |  | NA | 2 | 2 | 2 | 0 | 0 | 0 |  | 2 | 24 | 0 | 7 | 0 |  |
| 2650 | 1 | 19.398001 | 2.05 | 10.568099 | 89.4319 | 1 | 14.934976 | 0 | -3.0945086 | 2 | 95.445023 | 24.360556 | 73.129997 | 0 | 20 | 3 | 1 | 1 | 1 | 1 | 1 | 1 | 0 | 104.0513764 | 0.35051617 | 87.75481606 | -1.77160128 | 2 | 2 | 2 | 0 | 0 | 0 | 0 | 2 | 24 | 0 |  | | |
| 2650 | 0 | 23.546 | 2.53 | 10.744925 | 89.255074 | 1 | 14.973306 | 0 | -3.0945086 | 2 | 95.445023 | 24.462221 | 71.160004 | 0 | 21 | 3 | 1 | 0 | 0 | 0 | 0 | 0 | 0 |  | NA |  | NA | 2 | 2 | 2 | 0 | 0 | 0 |  | 2 | 24 | 0 | 7 | 0 |  |
| 2651 |  | 21.921 | 2.773 | 12.64997 | 87.350029 | 1 | 13.103354 | 0 | -1.5139655 | 2 | 130.09468 | 17.331112 | 75.007 | 0 | 0 | 3 |  | | | | | | | 136.7501233 | 3.139541865 | 102.1211803 | 0.384506373 | 2 | 2 |  | 0 | 0 | 0 | 0 | 4 |  | | | | |
| 2651 | 1 | 17.594999 | 1.78 | 10.116511 | 89.883492 | 1 | 14.502396 | 0 | -1.5139655 | 2 | 130.09468 | 25.306667 | 68.93 | 0 | 19 | 3 |  | 1 | 1 | 1 | 1 | 1 | 1 |  | NA |  | NA | 2 | 2 | 2 | 0 | 0 | 0 |  | 4 | 20 | 0 | 5.4000001 | 1 |  |
| 2651 | 1 | 20.421 | 2.1400001 | 10.479409 | 89.520592 | 1 | 14.521561 | 0 | -1.5139655 | 2 | 130.09468 | 24.583889 | 73.260002 | 0 | 20 | 3 |  | 1 | 1 | 1 | 1 | 1 | 1 | 109.4103803 | 0.810469799 | 105.2331702 | 0.967498168 | 2 | 2 | 2 | 0 | 0 | 0 | 0 | 4 | 24 | 0 |  | | |
| 2651 | 0 | 23.518999 | 2.53 | 10.75726 | 89.242737 | 1 | 14.559891 | 0 | -1.5139655 | 2 | 130.09468 | 24.537222 | 71.010002 | 0 | 21 | 3 |  | 0 | 0 | 0 | 0 | 0 | 0 |  | NA |  | NA | 2 | 2 | 2 | 0 | 0 | 0 |  | 4 | 25 | 0 | 7 | 0 |  |
| 2653 |  | 21.806 | 2.673 | 12.258094 | 87.741905 | 1 | 15.148528 | 0 | 1.6736273 | 2 | 95.864746 | 20.625555 | 75.217 | 0 | 0 | 3 | 1 |  | | | | | | 120.615674 | 1.758369075 | 84.94651479 | -2.090693394 | 2 | 2 |  | 0 | 0 | 1 | 1 | 1 |  | | | | |
| 2653 | 1 | 16.378 | 1.97 | 12.028331 | 87.971672 | 1 | 16.550308 | 0 | 1.6736273 | 2 | 95.864746 | 24.568333 | 74.339996 | 0 | 19 | 3 | 1 | 1 | 1 | 1 | 1 | 1 | 1 |  | NA |  | NA | 2 | 2 | 2 | 0 | 0 | 1 |  | 1 | 13 | 1 | 4.4000001 | 1 |  |
| 2653 | 1 | 19.975 | 2.1700001 | 10.86358 | 89.136421 | 1 | 16.591375 | 0 | 1.6736273 | 2 | 95.864746 | 24.346109 | 76.639999 | 0 | 20 | 3 | 1 | 1 | 1 | 1 | 1 | 1 | 1 | 106.2867312 | 0.53715617 | 85.81434988 | -1.968944844 | 2 | 2 | 1 | 0 | 0 | 1 | 0 | 1 | 10 | 1 |  | | |
| 2653 | 1 | 21.319 | 2.3699999 | 11.116843 | 88.883156 | 1 | 16.624229 | 0 | 1.6736273 | 2 | 95.864746 | 23.522221 | 74.519997 | 0 | 21 | 3 | 1 | 1 | 1 | 1 | 1 | 1 | 1 |  | NA |  | NA | 2 | 2 | 1 | 0 | 0 | 1 |  | 1 | 23 | 0 | 6.6999998 | 1 |  |
| 2654 |  | 23.207 | 2.992 | 12.892662 | 87.107338 | 1 | 14.299794 | 0 | -0.11307801 | 3 | 95.751808 | 20.326666 | 74.281 | 0 | 0 | 3 | 1 |  | | | | | | 116.4079956 | 1.411512665 | 88.18648271 | -1.728026247 | 2 | 2 |  | 0 | 0 | 1 | 1 | 2 |  | | | | |
| 2654 | 0 | 17.075001 | 1.91 | 11.185944 | 88.814056 | 1 | 15.622176 | 0 | -0.11307801 | 3 | 95.751808 | 25.287222 | 71.050003 | 0 | 19 | 3 | 1 | 0 | 0 | 0 | 0 | 0 | 0 |  | NA |  | NA | 2 | 2 | 2 | 0 | 0 | 1 |  | 2 | 24 | 0 | 7 | 0 |  |
| 2654 | 1 | 20.851 | 2.21 | 10.599012 | 89.400986 | 1 | 15.671458 | 0 | -0.11307801 | 3 | 95.751808 | 24.871664 | 74.300003 | 0 | 20 | 3 | 1 | 1 | 0 | 0 | 0 | 1 | 0 | 116.2110583 | 1.383317465 | 94.2644203 | -0.904292072 | 2 | 2 | 1 | 0 | 0 | 1 | 0 | 2 | 18 | 1 |  | | |
| 2654 | 1 | 22.474001 | 2.47 | 10.990478 | 89.009521 | 1 | 15.701574 | 0 | -0.11307801 | 3 | 95.751808 | 24.14889 | 72.330002 | 0 | 21 | 3 | 1 | 1 | 0 | 1 | 1 | 0 | 0 |  | NA |  | NA | 2 | 2 | 1 | 0 | 0 | 1 |  | 2 | 23 | 0 | 7 | 0 |  |
| 2656 |  | 22.279 | 2.766 | 12.415279 | 87.584724 | 0 | 11.49076 | 1 | 0.83030277 | 3 | 104.51991 | 21.052778 | 72.075 | 0 | 0 | 3 | 1 |  | | | | | | 109.9196391 | 0.906786493 | 101.7576627 | 0.289874779 | 2 | 2 |  | 0 | 0 | 0 | 0 | 3 |  | | | | |
| 2656 | 1 | 17.882 | 1.92 | 10.737054 | 89.262947 | 1 | 12.873374 | 1 | 0.83030277 | 3 | 104.51991 | 25.277779 | 71.07 | 0 | 19 | 3 | 1 | 1 | 1 | 1 | 1 | 1 | 1 |  | NA |  | NA | 2 | 2 | 2 | 0 | 0 | 0 |  | 3 | 20 | 0 | 5.6999998 | 1 |  |
| 2656 | 1 | 20.545 | 2.22 | 10.805549 | 89.19445 | 1 | 12.914442 | 1 | 0.83030277 | 3 | 104.51991 | 25.326111 | 73.220001 | 0 | 20 | 3 | 1 | 1 | 0 | 0 | 1 | 1 | 1 | 103.9045685 | 0.357886857 | 108.6099732 | 1.469407445 | 2 | 2 | 2 | 0 | 0 | 0 | 0 | 3 | 21 | 0 |  | | |
| 2656 | 1 | 23.15 | 2.54 | 10.971922 | 89.028076 | 1 | 12.952772 | 1 | 0.83030277 | 3 | 104.51991 | 24.117224 | 71.419998 | 0 | 21 | 3 | 1 | 1 | 1 | 0 | 0 | 1 | 0 |  | NA |  | NA | 2 | 2 | 2 | 0 | 0 | 0 |  | 3 | 17 | 1 | 7 | 0 |  |
| 2658 |  | 23.526 | 3.008 | 12.785854 | 87.214149 | 1 | 12.424367 | 0 | 1.2107996 | 2 | 102.54073 | 22.688889 | 69 | 0 | 0 | 2 | 1 |  | | | | | | 111.1995888 | 0.980024658 | 98.74367491 | -0.218184646 | 2 | 2 |  | 0 | 0 | 1 | 0 | 4 |  | | | | |
| 2658 | 0 | 19.634001 | 2.1400001 | 10.89946 | 89.10054 | 1 | 13.752225 | 0 | 1.2107996 | 2 | 102.54073 | 24.516109 | 77.800003 | 0 | 20 | 2 | 1 | 0 | 0 | 0 | 0 | 0 | 0 | 122.3385276 | 1.916785403 | 96.78199831 | -0.534480566 | 2 | 2 | 2 | 0 | 0 | 1 | 0 | 4 | 25 | 0 |  | | |
| 2658 | 0 | 20.267 | 2.1500001 | 10.608378 | 89.391624 | 1 | 13.79603 | 0 | 1.2107996 | 2 | 102.54073 | 24.047779 | 74.419998 | 0 | 21 | 2 | 1 | 0 | 0 | 0 | 0 | 0 | 0 |  | NA |  | NA | 2 | 2 | 2 | 0 | 0 | 1 |  | 4 | 25 | 0 | 7 | 0 |  |
| 2660 |  | 22.449 | 2.811 | 12.521716 | 87.478287 | 1 | 13.111567 | 0 | 1.3913015 | 3 | 108.75088 | 20.923334 | 71.136 | 0 | 0 | 3 | 1 |  | | | | | | 126.2710911 | 2.261348751 | 94.32614673 | -0.916341199 | 2 | 2 |  | 1 | 0 | 0 | 0 | 1 |  | | | | |
| 2660 | 0 | 18.659 | 1.89 | 10.12916 | 89.870842 | 1 | 14.507872 | 0 | 1.3913015 | 3 | 108.75088 | 25.455 | 70.580002 | 0 | 19 | 3 | 1 | 0 | 0 | 0 | 0 | 0 | 0 |  | NA |  | NA | 2 | 2 | 2 | 1 | 0 | 0 |  | 1 | 24 | 0 | 7 | 0 |  |
| 2660 | 0 | 20.292999 | 2.1600001 | 10.644066 | 89.355934 | 1 | 14.546202 | 0 | 1.3913015 | 3 | 108.75088 | 25.707224 | 71.839996 | 0 | 20 | 3 | 1 | 0 | 0 | 0 | 0 | 0 | 0 | 114.1747946 | 1.219163784 | 99.67037697 | -0.05668294 | 2 | 2 | 2 | 1 | 0 | 0 | 0 | 1 | 24 | 0 |  | | |
| 2660 | 0 | 23.164 | 2.52 | 10.87895 | 89.121048 | 1 | 14.568104 | 0 | 1.3913015 | 3 | 108.75088 | 24.396667 | 70.309998 | 0 | 21 | 3 | 1 | 0 | 0 | 0 | 0 | 0 | 0 |  | NA |  | NA | 2 | 2 | 2 | 1 | 0 | 0 |  | 1 | 24 | 0 | 7 | 0 |  |
| 2661 |  | 28.281 | 3.716 | 13.139564 | 86.860435 | 0 | 11.611225 | 1 | 1.7179354 | 2 | 122.99992 | 21.557777 | 72.114 | 0 | 0 | 2 | 0 |  | | | | | | 124.1422099 | 2.200433685 | 106.0381507 | 1.034020206 | 2 | 2 |  | 0 | 0 | 1 | 0 | 3 |  | | | | |
| 2661 | 0 | 20.805 | 2.27 | 10.910839 | 89.089165 | 1 | 12.673512 | 1 | 1.7179354 | 2 | 122.99992 | 25.246666 | 72.300003 | 0 | 20 | 2 | 0 | 0 | 0 | 0 | 0 | 0 | 0 | 84.70821262 | -1.408908352 | 108.5000873 | 1.449158558 | 2 | 2 | 2 | 0 | 0 | 1 | 1 | 3 | 13 | 1 |  | | |
| 2661 | 0 | 23.261 | 2.54 | 10.919565 | 89.080437 | 1 | 12.706366 | 1 | 1.7179354 | 2 | 122.99992 | 24.140556 | 71.019997 | 0 | 21 | 2 | 0 | 0 | 0 | 0 | 0 | 0 | 0 |  | NA |  | NA | 2 | 2 | 2 | 0 | 0 | 1 |  | 3 | 25 | 0 | 7 | 0 |  |
| 2662 |  | 21.726 | 2.669 | 12.28482 | 87.715179 | 1 | 15.819302 | 0 | -0.56690949 | 2 | 133.15152 | 20.459444 | 70.237 | 0 | 0 | 3 | 1 |  | | | | | | 137.2412727 | 3.125988502 | 103.9469169 | 0.709277323 | 2 | 2 |  | 0 | 0 | 0 | 0 | 4 |  | | | | |
| 2662 | 0 | 17.527 | 1.64 | 9.3569918 | 90.643005 | 1 | 17.193703 | 0 | -0.56690949 | 2 | 133.15152 | 26.012222 | 67.18 | 0 | 19 | 3 | 1 | 0 | 0 | 0 | 0 | 0 | 0 |  | NA |  | NA | 2 | 2 | 2 | 0 | 0 | 0 |  | 4 | 24 | 0 | 7 | 0 |  |
| 2662 | 0 | 20.049 | 2.1400001 | 10.673849 | 89.326149 | 1 | 17.232033 | 0 | -0.56690949 | 2 | 133.15152 | 25.215555 | 70.480003 | 0 | 20 | 3 | 1 | 0 | 0 | 0 | 0 | 0 | 0 | 125.1230209 | 2.112243084 | 100.5244242 | 0.088334666 | 2 | 2 | 2 | 0 | 0 | 0 | 0 | 4 | 24 | 0 |  | | |
| 2662 | 0 | 22.966 | 2.55 | 11.103371 | 88.896629 | 1 | 17.2731 | 0 | -0.56690949 | 2 | 133.15152 | 25.334442 | 68.699997 | 0 | 21 | 3 | 1 | 0 | 0 | 0 | 0 | 0 | 0 |  | NA |  | NA | 2 | 2 | 2 | 0 | 0 | 0 |  | 4 | 24 | 0 | 7 | 0 |  |
| 2668 |  | 22.278 | 2.756 | 12.370949 | 87.629051 | 1 | 17.141684 | 1 | -0.46375474 | 2 | 111.69784 | 21.107779 | 73.237 | 0 | 0 | 3 | 1 |  | | | | | | 111.5868621 | 1.053581703 | 106.8649431 | 1.069798019 | 2 | 2 |  | 0 | 0 | 1 | 0 | 4 |  | | | | |
| 2668 | 0 | 16.790001 | 1.91 | 11.375818 | 88.624184 | 1 | 18.439425 | 1 | -0.46375474 | 2 | 111.69784 | 25.229446 | 71.220001 | 0 | 19 | 3 | 1 | 0 | 0 | 0 | 0 | 0 | 0 |  | NA |  | NA | 2 | 2 | 2 | 0 | 0 | 1 |  | 4 | 24 | 0 | 7 | 0 |  |
| 2668 | 1 | 21.132 | 2.21 | 10.458073 | 89.541924 | 1 | 18.483231 | 1 | -0.46375474 | 2 | 111.69784 | 24.65889 | 74.879997 | 0 | 20 | 3 | 1 | 1 | 0 | 1 | 0 | 0 | 0 | 111.5544046 | 1.049495221 | 106.9039097 | 1.061467526 | 2 | 2 | 2 | 0 | 0 | 1 | 0 | 4 | 25 | 0 |  | | |
| 2668 | 1 | 21.763 | 2.3900001 | 10.981942 | 89.018059 | 1 | 18.543463 | 1 | -0.46375474 | 2 | 111.69784 | 24.284998 | 73 | 0 | 21 | 3 | 1 | 1 | 0 | 1 | 1 | 0 | 0 |  | NA |  | NA | 2 | 2 | 2 | 0 | 0 | 1 |  | 4 | 25 | 0 | 7 | 0 |  |
| 2669 |  | 22.314 | 2.773 | 12.427176 | 87.572823 | 1 | 15.216974 | 0 | 0.88780493 | 2 | 106.82586 | 21.102777 | 72.733 | 0 | 0 | 2 | 0 |  | | | | | | 114.0445533 | 1.203635066 | 100.8013493 | 0.138999452 | 2 | 2 |  | 0 | 0 | 0 | 0 | 3 |  | | | | |
| 2669 | 0 | 20.834 | 2.21 | 10.60766 | 89.392342 | 1 | 16.637918 | 0 | 0.88780493 | 2 | 106.82586 | 24.926664 | 74.32 | 0 | 20 | 2 | 0 | 0 | 0 | 0 | 0 | 0 | 0 | 95.197037 | -0.415088324 | 104.8006821 | 0.862133068 | 2 | 2 | 2 | 0 | 0 | 0 | 0 | 3 | 24 | 0 |  | | |
| 2669 | 0 | 21.708 | 2.3699999 | 10.917634 | 89.082367 | 1 | 16.668036 | 0 | 0.88780493 | 2 | 106.82586 | 24.444445 | 72.239998 | 0 | 21 | 2 | 0 | 0 | 0 | 0 | 0 | 0 | 0 |  | NA |  | NA | 2 | 2 | 2 | 0 | 0 | 0 |  | 3 | 24 | 0 | 7 | 0 |  |
| 2670 |  | 22.401 | 2.779 | 12.405696 | 87.594307 | 0 | 9.5906916 | 1 | 1.5928032 | 4 | 114.28291 | 21.477222 | 72.227 | 0 | 0 | 3 | 1 |  | | | | | | 134.704973 | 3.093703671 | 90.64749779 | -1.369715784 | 2 | 2 |  | 0 | 0 | 0 | 0 | 2 |  | | | | |
| 2670 | 0 | 17.632 | 1.92 | 10.889292 | 89.11071 | 0 | 10.956879 | 1 | 1.5928032 | 4 | 114.28291 | 25.362778 | 70.599998 | 0 | 19 | 3 | 1 | 0 | 0 | 0 | 0 | 0 |  | | NA |  | NA | 2 | 2 | 2 | 0 | 0 | 0 |  | 2 | 27 | 0 | 7 | 0 |  |
| 2670 | 0 | 21.364 | 2.25 | 10.531735 | 89.468262 | 0 | 10.992471 | 1 | 1.5928032 | 4 | 114.28291 | 24.839443 | 74.260002 | 0 | 20 | 3 | 1 | 0 | 0 | 0 | 0 | 0 |  | 102.6989335 | 0.246545661 | 81.66961342 | -2.492501291 | 2 | 2 | 2 | 0 | 0 | 0 | 0 | 2 | 26 | 0 |  | | |
| 2670 | 0 | 22.174999 | 2.4200001 | 10.913192 | 89.086807 | 0 | 11.04449 | 1 | 1.5928032 | 4 | 114.28291 | 24.576666 | 72.190002 | 0 | 21 | 3 | 1 | 0 | 0 | 0 | 0 | 0 |  | | NA |  | NA | 2 | 2 | 2 | 0 | 0 | 0 |  | 2 | 26 | 0 | 7 | 0 |  |
| 2672 |  | 22.178 | 2.78 | 12.534945 | 87.465057 | 0 | 10.997947 | 1 | 1.7815342 | 3 | 112.82021 | 20.038889 | 72.058 | 0 | 0 | 3 |  | | | | | | | 126.9282237 | 2.44560711 | 95.13849186 | -0.754673686 | 2 | 2 |  | 0 | 0 | 1 | 0 | 3 |  | | | | |
| 2672 | 0 | 17.164 | 1.79 | 10.428804 | 89.571198 | 1 | 12.323067 | 1 | 1.7815342 | 3 | 112.82021 | 26.090553 | 67.550003 | 0 | 19 | 3 |  | 0 | 0 | 0 | 0 | 0 | 0 |  | NA |  | NA | 2 | 2 | 2 | 0 | 0 | 1 |  | 3 | 24 | 0 | 7 | 0 |  |
| 2672 | 0 | 20.174999 | 2.1900001 | 10.85502 | 89.144981 | 1 | 12.372348 | 1 | 1.7815342 | 3 | 112.82021 | 25.852779 | 69.989998 | 0 | 20 | 3 |  | 0 | 0 | 0 | 0 | 0 | 0 | 95.8535719 | -0.381018343 | 90.61970452 | -1.385181769 | 2 | 2 | 2 | 0 | 0 | 1 | 1 | 3 | 25 | 0 |  | | |
| 2672 | 0 | 22.903999 | 2.51 | 10.958785 | 89.041214 | 1 | 12.399726 | 1 | 1.7815342 | 3 | 112.82021 | 24.728889 | 68.260002 | 0 | 21 | 3 |  | 0 | 0 | 0 | 0 | 0 | 0 |  | NA |  | NA | 2 | 2 | 2 | 0 | 0 | 1 |  | 3 | 24 | 0 | 7 | 0 |  |
| 2674 |  | 22.166 | 2.747 | 12.392854 | 87.607147 | 0 | 10.414784 | 1 | 1.7815342 | 2 | 112.95612 | 22.264444 | 69.307 | 0 | 0 | 3 | 0 |  | | | | | | 122.2414451 | 2.011728601 | 98.80866741 | -0.191248987 | 2 | 2 |  | 1 | 0 | 0 | 0 | 3 |  | | | | |
| 2674 | 0 | 17.384001 | 1.79 | 10.296824 | 89.703178 | 0 | 11.805613 | 1 | 1.7815342 | 2 | 112.95612 | 25.077778 | 72.339996 | 0 | 19 | 3 | 0 | 0 | 0 | 0 | 0 | 0 |  | | NA |  | NA | 2 | 2 | 2 | 1 | 0 | 0 |  | 3 | 27 | 0 | 7 | 0 |  |
| 2674 | 0 | 19.982 | 2.1600001 | 10.80973 | 89.190269 | 0 | 11.824778 | 1 | 1.7815342 | 2 | 112.95612 | 25.079998 | 75.459999 | 0 | 20 | 3 | 0 | 0 | 0 | 0 | 0 | 0 |  | 102.8118316 | 0.257847027 | 101.6729894 | 0.274062262 | 2 | 2 | 2 | 1 | 0 | 0 | 0 | 3 | 27 | 0 |  | | |
| 2674 | 0 | 21.304001 | 2.28 | 10.702215 | 89.297783 | 0 | 11.882273 | 1 | 1.7815342 | 2 | 112.95612 | 24.441113 | 72.559998 | 0 | 21 | 3 | 0 | 0 | 0 | 0 | 0 | 0 |  | | NA |  | NA | 2 | 2 | 2 | 1 | 0 | 0 |  | 3 | 27 | 0 | 7 | 0 |  |
| 2675 |  | 21.495 | 2.561 | 11.914399 | 88.085602 | 1 | 16.084873 | 1 | 1.4721255 | 2 | 100.61581 | 24.563889 | 66.421 | 0 | 0 | 3 | 1 |  | | | | | | 98.20670962 | -0.16379658 | 109.6864892 | 1.567155513 | 2 | 2 |  | 0 | 0 | 0 | 0 | 3 |  | | | | |
| 2675 | 0 | 15.062 | 1.6 | 10.622759 | 89.377243 | 1 | 17.437372 | 1 | 1.4721255 | 2 | 100.61581 | 24.393887 | 75.339996 | 0 | 19 | 3 | 1 | 0 | 0 | 0 | 0 | 0 | 0 |  | NA |  | NA | 2 | 2 | 2 | 0 | 0 | 0 |  | 3 | 24 | 0 | 7 | 0 |  |
| 2675 | 1 | 18.35 | 2 | 10.899182 | 89.100815 | 1 | 17.467487 | 1 | 1.4721255 | 2 | 100.61581 | 23.772778 | 80.029999 | 0 | 20 | 3 | 1 | 1 | 1 | 0 | 0 | 1 | 1 | 105.0024251 | 0.455383392 | 105.5490658 | 0.850412499 | 2 | 2 | 2 | 0 | 0 | 0 | 0 | 3 | 24 | 0 |  | | |
| 2675 | 0 | 19.368999 | 2.03 | 10.480665 | 89.519333 | 1 | 17.519506 | 1 | 1.4721255 | 2 | 100.61581 | 23.612221 | 76.360001 | 0 | 21 | 3 | 1 | 0 | 0 | 0 | 0 | 0 | 0 |  | NA |  | NA | 2 | 2 | 2 | 0 | 0 | 0 |  | 3 | 25 | 0 | 7 | 0 |  |
| 2676 |  | 22.133 | 2.786 | 12.587539 | 87.41246 | 1 | 15.885011 | 1 | -1.5018775 | 2 | 107.94865 | 19.419445 | 72.565 | 0 | 0 | 3 | 1 |  | | | | | | 118.8816455 | 1.716401479 | 99.54887363 | -0.067625331 |  | | | | | 0 | 0 | 3 |  | | | | |
| 2676 | 1 | 17.431999 | 1.89 | 10.84213 | 89.157867 | 1 | 17.267626 | 1 | -1.5018775 | 2 | 107.94865 | 26.412777 | 67.010002 | 0 | 19 | 3 | 1 | 1 | 0 | 1 | 1 | 1 | 1 |  | NA |  | NA |  | | 2 |  | | 0 |  | 3 | 24 | 0 | 7 | 0 |  |
| 2676 | 0 | 20.577999 | 2.27 | 11.031199 | 88.968803 | 1 | 17.305954 | 1 | -1.5018775 | 2 | 107.94865 | 25.936665 | 67.809998 | 0 | 20 | 3 | 1 | 0 | 0 | 0 | 0 | 0 | 0 | 95.9774885 | -0.366991658 | 103.6012781 | 0.542950838 |  | | 2 |  | | 0 | 0 | 3 | 25 | 0 |  | | |
| 2676 | 1 | 23.773001 | 2.5899999 | 10.894711 | 89.105286 | 1 | 17.344284 | 1 | -1.5018775 | 2 | 107.94865 | 24.80389 | 67.410004 | 0 | 21 | 3 | 1 | 1 | 0 | 1 | 1 | 1 | 1 |  | NA |  | NA |  | | 2 |  | | 0 |  | 3 | 23 | 0 | 7 | 0 |  |
| 2678 |  | 21.626 | 2.598 | 12.013317 | 87.986679 | 1 | 17.117043 | 1 | -1.2293358 | 3 | 101.06891 | 24.098888 | 67.023 | 0 | 0 | 3 | 1 |  | | | | | | 109.19442 | 0.836536971 | 99.00486414 | -0.1459248 | 2 | 2 |  | 0 | 0 | 0 | 0 | 4 |  | | | | |
| 2678 | 0 | 16.069 | 1.72 | 10.703839 | 89.296158 | 1 | 18.422998 | 1 | -1.2293358 | 3 | 101.06891 | 24.477221 | 75.760002 | 0 | 19 | 3 | 1 | 0 | 0 | 0 | 0 | 0 | 0 |  | NA |  | NA | 2 | 2 | 2 | 0 | 0 | 0 |  | 4 | 24 | 0 | 7 | 0 |  |
| 2678 | 0 | 19.936001 | 2.1099999 | 10.583867 | 89.41613 | 1 | 18.461329 | 1 | -1.2293358 | 3 | 101.06891 | 24.190001 | 76.129997 | 0 | 20 | 3 | 1 | 0 | 0 | 0 | 0 | 0 | 0 | 100.0352826 | 0.003213624 | 101.2777725 | 0.187966889 | 2 | 2 | 2 | 0 | 0 | 0 | 0 | 4 | 24 | 0 |  | | |
| 2678 | 0 | 18.801001 | 1.98 | 10.531354 | 89.468643 | 1 | 18.499659 | 1 | -1.2293358 | 3 | 101.06891 | 23.094444 | 75.029999 | 0 | 21 | 3 | 1 | 0 | 0 | 0 | 0 | 0 | 0 |  | NA |  | NA | 2 | 2 | 2 | 0 | 0 | 0 |  | 4 | 25 | 0 | 7 | 0 |  |
| 2681 |  | 21.555 | 2.578 | 11.960102 | 88.039902 | 1 | 17.946611 | 0 | -0.40354156 | 4 | 117.70744 | 24.343889 | 66.699 | 0 | 0 | 3 | 0 |  | | | | | | 139.4292226 | 3.272021001 | 90.30660041 | -1.418214818 | 2 | 2 |  | 0 | 0 | 0 | 0 | 2 |  | | | | |
| 2681 | 0 | 15.254 | 1.61 | 10.554608 | 89.445389 | 1 | 19.271732 | 0 | -0.40354156 | 4 | 117.70744 | 24.471109 | 74.989998 | 0 | 19 | 3 | 0 | 0 | 0 | 0 | 0 | 0 | 0 |  | NA |  | NA | 2 | 2 | 2 | 0 | 0 | 0 |  | 2 | 24 | 0 | 7 | 0 |  |
| 2681 | 1 | 18.448 | 2 | 10.841284 | 89.158714 | 1 | 19.304586 | 0 | -0.40354156 | 4 | 117.70744 | 23.938887 | 79.290001 | 0 | 20 | 3 | 0 | 1 | 1 | 1 | 1 | 0 | 0 | 131.9717654 | 2.653174894 | 93.74718423 | -0.943221283 | 2 | 2 | 2 | 0 | 0 | 0 | 0 | 2 | 25 | 0 |  | | |
| 2681 | 0 | 19.625 | 2.0599999 | 10.496815 | 89.503181 | 1 | 19.345654 | 0 | -0.40354156 | 4 | 117.70744 | 23.631668 | 75.980003 | 0 | 21 | 3 | 0 | 0 | 0 | 0 | 0 | 0 | 0 |  | NA |  | NA | 2 | 2 | 2 | 0 | 0 | 0 |  | 2 | 25 | 0 | 7 | 0 |  |
| 2682 |  | 22.101 | 2.73 | 12.352383 | 87.647621 | 1 | 13.073237 | 1 | -1.1548917 | 2 | 96.491951 | 22.442778 | 69.067 | 0 | 0 | 2 | 0 |  | | | | | | 116.4246849 | 1.500993152 | 88.71801143 | -1.622297482 | 2 | 2 |  | 0 | 0 | 0 | 0 | 1 |  | | | | |
| 2682 | 0 | 20.43 | 2.1800001 | 10.670583 | 89.329414 | 1 | 14.475018 | 1 | -1.1548917 | 2 | 96.491951 | 24.818331 | 73.360001 | 0 | 20 | 2 | 0 | 0 | 0 | 0 | 0 | 0 | 0 | 114.3879829 | 1.31187196 | 86.69845514 | -1.828736029 | 2 | 2 | 2 | 0 | 0 | 0 | 0 | 1 | 24 | 0 |  | | |
| 2682 | 0 | 21.884001 | 2.3599999 | 10.784134 | 89.215866 | 1 | 14.513347 | 1 | -1.1548917 | 2 | 96.491951 | 23.648335 | 72.669998 | 0 | 21 | 2 | 0 | 0 | 0 | 0 | 0 | 0 | 0 |  | NA |  | NA | 2 | 2 | 2 | 0 | 0 | 0 |  | 1 | 24 | 0 | 7 | 0 |  |
| 2684 |  | 22.148 | 2.742 | 12.38035 | 87.619652 | 0 | 11.586584 | 0 | -0.80598843 | 4 | 124.6219 | 22.347778 | 69.22 | 0 | 0 | 2 | 1 |  | | | | | | 144.2876128 | 3.800228235 | 92.50056766 | -1.191056618 | 2 | 2 |  | 1 | 0 | 0 | 0 | 3 |  | | | | |
| 2684 | 1 | 17.101999 | 1.74 | 10.17425 | 89.825752 | 1 | 12.947296 | 0 | -0.80598843 | 4 | 124.6219 | 25.239445 | 71.690002 | 0 | 19 | 2 | 1 | 1 | 1 | 1 | 1 | 1 | 1 |  | NA |  | NA | 2 | 2 | 2 | 1 | 0 | 0 |  | 3 | 25 | 0 | 7 | 0 |  |
| 2684 | 0 | 21.131001 | 2.26 | 10.695187 | 89.30481 | 1 | 13.021218 | 0 | -0.80598843 | 4 | 124.6219 | 24.452223 | 72.589996 | 0 | 21 | 2 | 1 | 0 | 0 | 0 | 0 | 0 | 0 |  | NA |  | NA | 2 | 2 | 2 | 1 | 0 | 0 |  | 3 | 25 | 0 | 7 | 0 |  |
| 2685 |  | 22.111 | 2.742 | 12.401068 | 87.59893 | 1 | 15.986311 | 0 | 1.1566851 | 2 | 126.25621 | 22.094999 | 69.449 | 0 | 0 | 3 | 1 |  | | | | | | 131.2509205 | 2.632151669 | 101.8132691 | 0.315895433 | 2 | 2 |  | 1 | 0 | 0 | 0 | 4 |  | | | | |
| 2685 | 0 | 16.754999 | 1.72 | 10.265593 | 89.734406 | 1 | 17.341547 | 0 | 1.1566851 | 2 | 126.25621 | 25.265554 | 71.489998 | 0 | 19 | 3 | 1 | 0 | 0 | 0 | 0 | 0 | 0 |  | NA |  | NA | 2 | 2 | 2 | 1 | 0 | 0 |  | 4 | 24 | 0 | 7 | 0 |  |
| 2685 | 0 | 19.079 | 2.03 | 10.639971 | 89.360031 | 1 | 17.382614 | 0 | 1.1566851 | 2 | 126.25621 | 24.830555 | 74.68 | 0 | 20 | 3 | 1 | 0 | 0 | 0 | 0 | 0 | 0 | 121.309593 | 1.79313378 | 105.1823423 | 0.921260341 | 2 | 2 | 2 | 1 | 0 | 0 | 0 | 4 | 25 | 0 |  | | |
| 2685 | 0 | 21.289 | 2.29 | 10.756729 | 89.243271 | 1 | 17.423683 | 0 | 1.1566851 | 2 | 126.25621 | 24.308332 | 72.480003 | 0 | 21 | 3 | 1 | 0 | 0 | 0 | 0 | 0 | 0 |  | NA |  | NA | 2 | 2 | 2 | 1 | 0 | 0 |  | 4 | 24 | 0 | 7 | 0 |  |
| 2686 |  | 22.089 | 2.797 | 12.662411 | 87.337585 | 1 | 16.785763 | 0 | 1.5357243 | 2 | 78.332001 | 18.178888 | 73.829 | 0 | 0 | 3 | 1 |  | | | | | | 81.35116968 | -1.63271055 | 105.54937 | 1.005308094 | 2 | 2 |  | 0 | 0 | 1 | 0 | 3 |  | | | | |
| 2686 | 1 | 17.191 | 1.87 | 10.877785 | 89.122215 | 1 | 18.179329 | 0 | 1.5357243 | 2 | 78.332001 | 26.94722 | 64.709999 | 0 | 19 | 3 | 1 | 1 | 1 | 1 | 0 | 1 | 1 |  | NA |  | NA | 2 | 2 | 2 | 0 | 0 | 1 |  | 3 | 24 | 0 | 7 | 0 |  |
| 2686 | 1 | 20.243999 | 2.26 | 11.163802 | 88.836197 | 1 | 18.217659 | 0 | 1.5357243 | 2 | 78.332001 | 26.359999 | 65.790001 | 0 | 20 | 3 | 1 | 1 | 0 | 0 | 0 | 1 | 0 | 76.76572707 | -2.026275811 | 109.4831311 | 1.751465806 | 2 | 2 | 2 | 0 | 0 | 1 | 0 | 3 | 24 | 0 |  | | |
| 2686 | 1 | 23.834999 | 2.5999999 | 10.908328 | 89.091675 | 1 | 18.255989 | 0 | 1.5357243 | 2 | 78.332001 | 25.221668 | 65.459999 | 0 | 21 | 3 | 1 | 1 | 0 | 1 | 1 | 1 | 0 |  | NA |  | NA | 2 | 2 | 2 | 0 | 0 | 1 |  | 3 | 21 | 0 | 6.8000002 | 1 |  |
| 2687 |  | 22.48 | 2.856 | 12.704626 | 87.295372 | 1 | 12.312115 | 0 | -1.5018775 | 2 | 116.37032 | 21.040001 | 70.576 | 0 | 0 | 3 | 1 |  | | | | | | 119.6721536 | 1.711098222 | 104.0306004 | 0.759004663 |  | | | | | 0 | 0 | 2 |  | | | | |
| 2687 | 0 | 17.355 | 1.73 | 9.9683084 | 90.031693 | 1 | 13.587954 | 0 | -1.5018775 | 2 | 116.37032 | 25.473886 | 70.720001 | 0 | 19 | 3 | 1 | 0 | 0 | 0 | 0 | 0 | 0 |  | NA |  | NA |  | | 2 |  | | 0 |  | 2 | 24 | 0 | 7 | 0 |  |
| 2687 | 1 | 19.298 | 2.03 | 10.519225 | 89.480774 | 1 | 13.634497 | 0 | -1.5018775 | 2 | 116.37032 | 25.177221 | 73.339996 | 0 | 20 | 3 | 1 | 1 | 1 | 0 | 1 | 1 | 1 | 102.7211447 | 0.236913964 | 102.4062743 | 0.432205793 |  | | 2 |  | | 0 | 0 | 2 | 24 | 0 |  | | |
| 2687 | 0 | 22.177999 | 2.4000001 | 10.821536 | 89.178467 | 1 | 13.670089 | 0 | -1.5018775 | 2 | 116.37032 | 24.345554 | 71.449997 | 0 | 21 | 3 | 1 | 0 | 0 | 0 | 0 | 0 | 0 |  | NA |  | NA |  | | 2 |  | | 0 |  | 2 | 24 | 0 | 7 | 0 |  |
| 2691 |  | 22.192 | 2.762 | 12.445927 | 87.554077 | 1 | 17.23477 | 0 | -0.052803483 | 3 | 115.24196 | 22.282223 | 69.292 | 0 | 0 | 3 | 1 |  | | | | | | 126.95054 | 2.263622832 | 96.92593226 | -0.494352021 | 2 | 2 |  | 1 |  | 0 | 0 | 4 |  | | | | |
| 2691 | 1 | 16.923 | 1.8 | 10.636412 | 89.363586 | 1 | 18.622862 | 0 | -0.052803483 | 3 | 115.24196 | 25.112225 | 72.669998 | 0 | 19 | 3 | 1 | 1 | 1 | 1 | 1 | 1 | 1 |  | NA |  | NA | 2 | 2 | 1 | 1 |  | 0 |  | 4 | 22 | 0 | 5.0999999 | 1 |  |
| 2691 | 1 | 20.332001 | 2.1900001 | 10.771198 | 89.228806 | 1 | 18.66119 | 0 | -0.052803483 | 3 | 115.24196 | 24.879442 | 73.440002 | 0 | 20 | 3 | 1 | 1 | 1 | 1 | 1 | 0 | 1 | 125.0938417 | 2.098183035 | 95.8265299 | -0.651197774 | 2 | 2 | 1 | 1 |  | 0 | 0 | 4 | 25 | 0 |  | | |
| 2691 | 1 | 21.607 | 2.3199999 | 10.737261 | 89.262741 | 1 | 18.69952 | 0 | -0.052803483 | 3 | 115.24196 | 23.758888 | 72.419998 | 0 | 21 | 3 | 1 | 1 | 1 | 0 | 0 | 1 | 1 |  | NA |  | NA | 2 | 2 | 2 | 1 |  | 0 |  | 4 | 24 | 0 | 7 | 0 |  |
| 2692 |  | 22.286 | 2.797 | 12.55048 | 87.449516 | 0 | 11.394935 | 1 | 1.4721255 | 3 | 82.673401 | 21.576666 | 70.186 | 0 | 0 | 3 | 1 |  | | | | | | 132.9096977 | 2.991388446 | 66.75076955 | -3.894076376 | 2 | 2 |  | 0 | 0 | 1 | 1 | 1 |  | | | | |
| 2692 | 0 | 16.865 | 1.75 | 10.376519 | 89.623482 | 1 | 12.761123 | 1 | 1.4721255 | 3 | 82.673401 | 25.446112 | 70.510002 | 0 | 19 | 3 | 1 | 0 | 0 | 0 | 0 | 0 | 0 |  | NA |  | NA | 2 | 2 | 2 | 0 | 0 | 1 |  | 1 | 24 | 0 | 7 | 0 |  |
| 2692 | 1 | 19.709 | 2.1199999 | 10.756507 | 89.243492 | 1 | 12.807666 | 1 | 1.4721255 | 3 | 82.673401 | 25.207779 | 73.209999 | 0 | 20 | 3 | 1 | 1 | 1 | 0 | 0 | 1 | 0 | 112.8720103 | 1.177453946 | 73.01636278 | -3.358048708 | 2 | 2 | 2 | 0 | 0 | 1 | 1 | 1 | 24 | 0 |  | | |
| 2692 | 0 | 21.664 | 2.3599999 | 10.893648 | 89.106354 | 1 | 12.829569 | 1 | 1.4721255 | 3 | 82.673401 | 24.337221 | 71.050003 | 0 | 21 | 3 | 1 | 0 | 0 | 0 | 0 | 0 | 0 |  | NA |  | NA | 2 | 2 | 2 | 0 | 0 | 1 |  | 1 | 24 | 0 | 7 | 0 |  |
| 2693 |  | 22.205 | 2.348 | 10.574195 | 89.425804 | 1 | 16.85421 | 1 | 1.6736273 | 3 | 109.40079 | 22.824444 | 70.841 | 0 | 0 | 2 |  | | | | | | | 128.2934855 | 2.563470103 | 91.11011877 | -1.227274922 | 2 | 2 |  | 0 | 0 | 0 | 0 |  | | | | | |
| 2693 | 0 | 21.489 | 2.2 | 10.237796 | 89.762207 | 1 | 18.269678 | 1 | 1.6736273 | 3 | 109.40079 | 25.725555 | 70.629997 | 0 | 20 | 2 |  | 0 | 0 | 0 | 0 | 0 | 0 |  | NA |  | NA | 2 | 2 | 2 | 0 | 0 | 0 |  | | 24 | 0 |  | | |
| 2693 | 0 | 30.441999 | 2.75 | 9.0335722 | 90.966431 | 1 | 18.310745 | 1 | 1.6736273 | 3 | 109.40079 | 24.325003 | 70.089996 | 0 | 21 | 2 |  | 0 | 0 | 0 | 0 | 0 | 0 |  | NA |  | NA | 2 | 2 | 2 | 0 | 0 | 0 |  | | 24 | 0 | 7 | 0 |  |
| 2695 |  | 21.896 | 2.685 | 12.262514 | 87.737488 | 1 | 14.135524 | 0 | -1.9491892 | 3 | 88.447289 | 20.75 | 74.674 | 0 | 0 | 3 | 0 |  | | | | | | 100.5766537 | 0.050322211 | 93.93195468 | -0.965876553 | 2 | 2 |  | 0 | 0 | 0 | 0 | 2 |  | | | | |
| 2695 | 0 | 15.955 | 1.89 | 11.845817 | 88.154182 | 1 | 15.214237 | 0 | -1.9491892 | 3 | 88.447289 | 24.820002 | 72.839996 | 0 | 19 | 3 | 0 | 0 | 0 | 0 | 0 | 0 | 0 |  | NA |  | NA | 2 | 2 | 2 | 0 | 0 | 0 |  | 2 | 24 | 0 | 7 | 0 |  |
| 2695 | 0 | 20.296 | 2.1600001 | 10.642491 | 89.357506 | 1 | 15.266256 | 0 | -1.9491892 | 3 | 88.447289 | 24.409443 | 76.32 | 0 | 20 | 3 | 0 | 0 | 0 | 0 | 0 | 0 | 0 |  | NA |  | NA | 2 | 2 | 2 | 0 | 0 | 0 |  | 2 | 24 | 0 |  | | |
| 2695 | 0 | 21.650999 | 2.4000001 | 11.084939 | 88.915062 | 1 | 15.304585 | 0 | -1.9491892 | 3 | 88.447289 | 23.626667 | 73.980003 | 0 | 21 | 3 | 0 | 0 | 0 | 0 | 0 | 0 | 0 |  | NA |  | NA | 2 | 2 | 2 | 0 | 0 | 0 |  | 2 | 25 | 0 | 7 | 0 |  |
| 2697 |  | 22.66 | 2.918 | 12.877316 | 87.122681 | 1 | 13.322382 | 1 | -0.009923273 | 2 | 102.16822 | 20.346666 | 71.271 | 0 | 0 | 3 | 1 |  | | | | | | 117.4473031 | 1.593607107 | 92.15172413 | -1.159050803 | 2 | 2 |  | 0 | 0 | 0 | 0 | 2 |  | | | | |
| 2697 | 0 | 17.658001 | 1.84 | 10.420205 | 89.579796 | 1 | 14.663929 | 1 | -0.009923273 | 2 | 102.16822 | 25.45278 | 71.489998 | 0 | 19 | 3 | 1 | 0 | 0 | 0 | 0 | 0 | 0 |  | NA |  | NA | 2 | 2 | 2 | 0 | 0 | 0 |  | 2 | 25 | 0 | 7 | 0 |  |
| 2697 | 1 | 20.263 | 2.1500001 | 10.610473 | 89.389526 | 1 | 14.702259 | 1 | -0.009923273 | 2 | 102.16822 | 25.28389 | 71.339996 | 0 | 20 | 3 | 1 | 1 | 0 | 1 | 1 | 0 | 1 | 122.2190298 | 2.022191801 | 105.0991812 | 0.817501656 | 2 | 2 | 2 | 0 | 0 | 0 | 0 | 2 | 23 | 0 |  | | |
| 2697 | 1 | 24.047001 | 2.6300001 | 10.936915 | 89.063087 | 1 | 14.740588 | 1 | -0.009923273 | 2 | 102.16822 | 24.064445 | 70.839996 | 0 | 21 | 3 | 1 | 1 | 1 | 0 | 0 | 1 | 0 |  | NA |  | NA | 2 | 2 | 2 | 0 | 0 | 0 |  | 2 | 24 | 0 | 6.8000002 | 1 |  |
| 2698 |  | 22.515 | 2.869 | 12.742616 | 87.257385 | 1 | 12.736482 | 1 | 0.61584717 | 3 | 122.80058 | 20.031666 | 72.59 | 0 | 0 | 3 | 1 |  | | | | | | 129.1172107 | 2.654057512 | 101.5039255 | 0.243504207 | 2 | 2 |  | 0 | 0 | 0 | 1 | 3 |  | | | | |
| 2698 | 0 | 17.778999 | 1.91 | 10.743012 | 89.256989 | 1 | 14.036961 | 1 | 0.61584717 | 3 | 122.80058 | 25.796665 | 69.18 | 0 | 19 | 3 | 1 | 0 | 0 | 0 | 0 | 0 | 0 |  | NA |  | NA | 2 | 2 | 2 | 0 | 0 | 0 |  | 3 | 24 | 0 | 7 | 0 |  |
| 2698 | 1 | 20.582001 | 2.24 | 10.883296 | 89.116707 | 1 | 14.069816 | 1 | 0.61584717 | 3 | 122.80058 | 25.763334 | 70.809998 | 0 | 20 | 3 | 1 | 1 | 1 | 0 | 1 | 1 | 1 | 96.03927852 | -0.36322957 | 114.3300778 | 2.5082226 | 2 | 2 | 2 | 0 | 0 | 0 | 1 | 3 | 21 | 0 |  | | |
| 2698 | 1 | 23.035999 | 2.52 | 10.9394 | 89.0606 | 1 | 14.108145 | 1 | 0.61584717 | 3 | 122.80058 | 24.530556 | 69.260002 | 0 | 21 | 3 | 1 | 1 | 1 | 1 | 1 | 1 | 1 |  | NA |  | NA | 2 | 2 | 2 | 0 | 0 | 0 |  | 3 | 21 | 0 | 5.0999999 | 1 |  |
| 2699 |  | 22.267 | 2.781 | 12.489334 | 87.510666 | 1 | 15.739904 | 1 | 0.90343398 | 3 | 103.50712 | 22.263889 | 69.238 | 0 | 0 | 3 | 1 |  | | | | | | 108.9375843 | 0.814578426 | 101.3695236 | 0.208828691 | 2 | 2 |  | 0 | 0 | 0 | 0 | 4 |  | | | | |
| 2699 | 1 | 16.684 | 1.73 | 10.369216 | 89.630783 | 1 | 16.840521 | 1 | 0.90343398 | 3 | 103.50712 | 25.030001 | 72.449997 | 0 | 19 | 3 | 1 | 1 | 1 | 1 | 0 | 1 | 0 |  | NA |  | NA | 2 | 2 | 2 | 0 | 0 | 0 |  | 4 | 24 | 0 | 7 | 0 |  |
| 2699 | 0 | 19.774 | 2.1300001 | 10.771721 | 89.228279 | 1 | 16.881588 | 1 | 0.90343398 | 3 | 103.50712 | 24.961666 | 74.769997 | 0 | 20 | 3 | 1 | 0 | 0 | 0 | 0 | 0 | 0 | 111.2519069 | 1.023359323 | 104.3363007 | 0.664465705 | 2 | 2 | 2 | 0 | 0 | 0 | 0 | 4 | 24 | 0 |  | | |
| 2699 | 0 | 21.684999 | 2.3399999 | 10.79087 | 89.209129 | 1 | 16.919918 | 1 | 0.90343398 | 3 | 103.50712 | 23.813889 | 72.779999 | 0 | 21 | 3 | 1 | 0 | 0 | 0 | 0 | 0 | 0 |  | NA |  | NA | 2 | 2 | 2 | 0 | 0 | 0 |  | 4 | 25 | 0 | 7 | 0 |  |
| 2701 |  | 22.153 | 2.742 | 12.377556 | 87.622444 | 0 | 11.947981 | 1 | 1.7179354 | 2 | 112.29668 | 22.82 | 68.605 | 0 | 0 | 3 | 1 |  | | | | | | 123.5233733 | 2.146060279 | 97.19903231 | -0.441280643 | 2 | 2 |  | 1 |  | 1 | 0 | 3 |  | | | | |
| 2701 | 1 | 16.271999 | 1.7 | 10.447395 | 89.552605 | 1 | 13.278576 | 1 | 1.7179354 | 2 | 112.29668 | 25.031666 | 72.470001 | 0 | 19 | 3 | 1 | 1 | 1 | 1 | 1 | 1 | 1 |  | NA |  | NA | 2 | 2 | 1 | 1 |  | 1 |  | 3 | 20 | 0 | 6.5999999 | 1 |  |
| 2701 | 0 | 18.749001 | 2 | 10.667235 | 89.332764 | 1 | 13.319644 | 1 | 1.7179354 | 2 | 112.29668 | 24.532221 | 75.970001 | 0 | 20 | 3 | 1 | 0 | 0 | 0 | 0 | 0 | 0 |  | NA |  | NA | 2 | 2 | 2 | 1 |  | 1 |  | 3 | 23 | 0 |  | | |
| 2701 | 1 | 20.836 | 2.23 | 10.70263 | 89.297371 | 1 | 13.366187 | 1 | 1.7179354 | 2 | 112.29668 | 24.067223 | 73.559998 | 0 | 21 | 3 | 1 | 1 | 0 | 1 | 0 | 0 | 0 |  | NA |  | NA | 2 | 2 | 2 | 1 |  | 1 |  | 3 | 22 | 0 | 7 | 0 |  |
| 2703 |  | 22.367 | 2.834 | 12.670452 | 87.329552 | 1 | 13.483915 | 0 | 1.9194371 | 4 | 129.57594 | 20.448334 | 71.567 | 0 | 0 | 3 | 1 |  | | | | | | 135.739613 | 3.047159266 | 102.2140902 | 0.400071226 | 2 | 2 |  | 0 | 0 | 1 | 0 | 1 |  | | | | |
| 2703 | 1 | 17.01 | 1.76 | 10.346855 | 89.653145 | 1 | 14.822724 | 0 | 1.9194371 | 4 | 129.57594 | 25.883331 | 68.540001 | 0 | 19 | 3 | 1 | 1 | 1 | 1 | 1 | 1 | 1 |  | NA |  | NA | 2 | 2 | 2 | 0 | 0 | 1 |  | 1 | 24 | 0 | 7 | 0 |  |
| 2703 | 1 | 19.872999 | 2.1400001 | 10.76838 | 89.231621 | 1 | 14.872005 | 0 | 1.9194371 | 4 | 129.57594 | 25.607779 | 71.129997 | 0 | 20 | 3 | 1 | 1 | 0 | 0 | 0 | 1 | 0 | 100.2288087 | 0.019862228 | 94.51820944 | -0.872879057 | 2 | 2 | 2 | 0 | 0 | 1 | 0 | 1 | 24 | 0 |  | | |
| 2703 | 1 | 22.000999 | 2.4100001 | 10.954048 | 89.045952 | 1 | 14.896646 | 0 | 1.9194371 | 4 | 129.57594 | 24.68722 | 69.139999 | 0 | 21 | 3 | 1 | 1 | 0 | 0 | 0 | 1 | 0 |  | NA |  | NA | 2 | 2 | 2 | 0 | 0 | 1 |  | 1 | 24 | 0 | 7 | 0 |  |
| 2704 |  | 22.405 | 2.874 | 12.827494 | 87.172508 | 1 | 18.026011 | 0 | -0.1784503 | 2 | 84.249115 | 18.508333 | 73.891 | 0 | 0 | 3 | 1 |  | | | | | | 93.74411052 | -0.538234476 | 95.93619017 | -0.639431236 | 2 | 2 |  | 0 | 0 | 0 | 1 | 3 |  | | | | |
| 2704 | 0 | 17.309999 | 1.88 | 10.860775 | 89.139229 | 1 | 19.375771 | 0 | -0.1784503 | 2 | 84.249115 | 26.60611 | 65.919998 | 0 | 19 | 3 | 1 | 0 | 0 | 0 | 0 | 0 | 0 |  | NA |  | NA | 2 | 2 | 2 | 0 | 0 | 0 |  | 3 | 24 | 0 | 7 | 0 |  |
| 2704 | 1 | 20.497999 | 2.28 | 11.123037 | 88.876961 | 1 | 19.414101 | 0 | -0.1784503 | 2 | 84.249115 | 26.174444 | 67.290001 | 0 | 20 | 3 | 1 | 0 | 1 | 0 | 0 | 0 | 1 | 95.1979496 | -0.410477923 | 104.3846045 | 0.75451963 | 2 | 2 | 2 | 0 | 0 | 0 | 0 | 3 | 25 | 0 |  | | |
| 2704 | 0 | 23.455999 | 2.5599999 | 10.914052 | 89.085945 | 1 | 19.452431 | 0 | -0.1784503 | 2 | 84.249115 | 25.040554 | 66.419998 | 0 | 21 | 3 | 1 | 0 | 0 | 0 | 0 | 0 | 0 |  | NA |  | NA | 2 | 2 | 2 | 0 | 0 | 0 |  | 3 | 24 | 0 | 7 | 0 |  |
| 2705 |  | 22.608 | 2.861 | 12.654813 | 87.345184 | 1 | 14.830937 | 1 | 0.24810082 | 2 | 95.223312 | 20.545555 | 72.865 | 0 | 0 | 3 | 1 |  | | | | | | 111.6291015 | 1.060742976 | 91.4626447 | -1.219568892 | 2 | 2 |  | 0 | 0 | 1 | 0 | 2 |  | | | | |
| 2705 | 1 | 17.934 | 1.99 | 11.096242 | 88.903755 | 1 | 16.120466 | 1 | 0.24810082 | 2 | 95.223312 | 25.478889 | 70.739998 | 0 | 19 | 3 | 1 | 1 | 1 | 1 | 0 | 1 | 0 |  | NA |  | NA | 2 | 2 | 2 | 0 | 0 | 1 |  | 2 | 24 | 0 | 7 | 0 |  |
| 2705 | 0 | 21.295 | 2.3199999 | 10.894576 | 89.105423 | 1 | 16.158794 | 1 | 0.24810082 | 2 | 95.223312 | 25.201111 | 71.760002 | 0 | 20 | 3 | 1 | 0 | 0 | 0 | 0 | 0 | 0 |  | NA |  | NA | 2 | 2 | 2 | 0 | 0 | 1 |  | 2 | 24 | 0 |  | | |
| 2705 | 1 | 23.533001 | 2.55 | 10.835847 | 89.164154 | 1 | 16.197124 | 1 | 0.24810082 | 2 | 95.223312 | 24.18 | 70.720001 | 0 | 21 | 3 | 1 | 1 | 1 | 0 | 0 | 0 | 0 |  | NA |  | NA | 2 | 2 | 2 | 0 | 0 | 1 |  | 2 | 15 | 1 | 6.4000001 | 1 |  |
| 2706 |  | 22.578 | 2.845 | 12.600761 | 87.399239 | 1 | 14.477755 | 1 | -1.0517983 | 2 | 108.77458 | 20.598888 | 73.395 | 0 | 0 | 3 | 0 |  | | | | | | 110.5669322 | 0.964780474 | 105.2430322 | 0.845495759 | 2 | 2 |  | 0 | 0 | 0 | 0 |  | | | | | |
| 2706 | 0 | 17.854 | 2.01 | 11.257981 | 88.74202 | 1 | 15.857632 | 1 | -1.0517983 | 2 | 108.77458 | 25.226665 | 71.75 | 0 | 19 | 3 | 0 | 0 | 0 | 0 | 0 | 0 | 0 |  | NA |  | NA | 2 | 2 | 2 | 0 | 0 | 0 |  | | 24 | 0 | 7 | 0 |  |
| 2706 | 0 | 21.138 | 2.3 | 10.880878 | 89.119125 | 1 | 15.895962 | 1 | -1.0517983 | 2 | 108.77458 | 24.966112 | 72.870003 | 0 | 20 | 3 | 0 | 0 | 0 | 0 | 0 | 0 | 0 | 104.4889732 | 0.409473168 | 111.4416511 | 1.882623609 | 2 | 2 | 2 | 0 | 0 | 0 | 0 |  | 25 | 0 |  | | |
| 2706 | 0 | 23.136 | 2.51 | 10.848893 | 89.151108 | 1 | 15.934292 | 1 | -1.0517983 | 2 | 108.77458 | 24.000553 | 71.720001 | 0 | 21 | 3 | 0 | 0 | 0 | 0 | 0 | 0 | 0 |  | NA |  | NA | 2 | 2 | 2 | 0 | 0 | 0 |  | | 25 | 0 | 7 | 0 |  |
| 2707 |  | 22.403 | 2.812 | 12.55189 | 87.448112 | 1 | 13.297741 | 1 | 0.47940791 | 3 | 119.3242 | 22.297777 | 69.248 | 0 | 0 | 3 | 1 |  | | | | | | 124.0188399 | 2.190629358 | 102.675537 | 0.432983852 | 2 | 2 |  | 1 | 0 | 0 | 0 | 3 |  | | | | |
| 2707 | 0 | 16.957001 | 1.73 | 10.202275 | 89.797722 | 1 | 14.633813 | 1 | 0.47940791 | 3 | 119.3242 | 25.09889 | 72.190002 | 0 | 19 | 3 | 1 | 0 | 0 | 0 | 0 | 0 | 0 |  | NA |  | NA | 2 | 2 | 2 | 1 | 0 | 0 |  | 3 | 24 | 0 | 7 | 0 |  |
| 2707 | 0 | 19.434 | 2.0799999 | 10.702891 | 89.297112 | 1 | 14.663929 | 1 | 0.47940791 | 3 | 119.3242 | 24.725554 | 75.769997 | 0 | 20 | 3 | 1 | 0 | 0 | 0 | 0 | 0 | 0 | 106.6970576 | 0.611529667 | 111.6409236 | 1.956850729 | 2 | 2 | 2 | 1 | 0 | 0 | 0 | 3 | 24 | 0 |  | | |
| 2707 | 0 | 20.754999 | 2.21 | 10.648037 | 89.351959 | 1 | 14.702259 | 1 | 0.47940791 | 3 | 119.3242 | 24.513889 | 72.959999 | 0 | 21 | 3 | 1 | 0 | 0 | 0 | 0 | 0 | 0 |  | NA |  | NA | 2 | 2 | 2 | 1 | 0 | 0 |  | 3 | 25 | 0 | 7 | 0 |  |
| 2708 |  | 22.405 | 2.813 | 12.555233 | 87.444763 | 1 | 13.158111 | 1 | 0.37003732 | 3 | 110.97848 | 22.276112 | 69.265 | 0 | 0 | 3 | 1 |  | | | | | | 119.9456807 | 1.821113261 | 98.99720308 | -0.157670055 | 2 | 2 |  | 0 | 0 | 1 | 0 | 3 |  | | | | |
| 2708 | 0 | 17.146999 | 1.8 | 10.497464 | 89.502533 | 1 | 14.516085 | 1 | 0.37003732 | 3 | 110.97848 | 25.007778 | 73.019997 | 0 | 19 | 3 | 1 | 0 | 0 | 0 | 0 | 0 | 0 |  | NA |  | NA | 2 | 2 | 2 | 0 | 0 | 1 |  | 3 | 24 | 0 | 7 | 0 |  |
| 2708 | 0 | 20.127001 | 2.1500001 | 10.682168 | 89.317833 | 1 | 14.554415 | 1 | 0.37003732 | 3 | 110.97848 | 24.919447 | 73.980003 | 0 | 20 | 3 | 1 | 0 | 0 | 0 | 0 | 0 | 0 | 111.6486935 | 1.062625882 | 106.6840789 | 1.083543602 | 2 | 2 | 2 | 0 | 0 | 1 | 0 | 3 | 24 | 0 |  | | |
| 2708 | 0 | 21.951 | 2.3699999 | 10.796774 | 89.203224 | 1 | 14.587269 | 1 | 0.37003732 | 3 | 110.97848 | 23.744444 | 72.769997 | 0 | 21 | 3 | 1 | 0 | 0 | 0 | 0 | 0 | 0 |  | NA |  | NA | 2 | 2 | 2 | 0 | 0 | 1 |  | 3 | 24 | 0 | 6.9000001 | 1 |  |
| 2709 |  | 23.238 | 3.006 | 12.935709 | 87.064293 | 1 | 17.544147 | 0 | -3.8109183 | 2 | 85.050339 | 21.837778 | 69.9 | 0 | 0 | 3 | 0 |  | | | | | | 110.0738218 | 0.856166471 | 82.61178164 | -2.303463926 | 2 | 2 |  | 1 | 1 | 0 | 1 | 1 |  | | | | |
| 2709 | 0 | 16.687 | 1.71 | 10.247499 | 89.752502 | 1 | 18.861053 | 0 | -3.8109183 | 2 | 85.050339 | 25.100555 | 72.349998 | 0 | 19 | 3 | 0 | 0 | 0 | 0 | 0 | 0 | 0 |  | NA |  | NA | 2 | 2 | 2 | 1 | 1 | 0 |  | 1 | 24 | 0 | 7 | 0 |  |
| 2709 | 1 | 18.830999 | 1.99 | 10.567681 | 89.43232 | 1 | 18.910336 | 0 | -3.8109183 | 2 | 85.050339 | 24.738888 | 75.290001 | 0 | 20 | 3 | 0 | 1 | 1 | 0 | 0 | 1 | 0 | 93.26062332 | -0.577307221 | 83.0723069 | -2.229016431 | 2 | 2 | 2 | 1 | 1 | 0 | 1 | 1 | 19 | 1 |  | | |
| 2709 | 1 | 21.461 | 2.3099999 | 10.76371 | 89.23629 | 1 | 18.937714 | 0 | -3.8109183 | 2 | 85.050339 | 23.923891 | 73.160004 | 0 | 21 | 3 | 0 | 1 | 0 | 0 | 0 | 1 | 0 |  | NA |  | NA | 2 | 2 | 2 | 1 | 1 | 0 |  | 1 | 24 | 0 | 7 | 0 |  |
| 2711 |  | 22.611 | 3.005 | 13.289991 | 86.710007 | 0 | 10.464066 | 1 | -1.1737348 | 2 | 117.90459 | 24.047222 | 65.311 | 0 | 0 | 3 |  | | | | | | | 135.1949469 | 3.176185222 | 93.36327407 | -1.010025433 | 2 | 1 |  | 0 | 0 | 1 | 0 | 3 |  | | | | |
| 2711 | 0 | 17.437 | 1.79 | 10.265528 | 89.734474 | 0 | 11.734428 | 1 | -1.1737348 | 2 | 117.90459 | 26.204443 | 66.870003 | 0 | 19 | 3 |  | 0 | 0 | 0 | 0 | 0 |  | | NA |  | NA | 2 | 1 | 2 | 0 | 0 | 1 |  | 3 | 27 | 0 | 7 | 0 |  |
| 2711 | 1 | 18.896 | 2.04 | 10.795936 | 89.204063 | 0 | 11.78371 | 1 | -1.1737348 | 2 | 117.90459 | 25.855001 | 68.959999 | 0 | 20 | 3 |  | 1 | 0 | 0 | 0 | 0 |  | 90.44682494 | -0.878958974 | 96.1876996 | -0.59410154 | 2 | 1 | 2 | 0 | 0 | 1 | 1 | 3 | 20 | 0 |  | | |
| 2711 | 1 | 24.792 | 2.78 | 11.213295 | 88.786705 | 0 | 11.824778 | 1 | -1.1737348 | 2 | 117.90459 | 25.413336 | 67.779999 | 0 | 21 | 3 |  | 1 | 0 | 1 | 0 | 0 |  | | NA |  | NA | 2 | 1 | 2 | 0 | 0 | 1 |  | 3 | 27 | 0 | 7 | 0 |  |
| 2712 |  | 22.703 | 2.983 | 13.139233 | 86.860764 | 0 | 10.321697 | 0 | 0.19843566 | 4 | 96.925148 | 23.088888 | 67.84 | 0 | 0 | 3 | 0 |  | | | | | | 103.8130835 | 0.338211429 | 99.74820097 | -0.045245068 | 2 | 2 |  | 0 | 0 | 0 | 0 | 4 |  | | | | |
| 2712 | 0 | 17.891001 | 1.83 | 10.228606 | 89.771393 | 0 | 11.66872 | 0 | 0.19843566 | 4 | 96.925148 | 25.844442 | 68.160004 | 0 | 19 | 3 | 0 | 0 | 0 | 0 | 0 | 0 |  | | NA |  | NA | 2 | 2 | 2 | 0 | 0 | 0 |  | 4 | 26 | 0 | 7 | 0 |  |
| 2712 | 0 | 20.214001 | 2.1500001 | 10.636192 | 89.363808 | 0 | 11.704312 | 0 | 0.19843566 | 4 | 96.925148 | 25.443892 | 70.860001 | 0 | 20 | 3 | 0 | 0 | 0 | 0 | 0 | 0 |  | 100.2563957 | 0.022725294 | 94.4164244 | -0.911920903 | 2 | 2 | 2 | 0 | 0 | 0 | 0 | 4 | 25 | 0 |  | | |
| 2712 | 1 | 23.868 | 2.6500001 | 11.102732 | 88.89727 | 0 | 11.742642 | 0 | 0.19843566 | 4 | 96.925148 | 25.236113 | 69.459999 | 0 | 21 | 3 | 0 | 1 | 0 | 1 | 1 | 0 |  | | NA |  | NA | 2 | 2 | 2 | 0 | 0 | 0 |  | 4 | 21 | 0 | 6.3000002 | 1 |  |
| 2713 |  | 22.66 | 3.161 | 13.949691 | 86.050308 | 1 | 13.664614 | 0 | 1.7815342 | 3 | 105.036 | 26.852222 | 59.254 | 0 | 0 | 3 | 0 |  | | | | | | 116.1997481 | 1.399787981 | 96.55675441 | -0.571828636 | 2 | 2 |  | 0 | 0 | 0 | 0 | 3 |  | | | | |
| 2713 | 1 | 16.542 | 1.73 | 10.458227 | 89.541771 | 1 | 15.017112 | 0 | 1.7815342 | 3 | 105.036 | 26.574999 | 65.129997 | 0 | 19 | 3 | 0 | 1 | 1 | 1 | 1 | 1 | 0 |  | NA |  | NA | 2 | 2 | 2 | 0 | 0 | 0 |  | 3 | 21 | 0 | 7 | 0 |  |
| 2713 | 1 | 17.834999 | 2.04 | 11.438184 | 88.561813 | 1 | 15.041752 | 0 | 1.7815342 | 3 | 105.036 | 26.663891 | 66.790001 | 0 | 20 | 3 | 0 | 1 | 1 | 1 | 1 | 1 | 1 | 78.87071933 | -1.870721354 | 101.7522065 | 0.308313383 | 2 | 2 | 2 | 0 | 0 | 0 | 0 | 3 | 16 | 1 |  | | |
| 2713 | 0 | 26.221001 | 2.99 | 11.403073 | 88.596924 | 1 | 15.091034 | 0 | 1.7815342 | 3 | 105.036 | 26.118334 | 65.18 | 0 | 21 | 3 | 0 | 0 | 0 | 0 | 0 | 0 | 0 |  | NA |  | NA | 2 | 2 | 2 | 0 | 0 | 0 |  | 3 | 19 | 1 | 4.8000002 | 1 |  |
| 2714 |  | 23.139 | 3.312 | 14.313497 | 85.686501 | 1 | 15.077344 | 1 | 0.6383335 | 3 | 127.10451 | 26.781111 | 59.468 | 0 | 0 | 3 | 0 |  | | | | | | 133.2907013 | 3.020470894 | 102.2031349 | 0.342384537 | 2 | 2 |  | 0 | 0 | 0 | 0 | 4 |  | | | | |
| 2714 | 1 | 16.445999 | 1.71 | 10.397666 | 89.602333 | 1 | 16.405203 | 1 | 0.6383335 | 3 | 127.10451 | 26.577221 | 65.160004 | 0 | 19 | 3 | 0 | 1 | 1 | 1 | 1 | 1 | 1 |  | NA |  | NA | 2 | 2 | 2 | 0 | 0 | 0 |  | 4 | 24 | 0 | 7 | 0 |  |
| 2714 | 1 | 17.738001 | 2.04 | 11.500732 | 88.499268 | 1 | 16.429842 | 1 | 0.6383335 | 3 | 127.10451 | 26.626112 | 66.93 | 0 | 20 | 3 | 0 | 1 | 1 | 0 | 0 | 1 | 1 | 117.9053035 | 1.626683382 | 111.8505494 | 1.935430748 | 2 | 2 | 2 | 0 | 0 | 0 | 0 | 4 | 23 | 0 |  | | |
| 2714 | 0 | 26.246 | 2.99 | 11.392212 | 88.607788 | 1 | 16.479124 | 1 | 0.6383335 | 3 | 127.10451 | 26.086668 | 65.290001 | 0 | 21 | 3 | 0 | 0 | 0 | 0 | 0 | 0 | 0 |  | NA |  | NA | 2 | 2 | 2 | 0 | 0 | 0 |  | 4 | 24 | 0 | 7 | 0 |  |
| 2715 |  | 22.536 | 3.2 | 14.199503 | 85.800499 | 0 | 9.1389456 | 1 | 1.7179354 | 3 | 114.48183 | 29.538889 | 52.715 | 0 | 0 | 3 | 1 |  | | | | | | 136.8196728 | 3.251948876 | 89.10696409 | -1.55730757 | 2 | 2 |  | 0 | 0 | 1 | 1 | 2 |  | | | | |
| 2715 | 0 | 15.925 | 1.6900001 | 10.612246 | 89.387756 | 0 | 10.49692 | 1 | 1.7179354 | 3 | 114.48183 | 27.44278 | 61.84 | 0 | 19 | 3 | 1 | 0 | 0 | 0 | 0 | 0 |  | | NA |  | NA | 2 | 2 | 2 | 0 | 0 | 1 |  | 2 | 26 | 0 | 7 | 0 |  |
| 2715 | 0 | 16.561001 | 1.96 | 11.835033 | 88.16497 | 0 | 10.521561 | 1 | 1.7179354 | 3 | 114.48183 | 27.744446 | 62.639999 | 0 | 20 | 3 | 1 | 0 | 0 | 0 | 0 | 0 |  | 128.4863253 | 2.579884596 | 88.56004957 | -1.662447248 | 2 | 2 | 2 | 0 | 0 | 1 | 0 | 2 | 26 | 0 |  | | |
| 2715 | 0 | 27.57 | 3.1900001 | 11.570548 | 88.429451 | 0 | 10.57358 | 1 | 1.7179354 | 3 | 114.48183 | 27.051668 | 61.330002 | 0 | 21 | 3 | 1 | 0 | 0 | 0 | 0 | 0 |  | | NA |  | NA | 2 | 2 | 2 | 0 | 0 | 1 |  | 2 | 27 | 0 | 7 | 0 |  |
| 2716 |  | 22.464 | 3.089 | 13.750891 | 86.249107 | 1 | 14.302532 | 0 | 1.5357243 | 3 | 108.44859 | 27.132778 | 58.268 | 0 | 0 | 3 |  | | | | | | | 143.5283329 | 3.67068119 | 80.73745833 | -2.527768663 | 2 | 2 |  | 0 | 0 | 0 | 1 | 1 |  | | | | |
| 2716 | 0 | 16.584 | 1.72 | 10.371443 | 89.628555 | 1 | 15.627652 | 0 | 1.5357243 | 3 | 108.44859 | 26.713888 | 64.470001 | 0 | 19 | 3 |  | 0 | 0 | 0 | 0 | 0 | 0 |  | NA |  | NA | 2 | 2 | 2 | 0 | 0 | 0 |  | 1 | 24 | 0 | 7 | 0 |  |
| 2716 | 0 | 19.097 | 2.21 | 11.572498 | 88.427505 | 1 | 15.671458 | 0 | 1.5357243 | 3 | 108.44859 | 26.920557 | 65.489998 | 0 | 20 | 3 |  | 0 | 0 | 0 | 0 | 0 | 0 | 120.5926559 | 1.751085675 | 74.89718757 | -3.015164016 | 2 | 2 | 2 | 0 | 0 | 0 | 0 | 1 | 24 | 0 |  | | |
| 2716 | 1 | 26.896 | 3.05 | 11.339976 | 88.660027 | 1 | 15.704312 | 0 | 1.5357243 | 3 | 108.44859 | 26.200558 | 64.160004 | 0 | 21 | 3 |  | 1 | 0 | 1 | 0 | 0 | 0 |  | NA |  | NA | 2 | 2 | 2 | 0 | 0 | 0 |  | 1 | 23 | 0 | 7 | 0 |  |
| 2717 |  | 24.235 | 3.193 | 13.175159 | 86.824837 | 1 | 17.201916 | 1 | 1.4721255 | 2 | 102.48205 | 20.247778 | 73.065 | 0 | 0 | 3 |  | | | | | | | 103.994309 | 0.363866584 | 105.2229061 | 0.80309019 | 2 | 2 |  | 1 | 1 | 0 | 0 | 3 |  | | | | |
| 2717 | 0 | 18.648001 | 2 | 10.72501 | 89.274986 | 1 | 18.324436 | 1 | 1.4721255 | 2 | 102.48205 | 25.725555 | 69.620003 | 0 | 19 | 3 |  | 0 | 0 | 0 | 0 | 0 | 0 |  | NA |  | NA | 2 | 2 | 2 | 1 | 1 | 0 |  | 3 | 24 | 0 | 7 | 0 |  |
| 2717 | 0 | 22.458 | 2.47 | 10.998308 | 89.001694 | 1 | 18.362764 | 1 | 1.4721255 | 2 | 102.48205 | 25.355555 | 70.230003 | 0 | 20 | 3 |  | 0 | 0 | 0 | 0 | 0 | 0 |  | NA |  | NA | 2 | 2 | 2 | 1 | 1 | 0 |  | 3 | 25 | 0 |  | | |
| 2717 | 0 | 24.879999 | 2.6600001 | 10.691319 | 89.308678 | 1 | 18.401094 | 1 | 1.4721255 | 2 | 102.48205 | 24.342779 | 69.620003 | 0 | 21 | 3 |  | 0 | 0 | 0 | 0 | 0 | 0 |  | NA |  | NA | 2 | 2 | 2 | 1 | 1 | 0 |  | 3 | 24 | 0 | 7 | 0 |  |
| 2718 |  | 23.057 | 2.982 | 12.933166 | 87.066833 | 1 | 13.385352 | 1 | -0.12736262 | 3 | 98.651772 | 21.565001 | 70.663 | 0 | 0 | 3 | 1 |  | | | | | | 112.1018547 | 1.106663633 | 95.74344459 | -0.64796707 | 2 | 2 |  | 0 | 0 | 1 | 0 | 3 |  | | | | |
| 2718 | 1 | 18.870001 | 1.9400001 | 10.280869 | 89.719131 | 1 | 14.735113 | 1 | -0.12736262 | 3 | 98.651772 | 25.779999 | 68.389999 | 0 | 19 | 3 | 1 | 1 | 0 | 0 | 1 | 1 | 1 |  | NA |  | NA | 2 | 2 | 1 | 0 | 0 | 1 |  | 3 | 24 | 0 | 7 | 0 |  |
| 2718 | 1 | 22.319 | 2.3299999 | 10.439535 | 89.560463 | 1 | 14.767967 | 1 | -0.12736262 | 3 | 98.651772 | 25.655001 | 71.120003 | 0 | 20 | 3 | 1 | 1 | 1 | 1 | 1 | 1 | 1 | 84.38959947 | -1.43329073 | 100.6094956 | 0.093335073 | 2 | 2 | 1 | 0 | 0 | 1 | 0 | 3 | 18 | 1 |  | | |
| 2718 | 1 | 23.621 | 2.5999999 | 11.007154 | 88.992844 | 1 | 14.809035 | 1 | -0.12736262 | 3 | 98.651772 | 24.824444 | 69.889999 | 0 | 21 | 3 | 1 | 1 | 1 | 1 | 1 | 1 | 1 |  | NA |  | NA | 2 | 2 | 1 | 0 | 0 | 1 |  | 3 | 18 | 1 | 6.6999998 | 1 |  |
| 2721 |  | 22.52 | 2.986 | 13.259325 | 86.740677 | 0 | 11.315537 | 0 | -1.9491892 | 3 | 125.98743 | 20.356112 | 71.598 | 0 | 0 | 3 | 1 |  | | | | | | 139.9776609 | 3.444733828 | 96.07424244 | -0.660575476 | 2 | 2 |  | 0 | 0 | 1 | 0 | 2 |  | | | | |
| 2721 | 1 | 17.864 | 1.8099999 | 10.132109 | 89.867889 | 1 | 12.591375 | 0 | -1.9491892 | 3 | 125.98743 | 25.423889 | 69.449997 | 0 | 19 | 3 | 1 | 1 | 1 | 1 | 1 | 1 | 1 |  | NA |  | NA | 2 | 2 | 2 | 0 | 0 | 1 |  | 2 | 24 | 0 | 7 | 0 |  |
| 2721 | 1 | 21.278 | 2.3599999 | 11.091268 | 88.90873 | 1 | 12.637919 | 0 | -1.9491892 | 3 | 125.98743 | 25.209999 | 71.599998 | 0 | 20 | 3 | 1 | 1 | 1 | 1 | 1 | 1 | 1 | 128.4589103 | 2.453343393 | 94.79735711 | -0.849506993 | 2 | 2 | 2 | 0 | 0 | 1 | 0 | 2 | 23 | 0 |  | | |
| 2721 | 1 | 25.426001 | 2.78 | 10.933689 | 89.066307 | 1 | 12.67625 | 0 | -1.9491892 | 3 | 125.98743 | 23.846109 | 70.769997 | 0 | 21 | 3 | 1 | 1 | 1 | 1 | 1 | 1 | 1 |  | NA |  | NA | 2 | 2 | 2 | 0 | 0 | 1 |  | 2 | 23 | 0 | 6.4000001 | 1 |  |
| 2722 |  | 22.655 | 3.171 | 13.99691 | 86.00309 | 1 | 16.117727 | 0 | 0.53517872 | 2 | 105.74299 | 22.336666 | 73.86 | 0 | 0 | 2 | 0 |  | | | | | | 108.3115538 | 0.712177825 | 104.2190051 | 0.757888879 | 2 | 2 |  | 0 | 0 | 0 | 0 | 3 |  | | | | |
| 2722 | 0 | 18.59 | 2.02 | 10.866057 | 89.133942 | 1 | 17.508556 | 0 | 0.53517872 | 2 | 105.74299 | 23.320555 | 79.440002 | 0 | 20 | 2 | 0 | 0 | 0 | 0 | 0 | 0 | 0 | 101.661367 | 0.141991692 | 108.1623786 | 1.504215157 | 2 | 2 | 2 | 0 | 0 | 0 | 0 | 3 | 22 | 0 |  | | |
| 2722 | 1 | 25.243 | 2.79 | 11.052569 | 88.947433 | 1 | 17.546886 | 0 | 0.53517872 | 2 | 105.74299 | 22.996111 | 76.629997 | 0 | 21 | 2 | 0 | 1 | 1 | 0 | 0 | 1 | 0 |  | NA |  | NA | 2 | 2 | 2 | 0 | 0 | 0 |  | 3 | 24 | 0 | 7 | 0 |  |
| 2723 |  | 23.437 | 3.552 | 15.155523 | 84.844475 | 1 | 17.075975 | 0 | -0.24929914 | 2 | 98.105995 | 21.967777 | 74.532 | 0 | 0 | 3 | 1 |  | | | | | | 125.3788297 | 2.13558825 | 83.67077821 | -2.201261382 | 2 | 2 |  | 0 | 0 | 0 | 1 | 1 |  | | | | |
| 2723 | 0 | 16.666 | 1.65 | 9.9003963 | 90.099602 | 1 | 18.384668 | 0 | -0.24929914 | 2 | 98.105995 | 23.535 | 76.669998 | 0 | 19 | 3 | 1 | 0 | 0 | 0 | 0 | 0 | 0 |  | NA |  | NA | 2 | 2 | 2 | 0 | 0 | 0 |  | 1 | 24 | 0 | 7 | 0 |  |
| 2723 | 1 | 18.290001 | 2.03 | 11.098961 | 88.901039 | 1 | 18.422998 | 0 | -0.24929914 | 2 | 98.105995 | 23.380554 | 80.269997 | 0 | 20 | 3 | 1 | 1 | 0 | 0 | 0 | 1 | 1 | 105.3856197 | 0.457331513 | 107.1912116 | 1.293360897 | 2 | 2 | 2 | 0 | 0 | 0 | 0 | 1 | 24 | 0 |  | | |
| 2723 | 1 | 27.040001 | 3.02 | 11.168638 | 88.83136 | 1 | 18.453114 | 0 | -0.24929914 | 2 | 98.105995 | 22.666113 | 76.639999 | 0 | 21 | 3 | 1 | 1 | 0 | 1 | 1 | 0 | 1 |  | NA |  | NA | 2 | 2 | 2 | 0 | 0 | 0 |  | 1 | 25 | 0 | 7 | 0 |  |
| 2724 |  | 23.786 | 3.663 | 15.399815 | 84.600182 | 1 | 16.848734 | 1 | -1.5056298 | 2 | 97.118591 | 21.782223 | 74.098 | 0 | 0 | 3 | 1 |  | | | | | | 106.6725372 | 0.607604674 | 97.89773758 | -0.306599749 | 2 | 2 |  | 0 |  | 1 | 0 | 3 |  | | | | |
| 2724 | 1 | 15.914 | 1.61 | 10.116879 | 89.883125 | 1 | 18.072554 | 1 | -1.5056298 | 2 | 97.118591 | 24.097776 | 75.220001 | 0 | 19 | 3 | 1 | 1 | 1 | 1 | 1 | 1 | 1 |  | NA |  | NA | 2 | 2 | 2 | 0 |  | 1 |  | 3 | 24 | 0 | 7 | 0 |  |
| 2724 | 1 | 18.74 | 2.02 | 10.779082 | 89.220917 | 1 | 18.110884 | 1 | -1.5056298 | 2 | 97.118591 | 23.256666 | 78.989998 | 0 | 20 | 3 | 1 | 1 | 1 | 0 | 1 | 1 | 0 | 95.40368568 | -0.419245595 | 96.20232113 | -0.53819118 | 2 | 2 | 2 | 0 |  | 1 | 1 | 3 | 24 | 0 |  | | |
| 2724 | 0 | 24.43 | 2.6800001 | 10.970119 | 89.029884 | 1 | 18.151951 | 1 | -1.5056298 | 2 | 97.118591 | 23.214447 | 76.540001 | 0 | 21 | 3 | 1 | 0 | 0 | 0 | 0 | 0 | 0 |  | NA |  | NA | 2 | 2 | 2 | 0 |  | 1 |  | 3 | 24 | 0 | 7 | 0 |  |
| 2727 |  | 23.203 | 3.329 | 14.347282 | 85.652718 | 1 | 15.091034 | 1 | 1.2144388 | 2 | 99.040894 | 27.133888 | 58.505 | 0 | 0 | 3 | 1 |  | | | | | | 104.9696023 | 0.453837345 | 101.022729 | 0.157386852 | 2 | 2 |  | 0 | 0 | 0 | 0 | 3 |  | | | | |
| 2727 | 0 | 16.391001 | 1.72 | 10.493563 | 89.506439 | 1 | 16.416153 | 1 | 1.2144388 | 2 | 99.040894 | 26.753887 | 64.519997 | 0 | 19 | 3 | 1 | 0 | 0 | 0 | 0 | 0 | 0 |  | NA |  | NA | 2 | 2 | 2 | 0 | 0 | 0 |  | 3 | 24 | 0 | 7 | 0 |  |
| 2727 | 0 | 17.664 | 2.03 | 11.492301 | 88.507698 | 1 | 16.443531 | 1 | 1.2144388 | 2 | 99.040894 | 26.843334 | 66.129997 | 0 | 20 | 3 | 1 | 0 | 0 | 0 | 0 | 0 | 0 | 101.9321883 | 0.176203457 | 104.0643749 | 0.624368601 | 2 | 2 | 2 | 0 | 0 | 0 | 0 | 3 | 24 | 0 |  | | |
| 2727 | 1 | 26.055 | 2.99 | 11.475724 | 88.524277 | 1 | 16.4846 | 1 | 1.2144388 | 2 | 99.040894 | 26.337221 | 64.589996 | 0 | 21 | 3 | 1 | 1 | 0 | 1 | 1 | 1 | 0 |  | NA |  | NA | 2 | 2 | 2 | 0 | 0 | 0 |  | 3 | 19 | 1 | 6.5999999 | 1 |  |
| 2728 |  | 23.69 | 3.368 | 14.216969 | 85.783028 | 0 | 11.290896 | 1 | -1.8791564 | 4 | 97.340157 | 24.487223 | 64.662 | 0 | 0 | 3 | 1 |  | | | | | | 122.6279357 | 2.060492812 | 84.81535031 | -2.133115046 | 2 | 2 |  | 0 | 0 | 1 | 1 | 3 |  | | | | |
| 2728 | 0 | 17.166 | 1.76 | 10.252826 | 89.747177 | 1 | 12.594113 | 1 | -1.8791564 | 4 | 97.340157 | 26.006664 | 67.300003 | 0 | 19 | 3 | 1 | 0 | 0 | 0 | 0 | 0 | 0 |  | NA |  | NA | 2 | 2 | 1 | 0 | 0 | 1 |  | 3 | 19 | 1 | 5.5999999 | 1 |  |
| 2728 | 0 | 19.013 | 2.1199999 | 11.150265 | 88.849731 | 1 | 12.621492 | 1 | -1.8791564 | 4 | 97.340157 | 25.98111 | 69.480003 | 0 | 20 | 3 | 1 | 0 | 0 | 0 | 0 | 0 | 0 | 111.4234534 | 1.045375228 | 80.16475654 | -2.643585257 | 2 | 2 | 1 | 0 | 0 | 1 | 1 | 3 | 24 | 0 |  | | |
| 2728 | 0 | 25.278999 | 2.8399999 | 11.234622 | 88.765381 | 1 | 12.668036 | 1 | -1.8791564 | 4 | 97.340157 | 25.481665 | 67.720001 | 0 | 21 | 3 | 1 | 0 | 0 | 0 | 0 | 0 | 0 |  | NA |  | NA | 2 | 2 | 2 | 0 | 0 | 1 |  | 3 | 25 | 0 | 7 | 0 |  |
| 2730 |  | 23.824 | 3.501 | 14.695265 | 85.304733 | 0 | 10.004107 | 0 | 0.86113787 | 4 | 83.292099 | 21.824444 | 72.449 | 0 | 0 | 3 | 1 |  | | | | | | 117.0926161 | 1.497758579 | 77.00501697 | -2.845687774 | 2 | 2 |  | 0 | 0 | 0 | 1 | 1 |  | | | | |
| 2730 | 1 | 16.832001 | 1.7 | 10.09981 | 89.900192 | 0 | 11.353868 | 0 | 0.86113787 | 4 | 83.292099 | 24.722221 | 72.550003 | 0 | 19 | 3 | 1 | 1 | 1 | 1 | 1 | 1 |  | | NA |  | NA | 2 | 2 | 2 | 0 | 0 | 0 |  | 1 | 27 | 0 | 7 | 0 |  |
| 2730 | 1 | 19.488001 | 2.0999999 | 10.775861 | 89.224136 | 0 | 11.394935 | 0 | 0.86113787 | 4 | 83.292099 | 24.194447 | 75.849998 | 0 | 20 | 3 | 1 | 1 | 0 | 1 | 1 | 1 |  | 110.4135918 | 0.915400388 | 86.42161339 | -1.959025472 | 2 | 2 | 2 | 0 | 0 | 0 | 1 | 1 | 27 | 0 |  | | |
| 2730 | 0 | 24.937 | 2.77 | 11.107992 | 88.892006 | 0 | 11.446954 | 0 | 0.86113787 | 4 | 83.292099 | 23.750555 | 73.699997 | 0 | 21 | 3 | 1 | 0 | 0 | 0 | 0 | 0 |  | | NA |  | NA | 2 | 2 | 2 | 0 | 0 | 0 |  | 1 | 27 | 0 | 7 | 0 |  |
| 2731 |  | 23.088 | 3.057 | 13.240644 | 86.759354 | 1 | 14.858316 | 1 | 1.3507731 | 3 | 84.794785 | 21.937778 | 70.528 | 0 | 0 | 3 | 1 |  | | | | | | 123.9532176 | 2.178542421 | 73.12758968 | -3.266017149 | 2 | 2 |  | 0 | 0 | 1 | 1 | 1 |  | | | | |
| 2731 | 0 | 18.355 | 1.86 | 10.133478 | 89.866524 | 1 | 16.093086 | 1 | 1.3507731 | 3 | 84.794785 | 25.662779 | 68.980003 | 0 | 19 | 3 | 1 | 0 | 0 | 0 | 0 | 0 | 0 |  | NA |  | NA | 2 | 2 | 2 | 0 | 0 | 1 |  | 1 | 24 | 0 | 7 | 0 |  |
| 2731 | 0 | 20.931999 | 2.1800001 | 10.414677 | 89.58532 | 1 | 16.131416 | 1 | 1.3507731 | 3 | 84.794785 | 25.052223 | 72.120003 | 0 | 20 | 3 | 1 | 0 | 0 | 0 | 0 | 0 | 0 | 119.4838446 | 1.769904318 | 73.98038437 | -3.125954796 | 2 | 2 | 2 | 0 | 0 | 1 | 1 | 1 | 24 | 0 |  | | |
| 2731 | 0 | 24.047001 | 2.6300001 | 10.936915 | 89.063087 | 1 | 16.19165 | 1 | 1.3507731 | 3 | 84.794785 | 24.711113 | 70.830002 | 0 | 21 | 3 | 1 | 0 | 0 | 0 | 0 | 0 | 0 |  | NA |  | NA | 2 | 2 | 2 | 0 | 0 | 1 |  | 1 | 24 | 0 | 7 | 0 |  |
| 2732 |  | 23.012 | 3.27 | 14.209977 | 85.790024 | 0 | 9.5879536 | 0 | -3.3403184 | 3 | 104.95327 | 22.609444 | 71.112 | 0 | 0 | 3 | 1 |  | | | | | | 112.9453206 | 1.134575962 | 99.3451966 | -0.1174371 | 2 | 2 |  | 0 | 0 | 0 | 0 | 3 |  | | | | |
| 2732 | 1 | 16.634001 | 1.6900001 | 10.159913 | 89.840088 | 0 | 10.918549 | 0 | -3.3403184 | 3 | 104.95327 | 24.678886 | 72.599998 | 0 | 19 | 3 | 1 | 1 | 0 | 1 | 0 | 0 |  | | NA |  | NA | 2 | 2 | 2 | 0 | 0 | 0 |  | 3 | 14 | 1 | 5 | 1 |  |
| 2732 | 0 | 18.955999 | 2.0699999 | 10.920026 | 89.079971 | 0 | 10.956879 | 0 | -3.3403184 | 3 | 104.95327 | 24.266111 | 75.82 | 0 | 20 | 3 | 1 | 0 | 0 | 0 | 0 | 0 |  | 113.7930513 | 1.209823257 | 108.3898683 | 1.701341528 | 2 | 2 | 2 | 0 | 0 | 0 | 0 | 3 | 27 | 0 |  | | |
| 2732 | 0 | 25.150999 | 2.8 | 11.132758 | 88.867241 | 0 | 10.992471 | 0 | -3.3403184 | 3 | 104.95327 | 23.888334 | 73.400002 | 0 | 21 | 3 | 1 | 0 | 0 | 0 | 0 | 0 |  | | NA |  | NA | 2 | 2 | 2 | 0 | 0 | 0 |  | 3 | 27 | 0 | 7 | 0 |  |
| 2733 |  | 22.955 | 2.922 | 12.729253 | 87.270744 | 1 | 13.639973 | 1 | 1.0322278 | 4 | 102.96753 | 20.665001 | 72.84 | 0 | 0 | 3 | 0 |  | | | | | | 124.2245056 | 2.208031907 | 88.65146834 | -1.616268337 | 2 | 2 |  | 0 | 0 | 1 | 0 | 1 |  | | | | |
| 2733 | 0 | 18.523001 | 2 | 10.797386 | 89.202614 | 1 | 15.00616 | 1 | 1.0322278 | 4 | 102.96753 | 25.453335 | 70.400002 | 0 | 19 | 3 | 0 | 0 | 0 | 0 | 0 | 0 | 0 |  | NA |  | NA | 2 | 2 | 2 | 0 | 0 | 1 |  | 1 | 15 | 1 | 3.7 | 1 |  |
| 2733 | 0 | 21.799 | 2.3699999 | 10.872058 | 89.127945 | 1 | 15.04449 | 1 | 1.0322278 | 4 | 102.96753 | 25.259445 | 71.970001 | 0 | 20 | 3 | 0 | 0 | 0 | 0 | 0 | 0 | 0 | 123.7822673 | 2.160689855 | 92.35814858 | -1.086755726 | 2 | 2 | 2 | 0 | 0 | 1 | 0 | 1 | 25 | 0 |  | | |
| 2733 | 0 | 23.889999 | 2.55 | 10.673923 | 89.32608 | 1 | 15.08282 | 1 | 1.0322278 | 4 | 102.96753 | 24.184998 | 70.699997 | 0 | 21 | 3 | 0 | 0 | 0 | 0 | 0 | 0 | 0 |  | NA |  | NA | 2 | 2 | 2 | 0 | 0 | 1 |  | 1 | 25 | 0 | 7 | 0 |  |
| 2736 |  | 23.526 | 3.308 | 14.061039 | 85.938965 | 1 | 13.221082 | 0 | 0.13273184 | 3 | 97.301178 | 24.753889 | 63.642 | 0 | 0 | 3 |  | | | | | | | 105.4866417 | 0.479794843 | 98.67346429 | -0.228262204 | 2 | 2 |  | 0 | 0 | 0 | 0 | 4 |  | | | | |
| 2736 | 0 | 17.143 | 1.77 | 10.324914 | 89.675087 | 1 | 14.529774 | 0 | 0.13273184 | 3 | 97.301178 | 26.259443 | 66.330002 | 0 | 19 | 3 |  | 0 | 0 | 0 | 0 | 0 | 0 |  | NA |  | NA | 2 | 2 | 2 | 0 | 0 | 0 |  | 4 | 25 | 0 | 7 | 0 |  |
| 2736 | 0 | 19.08 | 2.1199999 | 11.111111 | 88.888893 | 1 | 14.568104 | 0 | 0.13273184 | 3 | 97.301178 | 26.305 | 68.18 | 0 | 20 | 3 |  | 0 | 0 | 0 | 0 | 0 | 0 | 111.4087325 | 0.983029145 | 94.60397863 | -0.862959979 | 2 | 2 | 2 | 0 | 0 | 0 | 0 | 4 | 24 | 0 |  | | |
| 2736 | 0 | 25.521 | 2.8800001 | 11.284824 | 88.715172 | 1 | 14.620123 | 0 | 0.13273184 | 3 | 97.301178 | 25.619444 | 66.720001 | 0 | 21 | 3 |  | 0 | 0 | 0 | 0 | 0 | 0 |  | NA |  | NA | 2 | 2 | 2 | 0 | 0 | 0 |  | 4 | 25 | 0 | 7 | 0 |  |
| 2737 |  | 24.158 | 3.384 | 14.007782 | 85.992218 | 1 | 14.480493 | 1 | -0.1577317 | 4 | 84.412735 | 23.216667 | 67.23 | 0 | 0 | 3 | 1 |  | | | | | | 91.52995508 | -0.777135676 | 97.30936575 | -0.406450383 | 2 | 2 |  | 1 | 0 | 0 | 0 | 3 |  | | | | |
| 2737 | 0 | 17.562 | 1.8 | 10.249402 | 89.750595 | 1 | 15.761807 | 1 | -0.1577317 | 4 | 84.412735 | 25.933889 | 67.639999 | 0 | 19 | 3 | 1 | 0 | 0 | 0 | 0 | 0 | 0 |  | NA |  | NA | 2 | 2 | 2 | 1 | 0 | 0 |  | 3 | 24 | 0 | 7 | 0 |  |
| 2737 | 0 | 19.933001 | 2.1700001 | 10.88647 | 89.113533 | 1 | 15.791924 | 1 | -0.1577317 | 4 | 84.412735 | 25.788332 | 70.099998 | 0 | 20 | 3 | 1 | 0 | 0 | 0 | 0 | 0 | 0 | 90.73870695 | -0.847777266 | 102.1452108 | 0.327931472 | 2 | 2 | 2 | 1 | 0 | 0 | 0 | 3 | 24 | 0 |  | | |
| 2737 | 0 | 24.788 | 2.76 | 11.134419 | 88.865578 | 1 | 15.852156 | 1 | -0.1577317 | 4 | 84.412735 | 25.285 | 68.5 | 0 | 21 | 3 | 1 | 0 | 0 | 0 | 0 | 0 | 0 |  | NA |  | NA | 2 | 2 | 2 | 1 | 0 | 0 |  | 3 | 24 | 0 | 7 | 0 |  |
| 2741 |  | 23.104 | 2.97 | 12.854917 | 87.145081 | 1 | 13.941136 | 0 | 0.17228778 | 2 | 90.253601 | 21.524445 | 71.125 | 0 | 0 | 2 | 0 |  | | | | | | 107.2199121 | 0.627286546 | 89.97511307 | -1.508800159 | 2 | 2 |  | 1 | 0 | 0 | 0 | 2 |  | | | | |
| 2741 | 0 | 19.450001 | 2.04 | 10.488431 | 89.511566 | 1 | 15.307323 | 1 | 0.17228778 | 2 | 90.253601 | 25.52722 | 70.169998 | 0 | 19 | 2 | 0 | 0 | 0 | 0 | 0 | 0 | 0 |  | NA |  | NA | 2 | 2 | 2 | 1 | 0 | 0 |  | 2 | 24 | 0 | 7 | 0 |  |
| 2741 | 0 | 25.42 | 2.6800001 | 10.54288 | 89.457123 | 1 | 15.383984 | 1 | 0.17228778 | 2 | 90.253601 | 24.129444 | 70.220001 | 0 | 21 | 2 | 0 | 0 | 0 | 0 | 0 | 0 | 0 |  | NA |  | NA | 2 | 2 | 2 | 1 | 0 | 0 |  | 2 | 25 | 0 | 7 | 0 |  |
| 2744 |  | 23.109 | 2.967 | 12.839153 | 87.160843 | 0 | 10.214921 | 1 | 1.7815342 | 2 | 82.134293 | 21.402222 | 71.054 | 0 | 0 | 3 | 0 |  | | | | | | 94.48043688 | -0.501485953 | 92.9533574 | -1.065660048 | 2 | 2 |  | 1 | 0 | 0 | 1 | 3 |  | | | | |
| 2744 | 0 | 19.738001 | 2.01 | 10.183402 | 89.816597 | 0 | 11.581109 | 1 | 1.7815342 | 2 | 82.134293 | 25.709444 | 69.010002 | 0 | 19 | 3 | 0 | 0 | 0 | 0 | 0 | 0 |  | | NA |  | NA | 2 | 2 | 2 | 1 | 0 | 0 |  | 3 | 27 | 0 | 7 | 0 |  |
| 2744 | 0 | 22.566 | 2.48 | 10.989985 | 89.010017 | 0 | 11.613963 | 1 | 1.7815342 | 2 | 82.134293 | 25.458887 | 70.519997 | 0 | 20 | 3 | 0 | 0 | 0 | 0 | 0 | 0 |  | 97.37838165 | -0.240507556 | 95.99438514 | -0.625755516 | 2 | 2 | 2 | 1 | 0 | 0 | 0 | 3 | 25 | 0 |  | | |
| 2744 | 1 | 25.049999 | 2.6500001 | 10.578843 | 89.421158 | 0 | 11.633128 | 1 | 1.7815342 | 2 | 82.134293 | 24.406111 | 69.489998 | 0 | 21 | 3 | 0 | 1 | 0 | 1 | 0 | 0 |  | | NA |  | NA | 2 | 2 | 2 | 1 | 0 | 0 |  | 3 | 27 | 0 | 7 | 0 |  |
| 2745 |  | 23.642 | 3.126 | 13.222232 | 86.777771 | 1 | 13.212868 | 1 | -1.5018775 | 2 | 113.81374 | 21.368334 | 71.375 | 0 | 0 | 3 | 1 |  | | | | | | 128.0914842 | 2.560034814 | 95.16431146 | -0.734685936 |  | | | | | 0 | 0 | 2 |  | | | | |
| 2745 | 0 | 19.021 | 1.91 | 10.041533 | 89.958466 | 1 | 14.513347 | 1 | -1.5018775 | 2 | 113.81374 | 25.791113 | 68.419998 | 0 | 19 | 3 | 1 | 0 | 0 | 0 | 0 | 0 | 0 |  | NA |  | NA |  | | 2 |  | | 0 |  | 2 | 24 | 0 | 7 | 0 |  |
| 2745 | 1 | 21.745001 | 2.24 | 10.301218 | 89.698784 | 1 | 14.557153 | 1 | -1.5018775 | 2 | 113.81374 | 25.286667 | 71.419998 | 0 | 20 | 3 | 1 | 1 | 1 | 0 | 0 | 1 | 0 | 121.5583665 | 1.962832384 | 98.1119345 | -0.286549723 |  | | 2 |  | | 0 | 1 | 2 | 23 | 0 |  | | |
| 2745 | 0 | 24.01 | 2.6300001 | 10.95377 | 89.046234 | 1 | 14.61191 | 1 | -1.5018775 | 2 | 113.81374 | 24.721666 | 70.389999 | 0 | 21 | 3 | 1 | 0 | 0 | 0 | 0 | 0 | 0 |  | NA |  | NA |  | | 2 |  | | 0 |  | 2 | 24 | 0 | 7 | 0 |  |
| 2746 |  | 23.331 | 3.077 | 13.188461 | 86.811539 | 0 | 11.655031 | 1 | 0.53340524 | 2 | 75.327072 | 21.149445 | 71.839 | 0 | 0 | 3 | 1 |  | | | | | | 87.85099584 | -1.117587016 | 91.46071293 | -1.278227195 | 2 | 2 |  | 1 | 0 | 0 | 1 | 2 |  | | | | |
| 2746 | 0 | 18.801001 | 1.9 | 10.105844 | 89.894157 | 1 | 12.969199 | 1 | 0.53340524 | 2 | 75.327072 | 25.648888 | 68.970001 | 0 | 19 | 3 | 1 | 0 | 0 | 0 | 0 | 0 | 0 |  | NA |  | NA | 2 | 2 | 2 | 1 | 0 | 0 |  | 2 | 24 | 0 | 7 | 0 |  |
| 2746 | 1 | 21.399 | 2.22 | 10.374316 | 89.625687 | 1 | 13.004791 | 1 | 0.53340524 | 2 | 75.327072 | 24.971664 | 72.32 | 0 | 20 | 3 | 1 | 1 | 0 | 0 | 0 | 1 | 0 | 75.64777895 | -2.249949895 | 98.46131375 | -0.241116785 | 2 | 2 | 2 | 1 | 0 | 0 | 0 | 2 | 24 | 0 |  | | |
| 2746 | 0 | 23.437 | 2.5699999 | 10.965567 | 89.034431 | 1 | 13.045859 | 1 | 0.53340524 | 2 | 75.327072 | 24.912779 | 70.989998 | 0 | 21 | 3 | 1 | 0 | 0 | 0 | 0 | 0 | 0 |  | NA |  | NA | 2 | 2 | 2 | 1 | 0 | 0 |  | 2 | 24 | 0 | 7 | 0 |  |
| 2747 |  | 23.41 | 3.037 | 12.973088 | 87.026909 | 0 | 11.786448 | 0 | 0.13483685 | 4 | 121.3045 | 20.13611 | 73.522 | 0 | 0 | 3 | 0 |  | | | | | | 126.9033031 | 2.334780132 | 102.0107696 | 0.369454813 | 2 | 2 |  | 1 | 0 | 1 | 0 | 3 |  | | | | |
| 2747 | 0 | 18.403999 | 2.01 | 10.921539 | 89.078461 | 1 | 13.122519 | 0 | 0.13483685 | 4 | 121.3045 | 25.557777 | 70.239998 | 0 | 19 | 3 | 0 | 0 | 0 | 0 | 0 | 0 | 0 |  | NA |  | NA | 2 | 2 | 2 | 1 | 0 | 1 |  | 3 | 24 | 0 | 7 | 0 |  |
| 2747 | 1 | 21.834 | 2.3800001 | 10.900431 | 89.099571 | 1 | 13.160849 | 0 | 0.13483685 | 4 | 121.3045 | 25.279999 | 71.290001 | 0 | 20 | 3 | 0 | 1 | 1 | 1 | 1 | 1 | 1 | 123.1771954 | 1.998975342 | 97.47344526 | -0.427361128 | 2 | 2 | 2 | 1 | 0 | 1 | 0 | 3 | 20 | 0 |  | | |
| 2747 | 0 | 24.054001 | 2.5799999 | 10.725866 | 89.274132 | 1 | 13.199179 | 0 | 0.13483685 | 4 | 121.3045 | 24.246111 | 70.309998 | 0 | 21 | 3 | 0 | 0 | 0 | 0 | 0 | 0 | 0 |  | NA |  | NA | 2 | 2 | 2 | 1 | 0 | 1 |  | 3 | 24 | 0 | 7 | 0 |  |
| 2748 |  | 23.493 | 2.925 | 12.450517 | 87.549484 | 1 | 13.24846 | 1 | -3.5650916 | 2 | 110.3149 | 21.327223 | 71.208 | 0 | 0 | 3 | 1 |  | | | | | | 116.5996973 | 1.516644287 | 101.3075611 | 0.209361082 | 2 | 2 |  | 0 | 0 | 1 | 0 | 2 |  | | | | |
| 2748 | 1 | 20.782 | 2.1099999 | 10.153016 | 89.846985 | 1 | 14.609172 | 1 | -3.5650916 | 2 | 110.3149 | 25.902224 | 68.18 | 0 | 19 | 3 | 1 | 0 | 0 | 0 | 1 | 0 | 0 |  | NA |  | NA | 2 | 2 | 1 | 0 | 0 | 1 |  | 2 | 24 | 0 | 7 | 0 |  |
| 2748 | 0 | 24.049999 | 2.5599999 | 10.64449 | 89.355507 | 1 | 14.647502 | 1 | -3.5650916 | 2 | 110.3149 | 25.664444 | 69.82 | 0 | 20 | 3 | 1 | 0 | 0 | 0 | 0 | 0 | 0 | 117.445535 | 1.589614011 | 103.6087508 | 0.572086846 | 2 | 2 | 2 | 0 | 0 | 1 | 0 | 2 | 24 | 0 |  | | |
| 2748 | 1 | 25.750999 | 2.5899999 | 10.057862 | 89.942139 | 1 | 14.685832 | 1 | -3.5650916 | 2 | 110.3149 | 24.512779 | 68.800003 | 0 | 21 | 3 | 1 | 1 | 1 | 1 | 1 | 1 | 0 |  | NA |  | NA | 2 | 2 | 2 | 0 | 0 | 1 |  | 2 | 20 | 0 | 6.8000002 | 1 |  |
| 2752 |  | 22.938 | 3.061 | 13.344668 | 86.655334 | 0 | 9.8151951 | 1 | 1.5292045 | 4 | 123.1185 | 23.596666 | 66.686 | 0 | 0 | 3 | 1 |  | | | | | | 131.4978407 | 2.82046498 | 99.59354733 | -0.065476931 | 2 | 2 |  | 0 | 0 | 0 | 0 |  | | | | | |
| 2752 | 0 | 17.299999 | 1.85 | 10.693643 | 89.306358 | 0 | 11.134839 | 1 | 1.5292045 | 4 | 123.1185 | 25.532223 | 70.330002 | 0 | 19 | 3 | 1 | 0 | 0 | 0 | 0 | 0 |  | | NA |  | NA | 2 | 2 | 2 | 0 | 0 | 0 |  | | 25 | 0 | 7 | 0 |  |
| 2752 | 0 | 20.469999 | 2.3 | 11.235955 | 88.764046 | 0 | 11.173169 | 1 | 1.5292045 | 4 | 123.1185 | 25.48222 | 72.339996 | 0 | 20 | 3 | 1 | 0 | 0 | 0 | 0 | 0 |  | 121.85675 | 1.989599352 | 104.2897065 | 0.725460059 | 2 | 2 | 2 | 0 | 0 | 0 | 0 |  | 26 | 0 |  | | |
| 2752 | 1 | 22.155001 | 2.52 | 11.374407 | 88.625595 | 0 | 11.216974 | 1 | 1.5292045 | 4 | 123.1185 | 24.457224 | 71.800003 | 0 | 21 | 3 | 1 | 1 | 0 | 1 | 1 | 1 |  | | NA |  | NA | 2 | 2 | 2 | 0 | 0 | 0 |  | | 27 | 0 | 6.9000001 | 1 |  |
| 2753 |  | 22.841 | 3.041 | 13.313778 | 86.686226 | 1 | 12.621492 | 0 | -0.86958724 | 2 | 91.402206 | 20.85111 | 71.551 | 0 | 0 | 3 | 1 |  | | | | | | 115.2045773 | 1.324413381 | 88.48186216 | -1.709382048 | 2 | 2 |  | 0 | 0 | 0 | 0 | 1 |  | | | | |
| 2753 | 1 | 18.629 | 1.9299999 | 10.36019 | 89.639809 | 1 | 13.913757 | 0 | -0.86958724 | 2 | 91.402206 | 25.360003 | 69.970001 | 0 | 19 | 3 | 1 | 1 | 1 | 1 | 1 | 0 | 1 |  | NA |  | NA | 2 | 2 | 2 | 0 | 0 | 0 |  | 1 | 24 | 0 | 7 | 0 |  |
| 2753 | 1 | 21.622999 | 2.4100001 | 11.14554 | 88.854462 | 1 | 13.952087 | 0 | -0.86958724 | 2 | 91.402206 | 25.243891 | 71.870003 | 0 | 20 | 3 | 1 | 1 | 1 | 1 | 1 | 1 | 1 | 93.1093682 | -0.606108667 | 103.7950662 | 0.697433826 | 2 | 2 | 2 | 0 | 0 | 0 | 0 | 1 | 23 | 0 |  | | |
| 2753 | 0 | 25.118 | 2.8299999 | 11.26682 | 88.733177 | 1 | 13.993155 | 0 | -0.86958724 | 2 | 91.402206 | 23.992775 | 70.800003 | 0 | 21 | 3 | 1 | 0 | 0 | 0 | 0 | 0 | 0 |  | NA |  | NA | 2 | 2 | 2 | 0 | 0 | 0 |  | 1 | 23 | 0 | 7 | 0 |  |
| 2754 |  | 26.07 | 3.056 | 11.722286 | 88.277718 | 0 | 10.699521 | 1 | 1.5357243 | 3 | 95.953461 | 22.688889 | 70.933 | 0 | 0 | 3 | 1 |  | | | | | | 95.18058243 | -0.440064686 | 107.600463 | 1.327871542 | 2 | 2 |  | 0 |  | 1 | 0 | 3 |  | | | | |
| 2754 | 0 | 19.374001 | 2.21 | 11.40704 | 88.592957 | 0 | 11.775496 | 1 | 1.5357243 | 3 | 95.953461 | 25.140554 | 70.75 | 0 | 19 | 3 | 1 | 0 | 0 | 0 | 0 | 0 |  | | NA |  | NA | 2 | 2 | 2 | 0 |  | 1 |  | 3 | 27 | 0 | 7 | 0 |  |
| 2754 | 0 | 23.042999 | 2.74 | 11.890813 | 88.109184 | 0 | 11.830254 | 1 | 1.5357243 | 3 | 95.953461 | 24.417219 | 74.370003 | 0 | 20 | 3 | 1 | 0 | 0 | 0 | 0 | 0 |  | 115.8924327 | 1.452095607 | 104.8797246 | 0.824955694 | 2 | 2 | 2 | 0 |  | 1 | 0 | 3 | 27 | 0 |  | | |
| 2754 | 0 | 26.621 | 3.29 | 12.358665 | 87.641335 | 0 | 11.852156 | 1 | 1.5357243 | 3 | 95.953461 | 23.48 | 72.519997 | 0 | 21 | 3 | 1 | 0 | 0 | 0 | 0 | 0 |  | | NA |  | NA | 2 | 2 | 2 | 0 |  | 1 |  | 3 | 27 | 0 | 7 | 0 |  |
| 2755 |  | 23.583 | 3.232 | 13.704787 | 86.295212 | 0 | 10.562629 | 1 | 0.66368955 | 3 | 111.65145 | 21.766666 | 73.718 | 0 | 0 | 3 | 1 |  | | | | | | 126.1338246 | 2.365418084 | 94.41810898 | -0.858980743 | 1 | 2 |  | 0 | 0 | 1 | 0 | 3 |  | | | | |
| 2755 | 0 | 18.174999 | 1.98 | 10.894086 | 89.105911 | 0 | 11.865846 | 1 | 0.66368955 | 3 | 111.65145 | 24.748331 | 73.730003 | 0 | 19 | 3 | 1 | 0 | 0 | 0 | 0 | 0 |  | | NA |  | NA | 1 | 2 | 2 | 0 | 0 | 1 |  | 3 | 26 | 0 | 7 | 0 |  |
| 2755 | 0 | 21.186001 | 2.47 | 11.658642 | 88.341354 | 0 | 11.904175 | 1 | 0.66368955 | 3 | 111.65145 | 24.407221 | 76.709999 | 0 | 20 | 3 | 1 | 0 | 0 | 0 | 0 | 0 |  | 117.7375418 | 1.620263484 | 98.84866818 | -0.184179608 | 1 | 2 | 2 | 0 | 0 | 1 | 0 | 3 | 26 | 0 |  | | |
| 2755 | 0 | 23.105 | 2.6700001 | 11.555941 | 88.444061 | 0 | 11.942505 | 1 | 0.66368955 | 3 | 111.65145 | 23.625557 | 74.93 | 0 | 21 | 3 | 1 | 0 | 0 | 0 | 0 | 0 |  | | NA |  | NA | 1 | 2 | 2 | 0 | 0 | 1 |  | 3 | 27 | 0 | 7 | 0 |  |
| 2758 |  | 23.678 | 3.244 | 13.700481 | 86.299515 | 1 | 13.752225 | 1 | -1.3181577 | 2 | 87.490829 | 23.794445 | 65.408 | 0 | 0 | 3 | 0 |  | | | | | | 91.64816358 | -0.767242938 | 102.7466303 | 0.44066538 | 2 | 2 |  | 1 | 0 | 0 | 0 | 4 |  | | | | |
| 2758 | 0 | 16.839001 | 1.8099999 | 10.748856 | 89.251144 | 1 | 15.022587 | 1 | -1.3181577 | 2 | 87.490829 | 25.589998 | 70.139999 | 0 | 19 | 3 | 0 | 0 | 0 | 0 | 0 | 0 | 0 |  | NA |  | NA | 2 | 2 | 2 | 1 | 0 | 0 |  | 4 | 24 | 0 | 7 | 0 |  |
| 2758 | 0 | 19.974001 | 2.25 | 11.264644 | 88.735359 | 1 | 15.074607 | 1 | -1.3181577 | 2 | 87.490829 | 25.533888 | 71.760002 | 0 | 20 | 3 | 0 | 0 | 0 | 0 | 0 | 0 | 0 | 94.86547505 | -0.469608607 | 88.7032838 | -1.551136068 | 2 | 2 | 2 | 1 | 0 | 0 | 0 | 4 | 24 | 0 |  | | |
| 2758 | 0 | 20.507999 | 2.3599999 | 11.507705 | 88.492294 | 1 | 15.093771 | 1 | -1.3181577 | 2 | 87.490829 | 24.176666 | 70.940002 | 0 | 21 | 3 | 0 | 0 | 0 | 0 | 0 | 0 | 0 |  | NA |  | NA | 2 | 2 | 2 | 1 | 0 | 0 |  | 4 | 20 | 0 | 6.0999999 | 1 |  |
| 2762 |  | 25.304 | 3.373 | 13.329908 | 86.67009 | 1 | 14.691307 | 1 | -0.80004835 | 2 | 101.71766 | 22.817223 | 68.117 | 0 | 0 | 3 | 1 |  | | | | | | 120.9807855 | 1.910055557 | 90.09848129 | -1.401656882 | 2 | 2 |  | 0 | 0 | 0 | 1 | 2 |  | | | | |
| 2762 | 0 | 17.936001 | 2 | 11.150758 | 88.849243 | 1 | 15.895962 | 1 | -0.80004835 | 2 | 101.71766 | 25.40889 | 70.889999 | 0 | 19 | 3 | 1 | 0 | 0 | 0 | 0 | 0 | 0 |  | NA |  | NA | 2 | 2 | 2 | 0 | 0 | 0 |  | 2 | 24 | 0 | 7 | 0 |  |
| 2762 | 0 | 20.972 | 2.4100001 | 11.491513 | 88.508484 | 1 | 15.934292 | 1 | -0.80004835 | 2 | 101.71766 | 25.235558 | 72.129997 | 0 | 20 | 3 | 1 | 0 | 0 | 0 | 0 | 0 | 0 | 116.850688 | 1.531744681 | 98.98283444 | -0.150936612 | 2 | 2 | 2 | 0 | 0 | 0 | 0 | 2 | 24 | 0 |  | | |
| 2762 | 0 | 24.047001 | 2.8 | 11.643864 | 88.35614 | 1 | 15.969884 | 1 | -0.80004835 | 2 | 101.71766 | 23.757778 | 71.889999 | 0 | 21 | 3 | 1 | 0 | 0 | 0 | 0 | 0 | 0 |  | NA |  | NA | 2 | 2 | 2 | 0 | 0 | 0 |  | 2 | 25 | 0 | 7 | 0 |  |
| 2767 |  | 23.11 | 3.19 | 13.803548 | 86.196449 | 1 | 14.652977 | 1 | -1.0104688 | 2 | 95.282768 | 15.71 | 76.993 | 0 | 0 | 3 | 1 |  | | | | | | 95.16069769 | -0.4434138 | 107.438569 | 1.216312183 | 2 | 2 |  | 0 | 0 | 1 | 0 | 2 |  | | | | |
| 2767 | 0 | 17.278 | 1.71 | 9.8969784 | 90.10302 | 1 | 15.953457 | 1 | -1.0104688 | 2 | 95.282768 | 25.502777 | 68.260002 | 0 | 19 | 3 | 1 | 0 | 0 | 0 | 0 | 0 | 0 |  | NA |  | NA | 2 | 2 | 2 | 0 | 0 | 1 |  | 2 | 24 | 0 | 7 | 0 |  |
| 2767 | 0 | 20.007999 | 2.0799999 | 10.395842 | 89.604156 | 1 | 15.994524 | 1 | -1.0104688 | 2 | 95.282768 | 24.661667 | 72.599998 | 0 | 20 | 3 | 1 | 0 | 0 | 0 | 0 | 0 | 0 | 93.95228329 | -0.552694052 | 109.1891937 | 1.46933746 | 2 | 2 | 2 | 0 | 0 | 1 | 0 | 2 | 25 | 0 |  | | |
| 2767 | 0 | 24.115 | 2.6300001 | 10.906075 | 89.093925 | 1 | 16.030117 | 1 | -1.0104688 | 2 | 95.282768 | 24.238888 | 70.870003 | 0 | 21 | 3 | 1 | 0 | 0 | 0 | 0 | 0 | 0 |  | NA |  | NA | 2 | 2 | 2 | 0 | 0 | 1 |  | 2 | 25 | 0 | 7 | 0 |  |
| 2768 |  | 24.007 | 3.427 | 14.275003 | 85.724998 | 0 | 10.023272 | 1 | -0.17667681 | 3 | 84.227768 | 15.158889 | 78.051 | 0 | 0 | 3 | 1 |  | | | | | | 89.26070026 | -0.974642759 | 100.3308524 | 0.053789101 | 2 | 2 |  | 0 | 0 | 0 | 0 | 3 |  | | | | |
| 2768 | 0 | 17.386999 | 1.73 | 9.9499636 | 90.050034 | 0 | 11.266256 | 1 | -0.17667681 | 3 | 84.227768 | 25.592224 | 68.080002 | 0 | 19 | 3 | 1 | 0 | 0 | 0 | 0 | 0 |  | | NA |  | NA | 2 | 2 | 2 | 0 | 0 | 0 |  | 3 | 26 | 0 | 7 | 0 |  |
| 2768 | 0 | 19.775999 | 2.0599999 | 10.416667 | 89.583336 | 0 | 11.307323 | 1 | -0.17667681 | 3 | 84.227768 | 24.647776 | 72.489998 | 0 | 20 | 3 | 1 | 0 | 0 | 0 | 0 | 0 |  | 82.53832475 | -1.609396411 | 110.5766437 | 1.881121269 | 2 | 2 | 2 | 0 | 0 | 0 | 0 | 3 | 26 | 0 |  | | |
| 2768 | 0 | 23.993999 | 2.55 | 10.627657 | 89.372345 | 0 | 11.353868 | 1 | -0.17667681 | 3 | 84.227768 | 24.140556 | 71 | 0 | 21 | 3 | 1 | 0 | 0 | 0 | 0 | 0 |  | | NA |  | NA | 2 | 2 | 2 | 0 | 0 | 0 |  | 3 | 27 | 0 | 7 | 0 |  |
| 2769 |  | 22.954 | 3.182 | 13.862508 | 86.137489 | 1 | 12.665298 | 1 | 1.0060798 | 3 | 111.73213 | 16.814444 | 74.423 | 0 | 0 | 3 | 1 |  | | | | | | 124.8847435 | 2.270452592 | 95.62382999 | -0.673566293 | 2 | 2 |  | 0 | 0 | 1 | 0 | 2 |  | | | | |
| 2769 | 0 | 18.308001 | 1.87 | 10.214113 | 89.785889 | 1 | 14.020534 | 1 | 1.0060798 | 3 | 111.73213 | 25.753889 | 68.330002 | 0 | 19 | 3 | 1 | 0 | 0 | 0 | 0 | 0 | 0 |  | NA |  | NA | 2 | 2 | 2 | 0 | 0 | 1 |  | 2 | 24 | 0 | 7 | 0 |  |
| 2769 | 0 | 23.084 | 2.5599999 | 11.089932 | 88.910065 | 1 | 14.058864 | 1 | 1.0060798 | 3 | 111.73213 | 25.477777 | 68.639999 | 0 | 20 | 3 | 1 | 0 | 0 | 0 | 0 | 0 | 0 | 93.65851538 | -0.581961172 | 95.97094218 | -0.606918569 | 2 | 2 | 2 | 0 | 0 | 1 | 0 | 2 | 24 | 0 |  | | |
| 2769 | 0 | 24.371 | 2.6199999 | 10.750482 | 89.249519 | 1 | 14.097194 | 1 | 1.0060798 | 3 | 111.73213 | 23.686111 | 69.940002 | 0 | 21 | 3 | 1 | 0 | 0 | 0 | 0 | 0 | 0 |  | NA |  | NA | 2 | 2 | 2 | 0 | 0 | 1 |  | 2 | 24 | 0 | 7 | 0 |  |
| 2770 |  | 25.197 | 3.208 | 12.731674 | 87.268326 | 1 | 17.856262 | 1 | -1.1483787 | 2 | 110.20863 | 24.504444 | 71.391 | 0 | 0 | 3 | 0 |  | | | | | | 114.2880064 | 1.297574182 | 103.120824 | 0.468764263 | 2 | 2 |  | 0 | 0 | 0 | 0 | 3 |  | | | | |
| 2770 | 0 | 22.700001 | 2.27 | 10 | 90 | 1 | 19.140314 | 1 | -1.1483787 | 2 | 110.20863 | 26.194445 | 67.620003 | 0 | 19 | 3 | 0 | 0 | 0 | 0 | 0 | 0 | 0 |  | NA |  | NA | 2 | 2 | 2 | 0 | 0 | 0 |  | 3 | 24 | 0 | 7 | 0 |  |
| 2770 | 1 | 23.41 | 2.48 | 10.593763 | 89.406235 | 1 | 19.178644 | 1 | -1.1483787 | 2 | 110.20863 | 26.165001 | 70.199997 | 0 | 20 | 3 | 0 | 1 | 1 | 1 | 1 | 0 | 1 | 109.0466414 | 0.821866751 | 102.1229133 | 0.313373817 | 2 | 2 | 2 | 0 | 0 | 0 | 0 | 3 | 24 | 0 |  | | |
| 2770 | 0 | 25.457001 | 2.72 | 10.684684 | 89.315315 | 1 | 19.225187 | 1 | -1.1483787 | 2 | 110.20863 | 25.816113 | 67.889999 | 0 | 21 | 3 | 0 | 0 | 0 | 0 | 0 | 0 | 0 |  | NA |  | NA | 2 | 2 | 2 | 0 | 0 | 0 |  | 3 | 24 | 0 | 7 | 0 |  |
| 2773 |  | 26.116 | 3.44 | 13.172002 | 86.827995 | 0 | 10.225873 | 1 | 1.5357243 | 3 | 84.916893 | 22.081667 | 71.303 | 0 | 0 | 3 | 1 |  | | | | | | 95.70202002 | -0.390426831 | 94.96722556 | -0.776766456 | 2 | 2 |  | 0 | 0 | 0 | 1 | 3 |  | | | | |
| 2773 | 0 | 18.941 | 1.99 | 10.50631 | 89.49369 | 0 | 11.419576 | 1 | 1.5357243 | 3 | 84.916893 | 25.588888 | 69.809998 | 0 | 19 | 3 | 1 | 0 | 0 | 0 | 0 | 0 |  | | NA |  | NA | 2 | 2 | 2 | 0 | 0 | 0 |  | 3 | 27 | 0 | 7 | 0 |  |
| 2773 | 0 | 21.539 | 2.3800001 | 11.049725 | 88.950279 | 0 | 11.45243 | 1 | 1.5357243 | 3 | 84.916893 | 25.334999 | 72.730003 | 0 | 20 | 3 | 1 | 0 | 0 | 0 | 0 | 0 |  | 86.2050208 | -1.270902023 | 99.33940758 | -0.105803749 | 2 | 2 | 2 | 0 | 0 | 0 | 0 | 3 | 26 | 0 |  | | |
| 2773 | 0 | 22.414 | 2.49 | 11.109128 | 88.890869 | 0 | 11.49076 | 1 | 1.5357243 | 3 | 84.916893 | 25.006113 | 70.760002 | 0 | 21 | 3 | 1 | 0 | 0 | 0 | 0 | 0 |  | | NA |  | NA | 2 | 2 | 2 | 0 | 0 | 0 |  | 3 | 23 | 0 | 6.5 | 1 |  |
| 2774 |  | 22.854 | 3.171 | 13.875032 | 86.124969 | 0 | 11.835729 | 0 | 0.89390159 | 2 | 108.69128 | 16.587778 | 74.183 | 0 | 0 | 3 | 1 |  | | | | | | 112.024501 | 1.055395225 | 102.526045 | 0.467552004 | 2 | 2 |  | 1 | 0 | 0 | 0 | 3 |  | | | | |
| 2774 | 1 | 18.264999 | 1.84 | 10.073913 | 89.926086 | 1 | 13.051334 | 0 | 0.89390159 | 2 | 108.69128 | 25.992224 | 66.879997 | 0 | 19 | 3 | 1 | 1 | 1 | 1 | 1 | 1 | 1 |  | NA |  | NA | 2 | 2 | 1 | 1 | 0 | 0 |  | 3 | 20 | 0 | 6.0999999 | 1 |  |
| 2774 | 0 | 21.899 | 2.4100001 | 11.005069 | 88.994934 | 1 | 13.089664 | 0 | 0.89390159 | 2 | 108.69128 | 25.981665 | 68.339996 | 0 | 20 | 3 | 1 | 0 | 0 | 0 | 0 | 0 | 0 | 106.1621498 | 0.538200157 | 108.3714821 | 1.657493186 | 2 | 2 | 1 | 1 | 0 | 0 | 0 | 3 | 17 | 1 |  | | |
| 2774 | 0 | 24.712 | 2.75 | 11.128197 | 88.871803 | 1 | 13.127995 | 0 | 0.89390159 | 2 | 108.69128 | 23.976665 | 69.099998 | 0 | 21 | 3 | 1 | 0 | 0 | 0 | 0 | 0 | 0 |  | NA |  | NA | 2 | 2 | 2 | 1 | 0 | 0 |  | 3 | 23 | 0 | 7 | 0 |  |
| 2776 |  | 24.386 | 3.302 | 13.540556 | 86.459442 | 1 | 14.455853 | 1 | -2.4526858 | 3 | 118.35538 | 18.248333 | 75.666 | 0 | 0 | 3 | 0 |  | | | | | | 135.0641645 | 3.183888169 | 93.75243478 | -0.916364598 | 2 | 2 |  | 1 | 0 | 0 | 0 | 2 |  | | | | |
| 2776 | 0 | 17.141001 | 1.78 | 10.384458 | 89.61554 | 1 | 15.720739 | 1 | -2.4526858 | 3 | 118.35538 | 26.010555 | 67.980003 | 0 | 19 | 3 | 0 | 0 | 0 | 0 | 0 | 0 | 0 |  | NA |  | NA | 2 | 2 | 2 | 1 | 0 | 0 |  | 2 | 24 | 0 | 7 | 0 |  |
| 2776 | 0 | 20.021999 | 2.1400001 | 10.688244 | 89.311752 | 1 | 15.77002 | 1 | -2.4526858 | 3 | 118.35538 | 25.69611 | 70.589996 | 0 | 20 | 3 | 0 | 0 | 0 | 0 | 0 | 0 | 0 | 117.7569511 | 1.61472823 | 103.0077588 | 0.463907274 | 2 | 2 | 2 | 1 | 0 | 0 | 0 | 2 | 24 | 0 |  | | |
| 2776 | 0 | 22.639999 | 2.48 | 10.954064 | 89.045937 | 1 | 15.808351 | 1 | -2.4526858 | 3 | 118.35538 | 24.772221 | 68.989998 | 0 | 21 | 3 | 0 | 0 | 0 | 0 | 0 | 0 | 0 |  | NA |  | NA | 2 | 2 | 2 | 1 | 0 | 0 |  | 2 | 24 | 0 | 7 | 0 |  |
| 2780 |  | 23.772 | 3.04 | 12.788155 | 87.211845 | 1 | 12.328542 | 1 | 1.2144388 | 2 | 112.13074 | 22.734444 | 68.947 | 0 | 0 | 3 | 1 |  | | | | | | 119.3903902 | 1.771438057 | 100.3453825 | 0.05571583 | 2 | 2 |  | 1 | 0 | 1 | 0 |  | | | | | |
| 2780 | 1 | 16.407 | 1.75 | 10.666179 | 89.333824 | 1 | 13.642711 | 1 | 1.2144388 | 2 | 112.13074 | 24.657225 | 74.959999 | 0 | 19 | 3 | 1 | 1 | 1 | 1 | 1 | 1 | 1 |  | NA |  | NA | 2 | 2 | 2 | 1 | 0 | 1 |  | | 25 | 0 | 7 | 0 |  |
| 2780 | 1 | 20.052999 | 2.1300001 | 10.621853 | 89.378143 | 1 | 13.681041 | 1 | 1.2144388 | 2 | 112.13074 | 24.392776 | 75.239998 | 0 | 20 | 3 | 1 | 1 | 1 | 1 | 1 | 1 | 1 | 113.1557068 | 1.202092247 | 103.3158591 | 0.53530642 | 2 | 2 | 2 | 1 | 0 | 1 | 0 |  | 25 | 0 |  | | |
| 2780 | 1 | 19.782 | 2.0999999 | 10.615711 | 89.384293 | 1 | 13.71937 | 1 | 1.2144388 | 2 | 112.13074 | 23.273888 | 74.260002 | 0 | 21 | 3 | 1 | 1 | 1 | 0 | 1 | 0 | 0 |  | NA |  | NA | 2 | 2 | 2 | 1 | 0 | 1 |  | | 24 | 0 | 7 | 0 |  |
| 2782 |  | 23.593 | 3.198 | 13.554868 | 86.445129 | 1 | 15.296372 | 1 | -3.0572755 | 3 | 112.26388 | 19.039444 | 73.179 | 0 | 0 | 2 | 1 |  | | | | | | 147.7490989 | 4.317712625 | 80.82410659 | -2.480142924 | 2 | 2 |  | 0 | 0 | 0 | 0 | 1 |  | | | | |
| 2782 | 1 | 20.212 | 2.1400001 | 10.58777 | 89.412231 | 1 | 16.681725 | 1 | -3.0572755 | 3 | 112.26388 | 25.555555 | 70.849998 | 0 | 20 | 2 | 1 | 1 | 0 | 0 | 0 | 1 | 0 | 128.9345982 | 2.621068916 | 84.56062149 | -2.017706933 | 2 | 2 | 2 | 0 | 0 | 0 | 1 | 1 | 24 | 0 |  | | |
| 2782 | 1 | 24.604 | 2.7 | 10.973825 | 89.026176 | 1 | 16.720055 | 1 | -3.0572755 | 3 | 112.26388 | 24.257223 | 70.220001 | 0 | 21 | 2 | 1 | 1 | 0 | 1 | 1 | 0 | 0 |  | NA |  | NA | 2 | 2 | 2 | 0 | 0 | 0 |  | 1 | 25 | 0 | 7 | 0 |  |
| 2783 |  | 23.887 | 3.299 | 13.81086 | 86.18914 | 1 | 17.71937 | 0 | 0.97824323 | 2 | 93.282005 | 20.771667 | 72.687 | 0 | 0 | 3 | 1 |  | | | | | | 123.0501833 | 1.937562653 | 80.19975936 | -2.536543234 | 2 | 2 |  | 0 | 0 | 0 | 1 | 1 |  | | | | |
| 2783 | 0 | 17.981001 | 1.8200001 | 10.121796 | 89.878204 | 1 | 18.98152 | 0 | 0.97824323 | 2 | 93.282005 | 25.361668 | 70 | 0 | 19 | 3 | 1 | 0 | 0 | 0 | 0 | 0 | 0 |  | NA |  | NA | 2 | 2 | 2 | 0 | 0 | 0 |  | 1 | 24 | 0 | 7 | 0 |  |
| 2783 | 0 | 20.896999 | 2.22 | 10.623535 | 89.376465 | 1 | 19.01985 | 0 | 0.97824323 | 2 | 93.282005 | 24.791111 | 73.300003 | 0 | 20 | 3 | 1 | 0 | 0 | 0 | 0 | 0 | 0 |  | NA |  | NA | 2 | 2 | 2 | 0 | 0 | 0 |  | 1 | 24 | 0 |  | | |
| 2783 | 0 | 24.000999 | 2.6600001 | 11.082872 | 88.91713 | 1 | 19.077345 | 0 | 0.97824323 | 2 | 93.282005 | 24.382778 | 71.660004 | 0 | 21 | 3 | 1 | 0 | 0 | 0 | 0 | 0 | 0 |  | NA |  | NA | 2 | 2 | 2 | 0 | 0 | 0 |  | 1 | 24 | 0 | 7 | 0 |  |
| 2784 |  | 23.595 | 3.091 | 13.100233 | 86.899765 | 1 | 12.309377 | 0 | 0.19157845 | 2 | 112.99142 | 19.449444 | 74.763 | 0 | 0 | 3 | 1 |  | | | | | | 119.1698445 | 1.668089486 | 101.2403574 | 0.223963555 | 2 | 2 |  | 0 | 0 | 0 | 0 | 3 |  | | | | |
| 2784 | 1 | 17.114 | 1.85 | 10.809863 | 89.19014 | 1 | 13.607119 | 0 | 0.19157845 | 2 | 112.99142 | 25.661112 | 69.360001 | 0 | 19 | 3 | 1 | 1 | 1 | 1 | 0 | 1 | 1 |  | NA |  | NA | 2 | 2 | 2 | 0 | 0 | 0 |  | 3 | 24 | 0 | 7 | 0 |  |
| 2784 | 0 | 20.691 | 2.21 | 10.680972 | 89.319031 | 1 | 13.664614 | 0 | 0.19157845 | 2 | 112.99142 | 25.206112 | 72.32 | 0 | 20 | 3 | 1 | 0 | 0 | 0 | 0 | 0 | 0 | 102.070307 | 0.181002117 | 103.8958833 | 0.719663547 | 2 | 2 | 2 | 0 | 0 | 0 | 0 | 3 | 24 | 0 |  | | |
| 2784 | 0 | 23.011 | 2.52 | 10.951284 | 89.048714 | 1 | 13.702943 | 0 | 0.19157845 | 2 | 112.99142 | 24.27389 | 70.629997 | 0 | 21 | 3 | 1 | 0 | 0 | 0 | 0 | 0 | 0 |  | NA |  | NA | 2 | 2 | 2 | 0 | 0 | 0 |  | 3 | 25 | 0 | 6.6999998 | 1 |  |
| 2787 |  | 23.494 | 2.983 | 12.696858 | 87.303139 | 1 | 12.985626 | 1 | 1.3277028 | 2 | 124.05031 | 20.834444 | 72.534 | 0 | 0 | 3 |  | | | | | | | 128.7695436 | 2.622068642 | 102.9263726 | 0.477472005 | 2 | 2 |  | 0 | 0 | 0 | 0 | 4 |  | | | | |
| 2787 | 0 | 19.726 | 2.03 | 10.290986 | 89.709015 | 1 | 14.308008 | 1 | 1.3277028 | 2 | 124.05031 | 25.745558 | 68.510002 | 0 | 19 | 3 |  | 0 | 0 | 0 | 0 | 0 | 0 |  | NA |  | NA | 2 | 2 | 2 | 0 | 0 | 0 |  | 4 | 24 | 0 | 7 | 0 |  |
| 2787 | 1 | 23.688999 | 2.4300001 | 10.257927 | 89.742073 | 1 | 14.349076 | 1 | 1.3277028 | 2 | 124.05031 | 25.458332 | 71.580002 | 0 | 20 | 3 |  | 1 | 1 | 1 | 1 | 1 | 1 | 126.5102076 | 2.411783399 | 109.2899311 | 1.549729468 | 2 | 2 | 2 | 0 | 0 | 0 | 0 | 4 | 24 | 0 |  | | |
| 2787 | 0 | 23.483999 | 2.55 | 10.858458 | 89.141541 | 1 | 14.398357 | 1 | 1.3277028 | 2 | 124.05031 | 24.582779 | 70.669998 | 0 | 21 | 3 |  | 0 | 0 | 0 | 0 | 0 | 0 |  | NA |  | NA | 2 | 2 | 2 | 0 | 0 | 0 |  | 4 | 25 | 0 | 7 | 0 |  |
| 2788 |  | 23.356 | 2.996 | 12.827538 | 87.172462 | 0 | 11.616701 | 1 | 1.7179354 | 4 | 109.91318 | 20.756666 | 72.949 | 0 | 0 | 3 | 1 |  | | | | | | 117.9554421 | 1.638876781 | 96.78238395 | -0.506348742 | 1 | 2 |  | 0 | 0 | 1 | 0 | 3 |  | | | | |
| 2788 | 0 | 19.179001 | 2.04 | 10.636633 | 89.363365 | 1 | 12.889802 | 1 | 1.7179354 | 4 | 109.91318 | 25.400557 | 70.449997 | 0 | 19 | 3 | 1 | 0 | 0 | 0 | 0 | 0 | 0 |  | NA |  | NA | 1 | 2 | 2 | 0 | 0 | 1 |  | 3 | 24 | 0 | 7 | 0 |  |
| 2788 | 0 | 22.274 | 2.4300001 | 10.909581 | 89.090416 | 1 | 12.928131 | 1 | 1.7179354 | 4 | 109.91318 | 25.211109 | 72.040001 | 0 | 20 | 3 | 1 | 0 | 0 | 0 | 0 | 0 | 0 | 105.3597992 | 0.491187893 | 101.3589559 | 0.21894822 | 1 | 2 | 2 | 0 | 0 | 1 | 0 | 3 | 24 | 0 |  | | |
| 2788 | 1 | 24.398001 | 2.6099999 | 10.697598 | 89.302406 | 1 | 12.966461 | 1 | 1.7179354 | 4 | 109.91318 | 24.084442 | 70.959999 | 0 | 21 | 3 | 1 | 1 | 1 | 1 | 0 | 0 | 1 |  | NA |  | NA | 1 | 2 | 2 | 0 | 0 | 1 |  | 3 | 23 | 0 | 7 | 0 |  |
| 2789 |  | 23.852 | 3.154 | 13.223209 | 86.776787 | 0 | 11.854894 | 1 | -1.3817564 | 2 | 77.534218 | 22.59 | 70.246 | 0 | 0 | 3 | 1 |  | | | | | | 94.29848882 | -0.523806109 | 88.16014479 | -1.716100412 | 2 | 2 |  | 0 | 0 | 0 | 1 | 1 |  | | | | |
| 2789 | 0 | 19.256001 | 2.0799999 | 10.801826 | 89.198174 | 1 | 13.158111 | 1 | -1.3817564 | 2 | 77.534218 | 25.464998 | 70.610001 | 0 | 19 | 3 | 1 | 0 | 0 | 0 | 0 | 0 | 0 |  | NA |  | NA | 2 | 2 | 2 | 0 | 0 | 0 |  | 1 | 24 | 0 | 7 | 0 |  |
| 2789 | 0 | 21.615999 | 2.4100001 | 11.14915 | 88.850853 | 1 | 13.193703 | 1 | -1.3817564 | 2 | 77.534218 | 25.625555 | 71.610001 | 0 | 20 | 3 | 1 | 0 | 0 | 0 | 0 | 0 | 0 | 102.6885495 | 0.246488154 | 92.75296193 | -1.078134874 | 2 | 2 | 2 | 0 | 0 | 0 | 0 | 1 | 24 | 0 |  | | |
| 2789 | 0 | 24.476 | 2.74 | 11.194639 | 88.805359 | 1 | 13.226557 | 1 | -1.3817564 | 2 | 77.534218 | 24.143333 | 71.080002 | 0 | 21 | 3 | 1 | 0 | 0 | 0 | 0 | 0 | 0 |  | NA |  | NA | 2 | 2 | 2 | 0 | 0 | 0 |  | 1 | 25 | 0 | 7 | 0 |  |
| 2790 |  | 23.261 | 2.99 | 12.854134 | 87.145866 | 1 | 15.055442 | 0 | 0.88414335 | 3 | 93.363823 | 19.775 | 75.407 | 0 | 0 | 3 | 1 |  | | | | | | 115.6915233 | 1.344295997 | 86.4834328 | -1.91993248 | 2 | 2 |  | 0 | 0 | 0 | 0 | 1 |  | | | | |
| 2790 | 1 | 17.659 | 2.04 | 11.552183 | 88.447815 | 1 | 16.396988 | 0 | 0.88414335 | 3 | 93.363823 | 24.981668 | 73 | 0 | 19 | 3 | 1 | 1 | 0 | 1 | 1 | 1 | 1 |  | NA |  | NA | 2 | 2 | 2 | 0 | 0 | 0 |  | 1 | 19 | 1 | 5 | 1 |  |
| 2790 | 0 | 20.857 | 2.27 | 10.883636 | 89.116364 | 1 | 16.435318 | 0 | 0.88414335 | 3 | 93.363823 | 24.654444 | 73.779999 | 0 | 20 | 3 | 1 | 0 | 0 | 0 | 0 | 0 | 0 | 100.8548806 | 0.073440713 | 87.17001183 | -1.816013704 | 2 | 2 | 2 | 0 | 0 | 0 | 0 | 1 | 17 | 1 |  | | |
| 2790 | 0 | 22.6 | 2.47 | 10.929204 | 89.070793 | 1 | 16.473648 | 0 | 0.88414335 | 3 | 93.363823 | 23.810556 | 72.760002 | 0 | 21 | 3 | 1 | 0 | 0 | 0 | 0 | 0 | 0 |  | NA |  | NA | 2 | 2 | 2 | 0 | 0 | 0 |  | 1 | 24 | 0 | 7 | 0 |  |
| 2791 |  | 23.769 | 3.004 | 12.63831 | 87.361687 | 1 | 15.808351 | 0 | -0.21277946 | 2 | 109.86572 | 20.639999 | 72.887 | 0 | 0 | 3 |  | | | | | | | 112.6997373 | 1.085996426 | 104.2729779 | 0.771378051 | 2 | 2 |  | 0 | 0 | 0 | 0 | 4 |  | | | | |
| 2791 | 0 | 19.922001 | 2.05 | 10.290131 | 89.709869 | 1 | 17.114305 | 0 | -0.21277946 | 2 | 109.86572 | 25.836109 | 68.190002 | 0 | 19 | 3 |  | 0 | 0 | 0 | 0 | 0 | 0 |  | NA |  | NA | 2 | 2 | 2 | 0 | 0 | 0 |  | 4 | 24 | 0 | 7 | 0 |  |
| 2791 | 1 | 23.881001 | 2.3900001 | 10.007957 | 89.992043 | 1 | 17.149897 | 0 | -0.21277946 | 2 | 109.86572 | 25.429445 | 71.440002 | 0 | 20 | 3 |  | 1 | 1 | 1 | 1 | 1 | 1 | 111.1700129 | 0.949119698 | 100.7326272 | 0.123614233 | 2 | 2 | 2 | 0 | 0 | 0 | 0 | 4 | 25 | 0 |  | | |
| 2791 | 0 | 22.948999 | 2.51 | 10.937296 | 89.062706 | 1 | 17.182751 | 0 | -0.21277946 | 2 | 109.86572 | 24.993887 | 70.709999 | 0 | 21 | 3 |  | 0 | 0 | 0 | 0 | 0 | 0 |  | NA |  | NA | 2 | 2 | 2 | 0 | 0 | 0 |  | 4 | 24 | 0 | 7 | 0 |  |
| 2792 |  | 24.007 | 3.529 | 14.69988 | 85.300117 | 1 | 14.499658 | 0 | -0.40354156 | 2 | 113.68099 | 28.128334 | 56.182 | 0 | 0 | 3 | 0 |  | | | | | | 118.3977105 | 1.578097686 | 102.7732534 | 0.498867666 | 2 | 2 |  | 0 | 0 | 1 | 0 | 3 |  | | | | |
| 2792 | 0 | 16.128 | 1.7 | 10.540674 | 89.459328 | 1 | 15.794662 | 0 | -0.40354156 | 2 | 113.68099 | 27.086664 | 63.209999 | 0 | 19 | 3 | 0 | 0 | 0 | 0 | 0 | 0 | 0 |  | NA |  | NA | 2 | 2 | 2 | 0 | 0 | 1 |  | 3 | 25 | 0 | 7 | 0 |  |
| 2792 | 0 | 18.101 | 2.1199999 | 11.71206 | 88.287941 | 1 | 15.832991 | 0 | -0.40354156 | 2 | 113.68099 | 27.260555 | 64.279999 | 0 | 20 | 3 | 0 | 0 | 0 | 0 | 0 | 0 | 0 | 104.5178489 | 0.38895495 | 109.3367231 | 1.803816766 | 2 | 2 | 2 | 0 | 0 | 1 | 0 | 3 | 25 | 0 |  | | |
| 2792 | 0 | 27.295 | 3.1300001 | 11.467302 | 88.5327 | 1 | 15.874059 | 0 | -0.40354156 | 2 | 113.68099 | 26.574444 | 62.959999 | 0 | 21 | 3 | 0 | 0 | 0 | 0 | 0 | 0 | 0 |  | NA |  | NA | 2 | 2 | 2 | 0 | 0 | 1 |  | 3 | 25 | 0 | 7 | 0 |  |
| 2793 |  | 28.111 | 3.67 | 13.055387 | 86.944611 | 1 | 14.30527 | 0 | 0.19843566 | 3 | 100.91001 | 21.896667 | 69.49 | 0 | 0 | 3 | 0 |  | | | | | | 100.9740438 | 0.084873858 | 106.584599 | 1.252892508 | 2 | 2 |  | 1 | 0 | 0 | 0 | 3 |  | | | | |
| 2793 | 0 | 16.437 | 1.7 | 10.34252 | 89.657478 | 1 | 15.438741 | 0 | 0.19843566 | 3 | 100.91001 | 24.989447 | 72.809998 | 0 | 19 | 3 | 0 | 0 | 0 | 0 | 0 | 0 | 0 |  | NA |  | NA | 2 | 2 | 2 | 1 | 0 | 0 |  | 3 | 22 | 0 | 6.0999999 | 1 |  |
| 2793 | 1 | 18.691 | 1.98 | 10.593333 | 89.40667 | 1 | 15.488022 | 0 | 0.19843566 | 3 | 100.91001 | 24.597223 | 75.900002 | 0 | 20 | 3 | 0 | 1 | 0 | 0 | 0 | 1 | 1 | 97.97856115 | -0.174969824 | 107.4712401 | 1.406464784 | 2 | 2 | 2 | 1 | 0 | 0 | 0 | 3 | 19 | 1 |  | | |
| 2793 | 1 | 21.277 | 2.29 | 10.762795 | 89.237206 | 1 | 15.515401 | 0 | 0.19843566 | 3 | 100.91001 | 23.80611 | 73.699997 | 0 | 21 | 3 | 0 | 1 | 0 | 0 | 0 | 1 | 0 |  | NA |  | NA | 2 | 2 | 2 | 1 | 0 | 0 |  | 3 | 24 | 0 | 7 | 0 |  |
| 2796 |  | 23.158 | 2.697 | 11.646084 | 88.35392 | 1 | 17.462013 | 0 | 1.7179354 | 2 | 70.088821 | 21.215555 | 72.87 | 0 | 0 | 3 | 1 |  | | | | | | 95.68704193 | -0.371281719 | 78.28502551 | -2.714510862 | 2 | 2 |  | 0 | 0 | 0 | 1 | 1 |  | | | | |
| 2796 | 0 | 19.194 | 1.97 | 10.263624 | 89.736374 | 1 | 18.787132 | 0 | 1.7179354 | 2 | 70.088821 | 25.493889 | 70.629997 | 0 | 19 | 3 | 1 | 0 | 0 | 0 | 0 | 0 | 0 |  | NA |  | NA | 2 | 2 | 2 | 0 | 0 | 0 |  | 1 | 24 | 0 | 7 | 0 |  |
| 2796 | 0 | 22.822001 | 2.47 | 10.822889 | 89.177109 | 1 | 18.825462 | 0 | 1.7179354 | 2 | 70.088821 | 25.203888 | 70.800003 | 0 | 20 | 3 | 1 | 0 | 0 | 0 | 0 | 0 | 0 | 93.65480174 | -0.543865443 | 98.12548172 | -0.299843572 | 2 | 2 | 2 | 0 | 0 | 0 | 0 | 1 | 25 | 0 |  | | |
| 2796 | 1 | 29.982 | 2.8099999 | 9.3722897 | 90.627708 | 1 | 18.863792 | 0 | 1.7179354 | 2 | 70.088821 | 23.713888 | 71.279999 | 0 | 21 | 3 | 1 | 1 | 1 | 1 | 1 | 0 | 1 |  | NA |  | NA | 2 | 2 | 2 | 0 | 0 | 0 |  | 1 | 25 | 0 | 6.6999998 | 1 |  |
| 2797 |  | 24.879 | 3.178 | 12.773826 | 87.226173 | 0 | 10.992471 | 0 | 0.84458739 | 2 | 85.157967 | 24.356112 | 71.105 | 0 | 0 | 3 |  | | | | | | | 92.86918411 | -0.638683325 | 98.06937336 | -0.336067184 | 2 | 2 |  | 0 | 0 | 1 | 0 | 3 |  | | | | |
| 2797 | 0 | 22.917999 | 2.3900001 | 10.428485 | 89.571518 | 1 | 12.227242 | 0 | 0.84458739 | 2 | 85.157967 | 25.948334 | 69.089996 | 0 | 19 | 3 |  | 0 | 0 | 0 | 0 | 0 | 0 |  | NA |  | NA | 2 | 2 | 2 | 0 | 0 | 1 |  | 3 | 24 | 0 | 7 | 0 |  |
| 2797 | 0 | 23.405001 | 2.48 | 10.596026 | 89.403976 | 1 | 12.260096 | 0 | 0.84458739 | 2 | 85.157967 | 26.799999 | 68.589996 | 0 | 20 | 3 |  | 0 | 0 | 0 | 0 | 0 | 0 |  | NA |  | NA | 2 | 2 |  | 0 | 0 | 1 |  | 3 | 24 | 0 |  | | |
| 2797 | 0 | 26.201 | 2.75 | 10.495783 | 89.504219 | 1 | 12.298426 | 0 | 0.84458739 | 2 | 85.157967 | 25.081114 | 68.650002 | 0 | 21 | 3 |  | 0 | 0 | 0 | 0 | 0 | 0 |  | NA |  | NA | 2 | 2 | 2 | 0 | 0 | 1 |  | 3 | 25 | 0 | 7 | 0 |  |
| 2799 |  | 23.611 | 3.5 | 14.823599 | 85.176399 | 1 | 15.392197 | 1 | 1.0696787 | 4 | 110.36433 | 13.709444 | 77.127 | 0 | 0 | 3 | 1 |  | | | | | | 126.343016 | 2.3924042 | 93.6414919 | -0.915908959 | 2 | 2 |  | 1 | 0 | 0 | 0 | 2 |  | | | | |
| 2799 | 0 | 18.228001 | 1.87 | 10.258942 | 89.741058 | 1 | 16.678988 | 1 | 1.0696787 | 4 | 110.36433 | 26.410557 | 65.080002 | 0 | 19 | 3 | 1 | 0 | 0 | 0 | 0 | 0 | 0 |  | NA |  | NA | 2 | 2 | 2 | 1 | 0 | 0 |  | 2 | 25 | 0 | 7 | 0 |  |
| 2799 | 0 | 22.271 | 2.4400001 | 10.955952 | 89.044044 | 1 | 16.717318 | 1 | 1.0696787 | 4 | 110.36433 | 26.42333 | 66.779999 | 0 | 20 | 3 | 1 | 0 | 0 | 0 | 0 | 0 | 0 | 131.2765695 | 2.831441406 | 93.2469341 | -0.946725713 | 2 | 2 | 2 | 1 | 0 | 0 | 0 | 2 | 21 | 0 |  | | |
| 2799 | 0 | 25.893999 | 2.9400001 | 11.353982 | 88.646019 | 1 | 16.733744 | 1 | 1.0696787 | 4 | 110.36433 | 24.990002 | 66.440002 | 0 | 21 | 3 | 1 | 0 | 0 | 0 | 0 | 0 | 0 |  | NA |  | NA | 2 | 2 | 2 | 1 | 0 | 0 |  | 2 | 24 | 0 | 7 | 0 |  |
| 2801 |  | 23.709 | 3.34 | 14.087478 | 85.912521 | 1 | 15.066393 | 1 | 1.1228781 | 3 | 114.43112 | 18.309444 | 73.027 | 0 | 0 | 2 | 1 |  | | | | | | 119.9347707 | 1.814064349 | 102.0997399 | 0.32611231 | 2 | 2 |  | 0 | 0 | 1 | 0 | 4 |  | | | | |
| 2801 | 0 | 18.33 | 1.75 | 9.5471907 | 90.452812 | 1 | 16.355921 | 1 | 1.1228781 | 3 | 114.43112 | 25.926111 | 67.339996 | 0 | 19 | 2 | 1 | 0 | 0 | 0 | 0 | 0 | 0 |  | NA |  | NA | 2 | 2 | 2 | 0 | 0 | 1 |  | 4 | 24 | 0 | 7 | 0 |  |
| 2801 | 0 | 24.337 | 2.73 | 11.217487 | 88.782509 | 1 | 16.435318 | 1 | 1.1228781 | 3 | 114.43112 | 24.517221 | 68.529999 | 0 | 21 | 2 | 1 | 0 | 0 | 0 | 0 | 0 | 0 |  | NA |  | NA | 2 | 2 | 2 | 0 | 0 | 1 |  | 4 | 24 | 0 | 7 | 0 |  |
| 2807 |  | 25.891 | 3.751 | 14.487659 | 85.512337 | 1 | 12.386037 | 1 | -2.8429184 | 3 | 124.45919 | 16.98 | 75.857 | 0 | 0 | 3 | 1 |  | | | | | | 140.7478851 | 3.705429936 | 93.36754031 | -1.004150496 | 1 | 2 |  | 0 | 0 | 1 | 0 | 3 |  | | | | |
| 2807 | 0 | 17.542 | 1.75 | 9.9760571 | 90.023941 | 1 | 13.571527 | 1 | -2.8429184 | 3 | 124.45919 | 25.69278 | 68.120003 | 0 | 19 | 3 | 1 | 0 | 0 | 0 | 0 | 0 | 0 |  | NA |  | NA | 1 | 2 | 2 | 0 | 0 | 1 |  | 3 | 20 | 0 | 4.4000001 | 1 |  |
| 2807 | 1 | 20.492001 | 2.1800001 | 10.638298 | 89.361702 | 1 | 13.612594 | 1 | -2.8429184 | 3 | 124.45919 | 25.096668 | 71.730003 | 0 | 20 | 3 | 1 | 1 | 1 | 1 | 1 | 1 | 1 | 126.5217978 | 2.413115688 | 100.5671292 | 0.08825326 | 1 | 2 | 2 | 0 | 0 | 1 | 0 | 3 | 15 | 1 |  | | |
| 2807 | 1 | 24.275 | 2.6800001 | 11.040165 | 88.959831 | 1 | 13.650924 | 1 | -2.8429184 | 3 | 124.45919 | 24.453333 | 70.080002 | 0 | 21 | 3 | 1 | 1 | 1 | 1 | 1 | 1 | 1 |  | NA |  | NA | 1 | 2 | 2 | 0 | 0 | 1 |  | 3 | 6 | 1 | 2 | 1 |  |
| 2808 |  | 23.825 | 3.406 | 14.295908 | 85.704094 | 1 | 15.723477 | 1 | -2.6182134 | 2 | 120.99493 | 14.642222 | 78.591 | 0 | 0 | 2 | 0 |  | | | | | | 138.5241863 | 3.48792849 | 93.99476307 | -0.86272964 | 2 | 2 |  | 0 | 0 | 0 | 0 | 2 |  | | | | |
| 2808 | 0 | 17.952 | 1.83 | 10.193851 | 89.806152 | 1 | 17.032169 | 1 | -2.6182134 | 2 | 120.99493 | 25.06778 | 70.470001 | 0 | 19 | 2 | 0 | 0 | 0 | 0 | 0 | 0 | 0 |  | NA |  | NA | 2 | 2 | 2 | 0 | 0 | 0 |  | 2 | 25 | 0 | 7 | 0 |  |
| 2808 | 0 | 23.990999 | 2.3099999 | 9.6286106 | 90.371391 | 1 | 17.108829 | 1 | -2.6182134 | 2 | 120.99493 | 23.042776 | 72.059998 | 0 | 21 | 2 | 0 | 0 | 0 | 0 | 0 | 0 | 0 |  | NA |  | NA | 2 | 2 | 2 | 0 | 0 | 0 |  | 2 | 24 | 0 | 7 | 0 |  |
| 2809 |  | 24.69 | 3.182 | 12.887809 | 87.11219 | 0 | 10.464066 | 1 | -1.9491892 | 2 | 119.95472 | 23.611666 | 71.357 | 0 | 0 | 3 | 0 |  | | | | | | 133.3321551 | 3.009263232 | 95.45673441 | -0.705923533 | 2 | 2 |  | 0 |  | 0 | 0 | 3 |  | | | | |
| 2809 | 0 | 21.414 | 2.1700001 | 10.133558 | 89.86644 | 0 | 11.753593 | 1 | -1.9491892 | 2 | 119.95472 | 25.984446 | 68.339996 | 0 | 19 | 3 | 0 | 0 | 0 | 0 | 0 | 0 |  | | NA |  | NA | 2 | 2 | 2 | 0 |  | 0 |  | 3 | 27 | 0 | 7 | 0 |  |
| 2809 | 0 | 22.771 | 2.4400001 | 10.715384 | 89.284615 | 0 | 11.791924 | 1 | -1.9491892 | 2 | 119.95472 | 25.867775 | 71.040001 | 0 | 20 | 3 | 0 | 0 | 0 | 0 | 0 | 0 |  | 114.9560377 | 1.366780401 | 94.91404926 | -0.78552988 | 2 | 2 | 2 | 0 |  | 0 | 0 | 3 | 26 | 0 |  | | |
| 2809 | 1 | 24.820999 | 2.6900001 | 10.837598 | 89.162399 | 0 | 11.838467 | 1 | -1.9491892 | 2 | 119.95472 | 25.412775 | 68.940002 | 0 | 21 | 3 | 0 | 1 | 1 | 1 | 1 | 1 |  | | NA |  | NA | 2 | 2 | 2 | 0 |  | 0 |  | 3 | 24 | 0 | 7 | 0 |  |
| 2811 |  | 23.685 | 3.051 | 12.881571 | 87.118431 | 1 | 16.49555 | 1 | 1.3277028 | 2 | 97.385719 | 19.361111 | 76.589 | 0 | 0 | 3 | 1 |  | | | | | | 128.9837731 | 2.626658411 | 81.53521055 | -2.363147548 | 2 | 2 |  | 0 |  | 1 | 0 | 1 |  | | | | |
| 2811 | 1 | 15.761 | 1.88 | 11.928177 | 88.071823 | 1 | 17.776865 | 1 | 1.3277028 | 2 | 97.385719 | 24.997778 | 72.290001 | 0 | 19 | 3 | 1 | 1 | 1 | 1 | 1 | 1 | 0 |  | NA |  | NA | 2 | 2 | 2 | 0 |  | 1 |  | 1 | 24 | 0 | 7 | 0 |  |
| 2811 | 0 | 20.415001 | 2.1600001 | 10.580456 | 89.419548 | 1 | 17.83436 | 1 | 1.3277028 | 2 | 97.385719 | 24.451666 | 75.760002 | 0 | 20 | 3 | 1 | 0 | 0 | 0 | 0 | 0 | 0 | 120.8134824 | 1.887350912 | 93.62750753 | -0.888478766 | 2 | 2 | 2 | 0 |  | 1 | 0 | 1 | 24 | 0 |  | | |
| 2811 | 0 | 21.915001 | 2.4200001 | 11.042665 | 88.957336 | 1 | 17.87269 | 1 | 1.3277028 | 2 | 97.385719 | 23.699442 | 73.599998 | 0 | 21 | 3 | 1 | 0 | 0 | 0 | 0 | 0 | 0 |  | NA |  | NA | 2 | 2 | 2 | 0 |  | 1 |  | 1 | 25 | 0 | 6.6999998 | 1 |  |
| 2812 |  | 23.809 | 3.198 | 13.431895 | 86.568108 | 1 | 13.311431 | 1 | 0.15118304 | 3 | 90.902275 | 18.688889 | 74.678 | 0 | 0 | 3 | 1 |  | | | | | | 114.1042665 | 1.289291077 | 85.14836782 | -2.060573313 | 2 | 2 |  | 0 | 0 | 0 | 0 | 1 |  | | | | |
| 2812 | 0 | 16.929001 | 1.75 | 10.337291 | 89.662712 | 1 | 14.595483 | 1 | 0.15118304 | 3 | 90.902275 | 26.044998 | 67.879997 | 0 | 19 | 3 | 1 | 0 | 0 | 0 | 0 | 0 | 0 |  | NA |  | NA | 2 | 2 | 2 | 0 | 0 | 0 |  | 1 | 24 | 0 | 7 | 0 |  |
| 2812 | 1 | 19.708 | 2.0999999 | 10.655571 | 89.344429 | 1 | 14.639288 | 1 | 0.15118304 | 3 | 90.902275 | 25.634998 | 70.650002 | 0 | 20 | 3 | 1 | 1 | 0 | 0 | 0 | 0 | 1 | 109.4668523 | 0.863655358 | 92.27251282 | -1.107182271 | 2 | 2 | 2 | 0 | 0 | 0 | 0 | 1 | 25 | 0 |  | | |
| 2812 | 1 | 21.982 | 2.4000001 | 10.918024 | 89.081978 | 1 | 14.680356 | 1 | 0.15118304 | 3 | 90.902275 | 25.016111 | 69.080002 | 0 | 21 | 3 | 1 | 1 | 1 | 1 | 0 | 0 | 0 |  | NA |  | NA | 2 | 2 | 2 | 0 | 0 | 0 |  | 1 | 25 | 0 | 7 | 0 |  |
| 2813 |  | 27.153 | 3.169 | 11.670902 | 88.329102 | 0 | 9.9164953 | 1 | -2.6940265 | 3 | 112.54826 | 22.06889 | 71.845 | 0 | 0 | 3 | 1 |  | | | | | | 147.0302804 | 4.204267358 | 82.02962301 | -2.416331785 | 2 | 2 |  | 0 |  | 0 | 0 | 1 |  | | | | |
| 2813 | 1 | 18.639999 | 1.87 | 10.032189 | 89.967812 | 0 | 10.997947 | 1 | -2.6940265 | 3 | 112.54826 | 25.541666 | 69.75 | 0 | 19 | 3 | 1 | 1 | 1 | 1 | 1 | 0 |  | | NA |  | NA | 2 | 2 | 2 | 0 |  | 0 |  | 1 | 26 | 0 | 7 | 0 |  |
| 2813 | 1 | 21.063 | 2.25 | 10.682239 | 89.317764 | 0 | 11.036277 | 1 | -2.6940265 | 3 | 112.54826 | 25.218887 | 72.519997 | 0 | 20 | 3 | 1 | 1 | 1 | 1 | 1 | 0 |  | 106.1375266 | 0.560308602 | 63.53841729 | -4.111930316 | 2 | 2 | 2 | 0 |  | 0 | 1 | 1 | 26 | 0 |  | | |
| 2813 | 1 | 24.372999 | 2.6400001 | 10.831658 | 89.168343 | 0 | 11.071869 | 1 | -2.6940265 | 3 | 112.54826 | 24.629999 | 70.760002 | 0 | 21 | 3 | 1 | 1 | 0 | 1 | 0 | 0 |  | | NA |  | NA | 2 | 2 | 2 | 0 |  | 0 |  | 1 | 18 | 1 | 7 | 0 |  |
| 2814 |  | 24.478 | 2.775 | 11.336711 | 88.663292 | 0 | 11.816564 | 1 | -0.76465905 | 2 | 101.84796 | 20.791666 | 73.937 | 0 | 0 | 3 | 1 |  | | | | | | 115.4784622 | 1.414363772 | 94.28940696 | -0.876876242 | 2 | 2 |  | 0 | 0 | 0 | 1 | 2 |  | | | | |
| 2814 | 0 | 18.945 | 1.92 | 10.1346 | 89.865402 | 1 | 13.051334 | 1 | -0.76465905 | 2 | 101.84796 | 25.49778 | 70.050003 | 0 | 19 | 3 | 1 | 0 | 0 | 0 | 0 | 0 | 0 |  | NA |  | NA | 2 | 2 | 2 | 0 | 0 | 0 |  | 2 | 24 | 0 | 7 | 0 |  |
| 2814 | 0 | 21.504 | 2.3299999 | 10.835193 | 89.16481 | 1 | 13.089664 | 1 | -0.76465905 | 2 | 101.84796 | 25.449999 | 71.800003 | 0 | 20 | 3 | 1 | 0 | 0 | 0 | 0 | 0 | 0 | 104.054505 | 0.371534977 | 94.37228462 | -0.847482358 | 2 | 2 | 2 | 0 | 0 | 0 | 0 | 2 | 24 | 0 |  | | |
| 2814 | 1 | 27.68 | 2.76 | 9.9710979 | 90.0289 | 1 | 13.125257 | 1 | -0.76465905 | 2 | 101.84796 | 24.06389 | 70.959999 | 0 | 21 | 3 | 1 | 1 | 1 | 1 | 1 | 0 | 1 |  | NA |  | NA | 2 | 2 | 2 | 0 | 0 | 0 |  | 2 | 15 | 1 | 5.9000001 | 1 |  |
| 2815 |  | 22.923 | 2.821 | 12.306417 | 87.693581 | 1 | 16.205339 | 1 | -0.89183426 | 2 | 108.90531 | 21.468889 | 73.06 | 0 | 0 | 3 | 1 |  | | | | | | 108.5090453 | 0.775057489 | 105.8843925 | 0.922569156 | 2 | 2 |  | 0 | 0 | 0 | 0 | 3 |  | | | | |
| 2815 | 0 | 20.709 | 2.3800001 | 11.492588 | 88.507408 | 1 | 17.500341 | 1 | -0.89183426 | 2 | 108.90531 | 24.956665 | 71.5 | 0 | 19 | 3 | 1 | 0 | 0 | 0 | 0 | 0 | 0 |  | NA |  | NA | 2 | 2 | 2 | 0 | 0 | 0 |  | 3 | 24 | 0 | 7 | 0 |  |
| 2815 | 0 | 23.625 | 2.8299999 | 11.978835 | 88.021164 | 1 | 17.535934 | 1 | -0.89183426 | 2 | 108.90531 | 24.369444 | 74.410004 | 0 | 20 | 3 | 1 | 0 | 0 | 0 | 0 | 0 | 0 | 97.7317011 | -0.206895091 | 109.401928 | 1.485454771 | 2 | 2 | 2 | 0 | 0 | 0 | 0 | 3 | 24 | 0 |  | | |
| 2815 | 0 | 26.466 | 3.26 | 12.317691 | 87.682312 | 1 | 17.574265 | 1 | -0.89183426 | 2 | 108.90531 | 23.495001 | 72.870003 | 0 | 21 | 3 | 1 | 0 | 0 | 0 | 0 | 0 | 0 |  | NA |  | NA | 2 | 2 | 2 | 0 | 0 | 0 |  | 3 | 24 | 0 | 7 | 0 |  |
| 2817 |  | 23.886 | 3.44 | 14.401742 | 85.598259 | 1 | 17.152636 | 1 | -2.4363396 | 3 | 108.05882 | 14.512777 | 78.688 | 0 | 0 | 3 | 1 |  | | | | | | 119.0432612 | 1.728535692 | 97.06186189 | -0.424069049 | 2 | 2 |  | 0 | 0 | 0 | 0 |  | | | | | |
| 2817 | 0 | 17.548 | 1.77 | 10.086619 | 89.913383 | 1 | 18.444901 | 1 | -2.4363396 | 3 | 108.05882 | 25.230001 | 69.339996 | 0 | 19 | 3 | 1 | 0 | 0 | 0 | 0 | 0 | 0 |  | NA |  | NA | 2 | 2 | 2 | 0 | 0 | 0 |  | | 24 | 0 | 7 | 0 |  |
| 2817 | 0 | 21.153 | 2.1600001 | 10.211318 | 89.788681 | 1 | 18.483231 | 1 | -2.4363396 | 3 | 108.05882 | 24.925554 | 71.900002 | 0 | 20 | 3 | 1 | 0 | 0 | 0 | 0 | 0 | 0 | 107.3009933 | 0.663685014 | 98.12557624 | -0.268737494 | 2 | 2 | 2 | 0 | 0 | 0 | 0 |  | 21 | 0 |  | | |
| 2817 | 1 | 25.568001 | 2.8800001 | 11.26408 | 88.735916 | 1 | 18.502396 | 1 | -2.4363396 | 3 | 108.05882 | 23.678335 | 70.989998 | 0 | 21 | 3 | 1 | 1 | 1 | 0 | 0 | 1 | 1 |  | NA |  | NA | 2 | 2 | 2 | 0 | 0 | 0 |  | | 20 | 0 | 6.4000001 | 1 |  |
| 2821 |  | 23.758 | 2.963 | 12.471588 | 87.528412 | 1 | 17.900068 | 1 | 0.20952076 | 4 | 80.828354 | 24.022778 | 67.196 | 0 | 0 | 2 | 0 |  | | | | | | 88.21685437 | -1.077279033 | 95.65316619 | -0.615600924 | 2 | 2 |  | 1 | 1 | 0 | 0 | 3 |  | | | | |
| 2821 | 0 | 17.410999 | 1.86 | 10.682902 | 89.317101 | 1 | 19.216974 | 1 | 0.20952076 | 4 | 80.828354 | 23.715557 | 79.360001 | 0 | 20 | 2 | 0 | 0 | 0 | 0 | 0 | 0 | 0 | 103.734615 | 0.339681346 | 99.40993522 | -0.085230557 | 2 | 2 | 2 | 1 | 1 | 0 | 0 | 3 | 24 | 0 |  | | |
| 2821 | 0 | 19.632999 | 2.0599999 | 10.492538 | 89.507462 | 1 | 19.255304 | 1 | 0.20952076 | 4 | 80.828354 | 23.601667 | 76.400002 | 0 | 21 | 2 | 0 | 0 | 0 | 0 | 0 | 0 | 0 |  | NA |  | NA | 2 | 2 | 2 | 1 | 1 | 0 |  | 3 | 24 | 0 | 7 | 0 |  |
| 2822 |  | 24.215 | 3.272 | 13.512286 | 86.487717 | 0 | 10.740588 | 0 | 1.7179354 | 3 | 102.83505 | 18.956667 | 74.354 | 0 | 0 | 3 | 1 |  | | | | | | 114.2834295 | 1.256444802 | 98.22667302 | -0.310019931 | 2 | 2 |  | 0 | 0 | 1 | 1 | 3 |  | | | | |
| 2822 | 0 | 17.403999 | 1.78 | 10.227534 | 89.772469 | 1 | 12.005476 | 0 | 1.7179354 | 3 | 102.83505 | 25.749998 | 69.169998 | 0 | 19 | 3 | 1 | 0 | 0 | 0 | 0 | 0 | 0 |  | NA |  | NA | 2 | 2 | 2 | 0 | 0 | 1 |  | 3 | 27 | 0 | 7 | 0 |  |
| 2822 | 1 | 19.941 | 2.1400001 | 10.731659 | 89.268341 | 1 | 12.043806 | 0 | 1.7179354 | 3 | 102.83505 | 25.55611 | 71.809998 | 0 | 20 | 3 | 1 | 1 | 1 | 1 | 1 | 1 | 1 | 114.7716706 | 1.291967754 | 102.1054568 | 0.386266739 | 2 | 2 | 2 | 0 | 0 | 1 | 0 | 3 | 25 | 0 |  | | |
| 2822 | 0 | 22.202 | 2.4200001 | 10.89992 | 89.100082 | 1 | 12.1013 | 0 | 1.7179354 | 3 | 102.83505 | 24.748888 | 70 | 0 | 21 | 3 | 1 | 0 | 0 | 0 | 0 | 0 | 0 |  | NA |  | NA | 2 | 2 | 2 | 0 | 0 | 1 |  | 3 | 25 | 0 | 7 | 0 |  |
| 2823 |  | 24.087 | 3.224 | 13.384813 | 86.615189 | 0 | 11.370295 | 0 | 0.63891757 | 3 | 95.838814 | 19.327778 | 73.926 | 0 | 0 | 3 | 0 |  | | | | | | 101.7988709 | 0.159622521 | 100.7253491 | 0.131214076 | 2 | 2 |  | 0 | 0 | 0 | 0 | 3 |  | | | | |
| 2823 | 0 | 17.23 | 1.8 | 10.446895 | 89.553108 | 1 | 12.635181 | 0 | 0.63891757 | 3 | 95.838814 | 25.718332 | 69.209999 | 0 | 19 | 3 | 0 | 0 | 0 | 0 | 0 | 0 | 0 |  | NA |  | NA | 2 | 2 | 2 | 0 | 0 | 0 |  | 3 | 24 | 0 | 7 | 0 |  |
| 2823 | 0 | 20.437 | 2.24 | 10.960513 | 89.03949 | 1 | 12.681725 | 0 | 0.63891757 | 3 | 95.838814 | 25.522223 | 71.260002 | 0 | 20 | 3 | 0 | 0 | 0 | 0 | 0 | 0 | 0 | 90.56701084 | -0.838303178 | 94.89913003 | -0.833008842 | 2 | 2 | 2 | 0 | 0 | 0 | 0 | 3 | 24 | 0 |  | | |
| 2823 | 0 | 22.889 | 2.49 | 10.878588 | 89.121414 | 1 | 12.714579 | 0 | 0.63891757 | 3 | 95.838814 | 24.357779 | 69.75 | 0 | 21 | 3 | 0 | 0 | 0 | 0 | 0 | 0 | 0 |  | NA |  | NA | 2 | 2 | 2 | 0 | 0 | 0 |  | 3 | 24 | 0 | 7 | 0 |  |
| 2824 |  | 24.53 | 3.358 | 13.68936 | 86.310638 | 1 | 13.598905 | 1 | -1.0517983 | 2 | 96.15818 | 18.662222 | 74.663 | 0 | 0 | 3 | 1 |  | | | | | | 91.91357148 | -0.74303988 | 111.872061 | 2.060082101 | 2 | 2 |  | 0 | 0 | 0 | 0 | 3 |  | | | | |
| 2824 | 0 | 17.671 | 1.77 | 10.016411 | 89.983589 | 1 | 14.863791 | 1 | -1.0517983 | 2 | 96.15818 | 25.746668 | 69.519997 | 0 | 19 | 3 | 1 | 0 | 0 | 0 | 0 | 0 | 0 |  | NA |  | NA | 2 | 2 | 2 | 0 | 0 | 0 |  | 3 | 24 | 0 | 7 | 0 |  |
| 2824 | 0 | 19.867001 | 2.0799999 | 10.469622 | 89.53038 | 1 | 14.910336 | 1 | -1.0517983 | 2 | 96.15818 | 25.374447 | 72.129997 | 0 | 20 | 3 | 1 | 0 | 0 | 0 | 0 | 0 | 0 | 93.39534686 | -0.60501578 | 109.7656277 | 1.61238212 | 2 | 2 | 2 | 0 | 0 | 0 | 0 | 3 | 24 | 0 |  | | |
| 2824 | 0 | 21.913 | 2.3800001 | 10.861134 | 89.13887 | 1 | 14.94319 | 1 | -1.0517983 | 2 | 96.15818 | 24.778332 | 70.349998 | 0 | 21 | 3 | 1 | 0 | 0 | 0 | 0 | 0 | 0 |  | NA |  | NA | 2 | 2 | 2 | 0 | 0 | 0 |  | 3 | 24 | 0 | 7 | 0 |  |
| 2825 |  | 24.157 | 3.124 | 12.93207 | 87.067932 | 0 | 9.4647503 | 0 | 1.9194371 | 2 | 105.47252 | 19.276112 | 76.462 | 0 | 0 | 3 |  | | | | | | | 115.622594 | 1.364645288 | 95.75839926 | -0.716891975 | 2 | 2 |  | 0 | 0 | 1 | 1 | 3 |  | | | | |
| 2825 | 1 | 17.429001 | 2.02 | 11.589878 | 88.410118 | 0 | 10.748802 | 0 | 1.9194371 | 2 | 105.47252 | 24.887775 | 73.279999 | 0 | 19 | 3 |  | 1 | 1 | 1 | 1 | 1 |  | | NA |  | NA | 2 | 2 | 2 | 0 | 0 | 1 |  | 3 | 26 | 0 | 6.9000001 | 1 |  |
| 2825 | 0 | 20.608999 | 2.24 | 10.869039 | 89.130959 | 0 | 10.787132 | 0 | 1.9194371 | 2 | 105.47252 | 24.654999 | 74.43 | 0 | 20 | 3 |  | 0 | 0 | 0 | 0 | 0 |  | 117.8758455 | 1.567890264 | 84.78493582 | -2.142690403 | 2 | 2 | 2 | 0 | 0 | 1 | 1 | 3 | 27 | 0 |  | | |
| 2825 | 0 | 22.395 | 2.46 | 10.984594 | 89.015404 | 0 | 10.825462 | 0 | 1.9194371 | 2 | 105.47252 | 23.753332 | 73.129997 | 0 | 21 | 3 |  | 0 | 0 | 0 | 0 | 0 |  | | NA |  | NA | 2 | 2 | 2 | 0 | 0 | 1 |  | 3 | 24 | 0 | 7 | 0 |  |
| 2826 |  | 24.306 | 3.755 | 15.44886 | 84.55114 | 1 | 17.587954 | 1 | 0.6383335 | 2 | 102.2141 | 21.842777 | 72.791 | 0 | 0 | 3 | 1 |  | | | | | | 126.4705168 | 2.397797537 | 87.19405039 | -1.698439061 | 2 | 2 |  | 0 | 0 | 0 | 1 | 1 |  | | | | |
| 2826 | 1 | 17.120001 | 1.76 | 10.280374 | 89.719627 | 1 | 18.828199 | 1 | 0.6383335 | 2 | 102.2141 | 24.20389 | 74.669998 | 0 | 19 | 3 | 1 | 1 | 1 | 1 | 1 | 1 | 1 |  | NA |  | NA | 2 | 2 | 2 | 0 | 0 | 0 |  | 1 | 24 | 0 | 6.9000001 | 1 |  |
| 2826 | 1 | 21.774 | 2.4200001 | 11.114173 | 88.885826 | 1 | 18.866529 | 1 | 0.6383335 | 2 | 102.2141 | 24.04611 | 75.870003 | 0 | 20 | 3 | 1 | 1 | 1 | 1 | 1 | 1 | 1 | 128.5293582 | 2.580681685 | 90.58714435 | -1.271322665 | 2 | 2 | 2 | 0 | 0 | 0 | 0 | 1 | 24 | 0 |  | | |
| 2826 | 0 | 26.372999 | 2.9200001 | 11.071931 | 88.92807 | 1 | 18.90486 | 1 | 0.6383335 | 2 | 102.2141 | 23.089443 | 74.43 | 0 | 21 | 3 | 1 | 0 | 0 | 0 | 0 | 0 | 0 |  | NA |  | NA | 2 | 2 | 2 | 0 | 0 | 0 |  | 1 | 24 | 0 | 7 | 0 |  |
| 2828 |  | 24.071 | 3.596 | 14.939138 | 85.06086 | 0 | 11.797399 | 1 | -1.9491892 | 3 | 103.22982 | 21.856112 | 71.811 | 0 | 0 | 3 | 1 |  | | | | | | 126.2894186 | 2.396227481 | 87.35940332 | -1.818829523 | 2 | 2 |  | 0 | 0 | 0 | 0 | 2 |  | | | | |
| 2828 | 1 | 16.503 | 1.6799999 | 10.179967 | 89.82003 | 1 | 12.991102 | 1 | -1.9491892 | 3 | 103.22982 | 24.912224 | 71.910004 | 0 | 19 | 3 | 1 | 1 | 1 | 1 | 1 | 1 | 1 |  | NA |  | NA | 2 | 2 | 2 | 0 | 0 | 0 |  | 2 | 24 | 0 | 7 | 0 |  |
| 2828 | 1 | 19.079 | 2.0599999 | 10.797211 | 89.202789 | 1 | 13.032169 | 1 | -1.9491892 | 3 | 103.22982 | 24.283888 | 75.169998 | 0 | 20 | 3 | 1 | 1 | 1 | 1 | 1 | 1 | 1 | 118.0984051 | 1.651503947 | 94.28514971 | -0.852925401 | 2 | 2 | 2 | 0 | 0 | 0 | 0 | 2 | 20 | 0 |  | | |
| 2828 | 0 | 24.719 | 2.75 | 11.125046 | 88.874954 | 1 | 13.070499 | 1 | -1.9491892 | 3 | 103.22982 | 24.044443 | 73.029999 | 0 | 21 | 3 | 1 | 0 | 0 | 0 | 0 | 0 | 0 |  | NA |  | NA | 2 | 2 | 2 | 0 | 0 | 0 |  | 2 | 24 | 0 | 7 | 0 |  |
| 2829 |  | 24.109 | 3.714 | 15.405035 | 84.594963 | 0 | 9.4099932 | 0 | -1.6025475 | 3 | 94.431366 | 21.611111 | 73.47 | 0 | 0 | 3 | 1 |  | | | | | | 109.4084561 | 0.825467955 | 92.53605238 | -1.195147814 | 2 | 2 |  | 0 | 0 | 0 | 0 | 3 |  | | | | |
| 2829 | 0 | 16.160999 | 1.64 | 10.147887 | 89.852112 | 0 | 10.666667 | 0 | -1.6025475 | 3 | 94.431366 | 24.407221 | 73.970001 | 0 | 19 | 3 | 1 | 0 | 0 | 0 | 0 | 0 |  | | NA |  | NA | 2 | 2 | 2 | 0 | 0 | 0 |  | 3 | 19 | 1 | 5 | 1 |  |
| 2829 | 0 | 18.923 | 2.04 | 10.780532 | 89.219467 | 0 | 10.704996 | 0 | -1.6025475 | 3 | 94.431366 | 23.62278 | 77.599998 | 0 | 20 | 3 | 1 | 0 | 0 | 0 | 0 | 0 |  | 98.36653748 | -0.145336795 | 100.1775746 | 0.03182651 | 2 | 2 | 2 | 0 | 0 | 0 | 1 | 3 | 25 | 0 |  | | |
| 2829 | 0 | 24.407 | 2.6900001 | 11.021429 | 88.978569 | 0 | 10.746064 | 0 | -1.6025475 | 3 | 94.431366 | 23.532778 | 75.279999 | 0 | 21 | 3 | 1 | 0 | 0 | 0 | 0 | 0 |  | | NA |  | NA | 2 | 2 | 2 | 0 | 0 | 0 |  | 3 | 27 | 0 | 7 | 0 |  |
| 2830 |  | 24.259 | 3.248 | 13.388845 | 86.611153 | 1 | 14.015059 | 1 | 0.043770012 | 2 | 104.38855 | 18.336666 | 75.857 | 0 | 0 | 3 | 1 |  | | | | | | 117.4447442 | 1.591494209 | 94.21129964 | -0.859526938 | 2 | 2 |  | 0 | 0 | 0 | 0 | 2 |  | | | | |
| 2830 | 0 | 17.320999 | 1.83 | 10.56521 | 89.434792 | 1 | 15.285421 | 1 | 0.043770012 | 2 | 104.38855 | 26.030558 | 67.669998 | 0 | 19 | 3 | 1 | 0 | 0 | 0 | 0 | 0 | 0 |  | NA |  | NA | 2 | 2 | 2 | 0 | 0 | 0 |  | 2 | 25 | 0 | 7 | 0 |  |
| 2830 | 0 | 20.808001 | 2.27 | 10.909266 | 89.090736 | 1 | 15.342916 | 1 | 0.043770012 | 2 | 104.38855 | 25.637779 | 69.949997 | 0 | 20 | 3 | 1 | 0 | 0 | 0 | 0 | 0 | 0 | 102.9076254 | 0.265370391 | 92.6661339 | -1.040647684 | 2 | 2 | 2 | 0 | 0 | 0 | 0 | 2 | 25 | 0 |  | | |
| 2830 | 1 | 23.747999 | 2.5799999 | 10.864073 | 89.135925 | 1 | 15.362081 | 1 | 0.043770012 | 2 | 104.38855 | 24.583889 | 68.699997 | 0 | 21 | 3 | 1 | 1 | 1 | 1 | 0 | 0 | 0 |  | NA |  | NA | 2 | 2 | 2 | 0 | 0 | 0 |  | 2 | 23 | 0 | 7 | 0 |  |
| 2835 |  | 25.121 | 3.267 | 13.005055 | 86.994942 | 1 | 13.708419 | 0 | 0.51320136 | 2 | 109.90473 | 22.018333 | 72.091 | 0 | 0 | 3 | 1 |  | | | | | | 117.5372872 | 1.51329469 | 100.1881808 | 0.032931442 | 2 | 2 |  | 0 | 0 | 1 | 0 | 3 |  | | | | |
| 2835 | 0 | 19.632 | 2.0699999 | 10.544009 | 89.455994 | 1 | 14.948666 | 0 | 0.51320136 | 2 | 109.90473 | 25.625555 | 69.57 | 0 | 19 | 3 | 1 | 0 | 0 | 0 | 0 | 0 | 0 |  | NA |  | NA | 2 | 2 | 2 | 0 | 0 | 1 |  | 3 | 24 | 0 | 7 | 0 |  |
| 2835 | 0 | 22 | 2.4100001 | 10.954546 | 89.045456 | 1 | 14.98152 | 0 | 0.51320136 | 2 | 109.90473 | 25.372776 | 72.559998 | 0 | 20 | 3 | 1 | 0 | 0 | 0 | 0 | 0 | 0 | 114.9722159 | 1.281503567 | 100.6649628 | 0.115000129 | 2 | 2 | 1 | 0 | 0 | 1 | 0 | 3 | 12 | 1 |  | | |
| 2835 | 0 | 23.91 | 2.6800001 | 11.2087 | 88.791298 | 1 | 15.025325 | 0 | 0.51320136 | 2 | 109.90473 | 24.833332 | 70.610001 | 0 | 21 | 3 | 1 | 0 | 0 | 0 | 0 | 0 | 0 |  | NA |  | NA | 2 | 2 | 1 | 0 | 0 | 1 |  | 3 | 24 | 0 | 7 | 0 |  |
| 2836 |  | 25.411 | 3.292 | 12.95502 | 87.044983 | 0 | 9.4318962 | 1 | -1.7033793 | 2 | 109.71983 | 22.344999 | 72.928 | 0 | 0 | 3 | 1 |  | | | | | | 126.255359 | 2.337875626 | 92.65678529 | -1.095822178 | 2 | 2 |  | 0 | 0 | 0 | 0 | 2 |  | | | | |
| 2836 | 0 | 20.68 | 2.0999999 | 10.154738 | 89.845261 | 0 | 10.685832 | 1 | -1.7033793 | 2 | 109.71983 | 25.889997 | 68.709999 | 0 | 19 | 3 | 1 | 0 | 0 | 0 | 0 | 0 |  | | NA |  | NA | 2 | 2 | 2 | 0 | 0 | 0 |  | 2 | 27 | 0 | 7 | 0 |  |
| 2836 | 0 | 22.306999 | 2.3699999 | 10.624468 | 89.375534 | 0 | 10.724161 | 1 | -1.7033793 | 2 | 109.71983 | 25.734999 | 71.459999 | 0 | 20 | 3 | 1 | 0 | 0 | 0 | 0 | 0 |  | 119.4843766 | 1.769168043 | 103.2076108 | 0.537704193 | 2 | 2 | 1 | 0 | 0 | 0 | 0 | 2 | 24 | 0 |  | | |
| 2836 | 1 | 24.108999 | 2.5899999 | 10.742876 | 89.257126 | 0 | 10.762491 | 1 | -1.7033793 | 2 | 109.71983 | 25.336113 | 69.260002 | 0 | 21 | 3 | 1 | 1 | 1 | 1 | 1 | 1 |  | | NA |  | NA | 2 | 2 | 1 | 0 | 0 | 0 |  | 2 | 26 | 0 | 7 | 0 |  |
| 2837 |  | 24.126 | 3.662 | 15.178645 | 84.821358 | 0 | 9.7056808 | 0 | -0.99152374 | 2 | 104.19366 | 18.47611 | 70.6 | 0 | 0 | 3 | 1 |  | | | | | | 111.571224 | 1.016362217 | 99.19665688 | -0.143622049 | 2 | 2 |  | 0 | 0 | 0 | 0 | 4 |  | | | | |
| 2837 | 0 | 18.104 | 1.5700001 | 8.6721172 | 91.327881 | 0 | 10.986995 | 0 | -0.99152374 | 2 | 104.19366 | 26.359444 | 65.629997 | 0 | 19 | 3 | 1 | 0 | 0 | 0 | 0 | 0 |  | | NA |  | NA | 2 | 2 | 2 | 0 | 0 | 0 |  | 4 | 27 | 0 | 7 | 0 |  |
| 2837 | 0 | 19.698 | 2.1900001 | 11.117881 | 88.882118 | 0 | 11.025325 | 0 | -0.99152374 | 2 | 104.19366 | 26.408335 | 66.529999 | 0 | 20 | 3 | 1 | 0 | 0 | 0 | 0 | 0 |  | 111.6519468 | 1.025555456 | 99.89171346 | -0.019342831 | 2 | 2 | 2 | 0 | 0 | 0 | 0 | 4 | 26 | 0 |  | | |
| 2837 | 0 | 22.684999 | 2.6300001 | 11.593565 | 88.406433 | 0 | 11.063655 | 0 | -0.99152374 | 2 | 104.19366 | 25.151669 | 65.389999 | 0 | 21 | 3 | 1 | 0 | 0 | 0 | 0 | 0 |  | | NA |  | NA | 2 | 2 | 2 | 0 | 0 | 0 |  | 4 | 26 | 0 | 7 | 0 |  |
| 2844 |  | 24.478 | 3.721 | 15.201406 | 84.798592 | 1 | 12.867899 | 1 | 1.4721255 | 2 | 96.008911 | 16.605 | 73.72 | 0 | 0 | 3 | 1 |  | | | | | | 106.7761262 | 0.620798813 | 96.64533071 | -0.51936091 | 2 | 2 |  | 0 | 0 | 1 | 0 | 2 |  | | | | |
| 2844 | 0 | 17.032 | 1.46 | 8.5720997 | 91.427902 | 1 | 14.105408 | 1 | 1.4721255 | 2 | 96.008911 | 26.489445 | 65.120003 | 0 | 19 | 3 | 1 | 0 | 0 | 0 | 0 | 0 | 0 |  | NA |  | NA | 2 | 2 | 2 | 0 | 0 | 1 |  | 2 | 24 | 0 | 7 | 0 |  |
| 2844 | 0 | 19.848 | 2.0999999 | 10.580411 | 89.419586 | 1 | 14.143737 | 1 | 1.4721255 | 2 | 96.008911 | 25.754446 | 67.900002 | 0 | 20 | 3 | 1 | 0 | 0 | 0 | 0 | 0 | 0 | 91.82911323 | -0.749633128 | 94.63063786 | -0.793137703 | 2 | 2 | 2 | 0 | 0 | 1 | 0 | 2 | 24 | 0 |  | | |
| 2844 | 0 | 22.773001 | 2.5699999 | 11.285293 | 88.714706 | 1 | 14.187543 | 1 | 1.4721255 | 2 | 96.008911 | 25.883331 | 66.160004 | 0 | 21 | 3 | 1 | 0 | 0 | 0 | 0 | 0 | 0 |  | NA |  | NA | 2 | 2 | 2 | 0 | 0 | 1 |  | 2 | 24 | 0 | 7 | 0 |  |
| 2845 |  | 24.626 | 3.219 | 13.07155 | 86.928452 | 1 | 12.525667 | 1 | -2.2758231 | 2 | 107.21842 | 18.620001 | 74.594 | 0 | 0 | 3 | 1 |  | | | | | | 115.3522053 | 1.403874248 | 99.61925947 | -0.060848819 | 2 | 2 |  | 0 | 0 | 0 | 0 | 3 |  | | | | |
| 2845 | 0 | 17.849001 | 1.72 | 9.6363935 | 90.363609 | 1 | 13.744011 | 1 | -2.2758231 | 2 | 107.21842 | 25.807224 | 68.150002 | 0 | 19 | 3 | 1 | 0 | 0 | 0 | 0 | 0 | 0 |  | NA |  | NA | 2 | 2 | 2 | 0 | 0 | 0 |  | 3 | 24 | 0 | 7 | 0 |  |
| 2845 | 0 | 20.277 | 2.1400001 | 10.55383 | 89.446167 | 1 | 13.785079 | 1 | -2.2758231 | 2 | 107.21842 | 25.149443 | 71.230003 | 0 | 20 | 3 | 1 | 0 | 0 | 0 | 0 | 0 | 0 | 98.86335562 | -0.104206532 | 107.7013042 | 1.285636762 | 2 | 2 | 2 | 0 | 0 | 0 | 0 | 3 | 25 | 0 |  | | |
| 2845 | 0 | 23.537001 | 2.5699999 | 10.918978 | 89.081024 | 1 | 13.826146 | 1 | -2.2758231 | 2 | 107.21842 | 25.036667 | 69.540001 | 0 | 21 | 3 | 1 | 0 | 0 | 0 | 0 | 0 | 0 |  | NA |  | NA | 2 | 2 | 2 | 0 | 0 | 0 |  | 3 | 25 | 0 | 7 | 0 |  |
| 8112 | 0 | 18.926 | 6.384 | 33.731377 | 66.268623 | 0 | 11.425052 | 1 | 2.2397017 | 4 | 100.14785 | 16.647223 | 84.285 | 1 | 0 | 4 | 1 | 0 | 0 | 0 | 0 | 0 |  | 126.4242054 | 2.40558667 | 84.60257117 | -2.158937153 | 2 | 2 |  | 0 | 0 | 0 | 0 | 1 |  | | | | |
| 8112 | 1 | 24.479 | 7.5 | 30.638506 | 69.361496 | 1 | 12.183436 | 1 | 2.2397017 | 4 | 100.14785 | 21.40889 | 77.769997 | 1 | 1 | 4 | 1 | 1 | 0 | 0 | 0 | 1 | 0 | 125.6837878 | 2.342814207 | 87.84213509 | -1.752989359 | 2 | 2 | 2 | 0 | 0 | 0 |  | 1 | 24 | 0 | 7 | 0 |  |
| 8112 | 0 | 17.066999 | 5.5700002 | 32.636086 | 67.363914 | 1 | 12.336756 | 1 | 2.2397017 | 4 | 100.14785 | 19.269444 | 84.260002 | 1 | 5 | 4 | 1 | 0 | 0 | 0 | 0 | 0 | 0 |  | NA |  | NA | 2 | 2 | 2 | 0 | 0 | 0 |  | 1 | 24 | 0 | 7 | 0 |  |
| 8112 | 0 | 20.075001 | 5.0100002 | 24.956413 | 75.043587 | 1 | 12.394251 | 1 | 2.2397017 | 4 | 100.14785 | 17.605555 | 86.519997 | 1 | 6 | 4 | 1 | 0 | 0 | 0 | 0 | 0 | 0 | 111.0550232 | 1.011959231 | 87.97641231 | -1.731331894 | 2 | 2 | 2 | 0 | 0 | 0 | 0 | 1 | 24 | 0 | 7 | 0 |  |
| 8112 | 0 | 22.169001 | 5.0500002 | 22.779556 | 77.220444 | 1 | 12.668036 | 1 | 2.2397017 | 4 | 100.14785 | 17.310001 | 84.080002 | 1 | 12 | 4 | 1 | 0 | 0 | 0 | 0 | 0 | 0 | 115.1433155 | 1.384742019 | 92.33874196 | -1.144919629 | 2 | 2 | 2 | 0 | 0 | 0 |  | 1 | 23 | 0 | 7 | 0 |  |
| 8156 | 0 | 19.727 | 6.17 | 31.27693 | 68.723068 | 0 | 11.186858 | 1 | -0.65196854 | 2 | 99.198441 | 18.43111 | 78.415 | 1 | 0 | 3 | 0 | 0 | 0 | 0 | 0 | 0 |  | 113.7353435 | 1.252612336 | 93.35966534 | -1.013340349 | 2 | 2 |  | 1 | 0 | 0 | 0 | 3 |  | | | | |
| 8156 | 0 | 24.007 | 7.7399998 | 32.240597 | 67.759407 | 0 | 11.835729 | 1 | -0.65196854 | 2 | 99.198441 | 21.452776 | 77.199997 | 1 | 1 | 3 | 0 | 0 | 0 | 0 | 0 | 0 |  | 109.4404946 | 0.86391763 | 99.28755421 | -0.11452118 | 2 | 2 | 2 | 1 | 0 | 0 |  | 3 | 23 | 0 | 6.6999998 | 1 |  |
| 8156 | 1 | 17.612 | 6.4200001 | 36.452419 | 63.547581 | 0 | 11.906913 | 1 | -0.65196854 | 2 | 99.198441 | 20.260557 | 82.050003 | 1 | 3 | 3 | 0 | 1 | 0 | 0 | 0 | 0 |  | | NA |  | NA | 2 | 2 | 2 | 1 | 0 | 0 |  | 3 | 23 | 0 | 7 | 0 |  |
| 8156 | 0 | 16.118999 | 5.2199998 | 32.384144 | 67.61586 | 0 | 11.983573 | 1 | -0.65196854 | 2 | 99.198441 | 19.046665 | 83.519997 | 1 | 5 | 3 | 0 | 0 | 0 | 0 | 0 | 0 |  | | NA |  | NA | 2 | 2 | 2 | 1 | 0 | 0 |  | 3 | 27 | 0 | 7 | 0 |  |
| 8166 | 0 | 17.926 | 5.679 | 31.680241 | 68.319756 | 0 | 9.6618757 | 1 | 0.56553632 | 4 | 99.350586 | 21.937222 | 74.859 | 1 | 0 | 5 | 0 | 0 | 0 | 0 | 0 | 0 |  | 127.107855 | 2.423493397 | 83.47185306 | -2.245564334 | 2 | 2 |  | 0 | 0 | 0 | 1 |  | | | | | |
| 8166 | 1 | 22.764 | 6.9299998 | 30.442804 | 69.557198 | 0 | 10.288843 | 1 | 0.56553632 | 4 | 99.350586 | 21.142776 | 78.730003 | 1 | 1 | 5 | 0 | 1 | 0 | 0 | 0 | 0 |  | 142.6510413 | 3.835413699 | 87.20203077 | -1.826973452 | 2 | 2 | 2 | 0 | 0 | 0 |  | | 27 | 0 | 7 | 0 |  |
| 8166 | 0 | 16.841 | 5.5599999 | 33.014668 | 66.985336 | 0 | 10.370978 | 1 | 0.56553632 | 4 | 99.350586 | 20.324444 | 80.970001 | 1 | 3 | 5 | 0 | 0 | 0 | 0 | 0 | 0 |  | | NA |  | NA | 2 | 2 | 2 | 0 | 0 | 0 |  | | 25 | 0 | 7 | 0 |  |
| 8166 | 0 | 18.646999 | 5.3299999 | 28.583687 | 71.416313 | 0 | 10.447639 | 1 | 0.56553632 | 4 | 99.350586 | 19.333891 | 81.629997 | 1 | 5 | 5 | 0 | 0 | 0 | 0 | 0 | 0 |  | | NA |  | NA | 2 | 2 | 2 | 0 | 0 | 0 |  | | 27 | 0 | 7 | 0 |  |
| 8166 | 0 | 20.341999 | 5.1500001 | 25.31708 | 74.682922 | 0 | 10.477755 | 1 | 0.56553632 | 4 | 99.350586 | 17.003332 | 86.040001 | 1 | 6 | 5 | 0 | 0 | 0 | 0 | 0 | 0 |  | 138.133473 | 3.439803712 | 87.80036323 | -1.756066357 | 2 | 2 | 2 | 0 | 0 | 0 | 0 |  | 27 | 0 | 7 | 0 |  |
| 8166 | 0 | 19.826 | 4.8299999 | 24.361948 | 75.638054 | 0 | 10.784394 | 1 | 0.56553632 | 4 | 99.350586 | 17.261665 | 83.059998 | 1 | 12 | 5 | 0 | 0 | 0 | 0 | 0 | 0 |  | 131.1033608 | 2.817763095 | 87.22578403 | -1.833259242 | 2 | 2 | 2 | 0 | 0 | 0 |  | | 27 | 0 | 7 | 0 |  |
| 8177 | 0 | 17.324 | 5.571 | 32.1577 | 67.8423 | 1 | 13.221082 | 0 | 1.9938918 | 3 | 120.16954 | 24.102777 | 75.014 | 1 | 0 | 3 | 1 | 0 | 0 | 0 | 0 | 0 | 0 | 138.8894022 | 3.314608236 | 94.6184194 | -0.872123483 | 2 | 2 |  | 0 | 0 | 0 | 0 | 1 |  | | | | |
| 8177 | 1 | 20.587 | 6.25 | 30.358965 | 69.641037 | 1 | 13.927447 | 0 | 1.9938918 | 3 | 120.16954 | 21.383331 | 77.849998 | 1 | 1 | 3 | 1 | 1 | 1 | 1 | 1 | 1 | 0 | 131.5473618 | 2.689639585 | 84.53453541 | -2.14918592 | 2 | 2 | 2 | 0 | 0 | 0 |  | 1 | 11 | 1 | 7 | 0 |  |
| 8177 | 1 | 14.731 | 4.9400001 | 33.534725 | 66.465279 | 1 | 14.116359 | 0 | 1.9938918 | 3 | 120.16954 | 18.312222 | 83.660004 | 1 | 6 | 3 | 1 | 1 | 0 | 1 | 0 | 1 | 0 | 137.1441222 | 3.150161017 | 87.7451126 | -1.783222931 | 2 | 2 | 2 | 0 | 0 | 0 | 0 | 1 | 15 | 1 | 3.3 | 1 |  |
| 8177 | 0 | 15.54 | 4.0999999 | 26.383526 | 73.616478 | 1 | 14.436687 | 0 | 1.9938918 | 3 | 120.16954 | 18.446114 | 82.019997 | 1 | 12 | 3 | 1 | 0 | 0 | 0 | 0 | 0 | 0 | 135.7302447 | 3.026757102 | 83.29479422 | -2.274225789 | 2 | 2 | 2 | 0 | 0 | 0 |  | 1 | 21 | 0 | 6 | 1 |  |
| 8178 | 1 | 17.722 | 5.801 | 32.733326 | 67.266678 | 1 | 16.334019 | 0 | 0.18182354 | 3 | 108.65639 | 23.213333 | 76.489 | 1 | 0 | 3 | 0 | 1 | 0 | 1 | 1 | 1 | 1 | 124.736381 | 2.089830065 | 93.21393862 | -1.047958585 |  | | | | | 0 | 0 | 3 |  | | | | |
| 8178 | 1 | 20.805 | 6.3800001 | 30.665707 | 69.334297 | 1 | 17.073238 | 0 | 0.18182354 | 3 | 108.65639 | 21.429998 | 77.589996 | 1 | 1 | 3 | 0 | 1 | 0 | 0 | 0 | 0 | 0 | 132.398586 | 2.71225179 | 91.10015428 | -1.326476134 |  | | 2 |  | | 0 |  | 3 | 25 | 0 | 7 | 0 |  |
| 8178 | 1 | 13.728 | 4.54 | 33.071095 | 66.928902 | 1 | 17.264887 | 0 | 0.18182354 | 3 | 108.65639 | 17.106667 | 86.389999 | 1 | 6 | 3 | 0 | 1 | 0 | 1 | 1 | 1 | 1 | 130.2577967 | 2.534865014 | 92.46670749 | -1.141494173 |  | | 2 |  | | 0 | 0 | 3 | 22 | 0 | 5.6999998 | 1 |  |
| 8178 | 0 | 15.637 | 4.1199999 | 26.347765 | 73.652237 | 1 | 17.571526 | 0 | 0.18182354 | 3 | 108.65639 | 18.448889 | 81.860001 | 1 | 12 | 3 | 0 | 0 | 0 | 0 | 0 | 0 | 0 | 122.9137782 | 1.927483025 | 92.57029802 | -1.123901247 |  | | 2 |  | | 0 |  | 3 | 25 | 0 | 7 | 0 |  |
| 8218 | 0 | 17.913 | 6.054 | 33.796684 | 66.203316 | 1 | 13.568789 | 1 | 2.4412034 | 4 | 100.24466 | 18.972778 | 79.108 | 1 | 0 | 4 | 0 | 0 | 0 | 0 | 0 | 0 | 0 | 120.5563498 | 1.875515121 | 89.35975854 | -1.526732665 |  | | | | | 0 | 0 | 3 |  | | | | |
| 8218 | 0 | 23.476 | 7.52 | 32.032715 | 67.967285 | 1 | 14.16564 | 1 | 2.4412034 | 4 | 100.24466 | 22.248888 | 74.519997 | 1 | 1 | 4 | 0 | 0 | 0 | 0 | 0 | 0 | 0 | 113.6454143 | 1.245629698 | 86.77476444 | -1.836038956 |  | | 2 |  | | 0 |  | 3 | 24 | 0 | 7 | 0 |  |
| 8218 | 1 | 15.852 | 5.2800002 | 33.308102 | 66.691902 | 1 | 14.321697 | 1 | 2.4412034 | 4 | 100.24466 | 19.673334 | 82.330002 | 1 | 5 | 4 | 0 | 1 | 0 | 0 | 0 | 1 | 0 |  | NA |  | NA |  | | 2 |  | | 0 |  | 3 | 24 | 0 | 7 | 0 |  |
| 8218 | 0 | 20.660999 | 4.7800002 | 23.135378 | 76.864624 | 1 | 14.365503 | 1 | 2.4412034 | 4 | 100.24466 | 18.05611 | 85.209999 | 1 | 6 | 4 | 0 | 0 | 0 | 0 | 0 | 0 | 0 | 125.7337785 | 2.341755832 | 87.14545922 | -1.78417164 |  | | 2 |  | | 0 | 0 | 3 | 24 | 0 | 7 | 0 |  |
| 8218 | 0 | 20.153999 | 4.1999998 | 20.839535 | 79.160461 | 1 | 14.65024 | 1 | 2.4412034 | 4 | 100.24466 | 17.763332 | 81.739998 | 1 | 12 | 4 | 0 | 0 | 0 | 0 | 0 | 0 | 0 | 133.0698801 | 3.002863068 | 90.13521652 | -1.397871731 |  | | 2 |  | | 0 |  | 3 | 24 | 0 | 7 | 0 |  |
| 8236 | 1 | 18.105 | 5.519 | 30.483292 | 69.516708 | 0 | 11.882273 | 0 | 1.9938918 | 2 | 108.27961 | 22.345556 | 75.066 | 1 | 0 | 5 | 0 | 1 | 0 | 1 | 0 | 1 |  | 109.5063008 | 0.835873934 | 105.7391532 | 1.11500582 | 2 | 2 |  | 1 | 0 | 0 | 0 | 3 |  | | | | |
| 8236 | 0 | 24.141001 | 6.5799999 | 27.256533 | 72.743469 | 1 | 12.594113 | 0 | 1.9938918 | 2 | 108.27961 | 21.757778 | 75.389999 | 1 | 1 | 5 | 0 | 0 | 0 | 0 | 0 | 0 | 0 | 100.3176334 | 0.028020362 | 92.93002612 | -1.122768798 | 2 | 2 | 2 | 1 | 0 | 0 |  | 3 | 23 | 0 | 6.5 | 1 |  |
| 8236 | 1 | 18.431999 | 4.98 | 27.01823 | 72.981766 | 1 | 12.670774 | 0 | 1.9938918 | 2 | 108.27961 | 20.522223 | 80.150002 | 1 | 3 | 5 | 0 | 1 | 0 | 1 | 0 | 1 | 0 |  | NA |  | NA | 2 | 2 | 2 | 1 | 0 | 0 |  | 3 | 24 | 0 | 7 | 0 |  |
| 8236 | 1 | 16.319 | 5.1500001 | 31.558308 | 68.441696 | 1 | 12.747434 | 0 | 1.9938918 | 2 | 108.27961 | 19.934446 | 79.379997 | 1 | 5 | 5 | 0 | 1 | 0 | 0 | 0 | 1 | 0 |  | NA |  | NA | 2 | 2 | 2 | 1 | 0 | 0 |  | 3 | 24 | 0 | 6.9000001 | 1 |  |
| 8236 | 0 | 17.663 | 5.02 | 28.420994 | 71.57901 | 1 | 12.785763 | 0 | 1.9938918 | 2 | 108.27961 | 17.593332 | 84.989998 | 1 | 6 | 5 | 0 | 0 | 0 | 0 | 0 | 0 | 0 | 100.6952781 | 0.061234302 | 89.07995639 | -1.633900893 | 2 | 2 | 2 | 1 | 0 | 0 | 0 | 3 | 24 | 0 | 7 | 0 |  |
| 8236 | 1 | 18.405001 | 4.4000001 | 23.906546 | 76.093452 | 1 | 13.089664 | 0 | 1.9938918 | 2 | 108.27961 | 18.099445 | 80.18 | 1 | 12 | 5 | 0 | 1 | 1 | 1 | 1 | 0 | 1 | 97.26310618 | -0.241281267 | 96.7357037 | -0.546530442 | 2 | 2 | 2 | 1 | 0 | 0 |  | 3 | 24 | 0 | 7 | 0 |  |
| 8246 | 1 | 18.322 | 5.561 | 30.35149 | 69.648514 | 1 | 14.26694 | 0 | 0.42763337 | 2 | 104.88055 | 22.845556 | 74.917 | 1 | 0 | 3 | 0 | 1 | 1 | 1 | 1 | 1 | 1 | 110.1245399 | 0.875600754 | 102.0519224 | 0.366410961 | 2 | 2 |  | 0 | 0 | 0 | 0 | 4 |  | | | | |
| 8246 | 0 | 15.668 | 5 | 31.912178 | 68.087822 | 1 | 15.151266 | 0 | 0.42763337 | 2 | 104.88055 | 19.988331 | 78.849998 | 1 | 5 | 3 | 0 | 0 | 0 | 0 | 0 | 0 | 0 |  | NA |  | NA | 2 | 2 | 2 | 0 | 0 | 0 |  | 4 | 24 | 0 | 7 | 0 |  |
| 8246 | 0 | 16.632999 | 4.9200001 | 29.579754 | 70.42025 | 1 | 15.189596 | 0 | 0.42763337 | 2 | 104.88055 | 17.577778 | 84.790001 | 1 | 6 | 3 | 0 | 0 | 0 | 0 | 0 | 0 | 0 | 115.65175 | 1.339865613 | 103.3530258 | 0.602675584 | 2 | 2 | 2 | 0 | 0 | 0 | 0 | 4 | 24 | 0 | 7 | 0 |  |
| 8246 | 0 | 18.398001 | 4.25 | 23.100336 | 76.899666 | 1 | 15.493498 | 0 | 0.42763337 | 2 | 104.88055 | 18.134443 | 79.940002 | 1 | 12 | 3 | 0 | 0 | 0 | 0 | 0 | 0 | 0 | 108.315402 | 0.714839404 | 106.9383954 | 1.304300156 | 2 | 2 | 2 | 0 | 0 | 0 |  | 4 | 24 | 0 | 7 | 0 |  |
| 8253 | 1 | 18.044 | 5.41 | 29.982265 | 70.017731 | 1 | 16.914442 | 1 | 2.5048022 | 4 | 93.140427 | 22.797777 | 74.232 | 1 | 0 | 4 | 1 | 1 | 1 | 1 | 0 | 1 | 0 | 111.439084 | 1.040362793 | 89.13037969 | -1.475318792 | 2 | 2 |  | 1 | 1 | 0 | 0 |  | | | | | |
| 8253 | 0 | 24.370001 | 6.46 | 26.508001 | 73.491997 | 1 | 17.612595 | 1 | 2.5048022 | 4 | 93.140427 | 21.711668 | 75.5 | 1 | 1 | 4 | 1 | 0 | 0 | 0 | 0 | 0 | 0 | 121.0262764 | 1.906923017 | 88.95333948 | -1.486094191 | 2 | 2 | 2 | 1 | 1 | 0 |  | | 24 | 0 | 7 | 0 |  |
| 8253 | 0 | 18.080999 | 4.9200001 | 27.210886 | 72.789116 | 1 | 17.686516 | 1 | 2.5048022 | 4 | 93.140427 | 20.505558 | 80.160004 | 1 | 3 | 4 | 1 | 0 | 0 | 0 | 0 | 0 | 0 |  | NA |  | NA | 2 | 2 | 2 | 1 | 1 | 0 |  | | 24 | 0 | 7 | 0 |  |
| 8253 | 0 | 16.252001 | 5.1300001 | 31.565344 | 68.434654 | 1 | 17.763176 | 1 | 2.5048022 | 4 | 93.140427 | 19.953888 | 79.260002 | 1 | 5 | 4 | 1 | 0 | 0 | 0 | 0 | 0 | 0 |  | NA |  | NA | 2 | 2 | 2 | 1 | 1 | 0 |  | | 24 | 0 | 6.9000001 | 1 |  |
| 8253 | 0 | 18.282 | 4.3800001 | 23.957993 | 76.042007 | 1 | 18.105408 | 1 | 2.5048022 | 4 | 93.140427 | 18.105553 | 80.089996 | 1 | 12 | 4 | 1 | 0 | 0 | 0 | 0 | 0 | 0 | 118.4742677 | 1.675778477 | 85.82057188 | -1.851279742 | 2 | 2 | 2 | 1 | 1 | 0 |  | | 24 | 0 | 7 | 0 |  |
| 8254 | 0 | 18.351 | 5.62 | 30.625034 | 69.374969 | 1 | 15.186858 | 1 | 2.4412034 | 3 | 108.31396 | 22.602222 | 75.293 | 1 | 0 | 5 | 1 | 0 | 0 | 0 | 0 | 0 | 0 | 129.0410863 | 2.636752132 | 88.26069072 | -1.621883568 |  | | | | | 0 | 0 | 1 |  | | | | |
| 8254 | 0 | 23.780001 | 6.1500001 | 25.862068 | 74.137932 | 1 | 15.934292 | 1 | 2.4412034 | 3 | 108.31396 | 21.547224 | 76 | 1 | 1 | 5 | 1 | 0 | 0 | 0 | 0 | 0 | 0 | 118.5097061 | 1.682593391 | 91.71219785 | -1.164496469 |  | | 2 |  | | 0 |  | 1 | 18 | 1 | 6 | 1 |  |
| 8254 | 0 | 18.927999 | 4.73 | 24.989435 | 75.010567 | 1 | 16 | 1 | 2.4412034 | 3 | 108.31396 | 20.537779 | 79.620003 | 1 | 3 | 5 | 1 | 0 | 0 | 0 | 0 | 0 | 0 |  | NA |  | NA |  | | 2 |  | | 0 |  | 1 | 21 | 0 | 7 | 0 |  |
| 8254 | 0 | 15.822 | 5.0300002 | 31.791178 | 68.208824 | 1 | 16.07666 | 1 | 2.4412034 | 3 | 108.31396 | 19.994444 | 78.889999 | 1 | 5 | 5 | 1 | 0 | 0 | 0 | 0 | 0 | 0 |  | NA |  | NA |  | | 2 |  | | 0 |  | 1 | 24 | 0 | 7 | 0 |  |
| 8254 | 0 | 18.096001 | 5.02 | 27.740936 | 72.259064 | 1 | 16.128679 | 1 | 2.4412034 | 3 | 108.31396 | 17.358889 | 84.709999 | 1 | 6 | 5 | 1 | 0 | 0 | 0 | 0 | 0 | 0 | 115.438997 | 1.404074909 | 93.46880931 | -0.928350503 |  | | 2 |  | | 0 | 0 | 1 | 22 | 0 | 6.9000001 | 1 |  |
| 8254 | 0 | 18.288 | 4.2800002 | 23.403326 | 76.596672 | 1 | 16.421629 | 1 | 2.4412034 | 3 | 108.31396 | 18.129446 | 79.93 | 1 | 12 | 5 | 1 | 0 | 0 | 0 | 0 | 0 | 0 | 113.7836753 | 1.253579952 | 98.06598395 | -0.284109801 |  | | 2 |  | | 0 |  | 1 | 24 | 0 | 7 | 0 |  |
| 8261 | 0 | 18.261 | 5.505 | 30.146214 | 69.85379 | 0 | 10.90486 | 0 | 0.26426548 | 3 | 115.85738 | 22.742222 | 73.835 | 1 | 0 | 5 | 1 | 0 | 0 | 0 | 0 | 0 |  | 125.8854112 | 2.25599295 | 100.070915 | 0.012750812 | 2 | 2 |  | 0 | 0 | 0 | 0 | 4 |  | | | | |
| 8261 | 1 | 23.886 | 6.9299998 | 29.01281 | 70.98719 | 0 | 11.556468 | 0 | 0.26426548 | 3 | 115.85738 | 21.838888 | 75.18 | 1 | 1 | 5 | 1 | 1 | 0 | 0 | 0 | 0 |  | 137.8748212 | 3.264891214 | 98.08724514 | -0.331528444 | 2 | 2 | 2 | 0 | 0 | 0 |  | 4 | 22 | 0 | 6.8000002 | 1 |  |
| 8261 | 1 | 19.073999 | 5.1900001 | 27.209816 | 72.790184 | 0 | 11.635866 | 0 | 0.26426548 | 3 | 115.85738 | 20.537779 | 80.309998 | 1 | 3 | 5 | 1 | 1 | 0 | 0 | 0 | 0 |  | | NA |  | NA | 2 | 2 | 2 | 0 | 0 | 0 |  | 4 | 24 | 0 | 6.9000001 | 1 |  |
| 8261 | 0 | 16.242001 | 5.2600002 | 32.385174 | 67.61483 | 0 | 11.712525 | 0 | 0.26426548 | 3 | 115.85738 | 19.751667 | 80.339996 | 1 | 5 | 5 | 1 | 0 | 0 | 0 | 0 | 0 |  | | NA |  | NA | 2 | 2 | 2 | 0 | 0 | 0 |  | 4 | 21 | 0 | 6 | 1 |  |
| 8261 | 1 | 17.830999 | 5.0599999 | 28.377546 | 71.622452 | 0 | 11.745379 | 0 | 0.26426548 | 3 | 115.85738 | 17.973886 | 84.120003 | 1 | 6 | 5 | 1 | 1 | 1 | 0 | 0 | 0 |  | 135.9445572 | 3.099716197 | 99.29720696 | -0.123901177 | 2 | 2 | 2 | 0 | 0 | 0 | 0 | 4 | 23 | 0 | 7 | 0 |  |
| 8261 | 0 | 18.934999 | 4.4699998 | 23.607077 | 76.392921 | 1 | 12.07666 | 0 | 0.26426548 | 3 | 115.85738 | 18.027777 | 80.599998 | 1 | 12 | 5 | 1 | 0 | 0 | 0 | 0 | 0 | 0 | 129.58334 | 2.557870658 | 99.2837767 | -0.125839373 | 2 | 2 | 2 | 0 | 0 | 0 |  | 4 | 25 | 0 | 7 | 0 |  |
| 8283 | 1 | 21.98 | 6.656 | 30.282074 | 69.717926 | 0 | 9.6262836 | 1 | 0.59089935 | 4 | 108.28087 | 20.911667 | 77.306 | 1 | 0 | 5 | 1 | 1 | 1 | 1 | 1 | 1 |  | 128.4095272 | 2.537536546 | 89.59860659 | -1.507434233 | 2 | 2 |  | 1 | 0 | 0 | 0 | 2 |  | | | | |
| 8283 | 1 | 24.292 | 8.2200003 | 33.838303 | 66.161697 | 0 | 10.10267 | 1 | 0.59089935 | 4 | 108.28087 | 22.061666 | 74.660004 | 1 | 1 | 5 | 1 | 0 | 0 | 1 | 1 | 1 |  | 97.89836462 | -0.190487317 | 93.37359855 | -1.00518889 | 2 | 2 | 2 | 1 | 0 | 0 |  | 2 | 20 | 0 | 7 | 0 |  |
| 8283 | 1 | 16.909 | 5.79 | 34.242119 | 65.757881 | 0 | 10.182067 | 1 | 0.59089935 | 4 | 108.28087 | 20.436668 | 81.639999 | 1 | 3 | 5 | 1 | 0 | 0 | 1 | 1 | 1 |  | | NA |  | NA | 2 | 2 | 2 | 1 | 0 | 0 |  | 2 | 27 | 0 | 7 | 0 |  |
| 8283 | 1 | 17.488001 | 5.6300001 | 32.193504 | 67.806496 | 0 | 10.261465 | 1 | 0.59089935 | 4 | 108.28087 | 19.593887 | 81.779999 | 1 | 5 | 5 | 1 | 1 | 0 | 1 | 1 | 0 |  | | NA |  | NA | 2 | 2 | 2 | 1 | 0 | 0 |  | 2 | 21 | 0 | 6.9000001 | 1 |  |
| 8283 | 1 | 21.368 | 5.4200001 | 25.365032 | 74.634964 | 0 | 10.294319 | 1 | 0.59089935 | 4 | 108.28087 | 17.708889 | 85.470001 | 1 | 6 | 5 | 1 | 1 | 1 | 1 | 1 | 0 |  | 123.2301089 | 2.097784932 | 93.85214314 | -0.938873435 | 2 | 2 | 2 | 1 | 0 | 0 | 0 | 2 | 26 | 0 | 6.5999999 | 1 |  |
| 8283 | 0 | 22.091 | 5.1799998 | 23.448462 | 76.551537 | 0 | 10.622861 | 1 | 0.59089935 | 4 | 108.28087 | 18.304998 | 80.260002 | 1 | 12 | 5 | 1 | 0 | 0 | 0 | 0 | 0 |  | 122.9922809 | 2.083688085 | 93.27492755 | -1.023661963 | 2 | 2 | 2 | 1 | 0 | 0 |  | 2 | 27 | 0 | 7 | 0 |  |
| 8296 | 1 | 17.757 | 5.561 | 31.317226 | 68.68277 | 0 | 11.411362 | 0 | -0.20840913 | 3 | 115.84841 | 20.763889 | 75.574 | 1 | 0 | 5 | 1 | 1 | 1 | 1 | 1 | 0 | 1 | 124.9068157 | 2.168787114 | 99.01847236 | -0.172826332 | 2 | 2 |  | 0 | 0 | 0 | 0 |  | | | | | |
| 8296 | 0 | 24.356001 | 6.4099998 | 26.317949 | 73.682053 | 1 | 12.082135 | 0 | -0.20840913 | 3 | 115.84841 | 21.140556 | 78.449997 | 1 | 1 | 5 | 1 | 0 | 0 | 0 | 0 | 0 | 0 | 128.8258935 | 2.492329397 | 100.8464715 | 0.15216599 | 2 | 2 | 2 | 0 | 0 | 0 |  | | 24 | 0 | 7 | 0 |  |
| 8296 | 0 | 15.815 | 5.0599999 | 31.994942 | 68.005058 | 1 | 12.158795 | 0 | -0.20840913 | 3 | 115.84841 | 20.376667 | 80.290001 | 1 | 3 | 5 | 1 | 0 | 0 | 0 | 0 | 0 | 0 |  | NA |  | NA | 2 | 2 | 2 | 0 | 0 | 0 |  | | 24 | 0 | 7 | 0 |  |
| 8296 | 0 | 18.841999 | 4.9299998 | 26.164951 | 73.835052 | 1 | 12.235456 | 0 | -0.20840913 | 3 | 115.84841 | 19.406666 | 80.839996 | 1 | 5 | 5 | 1 | 0 | 0 | 0 | 0 | 0 | 0 |  | NA |  | NA | 2 | 2 | 2 | 0 | 0 | 0 |  | | 24 | 0 | 7 | 0 |  |
| 8296 | 0 | 19.268 | 5.27 | 27.351048 | 72.648949 | 1 | 12.273785 | 0 | -0.20840913 | 3 | 115.84841 | 17.028889 | 85.779999 | 1 | 6 | 5 | 1 | 0 | 0 | 0 | 0 | 0 | 0 | 135.7885779 | 3.076681691 | 98.89707706 | -0.1922786 | 2 | 2 | 2 | 0 | 0 | 0 | 0 |  | 23 | 0 | 7 | 0 |  |
| 8296 | 0 | 18.101999 | 4.8400002 | 26.737379 | 73.262619 | 1 | 12.555783 | 0 | -0.20840913 | 3 | 115.84841 | 17.392776 | 82.550003 | 1 | 12 | 5 | 1 | 0 | 0 | 0 | 0 | 0 | 0 | 134.6332854 | 2.97421254 | 95.51179883 | -0.741398689 | 2 | 2 | 2 | 0 | 0 | 0 |  | | 19 | 1 | 7 | 0 |  |
| 8300 | 1 | 19.303 | 5.168 | 26.773041 | 73.226959 | 1 | 13.158111 | 1 | 1.8494691 | 2 | 107.88025 | 25.178333 | 70.826 | 1 | 0 | 5 | 1 | 1 | 1 | 1 | 1 | 0 | 1 | 116.2056891 | 1.480920777 | 97.96430765 | -0.3172225 |  | | | | | 0 | 0 |  | | | | | |
| 8300 | 1 | 24.452 | 6.4099998 | 26.214624 | 73.785378 | 1 | 13.771389 | 1 | 1.8494691 | 2 | 107.88025 | 21.567223 | 76.559998 | 1 | 1 | 5 | 1 | 1 | 0 | 0 | 0 | 1 | 0 | 119.1835513 | 1.750101991 | 99.2903434 | -0.11055214 |  | | 2 |  | | 0 |  | | 24 | 0 | 7 | 0 |  |
| 8300 | 1 | 17.348 | 4.9200001 | 28.360619 | 71.639381 | 1 | 13.853525 | 1 | 1.8494691 | 2 | 107.88025 | 20.616667 | 79.599998 | 1 | 3 | 5 | 1 | 1 | 0 | 0 | 0 | 1 | 0 |  | NA |  | NA |  | | 2 |  | | 0 |  | | 25 | 0 | 7 | 0 |  |
| 8300 | 0 | 34.875999 | 5.0999999 | 14.623237 | 85.376762 | 1 | 13.930184 | 1 | 1.8494691 | 2 | 107.88025 | 19.843334 | 79.769997 | 1 | 5 | 5 | 1 | 0 | 0 | 0 | 0 | 0 | 0 |  | NA |  | NA |  | | 2 |  | | 0 |  | | 23 | 0 | 6.9000001 | 1 |  |
| 8300 | 0 | 22.917 | 5.0700002 | 22.123316 | 77.876686 | 1 | 13.965776 | 1 | 1.8494691 | 2 | 107.88025 | 17.395 | 84.309998 | 1 | 6 | 5 | 1 | 0 | 0 | 0 | 0 | 0 | 0 | 122.2279025 | 2.025807063 | 99.21024757 | -0.122466028 |  | | 2 |  | | 0 | 0 |  | 24 | 0 | 7 | 0 |  |
| 8300 | 0 | 16.707001 | 4.2399998 | 25.378582 | 74.621422 | 1 | 14.277892 | 1 | 1.8494691 | 2 | 107.88025 | 17.197779 | 83.059998 | 1 | 12 | 5 | 1 | 0 | 0 | 0 | 0 | 0 | 0 | 120.844066 | 1.899192443 | 99.98530614 | -0.002279017 |  | | 2 |  | | 0 |  | | 24 | 0 | 7 | 0 |  |
| 8308 | 0 | 18.162 | 5.661 | 31.169476 | 68.830528 | 0 | 10.028748 | 0 | -0.63312542 | 2 | 75.285286 | 23.353889 | 74.609 | 1 | 0 | 5 | 1 | 0 | 0 | 0 | 0 | 0 |  | 78.71010473 | -1.931426581 | 102.4574066 | 0.462501416 | 2 | 2 |  | 0 | 0 | 1 | 0 | 2 |  | | | | |
| 8308 | 0 | 24.066999 | 7.4499998 | 30.95525 | 69.044746 | 0 | 10.639288 | 0 | -0.63312542 | 2 | 75.285286 | 21.592775 | 77.330002 | 1 | 1 | 5 | 1 | 0 | 0 | 0 | 0 | 0 |  | 93.42884569 | -0.588495753 | 104.3274459 | 0.834572614 | 2 | 2 | 2 | 0 | 0 | 1 |  | 2 | 27 | 0 | 7 | 0 |  |
| 8308 | 0 | 16.629 | 5.6999998 | 34.277466 | 65.722534 | 0 | 10.71321 | 0 | -0.63312542 | 2 | 75.285286 | 20.530001 | 81.860001 | 1 | 3 | 5 | 1 | 0 | 0 | 0 | 0 | 0 |  | | NA |  | NA | 2 | 2 | 2 | 0 | 0 | 1 |  | 2 | 27 | 0 | 7 | 0 |  |
| 8308 | 1 | 17.486 | 5.6599998 | 32.368752 | 67.631248 | 0 | 10.78987 | 0 | -0.63312542 | 2 | 75.285286 | 19.858891 | 81.169998 | 1 | 5 | 5 | 1 | 1 | 0 | 0 | 0 | 0 |  | | NA |  | NA | 2 | 2 | 2 | 0 | 0 | 1 |  | 2 | 24 | 0 | 6.9000001 | 1 |  |
| 8308 | 0 | 19.271999 | 5.4499998 | 28.279369 | 71.720627 | 0 | 10.819986 | 0 | -0.63312542 | 2 | 75.285286 | 18.061668 | 85.519997 | 1 | 6 | 5 | 1 | 0 | 0 | 0 | 0 | 0 |  | 96.29015583 | -0.331336394 | 98.32840159 | -0.292540441 | 2 | 2 | 2 | 0 | 0 | 1 | 0 | 2 | 22 | 0 | 7 | 0 |  |
| 8308 | 0 | 19.23 | 4.52 | 23.50494 | 76.495056 | 0 | 11.115674 | 0 | -0.63312542 | 2 | 75.285286 | 17.861668 | 82.269997 | 1 | 12 | 5 | 1 | 0 | 0 | 0 | 0 | 0 |  | 92.24810698 | -0.694447002 | 101.2525424 | 0.22901751 | 2 | 2 | 2 | 0 | 0 | 1 |  | 2 | 27 | 0 | 7 | 0 |  |
| 8316 | 0 | 15.909 | 5.418 | 34.056194 | 65.943802 | 0 | 11.211499 | 1 | 2.2397017 | 4 | 87.238472 | 18.251667 | 79.186 | 1 | 0 | 4 | 0 | 0 | 0 | 0 | 0 | 0 |  | 94.31720988 | -0.520878885 | 98.55864031 | -0.231080466 | 2 | 2 |  | 0 | 0 | 0 | 0 | 3 |  | | | | |
| 8316 | 1 | 22.281 | 7.1700001 | 32.179886 | 67.820114 | 0 | 11.843943 | 1 | 2.2397017 | 4 | 87.238472 | 20.817223 | 79.110001 | 1 | 1 | 4 | 0 | 1 | 1 | 0 | 0 | 0 |  | 100.4168303 | 0.038232128 | 95.95448174 | -0.630694723 | 2 | 2 | 2 | 0 | 0 | 0 |  | 3 | 27 | 0 | 7 | 0 |  |
| 8316 | 1 | 15.694 | 4.8400002 | 30.839813 | 69.160187 | 0 | 11.917865 | 1 | 2.2397017 | 4 | 87.238472 | 20.05722 | 82.760002 | 1 | 3 | 4 | 0 | 1 | 0 | 0 | 0 | 0 |  | | NA |  | NA | 2 | 2 | 2 | 0 | 0 | 0 |  | 3 | 27 | 0 | 7 | 0 |  |
| 8316 | 0 | 16.785 | 5.4499998 | 32.469467 | 67.530533 | 0 | 11.994524 | 1 | 2.2397017 | 4 | 87.238472 | 19.156113 | 83.32 | 1 | 5 | 4 | 0 | 0 | 0 | 0 | 0 | 0 |  | | NA |  | NA | 2 | 2 | 2 | 0 | 0 | 0 |  | 3 | 25 | 0 | 6.9000001 | 1 |  |
| 8316 | 0 | 17.364 | 4.9299998 | 28.392075 | 71.607925 | 1 | 12.041068 | 1 | 2.2397017 | 4 | 87.238472 | 17.885555 | 84.940002 | 1 | 6 | 4 | 0 | 0 | 0 | 0 | 0 | 0 | 0 | 102.5466977 | 0.233541878 | 95.63838694 | -0.676805625 | 2 | 2 | 2 | 0 | 0 | 0 | 0 | 3 | 19 | 1 | 6.8000002 | 1 |  |
| 8353 | 1 | 16.818 | 5.523 | 32.839813 | 67.160187 | 1 | 15.523614 | 0 | -0.63541806 | 4 | 109.37119 | 20.032778 | 76.896 | 1 | 0 | 5 | 0 | 1 | 1 | 1 | 1 | 1 | 0 | 128.1691589 | 2.383746051 | 91.53873115 | -1.286218004 | 2 | 2 |  | 0 | 0 | 0 | 0 | 3 |  | | | | |
| 8353 | 0 | 23.261 | 7.4200001 | 31.898888 | 68.101112 | 1 | 16.142368 | 0 | -0.63541806 | 4 | 109.37119 | 21.434999 | 77.360001 | 1 | 1 | 5 | 0 | 0 | 0 | 0 | 0 | 0 | 0 | 120.1501756 | 1.710007782 | 94.21770537 | -0.906918315 | 2 | 2 | 2 | 0 | 0 | 0 |  | 3 | 21 | 0 | 6.9000001 | 1 |  |
| 8353 | 0 | 16.457001 | 5.21 | 31.658258 | 68.341743 | 1 | 16.219028 | 0 | -0.63541806 | 4 | 109.37119 | 20.33889 | 82.040001 | 1 | 3 | 5 | 0 | 0 | 0 | 0 | 0 | 0 | 0 |  | NA |  | NA | 2 | 2 | 2 | 0 | 0 | 0 |  | 3 | 25 | 0 | 7 | 0 |  |
| 8353 | 0 | 16.693001 | 5.4000001 | 32.348888 | 67.651115 | 1 | 16.295689 | 0 | -0.63541806 | 4 | 109.37119 | 19.377222 | 82.879997 | 1 | 5 | 5 | 0 | 0 | 0 | 0 | 0 | 0 | 0 |  | NA |  | NA | 2 | 2 | 2 | 0 | 0 | 0 |  | 3 | 24 | 0 | 7 | 0 |  |
| 8353 | 1 | 18.598 | 5.04 | 27.099688 | 72.900314 | 1 | 16.342232 | 0 | -0.63541806 | 4 | 109.37119 | 18.078333 | 84.610001 | 1 | 6 | 5 | 0 | 1 | 1 | 0 | 1 | 1 | 0 | 118.4279024 | 1.564366411 | 94.79443467 | -0.821297922 | 2 | 2 | 2 | 0 | 0 | 0 | 0 | 3 | 24 | 0 | 7 | 0 |  |
| 8353 | 0 | 20.736 | 4.6300001 | 22.328318 | 77.671684 | 1 | 16.626968 | 0 | -0.63541806 | 4 | 109.37119 | 17.482779 | 81.220001 | 1 | 12 | 5 | 0 | 0 | 0 | 0 | 0 | 0 | 0 | 121.1204135 | 1.78675182 | 95.41410066 | -0.727502605 | 2 | 2 | 2 | 0 | 0 | 0 |  | 3 | 22 | 0 | 7 | 0 |  |
| 8389 |  | 19.084 | 5.844 | 30.622511 | 69.377487 | 0 | 11.570157 | 0 | -0.026198076 | 3 | 104.87643 | 24.891666 | 71.32 | 1 | 0 | 5 | 1 |  | | | | | | 103.0604122 | 0.271024994 | 108.2745288 | 1.678010818 | 2 | 2 |  | 1 | 0 | 0 | 0 |  | | | | | |
| 8389 | 0 | 23.337999 | 6.6100001 | 28.322908 | 71.677094 | 1 | 12.191649 | 0 | -0.026198076 | 3 | 104.87643 | 20.868336 | 80.870003 | 1 | 1 | 5 | 1 | 0 | 0 | 0 | 0 | 0 | 0 | 107.1116129 | 0.625373047 | 107.8632617 | 1.570891204 | 2 | 2 | 2 | 1 | 0 | 0 |  | | 23 | 0 | 7 | 0 |  |
| 8389 | 0 | 20.975 | 6.1399999 | 29.272943 | 70.727058 | 1 | 12.271048 | 0 | -0.026198076 | 3 | 104.87643 | 20.423334 | 79.349998 | 1 | 3 | 5 | 1 | 0 | 0 | 0 | 0 | 0 | 0 |  | NA |  | NA | 2 | 2 | 2 | 1 | 0 | 0 |  | | 24 | 0 | 7 | 0 |  |
| 8389 | 0 | 17.284 | 5.1999998 | 30.085627 | 69.914375 | 1 | 12.344969 | 0 | -0.026198076 | 3 | 104.87643 | 19.707777 | 80.43 | 1 | 5 | 5 | 1 | 0 | 0 | 0 | 0 | 0 | 0 |  | NA |  | NA | 2 | 2 | 2 | 1 | 0 | 0 |  | | 24 | 0 | 7 | 0 |  |
| 8389 | 1 | 23.492001 | 5.4499998 | 23.199385 | 76.800613 | 1 | 12.402464 | 0 | -0.026198076 | 3 | 104.87643 | 17.215555 | 84.690002 | 1 | 6 | 5 | 1 | 1 | 0 | 0 | 0 | 1 | 0 | 93.45486372 | -0.581720624 | 107.3484047 | 1.452446242 | 2 | 2 | 2 | 1 | 0 | 0 | 0 |  | 24 | 0 | 7 | 0 |  |
| 8389 | 0 | 20.011 | 4.5100002 | 22.537605 | 77.462395 | 1 | 12.692677 | 0 | -0.026198076 | 3 | 104.87643 | 18.526667 | 78.779999 | 1 | 12 | 5 | 1 | 0 | 0 | 0 | 0 | 0 | 0 | 106.0921343 | 0.534392029 | 109.5048378 | 1.931300966 | 2 | 2 | 2 | 1 | 0 | 0 |  | | 25 | 0 | 7 | 0 |  |
| 8403 | 1 | 19.193 | 5.747 | 29.943209 | 70.056793 | 0 | 9.3634501 | 1 | 2.2397017 | 4 | 111.00166 | 21.309999 | 75.289 | 1 | 0 | 5 | 1 | 1 | 0 | 1 | 1 | 0 |  | 128.4173018 | 2.525797441 | 92.37729067 | -1.132980718 | 2 | 2 |  | 0 | 0 | 0 | 0 | 4 |  | | | | |
| 8403 | 1 | 24.122999 | 7.48 | 31.007753 | 68.992249 | 0 | 9.9603014 | 1 | 2.2397017 | 4 | 111.00166 | 21.754442 | 75.839996 | 1 | 1 | 5 | 1 | 1 | 0 | 0 | 0 | 0 |  | 125.1830191 | 2.263086473 | 92.17231084 | -1.171048551 | 2 | 2 | 2 | 0 | 0 | 0 |  | 4 | 25 | 0 | 6.9000001 | 1 |  |
| 8403 | 1 | 18.129 | 5.6700001 | 31.275856 | 68.724144 | 0 | 10.036961 | 1 | 2.2397017 | 4 | 111.00166 | 20.451109 | 81.209999 | 1 | 3 | 5 | 1 | 1 | 0 | 0 | 0 | 0 |  | | NA |  | NA | 2 | 2 | 2 | 0 | 0 | 0 |  | 4 | 27 | 0 | 7 | 0 |  |
| 8403 | 1 | 16.702 | 5.3800001 | 32.211712 | 67.788292 | 0 | 10.116359 | 1 | 2.2397017 | 4 | 111.00166 | 19.554443 | 81.419998 | 1 | 5 | 5 | 1 | 1 | 0 | 0 | 0 | 0 |  | | NA |  | NA | 2 | 2 | 2 | 0 | 0 | 0 |  | 4 | 27 | 0 | 7 | 0 |  |
| 8403 | 1 | 18.662001 | 5.2600002 | 28.185617 | 71.814384 | 0 | 10.149213 | 1 | 2.2397017 | 4 | 111.00166 | 17.963888 | 84.440002 | 1 | 6 | 5 | 1 | 1 | 0 | 0 | 0 | 0 |  | 138.5729394 | 3.465380796 | 91.4870638 | -1.26748336 | 2 | 2 | 2 | 0 | 0 | 0 | 0 | 4 | 24 | 0 | 7 | 0 |  |
| 8403 | 0 | 19.635 | 4.6900001 | 23.885918 | 76.114082 | 0 | 10.464066 | 1 | 2.2397017 | 4 | 111.00166 | 18.030556 | 81.129997 | 1 | 12 | 5 | 1 | 0 | 0 | 0 | 0 | 0 |  | 116.640261 | 1.507898612 | 88.93583974 | -1.610758739 | 2 | 2 | 2 | 0 | 0 | 0 |  | 4 | 27 | 0 | 7 | 0 |  |
| 8410 |  | 17.822 | 5.297 | 29.721693 | 70.278305 | 1 | 16.142368 | 0 | 1.9938918 | 2 | 93.257011 | 19.97611 | 75.612 | 1 | 0 | 4 | 1 |  | | | | | | 102.9858127 | 0.256967127 | 96.79877042 | -0.520083819 | 2 | 2 |  | 0 | 0 | 0 | 0 |  | | | | | |
| 8410 | 1 | 23.389 | 7.3699999 | 31.510538 | 68.489464 | 1 | 16.73922 | 0 | 1.9938918 | 2 | 93.257011 | 21.913334 | 75.599998 | 1 | 1 | 4 | 1 | 1 | 1 | 1 | 1 | 1 | 1 | 103.9555858 | 0.33916583 | 98.1031004 | -0.31158139 | 2 | 2 | 2 | 0 | 0 | 0 |  | | 24 | 0 | 7 | 0 |  |
| 8410 | 1 | 15.377 | 5.0999999 | 33.166416 | 66.83358 | 1 | 16.804928 | 0 | 1.9938918 | 2 | 93.257011 | 20.458887 | 81.989998 | 1 | 3 | 4 | 1 | 1 | 1 | 1 | 1 | 1 | 1 |  | NA |  | NA | 2 | 2 | 2 | 0 | 0 | 0 |  | | 25 | 0 | 7 | 0 |  |
| 8410 | 1 | 22.219 | 4.7399998 | 21.333092 | 78.666908 | 1 | 16.925394 | 0 | 1.9938918 | 2 | 93.257011 | 17.729443 | 85.959999 | 1 | 6 | 4 | 1 | 1 | 1 | 1 | 1 | 1 | 1 | 100.6783263 | 0.058252012 | 97.5696942 | -0.395150736 | 2 | 2 | 2 | 0 | 0 | 0 | 0 |  | 24 | 0 | 5.3000002 | 1 |  |
| 8410 | 0 | 20.450001 | 4.2199998 | 20.635695 | 79.364304 | 1 | 17.251198 | 0 | 1.9938918 | 2 | 93.257011 | 17.772223 | 81.839996 | 1 | 12 | 4 | 1 | 0 | 0 | 0 | 0 | 0 | 0 | 103.5055193 | 0.299987506 | 95.35678109 | -0.731222203 | 2 | 2 | 2 | 0 | 0 | 0 |  | | 24 | 0 | 7 | 0 |  |
| 8430 | 1 | 18.622 | 5.561 | 29.862528 | 70.137474 | 1 | 14.562629 | 1 | 1.5539941 | 4 | 106.61742 | 19.776667 | 75.929 | 1 | 0 | 5 |  | 1 | 1 | 1 | 0 | 1 | 0 | 125.0745209 | 2.281258611 | 91.30958209 | -1.245951301 | 2 | 2 |  | 1 | 0 | 1 | 0 | 3 |  | | | | |
| 8430 | 0 | 25.181999 | 6.5100002 | 25.851801 | 74.148201 | 1 | 15.167693 | 1 | 1.5539941 | 4 | 106.61742 | 21.099442 | 78.660004 | 1 | 1 | 5 |  | 0 | 0 | 0 | 0 | 0 | 0 |  | NA |  | NA | 2 | 2 | 2 | 1 | 0 | 1 |  | 3 | 24 | 0 | 7 | 0 |  |
| 8430 | 0 | 15.093 | 4.9899998 | 33.061684 | 66.938316 | 1 | 15.244353 | 1 | 1.5539941 | 4 | 106.61742 | 20.330002 | 81.019997 | 1 | 3 | 5 |  | 0 | 0 | 0 | 0 | 0 | 0 |  | NA |  | NA | 2 | 2 | 2 | 1 | 0 | 1 |  | 3 | 25 | 0 | 7 | 0 |  |
| 8430 | 0 | 17.714001 | 5.0300002 | 28.395618 | 71.604378 | 1 | 15.321013 | 1 | 1.5539941 | 4 | 106.61742 | 19.583334 | 80.790001 | 1 | 5 | 5 |  | 0 | 0 | 0 | 0 | 0 | 0 |  | NA |  | NA | 2 | 2 | 2 | 1 | 0 | 1 |  | 3 | 24 | 0 | 7 | 0 |  |
| 8430 | 0 | 19.99 | 5.5799999 | 27.913956 | 72.086044 | 1 | 15.381246 | 1 | 1.5539941 | 4 | 106.61742 | 16.983889 | 86.360001 | 1 | 6 | 5 |  | 0 | 0 | 0 | 0 | 0 | 0 | 119.5164061 | 1.775185394 | 92.4358172 | -1.078906529 | 2 | 2 | 2 | 1 | 0 | 1 | 0 | 3 | 25 | 0 | 7 | 0 |  |
| 8430 | 1 | 16.665001 | 4.9299998 | 29.582954 | 70.417046 | 1 | 15.682409 | 1 | 1.5539941 | 4 | 106.61742 | 18.01222 | 80.18 | 1 | 12 | 5 |  | 1 | 0 | 0 | 0 | 1 | 0 | 133.8833115 | 3.070908347 | 90.62347382 | -1.310768461 | 2 | 2 | 2 | 1 | 0 | 1 |  | 3 | 24 | 0 | 7 | 0 |  |
| 8434 | 0 | 22.97 | 6.153 | 26.787113 | 73.212883 | 0 | 9.0622864 | 1 | 0.36403459 | 3 | 103.74841 | 18.766111 | 81.849 | 1 | 0 | 5 | 1 | 0 | 0 | 0 | 0 | 0 |  | 117.5906859 | 1.557717781 | 94.13591453 | -0.884337154 | 2 | 2 |  | 0 | 0 | 1 | 0 | 2 |  | | | | |
| 8434 | 1 | 24.690001 | 7.4699998 | 30.255161 | 69.744835 | 0 | 9.5441475 | 1 | 0.36403459 | 3 | 103.74841 | 21.255001 | 78.389999 | 1 | 1 | 5 | 1 | 1 | 0 | 0 | 0 | 0 |  | 110.7941906 | 0.966639691 | 93.76932524 | -0.942299427 | 2 | 2 | 2 | 0 | 0 | 1 |  | 2 | 19 | 1 | 6 | 1 |  |
| 8434 | 1 | 16.576 | 5.2600002 | 31.732626 | 68.267372 | 0 | 9.6208076 | 1 | 0.36403459 | 3 | 103.74841 | 20.062222 | 83.75 | 1 | 3 | 5 | 1 | 1 | 0 | 0 | 0 | 0 |  | | NA |  | NA | 2 | 2 | 2 | 0 | 0 | 1 |  | 2 | 25 | 0 | 6.4000001 | 1 |  |
| 8434 | 0 | 17.312 | 5.6100001 | 32.40527 | 67.594734 | 0 | 9.6974678 | 1 | 0.36403459 | 3 | 103.74841 | 19.213333 | 84.629997 | 1 | 5 | 5 | 1 | 0 | 0 | 0 | 0 | 0 |  | | NA |  | NA | 2 | 2 | 1 | 0 | 0 | 1 |  | 2 | 9 | 1 | 3.7 | 1 |  |
| 8434 | 0 | 19.820999 | 5.1199999 | 25.83119 | 74.168808 | 0 | 9.7357969 | 1 | 0.36403459 | 3 | 103.74841 | 18.215557 | 85.330002 | 1 | 6 | 5 | 1 | 0 | 0 | 0 | 0 | 0 |  | 131.0472023 | 2.776950255 | 97.18813681 | -0.441674941 | 2 | 2 | 1 | 0 | 0 | 1 | 0 | 2 | 15 | 1 | 5.5 | 1 |  |
| 8434 | 0 | 22.584 | 5.1799998 | 22.936592 | 77.063408 | 0 | 10.075291 | 1 | 0.36403459 | 3 | 103.74841 | 17.202223 | 84.739998 | 1 | 12 | 5 | 1 | 0 | 0 | 0 | 0 | 0 |  | 109.2272554 | 0.833634729 | 95.33645545 | -0.721436479 | 2 | 2 | 2 | 0 | 0 | 1 |  | 2 | 17 | 1 | 7 | 0 |  |
| 8436 |  | 16.322 | 6.203 | 38.003922 | 61.996078 | 0 | 11.846681 | 1 | -0.14631924 | 3 | 87.027428 | 18.817223 | 77.449 | 1 | 0 | 5 | 1 |  | | | | | | 91.88307422 | -0.746192466 | 101.0096016 | 0.164824265 | 2 | 2 |  | 0 | 0 | 0 | 0 |  | | | | | |
| 8436 | 1 | 23.82 | 8.6099997 | 36.146095 | 63.853905 | 1 | 12.421629 | 1 | -0.14631924 | 3 | 87.027428 | 22.268333 | 73.470001 | 1 | 1 | 5 | 1 | 1 | 1 | 1 | 1 | 1 | 0 | 114.7441944 | 1.348461827 | 100.0757098 | 0.012167487 | 2 | 2 | 2 | 0 | 0 | 0 |  | | 25 | 0 | 7 | 0 |  |
| 8436 | 1 | 16.405001 | 5.7600002 | 35.111244 | 64.888756 | 1 | 12.498289 | 1 | -0.14631924 | 3 | 87.027428 | 20.385 | 81.870003 | 1 | 3 | 5 | 1 | 1 | 1 | 0 | 0 | 0 |  | | NA |  | NA | 2 | 2 | 2 | 0 | 0 | 0 |  | | 25 | 0 | 7 | 0 |  |
| 8436 | 0 | 16.941 | 5.8299999 | 34.413551 | 65.586449 | 1 | 12.574948 | 1 | -0.14631924 | 3 | 87.027428 | 19.526114 | 82.199997 | 1 | 5 | 5 | 1 | 0 | 0 | 0 | 0 | 0 |  | | NA |  | NA | 2 | 2 | 2 | 0 | 0 | 0 |  | | 27 | 0 | 7 | 0 |  |
| 8436 | 0 | 24.358999 | 5.29 | 21.71682 | 78.28318 | 1 | 12.618754 | 1 | -0.14631924 | 3 | 87.027428 | 17.584999 | 85.919998 | 1 | 6 | 5 | 1 | 0 | 0 | 0 | 0 | 0 | 0 | 119.2311145 | 1.756908831 | 93.60508045 | -0.967303749 | 2 | 2 | 2 | 0 | 0 | 0 | 0 |  | 24 | 0 | 6 | 1 |  |
| 8436 | 0 | 24.159 | 5.5700002 | 23.055592 | 76.944412 | 1 | 12.936345 | 1 | -0.14631924 | 3 | 87.027428 | 18.562223 | 79.300003 | 1 | 12 | 5 | 1 | 0 | 0 | 0 | 0 | 0 | 0 | 121.252902 | 1.940313693 | 100.1006017 | 0.016033675 | 2 | 2 | 2 | 0 | 0 | 0 |  | | 24 | 0 | 7 | 0 |  |
| 8451 | 1 | 21.476 | 5.912 | 27.528404 | 72.471596 | 0 | 10.097194 | 0 | 1.9938918 | 4 | 113.70047 | 27.435556 | 67.753 | 1 | 0 | 5 | 0 | 1 | 0 | 0 | 0 | 0 |  | 118.0756008 | 1.583299661 | 102.9726891 | 0.563855392 | 2 | 2 |  | 0 | 0 | 0 | 0 | 3 |  | | | | |
| 8451 | 1 | 23.4 | 6.73 | 28.760683 | 71.239319 | 0 | 10.661191 | 0 | 1.9938918 | 4 | 113.70047 | 20.997221 | 80.150002 | 1 | 1 | 5 | 0 | 1 | 1 | 1 | 1 | 0 |  | 112.0389291 | 1.060922778 | 94.70961078 | -0.874544495 | 2 | 2 | 2 | 0 | 0 | 0 |  | 3 | 27 | 0 | 6.8000002 | 1 |  |
| 8451 | 1 | 23.476999 | 6.5100002 | 27.729269 | 72.270729 | 0 | 10.740588 | 0 | 1.9938918 | 4 | 113.70047 | 20.484446 | 78.059998 | 1 | 3 | 5 | 0 | 1 | 1 | 1 | 1 | 0 |  | | NA |  | NA | 2 | 2 | 2 | 0 | 0 | 0 |  | 3 | 27 | 0 | 7 | 0 |  |
| 8451 | 0 | 16.882 | 5.0700002 | 30.031988 | 69.96801 | 0 | 10.81451 | 0 | 1.9938918 | 4 | 113.70047 | 19.810555 | 79.589996 | 1 | 5 | 5 | 0 | 0 | 0 | 0 | 0 | 0 |  | | NA |  | NA | 2 | 2 | 2 | 0 | 0 | 0 |  | 3 | 24 | 0 | 7 | 0 |  |
| 8451 | 0 | 25.173 | 5.5700002 | 22.126883 | 77.873116 | 0 | 10.85284 | 0 | 1.9938918 | 4 | 113.70047 | 17.4 | 83.470001 | 1 | 6 | 5 | 0 | 0 | 0 | 0 | 0 | 0 |  | 111.0481598 | 0.974227034 | 99.79394991 | -0.036901814 | 2 | 2 | 2 | 0 | 0 | 0 | 0 | 3 | 27 | 0 | 7 | 0 |  |
| 8451 | 0 | 21.537001 | 4.6300001 | 21.497887 | 78.502113 | 0 | 11.156742 | 0 | 1.9938918 | 4 | 113.70047 | 18.609446 | 77.339996 | 1 | 12 | 5 | 0 | 0 | 0 | 0 | 0 | 0 |  | 111.6713702 | 1.027886269 | 95.86703178 | -0.693934191 | 2 | 2 | 2 | 0 | 0 | 0 |  | 3 | 27 | 0 | 7 | 0 |  |
| 8453 | 0 | 19.959 | 5.373 | 26.920187 | 73.079811 | 0 | 10.392881 | 0 | 0.11822473 | 3 | 79.673203 | 21.728333 | 76.787 | 1 | 0 | 5 | 1 | 0 | 0 | 0 | 0 | 0 |  | 89.86296374 | -0.910667115 | 94.7431318 | -0.87056962 | 2 | 2 |  | 1 | 0 | 1 | 1 | 2 |  | | | | |
| 8453 | 0 | 20.407 | 5.6599998 | 27.73558 | 72.26442 | 0 | 10.973306 | 0 | 0.11822473 | 3 | 79.673203 | 20.506668 | 83.080002 | 1 | 1 | 5 | 1 | 0 | 0 | 0 | 0 | 0 |  | 100.0579588 | 0.005157692 | 91.68699121 | -1.307413312 | 2 | 2 | 2 | 1 | 0 | 1 |  | 2 | 23 | 0 | 6.0999999 | 1 |  |
| 8453 | 0 | 16.739 | 5.3699999 | 32.080769 | 67.919228 | 0 | 11.030801 | 0 | 0.11822473 | 3 | 79.673203 | 20.087221 | 81.839996 | 1 | 3 | 5 | 1 | 0 | 0 | 0 | 0 | 0 |  | | NA |  | NA | 2 | 2 | 2 | 1 | 0 | 1 |  | 2 | 23 | 0 | 7 | 0 |  |
| 8453 | 0 | 16.841 | 5.27 | 31.292679 | 68.707321 | 0 | 11.104723 | 0 | 0.11822473 | 3 | 79.673203 | 19.376665 | 82.580002 | 1 | 5 | 5 | 1 | 0 | 0 | 0 | 0 | 0 |  | | NA |  | NA | 2 | 2 | 2 | 1 | 0 | 1 |  | 2 | 27 | 0 | 7 | 0 |  |
| 8453 | 1 | 20.490999 | 5.1399999 | 25.084185 | 74.915817 | 0 | 11.14579 | 0 | 0.11822473 | 3 | 79.673203 | 16.943335 | 86.489998 | 1 | 6 | 5 | 1 | 1 | 0 | 0 | 0 | 0 |  | 107.1782797 | 0.634562546 | 92.32421639 | -1.21851876 | 2 | 2 | 2 | 1 | 0 | 1 | 1 | 2 | 18 | 1 | 5.9000001 | 1 |  |
| 8453 | 0 | 17.778 | 4.1799998 | 23.512205 | 76.487793 | 0 | 11.444217 | 0 | 0.11822473 | 3 | 79.673203 | 18.441668 | 81.32 | 1 | 12 | 5 | 1 | 0 | 0 | 0 | 0 | 0 |  | 92.46340728 | -0.674198221 | 93.49100079 | -1.050828107 | 2 | 2 | 2 | 1 | 0 | 1 |  | 2 | 18 | 1 | 5.5999999 | 1 |  |
| 8459 | 1 | 21.142 | 5.623 | 26.596348 | 73.403648 | 0 | 9.8836412 | 0 | 2.0574906 | 4 | 133.39476 | 18.481667 | 81.541 | 1 | 0 | 4 | 0 | 1 | 0 | 0 | 0 | 0 |  | 142.6134227 | 3.661846983 | 99.89324784 | -0.019285004 | 2 | 2 |  | 1 | 0 | 0 | 0 | 3 |  | | | | |
| 8459 | 0 | 16.601999 | 5.2600002 | 31.682934 | 68.31707 | 0 | 10.494182 | 0 | 2.0574906 | 4 | 133.39476 | 20.055555 | 83.769997 | 1 | 3 | 4 | 0 | 0 | 0 | 0 | 0 | 0 |  | | NA |  | NA | 2 | 2 | 2 | 1 | 0 | 0 |  | 3 | 22 | 0 | 7 | 0 |  |
| 8459 | 0 | 17.34 | 5.6199999 | 32.41061 | 67.589386 | 0 | 10.570842 | 0 | 2.0574906 | 4 | 133.39476 | 19.211113 | 84.629997 | 1 | 5 | 4 | 0 | 0 | 0 | 0 | 0 | 0 |  | | NA |  | NA | 2 | 2 | 2 | 1 | 0 | 0 |  | 3 | 24 | 0 | 6.5999999 | 1 |  |
| 8459 | 0 | 19.819 | 5.1199999 | 25.833796 | 74.166206 | 0 | 10.61191 | 0 | 2.0574906 | 4 | 133.39476 | 18.083336 | 85.629997 | 1 | 6 | 4 | 0 | 0 | 0 | 0 | 0 | 0 |  | 126.2142462 | 2.284698981 | 95.94207976 | -0.684424373 | 2 | 2 | 2 | 1 | 0 | 0 | 0 | 3 | 20 | 0 | 6 | 1 |  |
| 8459 | 0 | 22.591 | 5.1900001 | 22.973751 | 77.026253 | 0 | 10.902122 | 0 | 2.0574906 | 4 | 133.39476 | 17.189445 | 84.779999 | 1 | 12 | 4 | 0 | 0 | 0 | 0 | 0 | 0 |  | 141.8470406 | 3.605577628 | 97.91800895 | -0.361774317 | 2 | 2 | 2 | 1 | 0 | 0 |  | 3 | 23 | 0 | 7 | 0 |  |
| 8461 | 1 | 21.587 | 5.683 | 26.326029 | 73.673973 | 0 | 10.198494 | 0 | 2.4412034 | 3 | 125.35371 | 18.65889 | 81.414 | 1 | 0 | 4 | 0 | 1 | 1 | 1 | 1 | 0 |  | 122.1575013 | 1.935572951 | 109.209031 | 1.92975349 | 2 | 2 |  | 0 | 0 | 0 | 0 | 4 |  | | | | |
| 8461 | 0 | 24.664 | 7.4699998 | 30.287058 | 69.712944 | 0 | 10.718686 | 0 | 2.4412034 | 3 | 125.35371 | 21.262224 | 78.360001 | 1 | 1 | 4 | 0 | 0 | 0 | 0 | 0 | 0 |  | 124.7458872 | 2.159119832 | 75.59899155 | -2.96240293 | 2 | 2 | 2 | 0 | 0 | 0 |  | 4 | 23 | 0 | 6.3000002 | 1 |  |
| 8461 | 0 | 16.627001 | 5.27 | 31.695433 | 68.304565 | 0 | 10.795345 | 0 | 2.4412034 | 3 | 125.35371 | 20.075556 | 83.669998 | 1 | 3 | 4 | 0 | 0 | 0 | 0 | 0 | 0 |  | | NA |  | NA | 2 | 2 | 2 | 0 | 0 | 0 |  | 4 | 23 | 0 | 7 | 0 |  |
| 8461 | 1 | 17.296 | 5.6100001 | 32.435246 | 67.564751 | 0 | 10.872005 | 0 | 2.4412034 | 3 | 125.35371 | 19.222776 | 84.550003 | 1 | 5 | 4 | 0 | 1 | 0 | 0 | 0 | 0 |  | | NA |  | NA | 2 | 2 | 2 | 0 | 0 | 0 |  | 4 | 26 | 0 | 6.8000002 | 1 |  |
| 8461 | 0 | 22.486 | 5.1599998 | 22.947611 | 77.052391 | 0 | 11.206023 | 0 | 2.4412034 | 3 | 125.35371 | 17.217222 | 84.610001 | 1 | 12 | 4 | 0 | 0 | 0 | 0 | 0 | 0 |  | 120.1244538 | 1.760197334 | 108.9913053 | 1.852879364 | 2 | 2 | 2 | 0 | 0 | 0 |  | 4 | 23 | 0 | 7 | 0 |  |
| 8469 | 1 | 21.751 | 5.733 | 26.357409 | 73.642593 | 1 | 14.149213 | 1 | 1.9938918 | 4 | 114.17713 | 18.958334 | 80.812 | 1 | 0 | 4 | 1 | 0 | 0 | 0 | 0 | 1 | 0 | 116.3071565 | 1.487727245 | 105.0236852 | 0.814411894 | 2 | 2 |  | 0 | 0 | 0 | 0 |  | | | | | |
| 8469 | 1 | 24.570999 | 7.5100002 | 30.564489 | 69.435509 | 1 | 14.661191 | 1 | 1.9938918 | 4 | 114.17713 | 21.37611 | 77.900002 | 1 | 1 | 4 | 1 | 1 | 1 | 1 | 0 | 1 | 0 | 105.4309186 | 0.496291228 | 104.9695261 | 0.796486449 | 2 | 2 | 2 | 0 | 0 | 0 |  | | 23 | 0 | 7 | 0 |  |
| 8469 | 1 | 16.646 | 5.3000002 | 31.839481 | 68.160515 | 1 | 14.737851 | 1 | 1.9938918 | 4 | 114.17713 | 20.136112 | 83.389999 | 1 | 3 | 4 | 1 | 1 | 1 | 1 |  | 1 | 0 |  | NA |  | NA | 2 | 2 | 2 | 0 | 0 | 0 |  | | 23 | 0 | 7 | 0 |  |
| 8469 | 0 | 18.808001 | 5.29 | 28.126328 | 71.873672 | 1 | 14.863791 | 1 | 1.9938918 | 4 | 114.17713 | 18.43778 | 85.309998 | 1 | 6 | 4 | 1 | 0 | 0 | 0 | 0 | 0 | 0 | 120.2710021 | 1.845165675 | 106.6259902 | 1.071586106 | 2 | 2 | 2 | 0 | 0 | 0 | 0 |  | 24 | 0 | 7 | 0 |  |
| 8469 | 0 | 21.823 | 4.8600001 | 22.270082 | 77.729919 | 1 | 15.148528 | 1 | 1.9938918 | 4 | 114.17713 | 17.626112 | 83.769997 | 1 | 12 | 4 | 1 | 0 | 0 | 0 | 0 | 0 | 0 | 110.9551765 | 0.99886977 | 108.6273657 | 1.409231236 | 2 | 2 | 2 | 0 | 0 | 0 |  | | 24 | 0 | 7 | 0 |  |
| 8488 | 1 | 22.926 | 5.629 | 24.55291 | 75.44709 | 0 | 10.669405 | 0 | -0.59363079 | 4 | 117.95576 | 25.117222 | 72.162 | 1 | 0 | 5 | 1 | 1 | 0 | 0 | 0 | 0 |  | 131.4271886 | 2.728531098 | 94.93143691 | -0.840837154 | 2 | 2 |  | 0 | 0 | 0 | 0 |  | | | | | |
| 8488 | 0 | 24.195999 | 6.5799999 | 27.194578 | 72.80542 | 0 | 11.162218 | 0 | -0.59363079 | 4 | 117.95576 | 21.740557 | 75.480003 | 1 | 1 | 5 | 1 | 0 | 0 | 0 | 0 | 0 |  | 106.08263 | 0.538176201 | 86.53837735 | -1.949031855 | 2 | 2 | 2 | 0 | 0 | 0 |  | | 22 | 0 | 6.9000001 | 1 |  |
| 8488 | 0 | 18.34 | 4.98 | 27.153763 | 72.846237 | 0 | 11.238877 | 0 | -0.59363079 | 4 | 117.95576 | 20.520554 | 80.18 | 1 | 3 | 5 | 1 | 0 | 0 | 0 | 0 | 0 |  | | NA |  | NA | 2 | 2 | 2 | 0 | 0 | 0 |  | | 25 | 0 | 7 | 0 |  |
| 8488 | 0 | 16.386 | 5.1599998 | 31.490295 | 68.509705 | 0 | 11.315537 | 0 | -0.59363079 | 4 | 117.95576 | 19.937778 | 79.400002 | 1 | 5 | 5 | 1 | 0 | 0 | 0 | 0 | 0 |  | | NA |  | NA | 2 | 2 | 2 | 0 | 0 | 0 |  | | 27 | 0 | 7 | 0 |  |
| 8488 | 1 | 17.709999 | 5.02 | 28.34557 | 71.654434 | 0 | 11.353868 | 0 | -0.59363079 | 4 | 117.95576 | 17.595556 | 85.010002 | 1 | 6 | 5 | 1 | 1 | 1 | 0 | 0 | 0 |  | 140.3614449 | 3.476321465 | 98.57211108 | -0.249793994 | 2 | 2 | 2 | 0 | 0 | 0 | 0 |  | 20 | 0 | 4.9000001 | 1 |  |
| 8488 | 1 | 18.319 | 4.4200001 | 24.127954 | 75.872047 | 0 | 11.657768 | 0 | -0.59363079 | 4 | 117.95576 | 18.097221 | 80.160004 | 1 | 12 | 5 | 1 | 1 | 1 | 0 | 0 | 0 |  | 140.6970795 | 3.499453167 | 96.09315914 | -0.655915709 | 2 | 2 | 2 | 0 | 0 | 0 |  | | 27 | 0 | 7 | 0 |  |
| 8501 | 1 | 17.999 | 6.096 | 33.868549 | 66.131454 | 0 | 11.627652 | 1 | 2.2397017 | 2 | 88.273712 | 23.930555 | 76.362 | 1 | 0 | 5 | 1 | 1 | 1 | 1 | 1 | 1 |  | 100.6784443 | 0.062188977 | 93.85886699 | -0.940358616 | 2 | 2 |  | 0 | 0 | 0 | 0 | 2 |  | | | | |
| 8501 | 0 | 26.784 | 8.3400002 | 31.137993 | 68.862007 | 1 | 12.312115 | 1 | 2.2397017 | 2 | 88.273712 | 21.57889 | 78.669998 | 1 | 1 | 5 | 1 | 0 | 0 | 0 | 0 | 0 | 0 | 84.85912377 | -1.395548628 | 93.01676636 | -1.054825584 | 2 | 2 | 2 | 0 | 0 | 0 |  | 2 | 14 | 1 | 7 | 0 |  |
| 8501 | 1 | 15.371 | 6.3200002 | 41.11639 | 58.88361 | 1 | 12.377824 | 1 | 2.2397017 | 2 | 88.273712 | 20.611111 | 82.25 | 1 | 3 | 5 | 1 | 0 | 0 | 0 | 0 | 1 | 0 |  | NA |  | NA | 2 | 2 | 1 | 0 | 0 | 0 |  | 2 | 18 | 1 | 6.6999998 | 1 |  |
| 8501 | 0 | 18.806999 | 6.1999998 | 32.96645 | 67.033554 | 1 | 12.454483 | 1 | 2.2397017 | 2 | 88.273712 | 20.000555 | 81.639999 | 1 | 5 | 5 | 1 | 0 | 0 | 0 | 0 | 0 | 0 |  | NA |  | NA | 2 | 2 | 1 | 0 | 0 | 0 |  | 2 | 21 | 0 | 6.8000002 | 1 |  |
| 8501 | 0 | 20.129 | 5.8099999 | 28.863829 | 71.136169 | 1 | 12.498289 | 1 | 2.2397017 | 2 | 88.273712 | 17.664446 | 86.910004 | 1 | 6 | 5 | 1 | 0 | 0 | 0 | 0 | 0 | 0 | 102.4581169 | 0.225488293 | 96.07362475 | -0.60770933 | 2 | 2 | 2 | 0 | 0 | 0 | 0 | 2 | 21 | 0 | 7 | 0 |  |
| 8501 | 0 | 18.780001 | 4.5799999 | 24.387644 | 75.612358 | 1 | 12.813142 | 1 | 2.2397017 | 2 | 88.273712 | 17.788332 | 84.029999 | 1 | 12 | 5 | 1 | 0 | 0 | 0 | 0 | 0 | 0 | 96.54863997 | -0.317043487 | 95.2636295 | -0.725020511 | 2 | 2 | 2 | 0 | 0 | 0 |  | 2 | 24 | 0 | 6.8000002 | 1 |  |
| 8502 | 1 | 18.684 | 6.004 | 32.134445 | 67.865555 | 1 | 12.785763 | 1 | -1.8673207 | 4 | 72.777359 | 24.722778 | 75.165 | 1 | 0 | 5 |  | 1 | 1 | 1 | 1 | 1 | 1 | 82.06368993 | -1.654375362 | 94.49803123 | -0.836869869 | 2 | 2 |  | 0 | 0 | 1 | 1 | 2 |  | | | | |
| 8502 | 1 | 29.004999 | 8.8000002 | 30.339598 | 69.6604 | 1 | 13.399042 | 1 | -1.8673207 | 4 | 72.777359 | 21.933891 | 77.120003 | 1 | 1 | 5 |  | 1 | 1 | 0 | 0 | 1 | 0 | 94.54233249 | -0.501303507 | 92.26878304 | -1.141434468 | 2 | 2 | 2 | 0 | 0 | 1 |  | 2 | 24 | 0 | 7 | 0 |  |
| 8502 | 1 | 16.332001 | 6.3400002 | 38.819496 | 61.180504 | 1 | 13.475701 | 1 | -1.8673207 | 4 | 72.777359 | 20.655556 | 82.349998 | 1 | 3 | 5 |  | 1 | 1 | 0 | 0 | 1 | 0 |  | NA |  | NA | 2 | 2 | 2 | 0 | 0 | 1 |  | 2 | 24 | 0 | 7 | 0 |  |
| 8502 | 0 | 20.309999 | 5.6500001 | 27.81881 | 72.18119 | 1 | 13.571527 | 1 | -1.8673207 | 4 | 72.777359 | 19.628334 | 82.139999 | 1 | 5 | 5 |  | 0 | 0 | 0 | 0 | 0 | 0 |  | NA |  | NA | 2 | 2 | 2 | 0 | 0 | 1 |  | 2 | 23 | 0 | 7 | 0 |  |
| 8502 | 1 | 20.309999 | 5.8299999 | 28.705072 | 71.29493 | 1 | 13.601643 | 1 | -1.8673207 | 4 | 72.777359 | 17.290001 | 86.970001 | 1 | 6 | 5 |  | 1 | 1 | 0 | 0 | 1 | 0 | 94.2471961 | -0.528270658 | 90.60997157 | -1.361520935 | 2 | 2 | 2 | 0 | 0 | 1 | 0 | 2 | 16 | 1 | 5.3000002 | 1 |  |
| 8502 | 0 | 18.851 | 4.5799999 | 24.295794 | 75.704208 | 1 | 13.913757 | 1 | -1.8673207 | 4 | 72.777359 | 17.798334 | 83.870003 | 1 | 12 | 5 |  | 0 | 0 | 0 | 0 | 0 | 0 | 94.78356076 | -0.478677533 | 94.60912899 | -0.804727845 | 2 | 2 | 2 | 0 | 0 | 1 |  | 2 | 24 | 0 | 7 | 0 |  |
| 8504 | 1 | 17.201 | 5.157 | 29.980816 | 70.019188 | 1 | 16.262835 | 0 | 1.9938918 | 2 | 112.39799 | 19.790556 | 74.8 | 1 | 0 | 4 | 1 | 1 | 1 | 1 | 1 | 1 | 1 | 127.9764164 | 2.358689248 | 94.17552143 | -0.911943425 | 2 | 2 |  | 0 | 0 | 0 | 0 | 3 |  | | | | |
| 8504 | 1 | 23.218 | 7.3299999 | 31.570333 | 68.429665 | 1 | 16.873375 | 0 | 1.9938918 | 2 | 112.39799 | 22.144445 | 74.709999 | 1 | 1 | 4 | 1 | 1 | 1 | 1 | 1 | 1 | 1 | 135.3552301 | 2.9562422 | 92.38762447 | -1.156522528 | 2 | 2 | 2 | 0 | 0 | 0 |  | 3 | 15 | 1 | 7 | 0 |  |
| 8504 | 1 | 15.048 | 4.96 | 32.961189 | 67.038811 | 1 | 17.023956 | 0 | 1.9938918 | 2 | 112.39799 | 19.684444 | 82.370003 | 1 | 5 | 4 | 1 | 1 | 1 | 1 | 0 | 1 | 0 |  | NA |  | NA | 2 | 2 | 2 | 0 | 0 | 0 |  | 3 | 24 | 0 | 7 | 0 |  |
| 8504 | 1 | 21.268 | 4.4200001 | 20.782396 | 79.217606 | 1 | 17.075975 | 0 | 1.9938918 | 2 | 112.39799 | 17.456667 | 86.5 | 1 | 6 | 4 | 1 | 1 | 1 | 1 | 1 | 1 | 0 | 131.7475221 | 2.658969527 | 94.54021198 | -0.852061558 | 2 | 2 | 2 | 0 | 0 | 0 | 0 | 3 | 22 | 0 | 6.6999998 | 1 |  |
| 8504 | 1 | 19.958 | 3.8399999 | 19.240404 | 80.759598 | 1 | 17.385353 | 0 | 1.9938918 | 2 | 112.39799 | 17.774445 | 81.809998 | 1 | 12 | 4 | 1 | 1 | 0 | 0 | 0 | 1 | 0 | 93.95179761 | -0.521673404 | 82.43525596 | -2.323767325 | 2 | 2 | 1 | 0 | 0 | 0 |  | 3 | 16 | 1 | 7 | 0 |  |
| 8506 |  | 20.222 | 5.45 | 26.950846 | 73.049156 | 1 | 16.052019 | 0 | -0.32167307 | 2 | 96.909737 | 19.770555 | 78.425 | 1 | 0 | 4 | 1 |  | | | | | | 108.4243323 | 0.7220164 | 95.71511616 | -0.686579249 | 2 | 2 |  | 1 | 0 | 0 | 0 |  | | | | | |
| 8506 | 0 | 23.987 | 7.5 | 31.266935 | 68.733063 | 1 | 16.585899 | 0 | -0.32167307 | 2 | 96.909737 | 21.59 | 76.989998 | 1 | 1 | 4 | 1 | 0 | 0 | 0 | 0 | 0 | 0 | 109.8764214 | 0.843210672 | 93.98303912 | -0.936671264 | 2 | 2 | 2 | 1 | 0 | 0 |  | | 25 | 0 | 7 | 0 |  |
| 8506 | 0 | 16.540001 | 5.27 | 31.86215 | 68.137848 | 1 | 16.66256 | 0 | -0.32167307 | 2 | 96.909737 | 20.29611 | 82.550003 | 1 | 3 | 4 | 1 | 0 | 0 | 0 | 0 | 0 | 0 |  | NA |  | NA | 2 | 2 | 2 | 1 | 0 | 0 |  | | 25 | 0 | 6.6999998 | 1 |  |
| 8506 | 0 | 16.825001 | 5.54 | 32.927189 | 67.072807 | 1 | 16.73922 | 0 | -0.32167307 | 2 | 96.909737 | 19.392776 | 83.419998 | 1 | 5 | 4 | 1 | 0 | 0 | 0 | 0 | 0 | 0 |  | NA |  | NA | 2 | 2 | 2 | 1 | 0 | 0 |  | | 24 | 0 | 7 | 0 |  |
| 8506 | 1 | 21.357 | 4.8099999 | 22.521889 | 77.478111 | 1 | 17.106092 | 0 | -0.32167307 | 2 | 96.909737 | 17.479445 | 82.720001 | 1 | 12 | 4 | 1 | 1 | 1 | 1 | 1 | 0 | 1 | 108.7782014 | 0.748326797 | 91.60657062 | -1.259150277 | 2 | 2 | 2 | 1 | 0 | 0 |  | | 25 | 0 | 7 | 0 |  |
| 8510 | 1 | 19.171 | 6.691 | 34.901676 | 65.098328 | 1 | 14.231348 | 1 | 2.3033004 | 2 | 93.634712 | 17.096666 | 83.324 | 1 | 0 | 4 | 1 | 1 | 1 | 1 | 1 | 1 | 1 | 90.42929919 | -0.878783639 | 110.8875002 | 1.847322441 | 2 | 2 |  | 1 | 0 | 0 | 0 |  | | | | | |
| 8510 | 1 | 23.785999 | 7.5100002 | 31.573196 | 68.426804 | 1 | 15.008898 | 1 | 2.3033004 | 2 | 93.634712 | 21.839998 | 76.019997 | 1 | 1 | 4 | 1 | 0 | 0 | 1 | 1 | 1 | 0 | 93.59062934 | -0.58715624 | 108.7756578 | 1.439228631 | 2 | 2 | 2 | 1 | 0 | 0 |  | | 15 | 1 | 7 | 0 |  |
| 8510 | 1 | 16.291 | 5.23 | 32.103615 | 67.896385 | 1 | 15.085558 | 1 | 2.3033004 | 2 | 93.634712 | 20.39889 | 82.18 | 1 | 3 | 4 | 1 | 1 | 1 | 1 | 1 | 1 | 1 |  | NA |  | NA | 2 | 2 | 2 | 1 | 0 | 0 |  | | 24 | 0 | 7 | 0 |  |
| 8510 | 0 | 19.080999 | 4.9200001 | 25.784815 | 74.215187 | 1 | 15.200547 | 1 | 2.3033004 | 2 | 93.634712 | 18.358889 | 84.610001 | 1 | 6 | 4 | 1 | 0 | 0 | 0 | 0 | 0 | 0 | 87.94230763 | -1.105908687 | 111.4924254 | 1.917685977 | 2 | 2 | 2 | 1 | 0 | 0 | 0 |  | 20 | 0 | 6.4000001 | 1 |  |
| 8510 | 1 | 21.018 | 4.6100001 | 21.93358 | 78.066422 | 1 | 15.526352 | 1 | 2.3033004 | 2 | 93.634712 | 17.584999 | 82.349998 | 1 | 12 | 4 | 1 | 1 | 1 | 1 | 1 | 0 | 1 | 86.09413594 | -1.275453167 | 111.8762677 | 1.97435508 | 2 | 2 | 2 | 1 | 0 | 0 |  | | 17 | 1 | 7 | 0 |  |
| 8512 | 0 | 19.131 | 6.646 | 34.739429 | 65.260574 | 0 | 10.198494 | 1 | 1.6036592 | 2 | 124.92842 | 17.037777 | 83.49 | 1 | 0 | 4 | 1 | 0 | 0 | 0 | 0 | 0 |  | 123.866334 | 2.152358253 | 109.9405928 | 1.775991991 | 2 | 2 |  | 0 | 0 | 0 | 0 | 4 |  | | | | |
| 8512 | 1 | 23.724001 | 7.4499998 | 31.402796 | 68.597206 | 0 | 10.976044 | 1 | 1.6036592 | 2 | 124.92842 | 21.618889 | 76.760002 | 1 | 1 | 4 | 1 | 1 | 0 | 1 | 1 | 1 |  | 109.1941066 | 0.838307507 | 109.6209607 | 1.71085785 | 2 | 2 | 2 | 0 | 0 | 0 |  | 4 | 27 | 0 | 7 | 0 |  |
| 8512 | 1 | 16.357 | 5.2399998 | 32.035213 | 67.96479 | 0 | 11.049966 | 1 | 1.6036592 | 2 | 124.92842 | 20.327221 | 82.470001 | 1 | 3 | 4 | 1 | 1 | 1 | 1 | 1 | 1 | 0 |  | NA |  | NA | 2 | 2 | 2 | 0 | 0 | 0 |  | 4 | 27 | 0 | 7 | 0 |  |
| 8512 | 0 | 19.114 | 4.9699998 | 26.001883 | 73.998116 | 0 | 11.164955 | 1 | 1.6036592 | 2 | 124.92842 | 18.33889 | 84.650002 | 1 | 6 | 4 | 1 | 0 | 0 | 0 | 0 | 0 | 0 | 107.8408576 | 0.715984334 | 112.539249 | 2.286203746 | 2 | 2 | 2 | 0 | 0 | 0 | 0 | 4 | 24 | 0 | 6.6999998 | 1 |  |
| 8512 | 0 | 21.209 | 4.7199998 | 22.254702 | 77.7453 | 0 | 11.49076 | 1 | 1.6036592 | 2 | 124.92842 | 17.531666 | 82.57 | 1 | 12 | 4 | 1 | 0 | 0 | 0 | 0 | 0 | 0 | 112.9892288 | 1.186509481 | 104.8208231 | 0.817571412 | 2 | 2 | 2 | 0 | 0 | 0 |  | 4 | 25 | 0 | 7 | 0 |  |
| 8514 | 1 | 19.249 | 6.721 | 34.9161 | 65.0839 | 0 | 9.8781652 | 1 | 1.0032448 | 4 | 105.08755 | 16.930555 | 83.721 | 1 | 0 | 4 | 1 | 1 | 1 | 1 | 0 | 1 |  | 107.1091859 | 0.640786417 | 106.9691402 | 1.208848681 | 2 | 2 |  | 0 | 0 | 1 | 0 | 3 |  | | | | |
| 8514 | 1 | 23.819 | 7.5 | 31.487469 | 68.512535 | 0 | 10.658453 | 1 | 1.0032448 | 4 | 105.08755 | 21.673889 | 76.639999 | 1 | 1 | 4 | 1 | 1 | 1 | 1 | 1 | 1 |  | 109.9392771 | 0.903805044 | 105.1186884 | 0.873676116 | 2 | 2 | 2 | 0 | 0 | 1 |  | 3 | 22 | 0 | 7 | 0 |  |
| 8514 | 1 | 16.437 | 5.25 | 31.940136 | 68.059868 | 0 | 10.735113 | 1 | 1.0032448 | 4 | 105.08755 | 20.341665 | 82.339996 | 1 | 3 | 4 | 1 | 1 | 1 | 1 | 1 | 1 |  | | NA |  | NA | 2 | 2 | 2 | 0 | 0 | 1 |  | 3 | 23 | 0 | 6.8000002 | 1 |  |
| 8514 | 1 | 16.664 | 5.5 | 33.00528 | 66.99472 | 0 | 10.811772 | 1 | 1.0032448 | 4 | 105.08755 | 19.421112 | 83.25 | 1 | 5 | 4 | 1 | 1 | 1 | 1 | 1 | 0 |  | | NA |  | NA | 2 | 2 | 2 | 0 | 0 | 1 |  | 3 | 23 | 0 | 7 | 0 |  |
| 8514 | 1 | 19.447001 | 4.98 | 25.608061 | 74.391937 | 0 | 10.855578 | 1 | 1.0032448 | 4 | 105.08755 | 18.138889 | 84.919998 | 1 | 6 | 4 | 1 | 1 | 1 | 1 | 1 | 1 |  | 108.1609439 | 0.743628335 | 106.5902874 | 1.140127653 | 2 | 2 | 2 | 0 | 0 | 1 | 0 | 3 | 26 | 0 | 7 | 0 |  |
| 8515 | 1 | 19.105 | 6.642 | 34.76577 | 65.23423 | 0 | 10.617385 | 1 | -0.27200794 | 3 | 104.30301 | 17.130556 | 83.25 | 1 | 0 | 5 | 1 | 1 | 0 | 1 | 1 | 1 |  | 115.1998574 | 1.379899739 | 97.02609731 | -0.469620094 | 1 | 2 |  | 0 | 0 | 1 | 0 | 3 |  | | | | |
| 8515 | 1 | 23.784 | 7.5 | 31.533804 | 68.466194 | 0 | 11.392197 | 1 | -0.27200794 | 3 | 104.30301 | 21.745554 | 76.370003 | 1 | 1 | 5 | 1 | 1 | 0 | 1 | 1 | 1 |  | 125.3366226 | 2.306790357 | 86.11837768 | -1.975308111 | 1 | 2 | 2 | 0 | 0 | 1 |  | 3 | 21 | 0 | 7 | 0 |  |
| 8515 | 1 | 16.368 | 5.2399998 | 32.013683 | 67.986313 | 0 | 11.468857 | 1 | -0.27200794 | 3 | 104.30301 | 20.368334 | 82.25 | 1 | 3 | 5 | 1 | 1 | 0 | 1 | 1 | 1 |  | | NA |  | NA | 1 | 2 | 2 | 0 | 0 | 1 |  | 3 | 23 | 0 | 7 | 0 |  |
| 8515 | 1 | 16.561001 | 5.48 | 33.089787 | 66.91021 | 0 | 11.545517 | 1 | -0.27200794 | 3 | 104.30301 | 19.439999 | 83.199997 | 1 | 5 | 5 | 1 | 1 | 0 | 0 | 0 | 0 |  | | NA |  | NA | 1 | 2 | 2 | 0 | 0 | 1 |  | 3 | 23 | 0 | 7 | 0 |  |
| 8515 | 1 | 18.988001 | 4.9499998 | 26.069094 | 73.930908 | 0 | 11.583847 | 1 | -0.27200794 | 3 | 104.30301 | 18.347223 | 84.589996 | 1 | 6 | 5 | 1 | 1 | 0 | 1 | 1 | 0 |  | 129.766692 | 2.709493398 | 90.26464562 | -1.44129564 | 1 | 2 | 2 | 0 | 0 | 1 | 0 | 3 | 24 | 0 | 6.8000002 | 1 |  |
| 8515 | 1 | 21.066999 | 4.6599998 | 22.119904 | 77.880096 | 0 | 11.909651 | 1 | -0.27200794 | 3 | 104.30301 | 17.553888 | 82.32 | 1 | 12 | 5 | 1 | 1 | 1 | 1 | 1 | 1 |  | 119.0866932 | 1.742922477 | 88.00573916 | -1.735439666 | 1 | 2 | 2 | 0 | 0 | 1 |  | 3 | 25 | 0 | 7 | 0 |  |
| 8520 | 1 | 19.53 | 6.884 | 35.248337 | 64.751663 | 1 | 14.795345 | 1 | 2.2397017 | 3 | 93.162628 | 16.642221 | 84.057 | 1 | 0 | 5 | 1 | 1 | 1 | 1 | 1 | 1 | 1 | 102.2976717 | 0.210078902 | 97.22945642 | -0.415660197 | 2 | 2 |  | 0 | 0 | 0 | 0 | 3 |  | | | | |
| 8520 | 0 | 23.115999 | 7.3400002 | 31.752901 | 68.247101 | 1 | 15.488022 | 1 | 2.2397017 | 3 | 93.162628 | 21.542778 | 76.809998 | 1 | 1 | 5 | 1 | 0 | 0 | 0 | 0 | 0 | 0 | 107.4718988 | 0.681480276 | 96.7059704 | -0.485795358 | 2 | 2 | 2 | 0 | 0 | 0 |  | 3 | 23 | 0 | 7 | 0 |  |
| 8520 | 0 | 15.938 | 5.0999999 | 31.998995 | 68.001007 | 1 | 15.564682 | 1 | 2.2397017 | 3 | 93.162628 | 20.323334 | 82.209999 | 1 | 3 | 5 | 1 | 0 | 0 | 0 | 0 | 0 | 0 |  | NA |  | NA | 2 | 2 | 2 | 0 | 0 | 0 |  | 3 | 23 | 0 | 7 | 0 |  |
| 8520 | 0 | 16.424 | 5.3899999 | 32.817825 | 67.182175 | 1 | 15.641341 | 1 | 2.2397017 | 3 | 93.162628 | 19.58889 | 82.220001 | 1 | 5 | 5 | 1 | 0 | 0 | 0 | 0 | 0 | 0 |  | NA |  | NA | 2 | 2 | 2 | 0 | 0 | 0 |  | 3 | 23 | 0 | 6.9000001 | 1 |  |
| 8520 | 0 | 20.120001 | 4.8800001 | 24.254473 | 75.745529 | 1 | 15.69336 | 1 | 2.2397017 | 3 | 93.162628 | 17.513889 | 86.110001 | 1 | 6 | 5 | 1 | 0 | 0 | 0 | 0 | 0 | 0 | 107.6636076 | 0.698675315 | 94.90337482 | -0.737353314 | 2 | 2 | 2 | 0 | 0 | 0 | 0 | 3 | 23 | 0 | 6.5999999 | 1 |  |
| 8520 | 0 | 20.768999 | 4.52 | 21.763206 | 78.236794 | 1 | 16.008213 | 1 | 2.2397017 | 3 | 93.162628 | 17.587778 | 81.529999 | 1 | 12 | 5 | 1 | 0 | 0 | 0 | 0 | 0 | 0 | 106.4558363 | 0.588482568 | 103.559433 | 0.54973057 | 2 | 2 | 2 | 0 | 0 | 0 |  | 3 | 25 | 0 | 7 | 0 |  |
| 8521 | 1 | 19.537 | 6.875 | 35.18964 | 64.810356 | 0 | 11.069131 | 0 | -1.6032 | 3 | 103.0221 | 16.538889 | 84.204 | 1 | 0 | 5 | 1 | 1 | 1 | 1 | 1 | 0 |  | 105.1232482 | 0.453794844 | 104.8621505 | 0.941263959 | 2 | 2 |  | 0 | 0 | 0 | 0 | 4 |  | | | | |
| 8521 | 1 | 23.139 | 7.3699999 | 31.850986 | 68.14901 | 0 | 11.830254 | 0 | -1.6032 | 3 | 103.0221 | 21.478891 | 77.059998 | 1 | 1 | 5 | 1 | 1 | 1 | 1 | 1 | 1 |  | 101.5121464 | 0.133907204 | 110.3967847 | 2.169097988 | 2 | 2 | 2 | 0 | 0 | 0 |  | 4 | 22 | 0 | 7 | 0 |  |
| 8521 | 0 | 16.245001 | 5.1500001 | 31.702061 | 68.297935 | 0 | 11.8987 | 0 | -1.6032 | 3 | 103.0221 | 20.347221 | 82.169998 | 1 | 3 | 5 | 1 | 0 | 0 | 0 | 0 | 0 |  | | NA |  | NA | 2 | 2 | 2 | 0 | 0 | 0 |  | 4 | 25 | 0 | 7 | 0 |  |
| 8521 | 1 | 16.544001 | 5.4299998 | 32.821564 | 67.178436 | 0 | 11.983573 | 0 | -1.6032 | 3 | 103.0221 | 19.455553 | 82.75 | 1 | 5 | 5 | 1 | 1 | 0 | 1 | 1 | 1 |  | | NA |  | NA | 2 | 2 | 2 | 0 | 0 | 0 |  | 4 | 23 | 0 | 6.5999999 | 1 |  |
| 8521 | 0 | 18.681999 | 4.9299998 | 26.389038 | 73.610962 | 1 | 12.021903 | 0 | -1.6032 | 3 | 103.0221 | 18.091667 | 84.959999 | 1 | 6 | 5 | 1 | 0 | 0 | 0 | 0 | 0 | 0 | 104.4014824 | 0.388365094 | 104.45926 | 0.848194419 | 2 | 2 | 2 | 0 | 0 | 0 | 0 | 4 | 24 | 0 | 5.6999998 | 1 |  |
| 8521 | 0 | 20.902 | 4.6100001 | 22.055305 | 77.944695 | 1 | 12.350445 | 0 | -1.6032 | 3 | 103.0221 | 17.509443 | 81.57 | 1 | 12 | 5 | 1 | 0 | 0 | 0 | 0 | 0 | 0 | 107.0658593 | 0.620728455 | 106.0288266 | 1.169230843 | 2 | 2 | 2 | 0 | 0 | 0 |  | 4 | 25 | 0 | 7 | 0 |  |
| 8523 | 1 | 18.154 | 6.156 | 33.909882 | 66.090118 | 1 | 17.399042 | 0 | 2.4412034 | 2 | 97.202202 | 21.13611 | 78.649 | 1 | 0 | 5 | 1 | 1 | 1 | 1 | 0 | 1 | 1 | 113.0115994 | 1.103915396 | 91.84331945 | -1.224099397 |  | | | | | 0 | 1 | 3 |  | | | | |
| 8523 | 1 | 24.121 | 7.46 | 30.927408 | 69.072594 | 1 | 18.143738 | 0 | 2.4412034 | 2 | 97.202202 | 21.59333 | 77.349998 | 1 | 1 | 5 | 1 | 1 | 0 | 0 | 0 | 1 | 1 | 116.3600354 | 1.379981375 | 96.03238556 | -0.624242024 |  | | 2 |  | | 0 |  | 3 | 24 | 0 | 7 | 0 |  |
| 8523 | 1 | 16.629999 | 5.71 | 34.335541 | 65.664459 | 1 | 18.217659 | 0 | 2.4412034 | 2 | 97.202202 | 20.531668 | 81.870003 | 1 | 3 | 5 | 1 | 1 | 0 | 0 | 0 | 1 | 1 |  | NA |  | NA |  | | 2 |  | | 0 |  | 3 | 25 | 0 | 7 | 0 |  |
| 8523 | 0 | 17.507999 | 5.6700001 | 32.385197 | 67.614799 | 1 | 18.294319 | 0 | 2.4412034 | 2 | 97.202202 | 19.861111 | 81.18 | 1 | 5 | 5 | 1 | 0 | 0 | 0 | 0 | 0 | 0 |  | NA |  | NA |  | | 2 |  | | 0 |  | 3 | 23 | 0 | 6.9000001 | 1 |  |
| 8523 | 0 | 19.278999 | 5.46 | 28.320972 | 71.679024 | 1 | 18.324436 | 0 | 2.4412034 | 2 | 97.202202 | 18.061111 | 85.540001 | 1 | 6 | 5 | 1 | 0 | 0 | 0 | 0 | 0 | 0 | 109.7425626 | 0.825612303 | 101.1256467 | 0.188652649 |  | | 2 |  | | 0 | 0 | 3 | 24 | 0 | 7 | 0 |  |
| 8523 | 1 | 19.216 | 4.52 | 23.522064 | 76.477936 | 1 | 18.620123 | 0 | 2.4412034 | 2 | 97.202202 | 17.861113 | 82.300003 | 1 | 12 | 5 | 1 | 1 | 1 | 1 | 0 | 0 | 1 | 114.3216203 | 1.207740886 | 104.4583095 | 0.776272833 |  | | 2 |  | | 0 |  | 3 | 15 | 1 | 4.1999998 | 1 |  |
| 8524 | 0 | 18.151 | 6.21 | 34.21299 | 65.78701 | 1 | 17.084188 | 1 | 1.352069 | 2 | 108.20254 | 20.591667 | 78.992 | 1 | 0 | 4 | 1 | 0 | 0 | 0 | 0 | 0 | 0 | 111.6599379 | 1.060244071 | 103.4922091 | 0.530630961 | 2 | 2 |  | 0 | 0 | 0 | 0 |  | | | | | |
| 8524 | 0 | 25.184 | 7.9200001 | 31.44854 | 68.55146 | 1 | 17.820671 | 1 | 1.352069 | 2 | 108.20254 | 21.876665 | 76.370003 | 1 | 1 | 4 | 1 | 0 | 0 | 0 | 0 | 0 | 0 | 104.2092808 | 0.383222653 | 103.8070781 | 0.575068655 | 2 | 2 | 2 | 0 | 0 | 0 |  | | 23 | 0 | 7 | 0 |  |
| 8524 | 0 | 16.796 | 5.7399998 | 34.174801 | 65.825195 | 1 | 17.902807 | 1 | 1.352069 | 2 | 108.20254 | 20.564444 | 81.730003 | 1 | 3 | 4 | 1 | 0 | 0 | 0 | 0 | 0 | 0 |  | NA |  | NA | 2 | 2 | 2 | 0 | 0 | 0 |  | | 25 | 0 | 7 | 0 |  |
| 8524 | 0 | 17.431999 | 5.6900001 | 32.641121 | 67.358879 | 1 | 17.979465 | 1 | 1.352069 | 2 | 108.20254 | 19.819447 | 81.489998 | 1 | 5 | 4 | 1 | 0 | 0 | 0 | 0 | 0 | 0 |  | NA |  | NA | 2 | 2 | 2 | 0 | 0 | 0 |  | | 24 | 0 | 7 | 0 |  |
| 8524 | 0 | 19.635 | 4.5599999 | 23.223835 | 76.776169 | 1 | 18.305271 | 1 | 1.352069 | 2 | 108.20254 | 17.859446 | 82.209999 | 1 | 12 | 4 | 1 | 0 | 0 | 0 | 0 | 0 | 0 | 105.5724449 | 0.506964053 | 102.4100221 | 0.358609936 | 2 | 2 | 2 | 0 | 0 | 0 |  | | 24 | 0 | 7 | 0 |  |
| 8527 | 1 | 19.856 | 7.572 | 38.134567 | 61.865433 | 1 | 17.352499 | 0 | -2.8658047 | 4 | 107.27195 | 20.571667 | 84.64 | 1 | 0 | 5 | 1 | 1 | 1 | 1 | 1 | 0 | 1 | 114.5283536 | 1.231359526 | 100.8295357 | 0.140216815 | 2 | 2 |  | 0 | 0 | 1 | 0 | 2 |  | | | | |
| 8527 | 1 | 27.106001 | 8.4499998 | 31.173908 | 68.826096 | 1 | 18.138262 | 0 | -2.8658047 | 4 | 107.27195 | 21.568333 | 78.93 | 1 | 1 | 5 | 1 | 1 | 1 | 1 | 0 | 1 | 1 | 115.7888013 | 1.332430959 | 97.25763292 | -0.438378144 | 2 | 2 | 2 | 0 | 0 | 1 |  | 2 | 16 | 1 | 7 | 0 |  |
| 8527 | 1 | 15.286 | 6.4000001 | 41.868378 | 58.131622 | 1 | 18.20397 | 0 | -2.8658047 | 4 | 107.27195 | 20.631113 | 82.349998 | 1 | 3 | 5 | 1 | 1 | 1 | 1 | 0 | 1 | 0 |  | NA |  | NA | 2 | 2 | 2 | 0 | 0 | 1 |  | 2 | 19 | 1 | 6.8000002 | 1 |  |
| 8527 | 1 | 18.974001 | 6.2600002 | 32.992516 | 67.007484 | 1 | 18.28063 | 0 | -2.8658047 | 4 | 107.27195 | 20.039444 | 81.639999 | 1 | 5 | 5 | 1 | 1 | 0 | 1 | 0 | 1 | 0 |  | NA |  | NA | 2 | 2 | 2 | 0 | 0 | 1 |  | 2 | 21 | 0 | 6.6999998 | 1 |  |
| 8527 | 1 | 20.042 | 5.8299999 | 29.088913 | 70.911087 | 1 | 18.32991 | 0 | -2.8658047 | 4 | 107.27195 | 17.565001 | 87.07 | 1 | 6 | 5 | 1 | 1 | 1 | 1 | 1 | 1 | 1 | 116.4083634 | 1.382667361 | 96.77472828 | -0.510934452 | 2 | 2 | 2 | 0 | 0 | 1 | 0 | 2 | 17 | 1 | 3.7 | 1 |  |
| 8527 | 0 | 18.556 | 4.5500002 | 24.520372 | 75.47963 | 1 | 18.65024 | 0 | -2.8658047 | 4 | 107.27195 | 17.760557 | 84.410004 | 1 | 12 | 5 | 1 | 0 | 0 | 0 | 0 | 0 | 0 | 111.4699986 | 0.969388236 | 97.75745943 | -0.358671678 | 2 | 2 | 2 | 0 | 0 | 1 |  | 2 | 22 | 0 | 6.6999998 | 1 |  |
| 8528 | 1 | 18.117 | 6.214 | 34.299278 | 65.700722 | 0 | 11.096509 | 0 | 2.2397017 | 3 | 115.38712 | 23.782223 | 77.42 | 1 | 0 | 5 | 0 | 1 | 1 | 1 | 1 | 1 |  | 119.3714519 | 1.695906395 | 103.2169298 | 0.606731334 | 2 | 2 |  | 1 | 0 | 1 | 0 | 2 |  | | | | |
| 8528 | 1 | 30.169001 | 9.1099997 | 30.196558 | 69.803444 | 0 | 11.696098 | 0 | 2.2397017 | 3 | 115.38712 | 22.024443 | 77.279999 | 1 | 1 | 5 | 0 | 1 | 0 | 0 | 0 | 0 |  | 112.6879425 | 1.113885921 | 108.1369603 | 1.643715668 | 2 | 2 | 2 | 1 | 0 | 1 |  | 2 | 27 | 0 | 7 | 0 |  |
| 8528 | 1 | 16.156 | 6.5500002 | 40.542213 | 59.457787 | 0 | 11.778234 | 0 | 2.2397017 | 3 | 115.38712 | 20.706114 | 82.379997 | 1 | 3 | 5 | 0 | 1 | 0 | 0 | 0 | 0 |  | | NA |  | NA | 2 | 2 | 2 | 1 | 0 | 1 |  | 2 | 27 | 0 | 7 | 0 |  |
| 8528 | 0 | 19.025 | 6.2800002 | 33.009201 | 66.990799 | 0 | 11.854894 | 0 | 2.2397017 | 3 | 115.38712 | 20.047222 | 81.650002 | 1 | 5 | 5 | 0 | 0 | 0 | 0 | 0 | 0 |  | | NA |  | NA | 2 | 2 | 2 | 1 | 0 | 1 |  | 2 | 26 | 0 | 7 | 0 |  |
| 8528 | 0 | 20.306999 | 5.8000002 | 28.561583 | 71.438416 | 0 | 11.917865 | 0 | 2.2397017 | 3 | 115.38712 | 17.389444 | 87.260002 | 1 | 6 | 5 | 0 | 0 | 0 | 0 | 0 | 0 |  | 117.6324209 | 1.539944045 | 104.7394653 | 0.906491214 | 2 | 2 | 2 | 1 | 0 | 1 | 0 | 2 | 27 | 0 | 7 | 0 |  |
| 8528 | 0 | 17.476 | 4.8200002 | 27.580683 | 72.419319 | 1 | 12.180698 | 0 | 2.2397017 | 3 | 115.38712 | 17.169998 | 86.339996 | 1 | 12 | 5 | 0 | 0 | 0 | 0 | 0 | 0 | 0 | 124.5638826 | 2.130409389 | 105.654418 | 1.092928023 | 2 | 2 | 2 | 1 | 0 | 1 |  | 2 | 24 | 0 | 7 | 0 |  |
| 8529 | 0 | 17.746 | 5.959 | 33.579399 | 66.420601 | 0 | 10.455853 | 0 | 0.11822473 | 3 | 134.15335 | 16.842777 | 82.323 | 1 | 0 | 4 | 1 | 0 | 0 | 0 | 0 | 0 |  | 136.4310192 | 3.151325958 | 105.3268086 | 1.045823713 | 2 | 2 |  | 0 | 0 | 0 | 0 | 3 |  | | | | |
| 8529 | 0 | 22.506001 | 7.23 | 32.124763 | 67.875237 | 0 | 11.195072 | 0 | 0.11822473 | 3 | 134.15335 | 20.916113 | 78.910004 | 1 | 1 | 4 | 1 | 0 | 0 | 0 | 0 | 0 |  | 113.6296958 | 1.198306117 | 104.3262399 | 0.829330127 | 2 | 2 | 2 | 0 | 0 | 0 |  | 3 | 27 | 0 | 7 | 0 |  |
| 8529 | 0 | 15.876 | 4.9099998 | 30.927185 | 69.072815 | 0 | 11.271731 | 0 | 0.11822473 | 3 | 134.15335 | 20.096668 | 82.580002 | 1 | 3 | 4 | 1 | 0 | 0 | 0 | 0 | 0 |  | | NA |  | NA | 2 | 2 | 2 | 0 | 0 | 0 |  | 3 | 27 | 0 | 7 | 0 |  |
| 8529 | 0 | 16.812 | 5.4400001 | 32.357841 | 67.642159 | 0 | 11.348392 | 0 | 0.11822473 | 3 | 134.15335 | 19.173334 | 83.25 | 1 | 5 | 4 | 1 | 0 | 0 | 0 | 0 | 0 |  | | NA |  | NA | 2 | 2 | 2 | 0 | 0 | 0 |  | 3 | 26 | 0 | 7 | 0 |  |
| 8529 | 1 | 21.332001 | 4.7600002 | 22.313894 | 77.686104 | 0 | 11.737166 | 0 | 0.11822473 | 3 | 134.15335 | 17.228334 | 80.669998 | 1 | 12 | 4 | 1 | 1 | 1 | 1 | 1 | 1 |  | 110.5567238 | 0.928204933 | 101.4079537 | 0.256447068 | 2 | 2 | 2 | 0 | 0 | 0 |  | 3 | 20 | 0 | 7 | 0 |  |
| 8530 | 1 | 18.378 | 6.203 | 33.752312 | 66.247688 | 1 | 15.939767 | 0 | 1.1814024 | 3 | 145.53185 | 15.566112 | 84.237 | 1 | 0 | 4 | 1 | 0 | 0 | 1 | 0 | 1 | 0 | 163.0004835 | 5.195689119 | 95.63181234 | -0.699942764 | 2 | 2 |  | 0 | 0 | 0 | 0 | 4 |  | | | | |
| 8530 | 0 | 22.075001 | 7.1300001 | 32.298981 | 67.701019 | 1 | 16.70089 | 0 | 1.1814024 | 3 | 145.53185 | 20.631668 | 79.75 | 1 | 1 | 4 | 1 | 0 | 0 | 0 | 0 | 0 | 0 | 153.6903457 | 4.437437178 | 94.80045847 | -0.817426178 | 2 | 2 | 2 | 0 | 0 | 0 |  | 4 | 24 | 0 | 7 | 0 |  |
| 8530 | 0 | 15.511 | 4.7399998 | 30.558956 | 69.44104 | 1 | 16.77755 | 0 | 1.1814024 | 3 | 145.53185 | 19.963888 | 82.870003 | 1 | 3 | 4 | 1 | 0 | 0 | 0 | 0 | 0 | 0 |  | NA |  | NA | 2 | 2 | 2 | 0 | 0 | 0 |  | 4 | 25 | 0 | 7 | 0 |  |
| 8530 | 0 | 16.872999 | 5.46 | 32.359394 | 67.64061 | 1 | 16.85421 | 0 | 1.1814024 | 3 | 145.53185 | 19.06111 | 83.449997 | 1 | 5 | 4 | 1 | 0 | 0 | 0 | 0 | 0 | 0 |  | NA |  | NA | 2 | 2 | 2 | 0 | 0 | 0 |  | 4 | 24 | 0 | 7 | 0 |  |
| 8530 | 0 | 21.669001 | 4.8200002 | 22.243759 | 77.756241 | 1 | 17.188227 | 0 | 1.1814024 | 3 | 145.53185 | 17.069443 | 80.410004 | 1 | 12 | 4 | 1 | 0 | 0 | 0 | 0 | 0 | 0 | 136.9780609 | 3.083979344 | 99.07043176 | -0.153856082 | 2 | 2 | 2 | 0 | 0 | 0 |  | 4 | 24 | 0 | 7 | 0 |  |
| 8531 | 0 | 18.349 | 6.221 | 33.903755 | 66.096245 | 1 | 12.928131 | 1 | 2.3033004 | 3 | 107.95876 | 16.776667 | 82.796 | 1 | 0 | 5 | 1 | 0 | 0 | 0 | 0 | 0 | 0 | 124.1559203 | 2.203919108 | 93.57412407 | -0.967210893 | 2 | 2 |  | 1 | 0 | 0 | 0 | 3 |  | | | | |
| 8531 | 0 | 22.754 | 7.29 | 32.038322 | 67.961678 | 1 | 13.683778 | 1 | 2.3033004 | 3 | 107.95876 | 21.111111 | 78.300003 | 1 | 1 | 5 | 1 | 0 | 0 | 0 | 0 | 0 | 0 | 120.9229277 | 1.908347137 | 97.13063761 | -0.439603758 | 2 | 2 | 2 | 1 | 0 | 0 |  | 3 | 24 | 0 | 7 | 0 |  |
| 8531 | 0 | 15.988 | 4.98 | 31.148361 | 68.851639 | 1 | 13.760438 | 1 | 2.3033004 | 3 | 107.95876 | 20.165554 | 82.459999 | 1 | 3 | 5 | 1 | 0 | 0 | 0 | 0 | 0 | 0 |  | NA |  | NA | 2 | 2 | 2 | 1 | 0 | 0 |  | 3 | 25 | 0 | 7 | 0 |  |
| 8531 | 0 | 16.697001 | 5.4499998 | 32.640591 | 67.359406 | 1 | 13.837098 | 1 | 2.3033004 | 3 | 107.95876 | 19.23 | 83.230003 | 1 | 5 | 5 | 1 | 0 | 0 | 0 | 0 | 0 | 0 |  | NA |  | NA | 2 | 2 | 2 | 1 | 0 | 0 |  | 3 | 24 | 0 | 7 | 0 |  |
| 8531 | 0 | 17.926001 | 4.9499998 | 27.61352 | 72.386482 | 1 | 13.883641 | 1 | 2.3033004 | 3 | 107.95876 | 17.943331 | 84.889999 | 1 | 6 | 5 | 1 | 0 | 0 | 0 | 0 | 0 | 0 | 126.7618765 | 2.436868287 | 97.97660632 | -0.311023199 | 2 | 2 | 2 | 1 | 0 | 0 | 0 | 3 | 19 | 1 | 7 | 0 |  |
| 8531 | 1 | 21.232 | 4.73 | 22.277695 | 77.722305 | 1 | 14.168378 | 1 | 2.3033004 | 3 | 107.95876 | 17.277222 | 81.07 | 1 | 12 | 5 | 1 | 1 | 0 | 1 | 0 | 1 | 1 | 121.6637792 | 1.973911621 | 99.30581561 | -0.107296209 | 2 | 2 | 2 | 1 | 0 | 0 |  | 3 | 23 | 0 | 6.8000002 | 1 |  |
| 8534 | 1 | 18.702 | 6.442 | 34.445515 | 65.554489 | 1 | 13.530458 | 1 | 2.4412034 | 3 | 107.70673 | 17.568333 | 81.916 | 1 | 0 | 3 | 1 | 1 | 1 | 0 | 0 | 1 | 1 | 113.212149 | 1.207611036 | 101.4908042 | 0.237737229 |  | | | | | 0 | 0 | 3 |  | | | | |
| 8534 | 1 | 23.115 | 7.3899999 | 31.970581 | 68.029419 | 1 | 14.30527 | 1 | 2.4412034 | 3 | 107.70673 | 21.751112 | 76.089996 | 1 | 1 | 3 | 1 | 1 | 1 | 1 | 1 | 1 | 1 | 110.9195409 | 0.997191921 | 101.7282097 | 0.271773402 |  | | 2 |  | | 0 |  | 3 | 18 | 1 | 7 | 0 |  |
| 8534 | 1 | 18.538 | 4.8299999 | 26.05459 | 73.945412 | 1 | 14.494182 | 1 | 2.4412034 | 3 | 107.70673 | 18.363335 | 84.510002 | 1 | 6 | 3 | 1 | 1 | 1 | 1 | 0 | 1 | 1 | 116.8121283 | 1.532649064 | 102.8369913 | 0.448442046 |  | | 2 |  | | 0 | 0 | 3 | 24 | 0 | 6.0999999 | 1 |  |
| 8534 | 1 | 20.635 | 4.4299998 | 21.468378 | 78.531624 | 1 | 14.822724 | 1 | 2.4412034 | 3 | 107.70673 | 17.60111 | 81.610001 | 1 | 12 | 3 | 1 | 1 | 1 | 1 | 1 | 1 | 1 | 113.336106 | 1.21596027 | 103.9007655 | 0.617692086 |  | | 2 |  | | 0 |  | 3 | 25 | 0 | 7 | 0 |  |
| 8538 | 1 | 19.508 | 6.723 | 34.462784 | 65.537216 | 1 | 15.134839 | 0 | 1.9938918 | 2 | 87.336433 | 15.061111 | 85.533 | 1 | 0 | 5 | 0 | 1 | 0 | 0 | 0 | 1 | 0 | 98.45308832 | -0.134398114 | 94.9173555 | -0.812918421 | 2 | 2 |  | 0 | 0 | 0 | 0 | 4 |  | | | | |
| 8538 | 0 | 22.243 | 7.1700001 | 32.234859 | 67.765137 | 1 | 15.874059 | 0 | 1.9938918 | 2 | 87.336433 | 20.748888 | 79.400002 | 1 | 1 | 5 | 0 | 0 | 0 | 0 | 0 | 0 | 0 | 101.9618972 | 0.169229982 | 97.01122211 | -0.488392121 | 2 | 2 | 2 | 0 | 0 | 0 |  | 4 | 23 | 0 | 7 | 0 |  |
| 8538 | 0 | 15.642 | 4.8000002 | 30.686615 | 69.313385 | 1 | 15.950719 | 0 | 1.9938918 | 2 | 87.336433 | 20.015556 | 82.760002 | 1 | 3 | 5 | 0 | 0 | 0 | 0 | 0 | 0 | 0 |  | NA |  | NA | 2 | 2 | 2 | 0 | 0 | 0 |  | 4 | 23 | 0 | 7 | 0 |  |
| 8538 | 0 | 16.836 | 5.46 | 32.430508 | 67.569496 | 1 | 16.027378 | 0 | 1.9938918 | 2 | 87.336433 | 19.104445 | 83.379997 | 1 | 5 | 5 | 0 | 0 | 0 | 0 | 0 | 0 | 0 |  | NA |  | NA | 2 | 2 | 2 | 0 | 0 | 0 |  | 4 | 24 | 0 | 7 | 0 |  |
| 8538 | 1 | 16.995001 | 4.9099998 | 28.890848 | 71.109154 | 1 | 16.073921 | 0 | 1.9938918 | 2 | 87.336433 | 17.843891 | 84.980003 | 1 | 6 | 5 | 0 | 1 | 1 | 0 | 0 | 1 | 1 | 104.0797523 | 0.350906821 | 102.5874134 | 0.454984428 | 2 | 2 | 1 | 0 | 0 | 0 | 0 | 4 | 15 | 1 | 5.0999999 | 1 |  |
| 8538 | 0 | 19.625999 | 5.02 | 25.578316 | 74.421684 | 1 | 16.361397 | 0 | 1.9938918 | 2 | 87.336433 | 16.355 | 83.849998 | 1 | 12 | 5 | 0 | 0 | 0 | 0 | 0 | 0 | 0 | 105.3801726 | 0.461571289 | 98.25514131 | -0.288435387 | 2 | 2 | 2 | 0 | 0 | 0 |  | 4 | 24 | 0 | 7 | 0 |  |
| 8539 | 1 | 17.569 | 5.868 | 33.399738 | 66.600266 | 1 | 14.069816 | 0 | 2.4412034 | 4 | 105.57682 | 21.696112 | 77.096 | 1 | 0 | 4 | 0 | 0 | 0 | 1 | 1 | 1 | 0 | 112.0662817 | 1.043262632 | 100.7093199 | 0.124557051 | 2 | 2 |  | 0 | 0 | 0 | 0 |  | | | | | |
| 8539 | 1 | 23.881001 | 7.3099999 | 30.610107 | 69.389893 | 1 | 14.784394 | 0 | 2.4412034 | 4 | 105.57682 | 21.440002 | 78.07 | 1 | 1 | 4 | 0 | 0 | 0 | 0 | 0 | 1 | 0 | 105.7445443 | 0.496901755 | 98.35241802 | -0.277456801 | 2 | 2 | 2 | 0 | 0 | 0 |  | | 24 | 0 | 7 | 0 |  |
| 8539 | 1 | 16.145 | 5.7199998 | 35.428925 | 64.571075 | 1 | 14.850102 | 0 | 2.4412034 | 4 | 105.57682 | 20.503887 | 81.800003 | 1 | 3 | 4 | 0 | 0 | 0 | 0 | 0 | 1 | 0 |  | NA |  | NA | 2 | 2 | 2 | 0 | 0 | 0 |  | | 25 | 0 | 7 | 0 |  |
| 8539 | 0 | 17.496 | 5.6799998 | 32.464561 | 67.535439 | 1 | 14.926763 | 0 | 2.4412034 | 4 | 105.57682 | 19.846666 | 81.290001 | 1 | 5 | 4 | 0 | 0 | 0 | 0 | 0 | 0 | 0 |  | NA |  | NA | 2 | 2 | 2 | 0 | 0 | 0 |  | | 23 | 0 | 6.9000001 | 1 |  |
| 8539 | 0 | 19.367001 | 4.54 | 23.441936 | 76.558067 | 1 | 15.252566 | 0 | 2.4412034 | 4 | 105.57682 | 17.855555 | 82.269997 | 1 | 12 | 4 | 0 | 0 | 0 | 0 | 0 | 0 | 0 | 106.8281081 | 0.58853934 | 99.13882952 | -0.145912227 | 2 | 2 | 2 | 0 | 0 | 0 |  | | 25 | 0 | 7 | 0 |  |
| 8540 | 0 | 18.426 | 6.533 | 35.455334 | 64.544662 | 1 | 14.655715 | 0 | -0.46975747 | 2 | 101.06322 | 23.059999 | 79.041 | 1 | 0 | 4 | 0 | 0 | 0 | 0 | 0 | 0 | 0 | 115.2853627 | 1.313093916 | 93.61587584 | -1.006899228 | 2 | 2 |  | 0 | 0 | 0 | 0 | 4 |  | | | | |
| 8540 | 0 | 28.27 | 8.71 | 30.810045 | 69.189957 | 1 | 15.403149 | 0 | -0.46975747 | 2 | 101.06322 | 21.730555 | 78.330002 | 1 | 1 | 4 | 0 | 0 | 0 | 0 | 0 | 0 | 0 | 103.6792298 | 0.317697853 | 93.15767452 | -1.065031776 | 2 | 2 | 2 | 0 | 0 | 0 |  | 4 | 21 | 0 | 7 | 0 |  |
| 8540 | 1 | 15.595 | 6.5100002 | 41.744148 | 58.255852 | 1 | 15.474333 | 0 | -0.46975747 | 2 | 101.06322 | 20.660002 | 82.480003 | 1 | 3 | 4 | 0 | 1 | 0 | 1 | 0 | 1 | 1 |  | NA |  | NA | 2 | 2 | 2 | 0 | 0 | 0 |  | 4 | 22 | 0 | 6.5 | 1 |  |
| 8540 | 1 | 19.073999 | 6.3000002 | 33.029259 | 66.970741 | 1 | 15.550992 | 0 | -0.46975747 | 2 | 101.06322 | 20.054445 | 81.669998 | 1 | 5 | 4 | 0 | 1 | 0 | 0 | 0 | 1 | 0 |  | NA |  | NA | 2 | 2 | 2 | 0 | 0 | 0 |  | 4 | 22 | 0 | 6.9000001 | 1 |  |
| 8540 | 1 | 18.504 | 4.54 | 24.535236 | 75.464767 | 1 | 15.92334 | 0 | -0.46975747 | 2 | 101.06322 | 17.750555 | 84.580002 | 1 | 12 | 4 | 0 | 1 | 0 | 0 | 0 | 1 | 0 | 105.6022097 | 0.481604318 | 91.91032957 | -1.231739886 | 2 | 2 | 2 | 0 | 0 | 0 |  | 4 | 23 | 0 | 6.6999998 | 1 |  |
| 8543 | 0 | 17.444 | 5.64 | 32.332035 | 67.667969 | 1 | 16.416153 | 1 | 2.0574906 | 2 | 70.090446 | 19.584999 | 77.385 | 1 | 0 | 5 | 1 | 0 | 0 | 0 | 0 | 0 | 0 | 72.51938844 | -2.527752741 | 103.4103564 | 0.522704927 | 2 | 2 |  | 1 | 0 | 1 | 0 | 3 |  | | | | |
| 8543 | 0 | 23.752001 | 7.48 | 31.492085 | 68.507919 | 1 | 16.588638 | 1 | 2.0574906 | 2 | 70.090446 | 21.714998 | 76.379997 | 1 | 1 | 5 | 1 | 0 | 0 | 0 | 0 | 0 | 0 | 74.43460195 | -2.349687381 | 106.2095849 | 0.970174975 | 2 | 2 | 2 | 1 | 0 | 1 |  | 3 | 18 | 1 | 4.8000002 | 1 |  |
| 8543 | 0 | 16.247 | 5.2800002 | 32.49831 | 67.501694 | 1 | 16.66256 | 1 | 2.0574906 | 2 | 70.090446 | 20.347776 | 82.389999 | 1 | 3 | 5 | 1 | 0 | 0 | 0 | 0 | 0 | 0 |  | NA |  | NA | 2 | 2 | 2 | 1 | 0 | 1 |  | 3 | 25 | 0 | 7 | 0 |  |
| 8543 | 0 | 16.509001 | 5.48 | 33.194012 | 66.805984 | 1 | 16.73922 | 1 | 2.0574906 | 2 | 70.090446 | 19.505001 | 82.980003 | 1 | 5 | 5 | 1 | 0 | 0 | 0 | 0 | 0 | 0 |  | NA |  | NA | 2 | 2 | 2 | 1 | 0 | 1 |  | 3 | 23 | 0 | 6.9000001 | 1 |  |
| 8543 | 1 | 20.054001 | 4.9400001 | 24.63349 | 75.366508 | 1 | 16.780287 | 1 | 2.0574906 | 2 | 70.090446 | 18.022779 | 85.32 | 1 | 6 | 5 | 1 | 1 | 0 | 0 | 0 | 1 | 0 | 73.48821562 | -2.436883497 | 108.4545771 | 1.34013027 | 2 | 2 | 2 | 1 | 0 | 1 | 0 | 3 | 14 | 1 | 5.5999999 | 1 |  |
| 8543 | 0 | 21.202 | 4.6700001 | 22.026224 | 77.973778 | 1 | 17.067762 | 1 | 2.0574906 | 2 | 70.090446 | 17.639999 | 82.449997 | 1 | 12 | 5 | 1 | 0 | 0 | 0 | 0 | 0 | 0 | 74.95020891 | -2.300811024 | 108.2381944 | 1.2985896 | 2 | 2 | 2 | 1 | 0 | 1 |  | 3 | 24 | 0 | 7 | 0 |  |
| 8544 | 0 | 18.065 | 5.931 | 32.831444 | 67.168556 | 1 | 15.32375 | 0 | 2.3033004 | 2 | 76.455338 | 18.166111 | 80.935 | 1 | 0 | 5 | 1 | 0 | 0 | 0 | 0 | 0 | 0 | 81.53026361 | -1.628881343 | 99.2459936 | -0.127841167 | 2 | 2 |  | 1 | 0 | 0 | 0 | 1 |  | | | | |
| 8544 | 1 | 24.186001 | 7.54 | 31.175058 | 68.824944 | 1 | 16.041067 | 0 | 2.3033004 | 2 | 76.455338 | 21.527779 | 77.169998 | 1 | 1 | 5 | 1 | 1 | 0 | 1 | 0 | 1 | 0 | 88.04762237 | -1.043297528 | 99.96532141 | -0.005888273 | 2 | 2 | 2 | 1 | 0 | 0 |  | 1 | 21 | 0 | 6 | 1 |  |
| 8544 | 0 | 16.747999 | 5.3899999 | 32.182949 | 67.817055 | 1 | 16.117727 | 0 | 2.3033004 | 2 | 76.455338 | 20.249998 | 82.730003 | 1 | 3 | 5 | 1 | 0 | 0 | 0 | 0 | 0 | 0 |  | NA |  | NA | 2 | 2 | 2 | 1 | 0 | 0 |  | 1 | 25 | 0 | 7 | 0 |  |
| 8544 | 1 | 16.966999 | 5.5999999 | 33.005245 | 66.994751 | 1 | 16.194387 | 0 | 2.3033004 | 2 | 76.455338 | 19.406111 | 83.389999 | 1 | 5 | 5 | 1 | 1 | 0 | 1 | 0 | 1 | 0 |  | NA |  | NA | 2 | 2 | 2 | 1 | 0 | 0 |  | 1 | 24 | 0 | 7 | 0 |  |
| 8544 | 1 | 19.607 | 5.0700002 | 25.858112 | 74.141884 | 1 | 16.232718 | 0 | 2.3033004 | 2 | 76.455338 | 18.069445 | 85.239998 | 1 | 6 | 5 | 1 | 1 | 0 | 0 | 0 | 1 | 0 | 92.40352165 | -0.659705079 | 102.4850662 | 0.43550102 | 2 | 2 | 2 | 1 | 0 | 0 | 0 | 1 | 24 | 0 | 7 | 0 |  |
| 8544 | 0 | 21.6 | 4.9200001 | 22.777779 | 77.222221 | 1 | 16.585899 | 0 | 2.3033004 | 2 | 76.455338 | 17.586111 | 82.980003 | 1 | 12 | 5 | 1 | 0 | 0 | 0 | 0 | 0 | 0 | 82.78426456 | -1.506436387 | 99.73155848 | -0.045145587 | 2 | 2 | 2 | 1 | 0 | 0 |  | 1 | 24 | 0 | 7 | 0 |  |
| 8545 | 1 | 18.988 | 6.549 | 34.490204 | 65.509796 | 1 | 13.05681 | 1 | -2.3150556 | 3 | 120.61158 | 17.173334 | 83.301 | 1 | 0 | 4 | 0 | 1 | 0 | 1 | 1 | 0 | 0 | 125.8773201 | 2.359796148 | 102.4387814 | 0.395717786 | 2 | 2 |  | 0 | 0 | 0 | 0 |  | | | | | |
| 8545 | 1 | 24.162001 | 7.5599999 | 31.288799 | 68.711205 | 1 | 13.823409 | 1 | -2.3150556 | 3 | 120.61158 | 21.565556 | 77.010002 | 1 | 1 | 4 | 0 | 1 | 0 | 1 | 1 | 1 | 0 | 99.61396244 | -0.035383874 | 100.0894524 | 0.014014621 | 2 | 2 | 1 | 0 | 0 | 0 |  | | 14 | 1 | 4.1999998 | 1 |  |
| 8545 | 0 | 16.783001 | 5.4099998 | 32.234997 | 67.765007 | 1 | 13.900068 | 1 | -2.3150556 | 3 | 120.61158 | 20.273333 | 82.610001 | 1 | 3 | 4 | 0 | 0 | 0 | 0 | 0 | 0 | 0 |  | NA |  | NA | 2 | 2 | 1 | 0 | 0 | 0 |  | | 14 | 1 | 7 | 0 |  |
| 8545 | 1 | 16.908001 | 5.5999999 | 33.120415 | 66.879585 | 1 | 13.976728 | 1 | -2.3150556 | 3 | 120.61158 | 19.413889 | 83.309998 | 1 | 5 | 4 | 0 | 0 | 0 | 0 | 0 | 1 | 0 |  | NA |  | NA | 2 | 2 | 2 | 0 | 0 | 0 |  | | 18 | 1 | 5.5999999 | 1 |  |
| 8545 | 0 | 21.601999 | 4.9299998 | 22.82196 | 77.17804 | 1 | 14.30527 | 1 | -2.3150556 | 3 | 120.61158 | 17.577223 | 82.900002 | 1 | 12 | 4 | 0 | 0 | 0 | 0 | 0 | 0 | 0 | 124.8309799 | 2.260312591 | 102.7028769 | 0.428497015 | 2 | 2 | 2 | 0 | 0 | 0 |  | | 11 | 1 | 4 | 1 |  |
| 8546 |  | 18.558 | 6.325 | 34.082336 | 65.917664 | 1 | 16.490076 | 1 | 2.0574906 | 3 | 108.84109 | 16.850555 | 82.886 | 1 | 0 | 4 | 1 |  | | | | | | 126.7672769 | 2.426981823 | 92.03542924 | -1.113102059 | 2 | 2 |  | 0 | 0 | 0 | 0 | 4 |  | | | | |
| 8546 | 0 | 22.915001 | 7.3299999 | 31.98778 | 68.012222 | 1 | 17.251198 | 1 | 2.0574906 | 3 | 108.84109 | 21.201113 | 78.040001 | 1 | 1 | 4 | 1 | 0 | 0 | 0 | 0 | 0 | 0 | 123.9389162 | 2.170370564 | 97.19637658 | -0.404607353 | 2 | 2 | 2 | 0 | 0 | 0 |  | 4 | 24 | 0 | 7 | 0 |  |
| 8546 | 0 | 16.107 | 5.04 | 31.290743 | 68.709259 | 1 | 17.327858 | 1 | 2.0574906 | 3 | 108.84109 | 20.201111 | 82.389999 | 1 | 3 | 4 | 1 | 0 | 0 | 0 | 0 | 0 | 0 |  | NA |  | NA | 2 | 2 | 2 | 0 | 0 | 0 |  | 4 | 25 | 0 | 7 | 0 |  |
| 8546 | 0 | 16.677999 | 5.4499998 | 32.67778 | 67.32222 | 1 | 17.404518 | 1 | 2.0574906 | 3 | 108.84109 | 19.260555 | 83.209999 | 1 | 5 | 4 | 1 | 0 | 0 | 0 | 0 | 0 | 0 |  | NA |  | NA | 2 | 2 | 2 | 0 | 0 | 0 |  | 4 | 24 | 0 | 7 | 0 |  |
| 8546 | 0 | 21.156 | 4.7199998 | 22.310455 | 77.689545 | 1 | 17.738535 | 1 | 2.0574906 | 3 | 108.84109 | 17.353333 | 81.110001 | 1 | 12 | 4 | 1 | 0 | 0 | 0 | 0 | 0 | 0 | 109.4127634 | 0.855912726 | 102.6131678 | 0.391419341 | 2 | 2 | 2 | 0 | 0 | 0 |  | 4 | 24 | 0 | 7 | 0 |  |
| 8547 | 1 | 18.631 | 6.436 | 34.544575 | 65.455421 | 1 | 16.57221 | 0 | 1.5067414 | 2 | 105.24635 | 18.282778 | 80.92 | 1 | 0 | 5 | 1 | 1 | 1 | 1 | 1 | 1 | 1 | 104.0709242 | 0.349283561 | 108.1703901 | 1.536684789 | 2 | 2 |  | 0 | 0 | 0 | 0 | 3 |  | | | | |
| 8547 | 1 | 23.299999 | 7.46 | 32.01717 | 67.982834 | 1 | 17.336071 | 0 | 1.5067414 | 2 | 105.24635 | 22.04833 | 75.120003 | 1 | 1 | 5 | 1 | 1 | 0 | 0 | 0 | 1 | 0 | 99.45702951 | -0.046611123 | 104.4190265 | 0.782813095 | 2 | 2 | 2 | 0 | 0 | 0 |  | 3 | 23 | 0 | 7 | 0 |  |
| 8547 | 1 | 15.869 | 5.1799998 | 32.642258 | 67.357742 | 1 | 17.412731 | 0 | 1.5067414 | 2 | 105.24635 | 20.534998 | 81.699997 | 1 | 3 | 5 | 1 | 1 | 1 | 0 | 0 | 1 | 1 |  | NA |  | NA | 2 | 2 | 2 | 0 | 0 | 0 |  | 3 | 25 | 0 | 7 | 0 |  |
| 8547 | 0 | 15.969 | 5.3000002 | 33.189304 | 66.810692 | 1 | 17.489391 | 0 | 1.5067414 | 2 | 105.24635 | 19.654999 | 82.330002 | 1 | 5 | 5 | 1 | 0 | 0 | 0 | 0 | 0 | 0 |  | NA |  | NA | 2 | 2 | 2 | 0 | 0 | 0 |  | 3 | 23 | 0 | 6.9000001 | 1 |  |
| 8547 | 0 | 20.459 | 4.8200002 | 23.559315 | 76.440689 | 1 | 17.533195 | 0 | 1.5067414 | 2 | 105.24635 | 18.048334 | 85.169998 | 1 | 6 | 5 | 1 | 0 | 0 | 0 | 0 | 0 | 0 | 96.13785637 | -0.332232545 | 101.8772784 | 0.32091703 | 2 | 2 | 2 | 0 | 0 | 0 | 0 | 3 | 24 | 0 | 7 | 0 |  |
| 8547 | 0 | 20.162001 | 4.25 | 21.079256 | 78.920746 | 1 | 17.856262 | 0 | 1.5067414 | 2 | 105.24635 | 17.758333 | 81.639999 | 1 | 12 | 5 | 1 | 0 | 0 | 0 | 0 | 0 | 0 | 102.7251103 | 0.232745952 | 83.02689435 | -2.25642297 | 2 | 2 | 2 | 0 | 0 | 0 |  | 3 | 24 | 0 | 7 | 0 |  |
| 8550 | 1 | 18.796 | 6.547 | 34.831879 | 65.168121 | 1 | 14.099932 | 0 | 0.15852845 | 3 | 118.95365 | 18.35111 | 81.151 | 1 | 0 | 4 | 1 | 1 | 0 | 0 | 1 | 1 | 0 | 130.1271261 | 2.568250472 | 97.75678592 | -0.377442188 | 2 | 2 |  | 0 | 0 | 1 | 0 | 1 |  | | | | |
| 8550 | 1 | 15.988 | 5.25 | 32.837128 | 67.162872 | 1 | 14.945928 | 0 | 0.15852845 | 3 | 118.95365 | 20.582781 | 81.559998 | 1 | 3 | 4 | 1 | 1 | 1 | 1 | 1 | 0 | 1 |  | NA |  | NA | 2 | 2 | 2 | 0 | 0 | 1 |  | 1 | 18 | 1 | 4.1999998 | 1 |  |
| 8550 | 1 | 16.016001 | 5.3400002 | 33.341656 | 66.65834 | 1 | 15.022587 | 0 | 0.15852845 | 3 | 118.95365 | 19.678331 | 82.290001 | 1 | 5 | 4 | 1 | 1 | 0 | 0 | 0 | 1 | 0 |  | NA |  | NA | 2 | 2 | 2 | 0 | 0 | 1 |  | 1 | 23 | 0 | 7 | 0 |  |
| 8550 | 0 | 20.608 | 4.8499999 | 23.53455 | 76.465454 | 1 | 15.066393 | 0 | 0.15852845 | 3 | 118.95365 | 18.060556 | 85.169998 | 1 | 6 | 4 | 1 | 0 | 0 | 0 | 0 | 0 | 0 | 110.6615577 | 0.917040949 | 92.51802731 | -1.157665303 | 2 | 2 | 2 | 0 | 0 | 1 | 0 | 1 | 19 | 1 | 5.9000001 | 1 |  |
| 8550 | 0 | 20.17 | 4.27 | 21.170055 | 78.829948 | 1 | 15.35113 | 0 | 0.15852845 | 3 | 118.95365 | 17.78389 | 81.769997 | 1 | 12 | 4 | 1 | 0 | 0 | 0 | 0 | 0 | 0 | 112.3739035 | 1.061058034 | 88.23852608 | -1.709240267 | 2 | 2 | 2 | 0 | 0 | 1 |  | 1 | 21 | 0 | 6.6999998 | 1 |  |
| 8551 | 1 | 17.711 | 5.999 | 33.871605 | 66.128395 | 0 | 10.694045 | 0 | 2.4412034 | 2 | 113.54542 | 19.135 | 78.603 | 1 | 0 | 5 | 1 | 1 | 1 | 1 | 1 | 1 |  | 125.7451606 | 2.244614409 | 97.5051263 | -0.431284568 |  | | | | | 0 | 0 | 3 |  | | | | |
| 8551 | 1 | 23.504 | 7.5100002 | 31.952009 | 68.047989 | 0 | 11.427789 | 0 | 2.4412034 | 2 | 113.54542 | 22.093887 | 74.949997 | 1 | 1 | 5 | 1 | 1 | 0 | 0 | 0 | 0 |  | 104.9546405 | 0.438351234 | 102.6934747 | 0.50210404 |  | | 2 |  | | 0 |  | 3 | 25 | 0 | 7 | 0 |  |
| 8551 | 1 | 15.576 | 5.1900001 | 33.320496 | 66.679504 | 0 | 11.501711 | 0 | 2.4412034 | 2 | 113.54542 | 20.531668 | 81.669998 | 1 | 3 | 5 | 1 | 1 | 0 | 0 | 0 | 0 |  | | NA |  | NA |  | | 2 |  | | 0 |  | 3 | 26 | 0 | 7 | 0 |  |
| 8551 | 0 | 15.913 | 5.3499999 | 33.620308 | 66.379692 | 0 | 11.578371 | 0 | 2.4412034 | 2 | 113.54542 | 19.778332 | 81.900002 | 1 | 5 | 5 | 1 | 0 | 0 | 0 | 0 | 0 |  | | NA |  | NA |  | | 2 |  | | 0 |  | 3 | 24 | 0 | 6.8000002 | 1 |  |
| 8551 | 0 | 21.148001 | 4.6999998 | 22.224321 | 77.775681 | 0 | 11.638604 | 0 | 2.4412034 | 2 | 113.54542 | 17.511667 | 86.279999 | 1 | 6 | 5 | 1 | 0 | 0 | 0 | 0 | 0 |  | 122.3170065 | 1.944999179 | 102.4792195 | 0.459570186 |  | | 2 |  | | 0 | 0 | 3 | 27 | 0 | 7 | 0 |  |
| 8551 | 0 | 20.313999 | 4.2399998 | 20.872305 | 79.127693 | 0 | 11.942505 | 0 | 2.4412034 | 2 | 113.54542 | 17.835554 | 81.690002 | 1 | 12 | 5 | 1 | 0 | 0 | 0 | 0 | 0 |  | 117.0729306 | 1.491527653 | 103.7490934 | 0.706134878 |  | | 2 |  | | 0 |  | 3 | 27 | 0 | 7 | 0 |  |
| 8553 | 1 | 19.034 | 6.461 | 33.944519 | 66.055481 | 0 | 11.293634 | 0 | 2.4412034 | 2 | 115.86508 | 16.506666 | 84.635 | 1 | 0 | 4 |  | 1 | 1 | 1 | 0 | 0 |  | 132.6132953 | 2.825121786 | 92.25222923 | -1.227644308 |  | | | | | 0 | 0 |  | | | | | |
| 8553 | 0 | 24.275 | 7.4200001 | 30.566427 | 69.433571 | 1 | 12.060233 | 0 | 2.4412034 | 2 | 115.86508 | 21.29278 | 78.169998 | 1 | 1 | 4 |  | 0 | 0 | 0 | 0 | 0 | 0 | 122.8510732 | 1.985965124 | 100.9613739 | 0.173318287 |  | | 2 |  | | 0 |  | | 21 | 0 | 6.4000001 | 1 |  |
| 8553 | 0 | 16.597 | 5.2800002 | 31.812979 | 68.187019 | 1 | 12.13963 | 0 | 2.4412034 | 2 | 115.86508 | 20.111664 | 83.260002 | 1 | 3 | 4 |  | 0 | 0 | 0 | 0 | 0 | 0 |  | NA |  | NA |  | | 2 |  | | 0 |  | | 25 | 0 | 6.4000001 | 1 |  |
| 8553 | 1 | 17.48 | 5.6700001 | 32.437073 | 67.562927 | 1 | 12.21629 | 0 | 2.4412034 | 2 | 115.86508 | 19.450001 | 83.349998 | 1 | 5 | 4 |  | 1 | 0 | 0 | 0 | 1 | 0 |  | NA |  | NA |  | | 2 |  | | 0 |  | | 20 | 0 | 6.6999998 | 1 |  |
| 8553 | 0 | 21.41 | 5.1799998 | 24.194302 | 75.805702 | 1 | 12.583162 | 0 | 2.4412034 | 2 | 115.86508 | 16.825556 | 85.809998 | 1 | 12 | 4 |  | 0 | 0 | 0 | 0 | 0 | 0 | 118.36668 | 1.596276669 | 96.12223176 | -0.646342354 |  | | 2 |  | | 0 |  | | 25 | 0 | 7 | 0 |  |
| 8554 | 1 | 17.452 | 5.372 | 30.781572 | 69.21843 | 1 | 17.475702 | 0 | 1.9938918 | 2 | 94.470978 | 19.603333 | 77.307 | 1 | 0 | 4 | 1 | 1 | 1 | 1 | 1 | 1 | 1 | 96.95603673 | -0.261722678 | 104.0485149 | 0.712347796 | 2 | 2 |  | 0 | 0 | 0 | 0 |  | | | | | |
| 8554 | 1 | 22.988001 | 7.3400002 | 31.929703 | 68.070297 | 1 | 18.064339 | 0 | 1.9938918 | 2 | 94.470978 | 21.402779 | 77.279999 | 1 | 1 | 4 | 1 | 1 | 1 | 1 | 1 | 1 | 1 | 93.3025824 | -0.57636792 | 103.6806931 | 0.639445671 | 2 | 2 | 2 | 0 | 0 | 0 |  | | 19 | 1 | 7 | 0 |  |
| 8554 | 1 | 16.032 | 5.0799999 | 31.686626 | 68.313377 | 1 | 18.140999 | 0 | 1.9938918 | 2 | 94.470978 | 20.281109 | 82.300003 | 1 | 3 | 4 | 1 | 1 | 1 | 1 | 1 | 1 | 0 |  | NA |  | NA | 2 | 2 | 2 | 0 | 0 | 0 |  | | 21 | 0 | 7 | 0 |  |
| 8554 | 1 | 18.437 | 4.9200001 | 26.685471 | 73.314529 | 1 | 18.255989 | 0 | 1.9938918 | 2 | 94.470978 | 18.076113 | 84.949997 | 1 | 6 | 4 | 1 | 1 | 1 | 1 | 1 | 1 | 1 | 95.26969892 | -0.406034905 | 101.0954843 | 0.183683929 | 2 | 2 | 2 | 0 | 0 | 0 | 0 |  | 24 | 0 | 6.8000002 | 1 |  |
| 8554 | 1 | 20.219 | 4.8299999 | 23.888422 | 76.11158 | 1 | 18.581793 | 0 | 1.9938918 | 2 | 94.470978 | 16.921112 | 83.800003 | 1 | 12 | 4 | 1 | 1 | 1 | 1 | 1 | 1 | 1 | 92.67138372 | -0.629768352 | 106.6470946 | 1.190096151 | 2 | 2 | 2 | 0 | 0 | 0 |  | | 25 | 0 | 7 | 0 |  |
| 8555 | 0 | 19.473 | 6.835 | 35.09988 | 64.900116 | 1 | 13.563313 | 1 | 2.2397017 | 3 | 105.39181 | 16.563889 | 84.139 | 1 | 0 | 3 | 1 | 0 | 0 | 0 | 0 | 0 | 0 | 117.8109491 | 1.626076584 | 94.86589578 | -0.77312156 | 2 | 2 |  | 0 | 0 | 0 | 0 | 3 |  | | | | |
| 8555 | 1 | 23.339001 | 7.4200001 | 31.792278 | 68.207726 | 1 | 14.275154 | 1 | 2.2397017 | 3 | 105.39181 | 21.50889 | 77.089996 | 1 | 1 | 3 | 1 | 0 | 0 | 0 | 0 | 1 | 0 | 97.08345993 | -0.267291872 | 95.86473912 | -0.619607339 | 2 | 2 | 2 | 0 | 0 | 0 |  | 3 | 21 | 0 | 7 | 0 |  |
| 8555 | 0 | 18.448999 | 4.96 | 26.884928 | 73.115074 | 1 | 14.469542 | 1 | 2.2397017 | 3 | 105.39181 | 18.214447 | 84.589996 | 1 | 6 | 3 | 1 | 0 | 0 | 0 | 0 | 0 | 0 | 114.3049758 | 1.304914171 | 99.10835705 | -0.13674824 | 2 | 2 | 2 | 0 | 0 | 0 | 0 | 3 | 24 | 0 | 7 | 0 |  |
| 8555 | 0 | 20.761 | 4.7800002 | 23.023939 | 76.976059 | 1 | 14.792608 | 1 | 2.2397017 | 3 | 105.39181 | 17.198334 | 82.599998 | 1 | 12 | 3 | 1 | 0 | 0 | 0 | 0 | 0 | 0 | 117.6898357 | 1.611354245 | 94.89164736 | -0.751613595 | 2 | 2 | 2 | 0 | 0 | 0 |  | 3 | 25 | 0 | 7 | 0 |  |
| 8556 | 1 | 19.488 | 6.866 | 35.231937 | 64.768059 | 0 | 10.590007 | 0 | 2.4412034 | 3 | 119.46104 | 16.711666 | 84.099 | 1 | 0 | 5 |  | 1 | 1 | 1 | 0 | 1 |  | 113.7010268 | 1.205766809 | 112.0121891 | 2.617648192 |  | | | | | 0 | 0 | 2 |  | | | | |
| 8556 | 1 | 23.587999 | 7.4699998 | 31.668646 | 68.331352 | 0 | 11.370295 | 0 | 2.4412034 | 3 | 119.46104 | 21.681112 | 76.550003 | 1 | 1 | 5 |  | 1 | 1 | 1 | 1 | 1 |  | 112.1699727 | 1.070514818 | 111.6606231 | 2.49763006 |  | | 2 |  | | 0 |  | 2 | 20 | 0 | 7 | 0 |  |
| 8556 | 1 | 16.334 | 5.21 | 31.896658 | 68.10334 | 0 | 11.446954 | 0 | 2.4412034 | 3 | 119.46104 | 20.362223 | 82.18 | 1 | 3 | 5 |  | 1 | 1 | 0 | 0 | 0 |  | | NA |  | NA |  | | 2 |  | | 0 |  | 2 | 18 | 1 | 5.8000002 | 1 |  |
| 8556 | 1 | 16.573 | 5.4699998 | 33.005489 | 66.994507 | 0 | 11.523614 | 0 | 2.4412034 | 3 | 119.46104 | 19.429445 | 83.089996 | 1 | 5 | 5 |  | 1 | 1 | 1 | 1 | 1 |  | | NA |  | NA |  | | 2 |  | | 0 |  | 2 | 14 | 1 | 5.5 | 1 |  |
| 8556 | 1 | 19.862 | 4.9499998 | 24.921961 | 75.078041 | 0 | 11.578371 | 0 | 2.4412034 | 3 | 119.46104 | 17.818333 | 85.440002 | 1 | 6 | 5 |  | 1 | 1 | 1 | 1 | 1 |  | 108.4308392 | 0.743090298 | 95.39788248 | -0.764635793 |  | | 2 |  | | 0 | 1 | 2 | 16 | 1 | 3.5 | 1 |  |
| 8556 | 1 | 20.761999 | 4.75 | 22.878336 | 77.121666 | 0 | 11.890486 | 0 | 2.4412034 | 3 | 119.46104 | 17.252777 | 83 | 1 | 12 | 5 |  | 1 | 1 | 1 | 1 | 0 |  | 86.81911108 | -1.182708708 | 105.2743191 | 1.017375581 |  | | 2 |  | | 0 |  | 2 | 7 | 1 | 7 | 0 |  |
| 8558 | 0 | 18.86 | 6.484 | 34.379639 | 65.620361 | 1 | 13.700206 | 1 | 1.9938918 | 3 | 111.45274 | 17.381666 | 82.748 | 1 | 0 | 4 | 0 | 0 | 0 | 0 | 0 | 0 | 0 | 117.0138411 | 1.553151804 | 101.7231012 | 0.274380861 | 1 | 2 |  | 0 | 0 | 1 | 0 | 3 |  | | | | |
| 8558 | 0 | 24.129 | 7.5999999 | 31.497368 | 68.502632 | 1 | 14.461328 | 1 | 1.9938918 | 3 | 111.45274 | 21.781111 | 76.269997 | 1 | 1 | 4 | 0 | 0 | 0 | 0 | 0 | 0 | 0 | 114.8966906 | 1.35872711 | 107.6670262 | 1.261253068 | 1 | 2 | 2 | 0 | 0 | 1 |  | 3 | 23 | 0 | 7 | 0 |  |
| 8558 | 0 | 16.561001 | 5.3400002 | 32.244431 | 67.755569 | 1 | 14.540726 | 1 | 1.9938918 | 3 | 111.45274 | 20.344446 | 82.370003 | 1 | 3 | 4 | 0 | 0 | 0 | 0 | 0 | 0 | 0 |  | NA |  | NA | 1 | 2 | 2 | 0 | 0 | 1 |  | 3 | 24 | 0 | 7 | 0 |  |
| 8558 | 0 | 19.455 | 4.9899998 | 25.648932 | 74.351067 | 1 | 14.652977 | 1 | 1.9938918 | 3 | 111.45274 | 18.206669 | 85 | 1 | 6 | 4 | 0 | 0 | 0 | 0 | 0 | 0 | 0 | 84.67294971 | -1.40849372 | 99.94147963 | -0.00900411 | 1 | 2 | 2 | 0 | 0 | 1 | 0 | 3 | 13 | 1 | 4.3000002 | 1 |  |
| 8558 | 0 | 21.313 | 4.7600002 | 22.333788 | 77.666214 | 1 | 14.948666 | 1 | 1.9938918 | 3 | 111.45274 | 17.64889 | 82.580002 | 1 | 12 | 4 | 0 | 0 | 0 | 0 | 0 | 0 | 0 | 94.66782637 | -0.488381681 | 110.7986943 | 1.801725806 | 1 | 2 | 2 | 0 | 0 | 1 |  | 3 | 21 | 0 | 4.8000002 | 1 |  |
| 8560 | 1 | 23.321 | 6.47 | 27.743237 | 72.256767 | 1 | 14.061602 | 1 | 1.9938918 | 3 | 83.998512 | 19.778889 | 79.967 | 1 | 0 | 5 | 1 | 1 | 1 | 1 | 1 | 1 | 1 | 111.1842067 | 1.021765014 | 80.47773375 | -2.567000804 | 2 | 2 |  | 0 |  | 0 | 1 | 1 |  | | | | |
| 8560 | 1 | 24.209999 | 7.48 | 30.896324 | 69.103676 | 1 | 14.527037 | 1 | 1.9938918 | 3 | 83.998512 | 21.444443 | 77.550003 | 1 | 1 | 5 | 1 | 1 | 0 | 1 | 1 | 1 | 0 | 114.192999 | 1.294595698 | 92.93100179 | -1.028259635 | 2 | 2 | 2 | 0 |  | 0 |  | 1 | 21 | 0 | 6.5999999 | 1 |  |
| 8560 | 1 | 16.584 | 5.3099999 | 32.018814 | 67.981186 | 1 | 14.603696 | 1 | 1.9938918 | 3 | 83.998512 | 20.193333 | 83.019997 | 1 | 3 | 5 | 1 | 1 | 1 | 1 | 1 |  | 1 |  | NA |  | NA | 2 | 2 | 1 | 0 |  | 0 |  | 1 | 16 | 1 | 4.5999999 | 1 |  |
| 8560 | 1 | 17.091999 | 5.5999999 | 32.763866 | 67.23613 | 1 | 14.680356 | 1 | 1.9938918 | 3 | 83.998512 | 19.391666 | 83.589996 | 1 | 5 | 5 | 1 | 0 | 0 | 0 | 0 | 1 | 0 |  | NA |  | NA | 2 | 2 | 2 | 0 |  | 0 |  | 1 | 24 | 0 | 7 | 0 |  |
| 8560 | 0 | 19.721001 | 5.04 | 25.556513 | 74.443489 | 1 | 14.718686 | 1 | 1.9938918 | 3 | 83.998512 | 18.034443 | 85.5 | 1 | 6 | 5 | 1 | 0 | 0 | 0 | 0 | 0 | 0 | 118.5141423 | 1.686354245 | 103.594101 | 0.568976195 | 2 | 2 | 2 | 0 |  | 0 | 0 | 1 | 14 | 1 | 4.8000002 | 1 |  |
| 8560 | 0 | 21.893999 | 4.9499998 | 22.608934 | 77.391068 | 1 | 15.011636 | 1 | 1.9938918 | 3 | 83.998512 | 17.48889 | 83.43 | 1 | 12 | 5 | 1 | 0 | 0 | 0 | 0 | 0 | 0 | 104.8524723 | 0.443223091 | 99.87213776 | -0.019522521 | 2 | 2 | 1 | 0 |  | 0 |  | 1 | 14 | 1 | 7 | 0 |  |
| 8561 | 1 | 18.816 | 6.439 | 34.220875 | 65.779121 | 0 | 10.562629 | 0 | 2.0574906 | 2 | 97.349136 | 17.442223 | 82.711 | 1 | 0 | 5 | 0 | 1 | 0 | 0 | 0 | 0 |  | 96.5541955 | -0.307716955 | 107.7084517 | 1.570003816 | 2 | 2 |  | 1 | 0 | 0 | 0 | 3 |  | | | | |
| 8561 | 1 | 24.174999 | 7.5799999 | 31.354706 | 68.645294 | 0 | 11.321013 | 0 | 2.0574906 | 2 | 97.349136 | 21.571669 | 76.970001 | 1 | 1 | 5 | 0 | 1 | 1 | 1 | 1 | 1 |  | 91.48836888 | -0.76250437 | 108.7901766 | 1.80294118 | 2 | 2 | 2 | 1 | 0 | 0 |  | 3 | 15 | 1 | 5 | 1 |  |
| 8561 | 1 | 16.874001 | 5.4499998 | 32.298206 | 67.70179 | 0 | 11.397673 | 0 | 2.0574906 | 2 | 97.349136 | 20.283335 | 82.540001 | 1 | 3 | 5 | 0 | 1 | 1 | 0 | 0 | 0 |  | | NA |  | NA | 2 | 2 | 2 | 1 | 0 | 0 |  | 3 | 22 | 0 | 6.5 | 1 |  |
| 8561 | 0 | 16.886999 | 5.5999999 | 33.161606 | 66.838394 | 0 | 11.474333 | 0 | 2.0574906 | 2 | 97.349136 | 19.409443 | 83.269997 | 1 | 5 | 5 | 0 | 0 | 0 | 0 | 0 | 0 |  | | NA |  | NA | 2 | 2 | 2 | 1 | 0 | 0 |  | 3 | 27 | 0 | 7 | 0 |  |
| 8561 | 0 | 19.802 | 5.0900002 | 25.704475 | 74.295525 | 0 | 11.515401 | 0 | 2.0574906 | 2 | 97.349136 | 17.985556 | 85.279999 | 1 | 6 | 5 | 0 | 0 | 0 | 0 | 0 | 0 |  | 94.81226636 | -0.46292968 | 99.8911875 | -0.019403941 | 2 | 2 | 2 | 1 | 0 | 0 | 0 | 3 | 17 | 1 | 6.4000001 | 1 |  |
| 8561 | 1 | 21.413 | 4.9099998 | 22.929995 | 77.070007 | 0 | 11.805613 | 0 | 2.0574906 | 2 | 97.349136 | 17.637779 | 82.730003 | 1 | 12 | 5 | 0 | 1 | 1 | 1 | 1 | 1 |  | 90.30431207 | -0.867507063 | 109.9220889 | 2.056049609 | 2 | 2 | 2 | 1 | 0 | 0 |  | 3 | 20 | 0 | 5.5999999 | 1 |  |
| 8562 | 1 | 18.958 | 6.573 | 34.671379 | 65.328621 | 1 | 15.274469 | 1 | 1.5445096 | 2 | 118.52356 | 17.281111 | 82.667 | 1 | 0 | 5 | 0 | 1 | 1 | 1 | 1 | 1 | 1 | 113.5666216 | 1.235914482 | 111.5921947 | 1.932859804 | 2 | 2 |  | 0 | 0 | 0 | 0 | 3 |  | | | | |
| 8562 | 1 | 23.056999 | 7.1999998 | 31.226959 | 68.773041 | 1 | 16.060232 | 1 | 1.5445096 | 2 | 118.52356 | 21.418333 | 77.370003 | 1 | 1 | 5 | 0 | 1 | 1 | 0 | 0 | 1 | 1 | 123.9765715 | 2.176430999 | 103.4947111 | 0.53900807 | 2 | 2 | 2 | 0 | 0 | 0 |  | 3 | 22 | 0 | 7 | 0 |  |
| 8562 | 0 | 15.756 | 5.1100001 | 32.432091 | 67.567909 | 1 | 16.123203 | 1 | 1.5445096 | 2 | 118.52356 | 20.315001 | 82.32 | 1 | 3 | 5 | 0 | 0 | 0 | 0 | 0 | 0 | 0 |  | NA |  | NA | 2 | 2 | 2 | 0 | 0 | 0 |  | 3 | 24 | 0 | 7 | 0 |  |
| 8562 | 0 | 16.379999 | 5.4000001 | 32.967037 | 67.032967 | 1 | 16.2026 | 1 | 1.5445096 | 2 | 118.52356 | 19.505556 | 82.699997 | 1 | 5 | 5 | 0 | 0 | 0 | 0 | 0 | 0 | 0 |  | NA |  | NA | 2 | 2 | 2 | 0 | 0 | 0 |  | 3 | 24 | 0 | 7 | 0 |  |
| 8562 | 0 | 19.398001 | 4.8899999 | 25.208782 | 74.791214 | 1 | 16.24367 | 1 | 1.5445096 | 2 | 118.52356 | 18.035553 | 85.099998 | 1 | 6 | 5 | 0 | 0 | 0 | 0 | 0 | 0 | 0 | 128.517032 | 2.585395369 | 100.2262073 | 0.03389876 | 2 | 2 | 2 | 0 | 0 | 0 | 0 | 3 | 21 | 0 | 6.9000001 | 1 |  |
| 8562 | 0 | 20.790001 | 4.54 | 21.837421 | 78.162582 | 1 | 16.569473 | 1 | 1.5445096 | 2 | 118.52356 | 17.57 | 81.720001 | 1 | 12 | 5 | 0 | 0 | 0 | 0 | 0 | 0 | 0 | 118.8068808 | 1.708122795 | 107.4840552 | 1.18118816 | 2 | 2 | 2 | 0 | 0 | 0 |  | 3 | 25 | 0 | 7 | 0 |  |
| 8563 | 1 | 18.373 | 5.667 | 30.844173 | 69.155823 | 1 | 16.380562 | 0 | -1.7323623 | 3 | 49.487652 | 17.706112 | 81.921 | 1 | 0 | 5 | 0 | 1 | 1 | 1 | 1 | 1 | 1 | 55.62572923 | -4.008936317 | 97.84110475 | -0.354816275 | 2 | 2 |  | 0 | 0 | 0 | 0 | 3 |  | | | | |
| 8563 | 0 | 24.587999 | 7.3800001 | 30.014643 | 69.985359 | 1 | 17.034908 | 0 | -1.7323623 | 3 | 49.487652 | 21.098886 | 79.019997 | 1 | 1 | 5 | 0 | 0 | 0 | 0 | 0 | 0 | 0 | 83.02760845 | -1.481668354 | 97.4356953 | -0.416082604 | 2 | 2 | 2 | 0 | 0 | 0 |  | 3 | 22 | 0 | 5.6999998 | 1 |  |
| 8563 | 0 | 16.455999 | 5.2199998 | 31.720953 | 68.279045 | 1 | 17.108829 | 0 | -1.7323623 | 3 | 49.487652 | 19.981113 | 84.120003 | 1 | 3 | 5 | 0 | 0 | 0 | 0 | 0 | 0 | 0 |  | NA |  | NA | 2 | 2 | 2 | 0 | 0 | 0 |  | 3 | 22 | 0 | 6 | 1 |  |
| 8563 | 0 | 17.469999 | 5.6300001 | 32.226677 | 67.773323 | 1 | 17.18549 | 0 | -1.7323623 | 3 | 49.487652 | 19.177776 | 84.879997 | 1 | 5 | 5 | 0 | 0 | 0 | 0 | 0 | 0 | 0 |  | NA |  | NA | 2 | 2 | 2 | 0 | 0 | 0 |  | 3 | 23 | 0 | 6.3000002 | 1 |  |
| 8563 | 0 | 19.944 | 5.1399999 | 25.772161 | 74.227837 | 1 | 17.22382 | 0 | -1.7323623 | 3 | 49.487652 | 18.184446 | 85.529999 | 1 | 6 | 5 | 0 | 0 | 0 | 0 | 0 | 0 | 0 | 70.09280445 | -2.644736283 | 99.74653343 | -0.042309058 | 2 | 2 | 2 | 0 | 0 | 0 | 0 | 3 | 17 | 1 | 4.8000002 | 1 |  |
| 8563 | 0 | 22.882 | 5.25 | 22.943798 | 77.056198 | 1 | 17.563313 | 0 | -1.7323623 | 3 | 49.487652 | 17.115555 | 85.230003 | 1 | 12 | 5 | 0 | 0 | 0 | 0 | 0 | 0 | 0 | 93.44281164 | -0.565389755 | 97.08808309 | -0.467507181 | 2 | 2 | 2 | 0 | 0 | 0 |  | 3 | 24 | 0 | 7 | 0 |  |
| 8564 | 1 | 19.155 | 6.429 | 33.563038 | 66.436958 | 0 | 9.8945923 | 0 | 2.3033004 | 4 | 123.66071 | 15.672778 | 86.275 | 1 | 0 | 5 | 1 | 1 | 1 | 1 | 1 | 1 |  | 137.555349 | 3.2387766 | 95.81288435 | -0.70716804 | 2 | 2 |  | 1 |  | 1 | 0 | 2 |  | | | | |
| 8564 | 1 | 24.844 | 7.4299998 | 29.906616 | 70.093384 | 0 | 10.658453 | 0 | 2.3033004 | 4 | 123.66071 | 21.112776 | 78.959999 | 1 | 1 | 5 | 1 | 1 | 1 | 0 | 0 | 0 |  | 133.743201 | 2.924711273 | 100.3212081 | 0.05810213 | 2 | 2 | 2 | 1 |  | 1 |  | 2 | 21 | 0 | 6.5999999 | 1 |  |
| 8564 | 0 | 16.497999 | 5.2199998 | 31.6402 | 68.359802 | 0 | 10.735113 | 0 | 2.3033004 | 4 | 123.66071 | 19.972776 | 84.190002 | 1 | 3 | 5 | 1 | 0 | 0 | 0 | 0 | 0 |  | | NA |  | NA | 2 | 2 | 2 | 1 |  | 1 |  | 2 | 25 | 0 | 7 | 0 |  |
| 8564 | 1 | 17.433001 | 5.5999999 | 32.122982 | 67.877014 | 0 | 10.811772 | 0 | 2.3033004 | 4 | 123.66071 | 19.162775 | 84.82 | 1 | 5 | 5 | 1 | 1 | 0 | 0 | 0 | 0 |  | | NA |  | NA | 2 | 2 | 2 | 1 |  | 1 |  | 2 | 26 | 0 | 6.9000001 | 1 |  |
| 8564 | 1 | 19.952 | 5.1199999 | 25.661587 | 74.338409 | 0 | 10.85284 | 0 | 2.3033004 | 4 | 123.66071 | 18.035553 | 85.93 | 1 | 6 | 5 | 1 | 1 | 1 | 1 | 1 | 1 |  | 133.4531002 | 2.899655645 | 93.70505625 | -1.023011227 | 2 | 2 | 2 | 1 |  | 1 | 0 | 2 | 18 | 1 | 5.3000002 | 1 |  |
| 8564 | 0 | 22.992001 | 5.2800002 | 22.96451 | 77.035492 | 0 | 11.143053 | 0 | 2.3033004 | 4 | 123.66071 | 17.084444 | 85.400002 | 1 | 12 | 5 | 1 | 0 | 0 | 0 | 0 | 0 |  | 112.5973685 | 1.108649545 | 95.84918072 | -0.696799473 | 2 | 2 | 2 | 1 |  | 1 |  | 2 | 17 | 1 | 3.8 | 1 |  |
| 8565 | 0 | 18.932 | 6.434 | 33.984787 | 66.015213 | 1 | 13.379877 | 1 | 2.2397017 | 4 | 111.78265 | 16.843889 | 83.885 | 1 | 0 | 4 | 1 | 0 | 0 | 0 | 0 | 0 | 0 | 124.6617097 | 2.248683583 | 97.34566201 | -0.409749453 | 2 | 2 |  | 0 | 0 | 0 | 0 | 2 |  | | | | |
| 8565 | 1 | 16.485001 | 5.27 | 31.968454 | 68.031548 | 1 | 14.217659 | 1 | 2.2397017 | 4 | 111.78265 | 20.199446 | 83.080002 | 1 | 3 | 4 | 1 | 1 | 0 | 1 | 1 |  | 1 |  | NA |  | NA | 2 | 2 | 2 | 0 | 0 | 0 |  | 2 | 19 | 1 | 4.1999998 | 1 |  |
| 8565 | 0 | 16.899 | 5.54 | 32.783005 | 67.216995 | 1 | 14.294319 | 1 | 2.2397017 | 4 | 111.78265 | 19.306665 | 84.029999 | 1 | 5 | 4 | 1 | 0 | 0 | 0 | 0 | 0 | 0 |  | NA |  | NA | 2 | 2 | 2 | 0 | 0 | 0 |  | 2 | 18 | 1 | 5.3000002 | 1 |  |
| 8565 | 0 | 19.502001 | 5.0599999 | 25.946054 | 74.053947 | 1 | 14.332649 | 1 | 2.2397017 | 4 | 111.78265 | 18.285002 | 84.949997 | 1 | 6 | 4 | 1 | 0 | 0 | 0 | 0 | 0 | 0 | 117.7709584 | 1.620171708 | 94.19807477 | -0.856237293 | 2 | 2 | 2 | 0 | 0 | 0 | 0 | 2 | 17 | 1 | 2.9000001 | 1 |  |
| 8565 | 0 | 21.892 | 4.9499998 | 22.610998 | 77.389 | 1 | 14.625599 | 1 | 2.2397017 | 4 | 111.78265 | 17.381666 | 83.650002 | 1 | 12 | 4 | 1 | 0 | 0 | 0 | 0 | 0 | 0 | 113.4751158 | 1.229066998 | 97.3107829 | -0.405076894 | 2 | 2 | 2 | 0 | 0 | 0 |  | 2 | 23 | 0 | 5.8000002 | 1 |  |
| 8566 | 1 | 17.033 | 5.656 | 33.206131 | 66.793869 | 0 | 11.748117 | 0 | 1.9361382 | 4 | 94.93367 | 19.240555 | 77.556 | 1 | 0 | 5 | 1 | 1 | 1 | 1 | 0 | 0 |  | 101.5433069 | 0.136728096 | 99.88337974 | -0.020746146 | 2 | 2 |  | 0 | 0 | 0 | 0 | 2 |  | | | | |
| 8566 | 1 | 23.099001 | 7.4000001 | 32.036018 | 67.963982 | 1 | 12.443532 | 0 | 1.9361382 | 4 | 94.93367 | 22.031666 | 75.089996 | 1 | 1 | 5 | 1 | 1 | 1 | 1 | 1 | 1 | 1 | 89.68221601 | -0.920027416 | 102.7499344 | 0.507166072 | 2 | 2 | 2 | 0 | 0 | 0 |  | 2 | 19 | 1 | 7 | 0 |  |
| 8566 | 1 | 15.635 | 5.0700002 | 32.427246 | 67.572754 | 1 | 12.520191 | 0 | 1.9361382 | 4 | 94.93367 | 20.492779 | 81.82 | 1 | 3 | 5 | 1 | 1 | 0 | 0 | 1 | 1 | 1 |  | NA |  | NA | 2 | 2 | 2 | 0 | 0 | 0 |  | 2 | 22 | 0 | 6.9000001 | 1 |  |
| 8566 | 1 | 15.822 | 5.2600002 | 33.24485 | 66.75515 | 1 | 12.596851 | 0 | 1.9361382 | 4 | 94.93367 | 19.612223 | 82.550003 | 1 | 5 | 5 | 1 | 1 | 0 | 1 | 1 | 1 | 0 |  | NA |  | NA | 2 | 2 | 2 | 0 | 0 | 0 |  | 2 | 25 | 0 | 7 | 0 |  |
| 8566 | 1 | 19.552 | 4.6999998 | 24.03846 | 75.96154 | 1 | 12.635181 | 0 | 1.9361382 | 4 | 94.93367 | 18.16889 | 85.089996 | 1 | 6 | 5 | 1 | 1 | 1 | 1 | 1 | 1 | 1 | 92.18867793 | -0.693981919 | 101.3470701 | 0.242733199 | 2 | 2 | 2 | 0 | 0 | 0 | 0 | 2 | 24 | 0 | 6.9000001 | 1 |  |
| 8566 | 0 | 20.423 | 4.2600002 | 20.858837 | 79.141167 | 1 | 12.966461 | 0 | 1.9361382 | 4 | 94.93367 | 17.682222 | 81.75 | 1 | 12 | 5 | 1 | 0 | 0 | 0 | 0 | 0 | 0 | 87.20251327 | -1.139842962 | 101.4825098 | 0.266655414 | 2 | 2 | 2 | 0 | 0 | 0 |  | 2 | 25 | 0 | 7 | 0 |  |
| 8569 | 0 | 19.124 | 6.156 | 32.189919 | 67.810081 | 1 | 18.031485 | 1 | 0.34508947 | 2 | 111.58488 | 19.293333 | 77.893 | 1 | 0 | 3 | 1 | 0 | 0 | 0 | 0 | 0 | 0 | 116.3733379 | 1.486016365 | 102.3910341 | 0.356547822 | 2 | 2 |  | 0 | 0 | 0 | 0 | 1 |  | | | | |
| 8569 | 1 | 23.242001 | 7.6500001 | 32.914551 | 67.085449 | 1 | 18.57358 | 1 | 0.34508947 | 2 | 111.58488 | 21.693892 | 76.099998 | 1 | 1 | 3 | 1 | 0 | 0 | 1 | 1 | 1 | 0 | 107.1551588 | 0.650577058 | 93.47596815 | -0.903384803 | 2 | 2 | 2 | 0 | 0 | 0 |  | 1 | 15 | 1 | 7 | 0 |  |
| 8569 | 0 | 17.006001 | 5.6300001 | 33.105961 | 66.894035 | 1 | 18.715948 | 1 | 0.34508947 | 2 | 111.58488 | 19.426113 | 82.410004 | 1 | 5 | 3 | 1 | 0 | 0 | 0 | 0 | 0 | 0 |  | NA |  | NA | 2 | 2 | 2 | 0 | 0 | 0 |  | 1 | 24 | 0 | 7 | 0 |  |
| 8569 | 0 | 23.047001 | 5.3899999 | 23.38699 | 76.613007 | 1 | 18.751539 | 1 | 0.34508947 | 2 | 111.58488 | 17.562777 | 85.720001 | 1 | 6 | 3 | 1 | 0 | 0 | 0 | 0 | 0 | 0 | 108.8491019 | 0.804169236 | 92.55178712 | -1.022575783 | 2 | 2 | 2 | 0 | 0 | 0 | 0 | 1 | 20 | 0 | 6.1999998 | 1 |  |
| 8571 | 1 | 17.896 | 6.422 | 35.885113 | 64.114883 | 0 | 11.427789 | 0 | 2.4412034 | 4 | 119.67194 | 18.084999 | 80.318 | 1 | 0 | 5 | 0 | 1 | 1 | 1 | 1 | 1 |  | 152.255794 | 4.463558815 | 83.806322 | -2.244209191 |  | | | | | 0 | 0 | 1 |  | | | | |
| 8571 | 1 | 23.323 | 7.5500002 | 32.371479 | 67.628517 | 1 | 12.183436 | 0 | 2.4412034 | 4 | 119.67194 | 21.651667 | 76.410004 | 1 | 1 | 5 | 0 | 1 | 0 | 1 | 0 | 1 | 0 | 141.1071244 | 3.522820589 | 85.7710721 | -2.029671439 |  | | 2 |  | | 0 |  | 1 | 23 | 0 | 7 | 0 |  |
| 8571 | 1 | 15.726 | 5.6199999 | 35.736996 | 64.263008 | 1 | 12.243669 | 0 | 2.4412034 | 4 | 119.67194 | 20.323334 | 81.889999 | 1 | 3 | 5 | 0 | 1 | 0 | 1 | 0 | 1 | 0 |  | NA |  | NA |  | | 2 |  | | 0 |  | 1 | 23 | 0 | 6.1999998 | 1 |  |
| 8571 | 1 | 16.976 | 5.5799999 | 32.869934 | 67.130066 | 1 | 12.325805 | 0 | 2.4412034 | 4 | 119.67194 | 19.493332 | 82.269997 | 1 | 5 | 5 | 0 | 1 | 1 | 1 | 0 | 1 | 0 |  | NA |  | NA |  | | 2 |  | | 0 |  | 1 | 24 | 0 | 7 | 0 |  |
| 8571 | 0 | 21.388 | 5.3400002 | 24.967272 | 75.03273 | 1 | 12.358659 | 0 | 2.4412034 | 4 | 119.67194 | 17.696112 | 85.550003 | 1 | 6 | 5 | 0 | 0 | 0 | 0 | 0 | 0 | 0 | 144.3117569 | 3.784793519 | 87.06212299 | -1.881275692 |  | | 2 |  | | 0 | 0 | 1 | 23 | 0 | 6 | 1 |  |
| 8571 | 0 | 22.531 | 5.1599998 | 22.901779 | 77.098221 | 1 | 12.695415 | 0 | 2.4412034 | 4 | 119.67194 | 18.291113 | 80.260002 | 1 | 12 | 5 | 0 | 0 | 0 | 0 | 0 | 0 | 0 | 130.0995029 | 2.59086979 | 82.58407619 | -2.361811991 |  | | 2 |  | | 0 |  | 1 | 24 | 0 | 7 | 0 |  |
| 8572 | 1 | 18.158 | 6.386 | 35.169071 | 64.830925 | 0 | 10.710472 | 1 | 2.2397017 | 4 | 132.92479 | 18.229445 | 80.42 | 1 | 0 | 5 | 1 | 1 | 0 | 1 | 1 | 1 |  | 136.2588432 | 3.279187658 | 104.0465411 | 0.683724907 | 1 | 2 |  | 0 | 0 | 1 | 0 | 2 |  | | | | |
| 8572 | 1 | 23.719999 | 7.8200002 | 32.96796 | 67.032036 | 0 | 11.455168 | 1 | 2.2397017 | 4 | 132.92479 | 21.881113 | 75.480003 | 1 | 1 | 5 | 1 | 1 | 1 | 1 | 1 | 0 |  | 116.6994471 | 1.523839662 | 108.3575242 | 1.463727299 | 1 | 2 | 2 | 0 | 0 | 1 |  | 2 | 25 | 0 | 7 | 0 |  |
| 8572 | 1 | 16.266001 | 5.5700002 | 34.243206 | 65.756798 | 0 | 11.526352 | 1 | 2.2397017 | 4 | 132.92479 | 20.399446 | 81.889999 | 1 | 3 | 5 | 1 | 1 | 1 | 1 | 1 | 0 |  | | NA |  | NA | 1 | 2 | 2 | 0 | 0 | 1 |  | 2 | 24 | 0 | 7 | 0 |  |
| 8572 | 0 | 16.891001 | 5.4899998 | 32.502514 | 67.49749 | 0 | 11.608487 | 1 | 2.2397017 | 4 | 132.92479 | 19.544445 | 82.139999 | 1 | 5 | 5 | 1 | 0 | 0 | 0 | 0 | 0 |  | | NA |  | NA | 1 | 2 | 2 | 0 | 0 | 1 |  | 2 | 25 | 0 | 7 | 0 |  |
| 8572 | 1 | 21.148001 | 5.23 | 24.730471 | 75.269531 | 0 | 11.641341 | 1 | 2.2397017 | 4 | 132.92479 | 17.723888 | 85.589996 | 1 | 6 | 5 | 1 | 1 | 0 | 1 | 1 | 1 |  | 138.1456902 | 3.466622312 | 105.8667661 | 1.002798542 | 1 | 2 | 2 | 0 | 0 | 1 | 0 | 2 | 26 | 0 | 6.9000001 | 1 |  |
| 8572 | 0 | 21.660999 | 4.9099998 | 22.667467 | 77.332535 | 0 | 11.964408 | 1 | 2.2397017 | 4 | 132.92479 | 18.12611 | 80.82 | 1 | 12 | 5 | 1 | 0 | 0 | 0 | 0 | 0 |  | 137.6990118 | 3.428915771 | 98.82720205 | -0.187498064 | 1 | 2 | 2 | 0 | 0 | 1 |  | 2 | 18 | 1 | 5.8000002 | 1 |  |
| 8574 | 0 | 19.207 | 6.804 | 35.424583 | 64.575417 | 0 | 10.444901 | 0 | -0.98386347 | 3 | 118.51118 | 20.711666 | 80.118 | 1 | 0 | 5 | 1 | 0 | 0 | 0 | 0 | 0 |  | 134.4685773 | 2.985661743 | 94.37797859 | -0.925333881 | 2 | 2 |  | 0 | 0 | 0 | 0 |  | | | | | |
| 8574 | 0 | 21.528999 | 6.4299998 | 29.866692 | 70.133308 | 0 | 11.175907 | 0 | -0.98386347 | 3 | 118.51118 | 21.18222 | 78.889999 | 1 | 1 | 5 | 1 | 0 | 0 | 0 | 0 | 0 |  | 119.0253823 | 1.665644156 | 93.86067892 | -0.998478556 | 2 | 2 | 2 | 0 | 0 | 0 |  | | 23 | 0 | 6.9000001 | 1 |  |
| 8574 | 1 | 16.584999 | 5.5700002 | 33.584568 | 66.415436 | 0 | 11.255304 | 0 | -0.98386347 | 3 | 118.51118 | 20.39889 | 80.400002 | 1 | 3 | 5 | 1 | 1 | 0 | 0 | 0 | 0 |  | | NA |  | NA | 2 | 2 | 2 | 0 | 0 | 0 |  | | 26 | 0 | 7 | 0 |  |
| 8574 | 0 | 17.629 | 5.4099998 | 30.68807 | 69.311928 | 0 | 11.331964 | 0 | -0.98386347 | 3 | 118.51118 | 19.282223 | 81.639999 | 1 | 5 | 5 | 1 | 0 | 0 | 0 | 0 | 0 |  | | NA |  | NA | 2 | 2 | 2 | 0 | 0 | 0 |  | | 27 | 0 | 7 | 0 |  |
| 8574 | 0 | 17.347 | 4.8000002 | 27.670492 | 72.329506 | 0 | 11.381246 | 0 | -0.98386347 | 3 | 118.51118 | 16.891111 | 86.370003 | 1 | 6 | 5 | 1 | 0 | 0 | 0 | 0 | 0 |  | 129.8802077 | 2.592572369 | 93.41788015 | -1.061788273 | 2 | 2 | 2 | 0 | 0 | 0 | 0 |  | 27 | 0 | 7 | 0 |  |
| 8574 | 0 | 16.981001 | 4.2800002 | 25.204639 | 74.795357 | 0 | 11.665982 | 0 | -0.98386347 | 3 | 118.51118 | 18.402224 | 81.110001 | 1 | 12 | 5 | 1 | 0 | 0 | 0 | 0 | 0 |  | 137.351822 | 3.21917227 | 94.32593696 | -0.926717247 | 2 | 2 | 2 | 0 | 0 | 0 |  | | 27 | 0 | 7 | 0 |  |
| 8578 | 1 | 18.316 | 6.39 | 34.887531 | 65.112473 | 0 | 10.965093 | 1 | -0.71556735 | 4 | 97.366501 | 18.084999 | 80.353 | 1 | 0 | 5 | 1 | 1 | 1 | 1 | 1 | 1 |  | 97.0555117 | -0.26928292 | 107.350556 | 1.280193367 | 2 | 2 |  | 0 | 0 | 0 | 0 | 3 |  | | | | |
| 8578 | 0 | 24.101 | 7.9299998 | 32.903198 | 67.096802 | 0 | 11.701574 | 1 | -0.71556735 | 4 | 97.366501 | 21.791111 | 75.769997 | 1 | 1 | 5 | 1 | 0 | 0 | 0 | 0 | 0 |  | 72.3813759 | -2.552860994 | 112.1022088 | 2.184453768 | 2 | 2 | 2 | 0 | 0 | 0 |  | 3 | 22 | 0 | 7 | 0 |  |
| 8578 | 0 | 17.278999 | 5.9499998 | 34.434864 | 65.56514 | 0 | 11.778234 | 1 | -0.71556735 | 4 | 97.366501 | 20.374447 | 81.800003 | 1 | 3 | 5 | 1 | 0 | 0 | 0 | 0 | 0 |  | | NA |  | NA | 2 | 2 | 2 | 0 | 0 | 0 |  | 3 | 22 | 0 | 6.8000002 | 1 |  |
| 8578 | 0 | 16.544001 | 5.5300002 | 33.426014 | 66.573982 | 0 | 11.854894 | 1 | -0.71556735 | 4 | 97.366501 | 19.355553 | 82.660004 | 1 | 5 | 5 | 1 | 0 | 0 | 0 | 0 | 0 |  | | NA |  | NA | 2 | 2 | 2 | 0 | 0 | 0 |  | 3 | 27 | 0 | 7 | 0 |  |
| 8578 | 0 | 18.771 | 5.3200002 | 28.341591 | 71.658409 | 0 | 11.890486 | 1 | -0.71556735 | 4 | 97.366501 | 18.033333 | 84.470001 | 1 | 6 | 5 | 1 | 0 | 0 | 0 | 0 | 0 |  | 82.36256891 | -1.625955878 | 114.3992911 | 2.643791119 | 2 | 2 | 2 | 0 | 0 | 0 | 0 | 3 | 27 | 0 | 7 | 0 |  |
| 8578 | 0 | 20.917 | 4.96 | 23.71277 | 76.287231 | 1 | 12.243669 | 1 | -0.71556735 | 4 | 97.366501 | 18.018333 | 81.360001 | 1 | 12 | 5 | 1 | 0 | 0 | 0 | 0 | 0 | 0 | 86.83316504 | -1.212889426 | 101.8777263 | 0.307439629 | 2 | 2 | 2 | 0 | 0 | 0 |  | 3 | 25 | 0 | 7 | 0 |  |
| 8579 | 0 | 16.738 | 6.131 | 36.629227 | 63.370773 | 0 | 10.992471 | 1 | 0.59089935 | 2 | 105.47066 | 19.356112 | 77.025 | 1 | 0 | 2 | 1 | 0 | 0 | 0 | 0 | 0 |  | 106.4416704 | 0.58781452 | 106.6744592 | 1.155110027 | 2 | 2 |  | 0 | 0 | 0 | 0 | 3 |  | | | | |
| 8579 | 0 | 20.239 | 5.3099999 | 26.236473 | 73.763527 | 0 | 11.82204 | 1 | 0.59089935 | 2 | 105.47066 | 17.867779 | 85.050003 | 1 | 6 | 2 | 1 | 0 | 0 | 0 | 0 | 0 |  | 102.4138429 | 0.221277278 | 105.1909796 | 0.88013302 | 2 | 2 | 2 | 0 | 0 | 0 | 0 | 3 | 27 | 0 | 7 | 0 |  |
| 8579 | 1 | 22.114 | 5.1700001 | 23.378855 | 76.621147 | 1 | 12.134154 | 1 | 0.59089935 | 2 | 105.47066 | 18.236664 | 80.510002 | 1 | 12 | 2 | 1 | 1 | 0 | 1 | 1 | 0 |  | 108.4784976 | 0.776448235 | 101.9016981 | 0.311888605 | 2 | 2 | 2 | 0 | 0 | 0 |  | 3 | 23 | 0 | 7 | 0 |  |
| 8580 | 0 | 19.194 | 6.687 | 34.839012 | 65.160988 | 1 | 14.45859 | 1 | -0.65196854 | 3 | 96.466042 | 16.915001 | 83.474 | 1 | 0 | 4 | 1 | 0 | 0 | 0 | 0 | 0 |  | 104.7997212 | 0.438862461 | 98.45223446 | -0.236149567 | 2 | 2 |  | 1 | 0 | 0 | 0 |  | | | | | |
| 8580 | 1 | 23.186001 | 7.3299999 | 31.613903 | 68.386093 | 1 | 15.244353 | 1 | -0.65196854 | 3 | 96.466042 | 21.394445 | 77.360001 | 1 | 1 | 4 | 1 | 1 | 1 | 1 | 1 | 1 | 1 | 107.3141704 | 0.667361495 | 97.19893529 | -0.416612117 | 2 | 2 | 2 | 1 | 0 | 0 |  | | 24 | 0 | 7 | 0 |  |
| 8580 | 1 | 16.164 | 5.1399999 | 31.799059 | 68.200943 | 1 | 15.315537 | 1 | -0.65196854 | 3 | 96.466042 | 20.287222 | 82.419998 | 1 | 3 | 4 | 1 | 1 | 1 | 1 | 1 | 1 | 0 |  | NA |  | NA | 2 | 2 | 2 | 1 | 0 | 0 |  | | 25 | 0 | 7 | 0 |  |
| 8580 | 1 | 19.18 | 4.9699998 | 25.912407 | 74.087593 | 1 | 15.438741 | 1 | -0.65196854 | 3 | 96.466042 | 18.041666 | 84.919998 | 1 | 6 | 4 | 1 | 1 | 0 | 0 | 0 | 0 | 0 | 106.0267869 | 0.549914073 | 98.89814502 | -0.165579464 | 2 | 2 | 2 | 1 | 0 | 0 | 0 |  | 24 | 0 | 7 | 0 |  |
| 8580 | 0 | 20.962 | 4.6399999 | 22.135292 | 77.864708 | 1 | 15.764544 | 1 | -0.65196854 | 3 | 96.466042 | 17.541668 | 81.610001 | 1 | 12 | 4 | 1 | 0 | 0 | 0 | 0 | 0 | 0 | 107.9721824 | 0.726734672 | 91.09573391 | -1.248036061 | 2 | 2 | 2 | 1 | 0 | 0 |  | | 21 | 0 | 6.5999999 | 1 |  |
| 8581 | 1 | 17.645 | 5.498 | 31.158968 | 68.841034 | 1 | 12.394251 | 1 | 0.95540243 | 2 | 98.413857 | 20.996666 | 75.771 | 1 | 0 | 5 | 1 | 1 | 1 | 1 | 1 | 0 | 1 | 113.6817087 | 1.251597752 | 92.55706951 | -1.11846543 | 2 | 2 |  | 0 | 0 | 1 | 0 | 3 |  | | | | |
| 8581 | 0 | 23.333 | 7.3800001 | 31.629025 | 68.370979 | 1 | 12.99384 | 1 | 0.95540243 | 2 | 98.413857 | 21.507221 | 77.050003 | 1 | 1 | 5 | 1 | 0 | 0 | 0 | 0 | 0 | 0 | 104.698174 | 0.430608722 | 94.32004589 | -0.859722057 | 2 | 2 | 2 | 0 | 0 | 1 |  | 3 | 24 | 0 | 7 | 0 |  |
| 8581 | 0 | 16.476 | 5.3200002 | 32.289391 | 67.710609 | 1 | 13.070499 | 1 | 0.95540243 | 2 | 98.413857 | 20.396666 | 81.989998 | 1 | 3 | 5 | 1 | 0 | 0 | 0 | 0 | 0 | 0 |  | NA |  | NA | 2 | 2 | 2 | 0 | 0 | 1 |  | 3 | 25 | 0 | 7 | 0 |  |
| 8581 | 0 | 16.766001 | 5.4000001 | 32.208038 | 67.791962 | 1 | 13.14716 | 1 | 0.95540243 | 2 | 98.413857 | 19.678331 | 81.610001 | 1 | 5 | 5 | 1 | 0 | 0 | 0 | 0 | 0 | 0 |  | NA |  | NA | 2 | 2 | 2 | 0 | 0 | 1 |  | 3 | 24 | 0 | 7 | 0 |  |
| 8581 | 1 | 19.268 | 5.0999999 | 26.468756 | 73.531242 | 1 | 13.18549 | 1 | 0.95540243 | 2 | 98.413857 | 17.969444 | 85.120003 | 1 | 6 | 5 | 1 | 1 | 1 | 0 | 0 | 1 | 0 | 108.6872778 | 0.795276061 | 92.70650214 | -1.08488876 | 2 | 2 | 2 | 0 | 0 | 1 | 0 | 3 | 23 | 0 | 6.6999998 | 1 |  |
| 8581 | 0 | 20.306999 | 4.54 | 22.356823 | 77.643173 | 1 | 13.511293 | 1 | 0.95540243 | 2 | 98.413857 | 17.680555 | 81.269997 | 1 | 12 | 5 | 1 | 0 | 0 | 0 | 0 | 0 | 0 | 106.562537 | 0.600822104 | 91.95727713 | -1.181899051 | 2 | 2 | 2 | 0 | 0 | 1 |  | 3 | 24 | 0 | 7 | 0 |  |
| 8586 | 0 | 18.847 | 6.591 | 34.971085 | 65.028915 | 0 | 11.633128 | 1 | 2.4412034 | 3 | 111.92393 | 19.345556 | 80.759 | 1 | 0 | 5 | 1 | 0 | 0 | 0 | 0 | 0 |  | 111.4403489 | 1.045874992 | 107.5780001 | 1.315393261 | 2 | 2 |  | 0 | 0 | 0 | 0 | 4 |  | | | | |
| 8586 | 0 | 23.832001 | 7.4400001 | 31.218529 | 68.781471 | 1 | 12.410678 | 1 | 2.4412034 | 3 | 111.92393 | 21.665556 | 76.800003 | 1 | 1 | 5 | 1 | 0 | 0 | 0 | 0 | 0 | 0 | 117.1177041 | 1.564661102 | 105.171176 | 0.869201695 | 2 | 2 | 2 | 0 | 0 | 0 |  | 4 | 24 | 0 | 7 | 0 |  |
| 8586 | 0 | 16.416 | 5.5100002 | 33.564816 | 66.435181 | 1 | 12.479124 | 1 | 2.4412034 | 3 | 111.92393 | 20.501112 | 81.919998 | 1 | 3 | 5 | 1 | 0 | 0 | 0 | 0 | 0 | 0 |  | NA |  | NA | 2 | 2 | 2 | 0 | 0 | 0 |  | 4 | 27 | 0 | 7 | 0 |  |
| 8586 | 0 | 16.799999 | 5.54 | 32.976192 | 67.023811 | 1 | 12.555783 | 1 | 2.4412034 | 3 | 111.92393 | 19.693888 | 82.040001 | 1 | 5 | 5 | 1 | 0 | 0 | 0 | 0 | 0 | 0 |  | NA |  | NA | 2 | 2 | 2 | 0 | 0 | 0 |  | 4 | 24 | 0 | 7 | 0 |  |
| 8586 | 0 | 19.722 | 5.1700001 | 26.21438 | 73.785622 | 1 | 12.599589 | 1 | 2.4412034 | 3 | 111.92393 | 18.087778 | 84.93 | 1 | 6 | 5 | 1 | 0 | 0 | 0 | 0 | 0 | 0 | 115.4454584 | 1.412316929 | 106.8249459 | 1.159799803 | 2 | 2 | 2 | 0 | 0 | 0 | 0 | 4 | 24 | 0 | 7 | 0 |  |
| 8586 | 0 | 19.91 | 4.5100002 | 22.651936 | 77.348068 | 1 | 12.887064 | 1 | 2.4412034 | 3 | 111.92393 | 17.808889 | 81.889999 | 1 | 12 | 5 | 1 | 0 | 0 | 0 | 0 | 0 | 0 | 117.7820523 | 1.62479323 | 107.6871938 | 1.308602033 | 2 | 2 | 2 | 0 | 0 | 0 |  | 4 | 24 | 0 | 7 | 0 |  |
| 8587 | 0 | 18.997 | 6.653 | 35.02132 | 64.978683 | 0 | 10.781656 | 1 | 0.11822473 | 3 | 121.26395 | 19.532778 | 80.492 | 1 | 0 | 5 | 1 | 0 | 0 | 0 | 0 | 0 |  | 127.2915093 | 2.474435314 | 102.0872779 | 0.346190996 | 2 | 2 |  | 0 | 0 | 0 | 0 | 4 |  | | | | |
| 8587 | 1 | 22.573999 | 6.9200001 | 30.654737 | 69.345261 | 0 | 11.575633 | 1 | 0.11822473 | 3 | 121.26395 | 21.251665 | 78.360001 | 1 | 1 | 5 | 1 | 1 | 0 | 1 | 1 | 0 |  | 116.2184674 | 1.480751238 | 104.6894823 | 0.793670006 | 2 | 2 | 2 | 0 | 0 | 0 |  | 4 | 19 | 1 | 7 | 0 |  |
| 8587 | 0 | 16.42 | 5.54 | 33.739342 | 66.260658 | 0 | 11.649555 | 1 | 0.11822473 | 3 | 121.26395 | 20.403332 | 81.199997 | 1 | 3 | 5 | 1 | 0 | 0 | 0 | 0 | 0 |  | | NA |  | NA | 2 | 2 | 2 | 0 | 0 | 0 |  | 4 | 25 | 0 | 7 | 0 |  |
| 8587 | 0 | 18.087 | 5.29 | 29.247526 | 70.752472 | 0 | 11.728952 | 1 | 0.11822473 | 3 | 121.26395 | 19.57 | 80.940002 | 1 | 5 | 5 | 1 | 0 | 0 | 0 | 0 | 0 |  | | NA |  | NA | 2 | 2 | 2 | 0 | 0 | 0 |  | 4 | 23 | 0 | 6.9000001 | 1 |  |
| 8587 | 0 | 19.466999 | 5.1300001 | 26.352291 | 73.647713 | 0 | 11.764544 | 1 | 0.11822473 | 3 | 121.26395 | 17.217222 | 86.110001 | 1 | 6 | 5 | 1 | 0 | 0 | 0 | 0 | 0 |  | 127.4653568 | 2.502606732 | 104.1846818 | 0.703647365 | 2 | 2 | 1 | 0 | 0 | 0 | 0 | 4 | 20 | 0 | 4.6999998 | 1 |  |
| 8587 | 0 | 17.965 | 4.3699999 | 24.325075 | 75.674927 | 1 | 12.07666 | 1 | 0.11822473 | 3 | 121.26395 | 18.255001 | 80.699997 | 1 | 12 | 5 | 1 | 0 | 0 | 0 | 0 | 0 | 0 | 128.3420402 | 2.583427868 | 102.0148666 | 0.331039174 | 2 | 2 | 2 | 0 | 0 | 0 |  | 4 | 25 | 0 | 7 | 0 |  |
| 8588 | 1 | 18.244 | 6.157 | 33.748081 | 66.251915 | 1 | 12.128679 | 1 | 1.4156679 | 4 | 94.024963 | 21.623333 | 78.16 | 1 | 0 | 3 | 1 | 0 | 0 | 1 | 0 | 0 | 0 | 104.0708528 | 0.373209459 | 98.78251733 | -0.194189085 | 2 | 2 |  | 0 | 0 | 0 | 0 | 4 |  | | | | |
| 8588 | 0 | 21.492001 | 6.5500002 | 30.476456 | 69.523544 | 1 | 12.895277 | 1 | 1.4156679 | 4 | 94.024963 | 21.283333 | 78.290001 | 1 | 1 | 3 | 1 | 0 | 0 | 0 | 0 | 0 | 0 | 106.5791972 | 0.602773757 | 99.9345647 | -0.010421502 | 2 | 2 | 2 | 0 | 0 | 0 |  | 4 | 25 | 0 | 7 | 0 |  |
| 8588 | 0 | 16.094 | 5.52 | 34.298496 | 65.7015 | 1 | 12.969199 | 1 | 1.4156679 | 4 | 94.024963 | 20.48111 | 80.660004 | 1 | 3 | 3 | 1 | 0 | 0 | 0 | 0 | 0 | 0 |  | NA |  | NA | 2 | 2 | 2 | 0 | 0 | 0 |  | 4 | 25 | 0 | 7 | 0 |  |
| 8588 | 0 | 18.768999 | 4.4200001 | 23.549471 | 76.450531 | 1 | 13.393566 | 1 | 1.4156679 | 4 | 94.024963 | 17.908335 | 81.970001 | 1 | 12 | 3 | 1 | 0 | 0 | 0 | 0 | 0 | 0 | 102.0708668 | 0.189841243 | 95.00756879 | -0.755029362 | 2 | 2 | 2 | 0 | 0 | 0 |  | 4 | 24 | 0 | 7 | 0 |  |
| 8589 | 1 | 19.158 | 6.692 | 34.930576 | 65.06942 | 1 | 12.150581 | 1 | 1.6710101 | 3 | 110.72977 | 19.797777 | 79.746 | 1 | 0 | 4 | 1 | 1 | 1 | 1 | 1 | 1 | 0 | 134.938622 | 3.18050998 | 87.68756429 | -1.773173992 | 2 | 2 |  | 0 | 0 | 1 | 0 | 2 |  | | | | |
| 8589 | 1 | 23.632 | 7.0300002 | 29.747801 | 70.252197 | 1 | 12.944558 | 1 | 1.6710101 | 3 | 110.72977 | 21.315554 | 78.309998 | 1 | 1 | 4 | 1 | 1 | 0 | 0 | 0 | 1 | 0 | 126.5746695 | 2.423266655 | 86.38897784 | -1.919791505 | 2 | 2 | 2 | 0 | 0 | 1 |  | 2 | 21 | 0 | 7 | 0 |  |
| 8589 | 1 | 17.216999 | 5.9299998 | 34.442703 | 65.557297 | 1 | 13.021218 | 1 | 1.6710101 | 3 | 110.72977 | 20.498335 | 80.330002 | 1 | 3 | 4 | 1 | 1 | 1 | 1 | 1 | 1 | 0 |  | NA |  | NA | 2 | 2 | 2 | 0 | 0 | 1 |  | 2 | 24 | 0 | 7 | 0 |  |
| 8589 | 0 | 21.818001 | 5.5599999 | 25.483543 | 74.516457 | 1 | 13.133471 | 1 | 1.6710101 | 3 | 110.72977 | 17.273333 | 85.360001 | 1 | 6 | 4 | 1 | 0 | 0 | 0 | 0 | 0 | 0 | 122.1388482 | 2.020401255 | 83.11364083 | -2.305527204 | 2 | 2 | 2 | 0 | 0 | 1 | 0 | 2 | 23 | 0 | 5.8000002 | 1 |  |
| 8589 | 0 | 17.603001 | 4.77 | 27.097652 | 72.902351 | 1 | 13.445585 | 1 | 1.6710101 | 3 | 110.72977 | 18.342777 | 79.860001 | 1 | 12 | 4 | 1 | 0 | 0 | 0 | 0 | 0 | 0 | 132.2250579 | 2.933206851 | 95.8259949 | -0.635158713 | 2 | 2 | 2 | 0 | 0 | 1 |  | 2 | 20 | 0 | 6.1999998 | 1 |  |
| 8591 | 1 | 17.862 | 5.861 | 32.812675 | 67.187325 | 0 | 11.964408 | 1 | 2.4412034 | 4 | 109.31512 | 21.633333 | 77.654 | 1 | 0 | 3 |  | 1 | 1 | 1 | 1 | 1 |  | 116.5645316 | 1.513658246 | 99.79457892 | -0.033129987 |  | | | | | 0 | 0 | 3 |  | | | | |
| 8591 | 0 | 22.355 | 6.52 | 29.165735 | 70.834267 | 1 | 12.706366 | 1 | 2.4412034 | 4 | 109.31512 | 21.070002 | 79.68 | 1 | 1 | 3 |  | 0 | 0 | 0 | 0 | 0 | 0 | 123.86355 | 2.177735826 | 99.83156429 | -0.026890039 |  | | 2 |  | | 0 |  | 3 | 25 | 0 | 7 | 0 |  |
| 8591 | 0 | 16.721001 | 5.5799999 | 33.371208 | 66.628792 | 1 | 12.783026 | 1 | 2.4412034 | 4 | 109.31512 | 20.402777 | 80.769997 | 1 | 3 | 3 |  | 0 | 0 | 0 | 0 | 0 | 0 |  | NA |  | NA |  | | 2 |  | | 0 |  | 3 | 24 | 0 | 7 | 0 |  |
| 8591 | 0 | 17.271 | 4.2600002 | 24.665627 | 75.334373 | 1 | 13.22382 | 1 | 2.4412034 | 4 | 109.31512 | 18.402224 | 81.25 | 1 | 12 | 3 |  | 0 | 0 | 0 | 0 | 0 | 0 | 123.4742842 | 2.141426228 | 82.75217773 | -2.344294884 |  | | 2 |  | | 0 |  | 3 | 25 | 0 | 7 | 0 |  |
| 8593 | 1 | 15.312 | 6.362 | 41.54911 | 58.45089 | 1 | 16.273785 | 0 | 1.0243496 | 3 | 92.183952 | 18.143333 | 78.255 | 1 | 0 | 3 | 1 | 1 | 0 | 0 | 1 | 1 | 1 | 121.9606523 | 1.859810676 | 83.0104286 | -2.28100943 | 2 | 2 |  | 0 | 0 | 1 | 1 | 1 |  | | | | |
| 8593 | 1 | 23.441999 | 8.5 | 36.259705 | 63.740295 | 1 | 16.960985 | 0 | 1.0243496 | 3 | 92.183952 | 22.166668 | 73.830002 | 1 | 1 | 3 | 1 | 1 | 0 | 1 | 1 | 1 | 1 | 124.3293795 | 2.049962568 | 91.52871573 | -1.27118511 | 2 | 2 | 2 | 0 | 0 | 1 |  | 1 | 18 | 1 | 7 | 0 |  |
| 8593 | 0 | 20.384001 | 5.5900002 | 27.42347 | 72.57653 | 1 | 17.149897 | 0 | 1.0243496 | 3 | 92.183952 | 18.508335 | 84.169998 | 1 | 6 | 3 | 1 |  | | 0 | 0 | 0 |  | 119.0182081 | 1.607650157 | 87.38166691 | -1.787756844 | 2 | 2 | 2 | 0 | 0 | 1 | 0 | 1 | 22 | 0 | 6.5999999 | 1 |  |
| 8593 | 1 | 25.334999 | 5.77 | 22.774818 | 77.225182 | 1 | 17.470226 | 0 | 1.0243496 | 3 | 92.183952 | 18.585001 | 79.080002 | 1 | 12 | 3 | 1 | 1 | 0 | 0 | 0 | 1 | 0 | 131.7419397 | 2.6537884 | 88.73230158 | -1.621244751 | 2 | 2 | 2 | 0 | 0 | 1 |  | 1 | 22 | 0 | 5.6999998 | 1 |  |
| 8594 | 0 | 18.927 | 5.578 | 29.471127 | 70.528877 | 1 | 18.061602 | 0 | 1.2820363 | 2 | 89.350571 | 22.313334 | 74.5 | 1 | 0 | 5 | 1 | 0 | 0 | 0 | 0 | 0 | 0 | 100.8048034 | 0.068794261 | 91.9381167 | -1.203696773 | 2 | 2 |  | 0 | 0 | 0 | 0 |  | | | | | |
| 8594 | 0 | 22.721001 | 6.9299998 | 30.500416 | 69.49958 | 1 | 18.652977 | 0 | 1.2820363 | 2 | 89.350571 | 21.143333 | 78.720001 | 1 | 1 | 5 | 1 | 0 | 0 | 0 | 0 | 0 | 0 | 86.49867529 | -1.166722267 | 102.7550569 | 0.469360792 | 2 | 2 | 2 | 0 | 0 | 0 |  | | 24 | 0 | 7 | 0 |  |
| 8594 | 0 | 16.922001 | 5.5799999 | 32.974823 | 67.025177 | 1 | 18.735113 | 0 | 1.2820363 | 2 | 89.350571 | 20.333332 | 80.959999 | 1 | 3 | 5 | 1 | 0 | 0 | 0 | 0 | 0 | 0 |  | NA |  | NA | 2 | 2 | 2 | 0 | 0 | 0 |  | | 24 | 0 | 7 | 0 |  |
| 8594 | 0 | 18.732 | 5.3600001 | 28.614138 | 71.385864 | 1 | 18.811773 | 0 | 1.2820363 | 2 | 89.350571 | 19.345554 | 81.529999 | 1 | 5 | 5 | 1 | 0 | 0 | 0 | 0 | 0 | 0 |  | NA |  | NA | 2 | 2 | 2 | 0 | 0 | 0 |  | | 24 | 0 | 7 | 0 |  |
| 8594 | 0 | 20.187 | 5.1399999 | 25.461931 | 74.538071 | 1 | 18.841888 | 0 | 1.2820363 | 2 | 89.350571 | 17.019444 | 86.010002 | 1 | 6 | 5 | 1 | 0 | 0 | 0 | 0 | 0 | 0 | 93.88557126 | -0.524335692 | 98.59972148 | -0.225857448 | 2 | 2 | 2 | 0 | 0 | 0 | 0 |  | 24 | 0 | 7 | 0 |  |
| 8594 | 0 | 18.862 | 4.54 | 24.069557 | 75.930443 | 1 | 19.148529 | 0 | 1.2820363 | 2 | 89.350571 | 18.096664 | 80.339996 | 1 | 12 | 5 | 1 | 0 | 0 | 0 | 0 | 0 | 0 | 89.8781812 | -0.870296871 | 100.887675 | 0.146794467 | 2 | 2 | 2 | 0 | 0 | 0 |  | | 23 | 0 | 7 | 0 |  |
| 8599 | 0 | 16.931 | 5.674 | 33.512493 | 66.487511 | 1 | 12.156057 | 1 | -1.2354536 | 2 | 97.275085 | 19.164444 | 78.655 | 1 | 0 | 3 | 1 | 0 | 0 | 0 | 0 | 0 | 0 | 104.8989459 | 0.449049885 | 99.24923585 | -0.120210196 | 2 | 2 |  | 0 | 0 | 0 | 0 | 4 |  | | | | |
| 8599 | 1 | 22.315001 | 6.9000001 | 30.920904 | 69.079094 | 1 | 12.865161 | 1 | -1.2354536 | 2 | 97.275085 | 20.91 | 79.199997 | 1 | 1 | 3 | 1 | 1 | 1 | 1 | 1 | 1 | 0 | 95.10902845 | -0.449441089 | 100.6495393 | 0.104148959 | 2 | 2 | 2 | 0 | 0 | 0 |  | 4 | 21 | 0 | 7 | 0 |  |
| 8599 | 1 | 16.075001 | 5.1500001 | 32.037323 | 67.962677 | 1 | 12.941821 | 1 | -1.2354536 | 2 | 97.275085 | 20.15889 | 81.809998 | 1 | 3 | 3 | 1 | 1 | 1 | 1 | 1 | 1 | 0 |  | NA |  | NA | 2 | 2 | 2 | 0 | 0 | 0 |  | 4 | 24 | 0 | 7 | 0 |  |
| 8599 | 1 | 19.742001 | 4.5799999 | 23.199268 | 76.800728 | 1 | 13.368925 | 1 | -1.2354536 | 2 | 97.275085 | 17.809444 | 80.169998 | 1 | 12 | 3 | 1 | 1 | 1 | 1 | 1 | 1 | 1 | 99.23399801 | -0.070275061 | 102.5181697 | 0.406388713 | 2 | 2 | 2 | 0 | 0 | 0 |  | 4 | 25 | 0 | 7 | 0 |  |
| 8600 | 0 | 16.946 | 5.676 | 33.494629 | 66.505371 | 0 | 11.561944 | 0 | 2.2397017 | 3 | 103.03148 | 19.195 | 78.638 | 1 | 0 | 4 | 0 | 0 | 0 | 0 | 0 | 0 |  | 111.2215837 | 0.987011877 | 97.27094784 | -0.467019458 | 2 | 2 |  | 0 | 0 | 0 | 0 | 4 |  | | | | |
| 8600 | 0 | 21.039 | 6.46 | 30.704882 | 69.29512 | 1 | 12.284737 | 0 | 2.2397017 | 3 | 103.03148 | 20.773333 | 80.089996 | 1 | 1 | 4 | 0 | 0 | 0 | 0 | 0 | 0 | 0 | 106.3094543 | 0.554875241 | 105.5296223 | 1.06536806 | 2 | 2 | 2 | 0 | 0 | 0 |  | 4 | 25 | 0 | 7 | 0 |  |
| 8600 | 0 | 15.926 | 5.1199999 | 32.148685 | 67.851311 | 1 | 12.347707 | 0 | 2.2397017 | 3 | 103.03148 | 20.131666 | 81.489998 | 1 | 3 | 4 | 0 | 0 | 0 | 0 | 0 | 0 | 0 |  | NA |  | NA | 2 | 2 | 2 | 0 | 0 | 0 |  | 4 | 25 | 0 | 7 | 0 |  |
| 8600 | 1 | 20.52 | 5.1999998 | 25.341129 | 74.658867 | 1 | 12.473648 | 0 | 2.2397017 | 3 | 103.03148 | 16.974445 | 86.489998 | 1 | 6 | 4 | 0 | 1 | 0 | 0 | 0 | 1 | 1 | 111.9352843 | 1.043031754 | 102.3377194 | 0.428045036 | 2 | 2 | 2 | 0 | 0 | 0 | 0 | 4 | 21 | 0 | 7 | 0 |  |
| 8600 | 0 | 19.169001 | 4.5500002 | 23.73624 | 76.263756 | 1 | 12.783026 | 0 | 2.2397017 | 3 | 103.03148 | 17.956667 | 80.019997 | 1 | 12 | 4 | 0 | 0 | 0 | 0 | 0 | 0 | 0 | 90.57642565 | -0.837644665 | 104.6334846 | 0.875152265 | 2 | 2 | 2 | 0 | 0 | 0 |  | 4 | 24 | 0 | 7 | 0 |  |
| 8601 | 0 | 17.641 | 5.885 | 33.359787 | 66.640213 | 1 | 15.578371 | 0 | 2.3033004 | 4 | 106.37358 | 20.684999 | 76.133 | 1 | 0 | 3 | 0 | 0 | 0 | 0 | 0 | 0 | 0 | 120.2896657 | 1.726728518 | 94.74614016 | -0.834691859 | 2 | 2 |  | 1 | 0 | 0 | 0 |  | | | | | |
| 8601 | 0 | 22.584999 | 7 | 30.994024 | 69.005974 | 1 | 16.287474 | 0 | 2.3033004 | 4 | 106.37358 | 21.110556 | 78.709999 | 1 | 1 | 3 | 0 | 0 | 0 | 0 | 0 | 0 | 0 | 102.5430489 | 0.218784468 | 97.00774116 | -0.486760336 | 2 | 2 | 2 | 1 | 0 | 0 |  | | 24 | 0 | 7 | 0 |  |
| 8601 | 0 | 17.547001 | 5.75 | 32.769131 | 67.230865 | 1 | 16.364134 | 0 | 2.3033004 | 4 | 106.37358 | 20.383335 | 81.230003 | 1 | 3 | 3 | 0 | 0 | 0 | 0 | 0 | 0 | 0 |  | NA |  | NA | 2 | 2 | 2 | 1 | 0 | 0 |  | | 24 | 0 | 7 | 0 |  |
| 8601 | 1 | 18.136999 | 4.52 | 24.921432 | 75.078568 | 1 | 16.796715 | 0 | 2.3033004 | 4 | 106.37358 | 18.288332 | 79.389999 | 1 | 12 | 3 | 0 | 1 | 0 | 0 | 0 | 1 | 0 | 101.1980569 | 0.102943002 | 90.13492509 | -1.45488077 | 2 | 2 | 2 | 1 | 0 | 0 |  | | 24 | 0 | 6.9000001 | 1 |  |
| 8602 | 1 | 18.044 | 6.071 | 33.645535 | 66.354469 | 1 | 14.182067 | 0 | 2.4412034 | 3 | 118.03062 | 20.055 | 78.416 | 1 | 0 | 4 |  | 1 | 1 | 1 | 1 | 1 | 1 | 122.5261888 | 1.930161978 | 103.2196693 | 0.585166896 |  | | | | | 0 | 0 | 3 |  | | | | |
| 8602 | 1 | 21.454 | 6.5700002 | 30.623661 | 69.376343 | 1 | 14.94319 | 0 | 2.4412034 | 3 | 118.03062 | 21.043333 | 79.389999 | 1 | 1 | 4 |  | 0 | 0 | 1 | 1 | 1 | 0 | 88.3681686 | -1.021230983 | 101.7407754 | 0.306937649 |  | | 2 |  | | 0 |  | 3 | 25 | 0 | 7 | 0 |  |
| 8602 | 1 | 16.59 | 5.52 | 33.273056 | 66.726944 | 1 | 15.008898 | 0 | 2.4412034 | 3 | 118.03062 | 20.338333 | 80.93 | 1 | 3 | 4 |  | 0 | 0 | 1 | 1 | 1 | 0 |  | NA |  | NA |  | | 2 |  | | 0 |  | 3 | 25 | 0 | 7 | 0 |  |
| 8602 | 1 | 20.007999 | 5.1599998 | 25.789684 | 74.210312 | 1 | 15.126626 | 0 | 2.4412034 | 3 | 118.03062 | 17.069443 | 86.080002 | 1 | 6 | 4 |  | 1 | 1 | 1 | 1 | 1 | 0 | 89.20922506 | -0.945591654 | 101.8334013 | 0.322952124 |  | | 2 |  | | 0 | 1 | 3 | 22 | 0 | 6.9000001 | 1 |  |
| 8602 | 1 | 18.193001 | 4.4299998 | 24.350021 | 75.649979 | 1 | 15.433265 | 0 | 2.4412034 | 3 | 118.03062 | 18.231667 | 80.230003 | 1 | 12 | 4 |  | 1 | 1 | 1 | 1 | 0 | 1 | 94.56800549 | -0.472774115 | 102.0786762 | 0.365994422 |  | | 2 |  | | 0 |  | 3 | 25 | 0 | 7 | 0 |  |
| 8603 | 1 | 18.94 | 6.641 | 35.063358 | 64.936646 | 0 | 11.605749 | 0 | 2.4412034 | 4 | 99.572838 | 20.168333 | 80.304 | 1 | 0 | 5 | 1 | 0 | 0 | 1 | 0 | 0 |  | 108.9442312 | 0.788003753 | 97.57352099 | -0.417041591 | 2 | 2 |  | 0 | 0 | 0 | 1 | 3 |  | | | | |
| 8603 | 1 | 21.393999 | 6.48 | 30.288868 | 69.711136 | 1 | 12.410678 | 0 | 2.4412034 | 4 | 99.572838 | 21.171667 | 79.110001 | 1 | 1 | 5 | 1 | 1 | 1 | 1 | 0 | 1 | 1 | 131.814472 | 2.740351635 | 92.33005628 | -1.208567461 | 2 | 2 | 2 | 0 | 0 | 0 |  | 3 | 25 | 0 | 7 | 0 |  |
| 8603 | 1 | 16.069 | 5.5599999 | 34.600784 | 65.399216 | 1 | 12.476386 | 0 | 2.4412034 | 4 | 99.572838 | 20.407223 | 80.709999 | 1 | 3 | 5 | 1 | 1 | 1 | 1 | 0 | 1 | 1 |  | NA |  | NA | 2 | 2 | 2 | 0 | 0 | 0 |  | 3 | 25 | 0 | 6.9000001 | 1 |  |
| 8603 | 1 | 17.927 | 5.3099999 | 29.620125 | 70.379875 | 1 | 12.553046 | 0 | 2.4412034 | 4 | 99.572838 | 19.595003 | 81.010002 | 1 | 5 | 5 | 1 | 1 | 0 | 1 | 1 | 1 | 0 |  | NA |  | NA | 2 | 2 | 2 | 0 | 0 | 0 |  | 3 | 24 | 0 | 7 | 0 |  |
| 8603 | 1 | 18.76 | 5.0599999 | 26.972281 | 73.027718 | 1 | 12.596851 | 0 | 2.4412034 | 4 | 99.572838 | 17.130554 | 86.309998 | 1 | 6 | 5 | 1 | 1 | 1 | 1 | 1 | 1 | 1 | 134.9091043 | 2.995280736 | 94.06349628 | -0.958632606 | 2 | 2 | 2 | 0 | 0 | 0 | 0 | 3 | 23 | 0 | 6.6999998 | 1 |  |
| 8603 | 1 | 17.593 | 4.3099999 | 24.498379 | 75.501617 | 1 | 12.908966 | 0 | 2.4412034 | 4 | 99.572838 | 18.279999 | 81.290001 | 1 | 12 | 5 | 1 | 1 | 0 | 0 | 0 | 1 | 0 | 112.9688322 | 1.129499169 | 101.113898 | 0.199391482 | 2 | 2 | 2 | 0 | 0 | 0 |  | 3 | 24 | 0 | 7 | 0 |  |
| 8604 | 1 | 18.697 | 6.497 | 34.74889 | 65.251106 | 1 | 13.352498 | 0 | 2.2397017 | 4 | 126.54912 | 19.030556 | 80.644 | 1 | 0 | 4 | 1 | 1 | 1 | 1 | 0 | 0 | 1 | 146.2602823 | 3.919285482 | 92.74281918 | -1.142585177 | 2 | 2 |  | 0 | 0 | 1 | 0 | 3 |  | | | | |
| 8604 | 0 | 23.965 | 7.6300001 | 31.838099 | 68.161903 | 1 | 14.116359 | 0 | 2.2397017 | 4 | 126.54912 | 21.948891 | 75.699997 | 1 | 1 | 4 | 1 | 0 | 0 | 0 | 0 | 0 | 0 | 139.9940299 | 3.385104867 | 97.20765232 | -0.466062271 | 2 | 2 | 2 | 0 | 0 | 1 |  | 3 | 24 | 0 | 7 | 0 |  |
| 8604 | 0 | 16.445999 | 5.4200001 | 32.956345 | 67.043655 | 1 | 14.193019 | 0 | 2.2397017 | 4 | 126.54912 | 20.549999 | 81.699997 | 1 | 3 | 4 | 1 | 0 | 0 | 0 | 0 | 0 | 0 |  | NA |  | NA | 2 | 2 | 2 | 0 | 0 | 1 |  | 3 | 24 | 0 | 7 | 0 |  |
| 8604 | 0 | 16.5 | 5.48 | 33.21212 | 66.78788 | 1 | 14.269678 | 0 | 2.2397017 | 4 | 126.54912 | 19.680002 | 82.160004 | 1 | 5 | 4 | 1 | 0 | 0 | 0 | 0 | 0 | 0 |  | NA |  | NA | 2 | 2 | 2 | 0 | 0 | 1 |  | 3 | 23 | 0 | 7 | 0 |  |
| 8604 | 1 | 20.112 | 4.4400001 | 22.076372 | 77.92363 | 1 | 14.598221 | 0 | 2.2397017 | 4 | 126.54912 | 17.790001 | 81.849998 | 1 | 12 | 4 | 1 | 0 | 0 | 0 | 0 | 1 | 0 | 122.5260152 | 1.924874554 | 95.11282205 | -0.787853234 | 2 | 2 | 2 | 0 | 0 | 1 |  | 3 | 24 | 0 | 7 | 0 |  |
| 8608 | 0 | 17.321 | 5.529 | 31.92079 | 68.079208 | 1 | 16.643394 | 0 | -0.65196854 | 2 | 99.867645 | 24.572222 | 74.021 | 1 | 0 | 4 | 0 | 0 | 0 | 0 | 0 | 0 | 0 | 114.0351687 | 1.193904065 | 90.31438349 | -1.433833195 | 2 | 2 |  | 0 | 0 | 0 | 0 | 3 |  | | | | |
| 8608 | 0 | 20.805 | 6.3800001 | 30.665707 | 69.334297 | 1 | 17.316906 | 0 | -0.65196854 | 2 | 99.867645 | 21.429998 | 77.589996 | 1 | 1 | 4 | 0 | 0 | 0 | 0 | 0 | 0 | 0 | 121.5611934 | 1.817673678 | 82.10825871 | -2.357659727 | 2 | 2 | 2 | 0 | 0 | 0 |  | 3 | 25 | 0 | 7 | 0 |  |
| 8608 | 0 | 15.749 | 5.4000001 | 34.287891 | 65.712105 | 1 | 17.396303 | 0 | -0.65196854 | 2 | 99.867645 | 20.524445 | 80.389999 | 1 | 3 | 4 | 0 | 0 | 0 | 0 | 0 | 0 | 0 |  | NA |  | NA | 2 | 2 | 2 | 0 | 0 | 0 |  | 3 | 25 | 0 | 7 | 0 |  |
| 8608 | 0 | 13.617 | 4.5 | 33.046928 | 66.953072 | 1 | 17.514032 | 0 | -0.65196854 | 2 | 99.867645 | 17.075001 | 86.410004 | 1 | 6 | 4 | 0 | 0 | 0 | 0 | 0 | 0 | 0 | 127.2017218 | 2.281352851 | 83.54249088 | -2.208087788 | 2 | 2 | 2 | 0 | 0 | 0 | 0 | 3 | 23 | 0 | 6.4000001 | 1 |  |
| 8608 | 0 | 15.637 | 4.1199999 | 26.347765 | 73.652237 | 1 | 17.815195 | 0 | -0.65196854 | 2 | 99.867645 | 18.448889 | 81.860001 | 1 | 12 | 4 | 0 | 0 | 0 | 0 | 0 | 0 | 0 | 117.07406 | 1.441316201 | 85.83511102 | -1.955521455 | 2 | 2 | 2 | 0 | 0 | 0 |  | 3 | 25 | 0 | 7 | 0 |  |
| 8611 | 1 | 17.483 | 5.51 | 31.516331 | 68.483673 | 0 | 10.187543 | 1 | 0.59089935 | 2 | 97.775703 | 24.891111 | 73.538 | 1 | 0 | 4 | 1 | 1 | 0 | 0 | 0 | 0 |  | 104.743792 | 0.429695294 | 99.91517182 | -0.013749034 | 1 | 2 |  | 0 | 0 | 1 | 0 | 3 |  | | | | |
| 8611 | 0 | 20.683001 | 6.29 | 30.411448 | 69.588554 | 0 | 10.844627 | 1 | 0.59089935 | 2 | 97.775703 | 21.368332 | 77.889999 | 1 | 1 | 4 | 1 | 0 | 0 | 0 | 0 | 0 |  | 106.1795853 | 0.56331693 | 102.4341475 | 0.405024472 | 1 | 2 | 2 | 0 | 0 | 1 |  | 3 | 18 | 1 | 7 | 0 |  |
| 8611 | 0 | 15.698 | 5.4099998 | 34.462986 | 65.53701 | 0 | 10.924025 | 1 | 0.59089935 | 2 | 97.775703 | 20.504442 | 80.209999 | 1 | 3 | 4 | 1 | 0 | 0 | 0 | 0 | 0 |  | | NA |  | NA | 1 | 2 | 2 | 0 | 0 | 1 |  | 3 | 22 | 0 | 7 | 0 |  |
| 8611 | 1 | 15.067 | 4.9699998 | 32.985996 | 67.014008 | 0 | 11.033539 | 1 | 0.59089935 | 2 | 97.775703 | 18.312777 | 83.620003 | 1 | 6 | 4 | 1 | 1 | 1 | 1 | 1 | 1 |  | 105.8424131 | 0.533346002 | 100.6768439 | 0.110747876 | 1 | 2 | 1 | 0 | 0 | 1 | 0 | 3 | 22 | 0 | 6.6999998 | 1 |  |
| 8611 | 0 | 15.668 | 4.1300001 | 26.359459 | 73.640541 | 0 | 11.342916 | 1 | 0.59089935 | 2 | 97.775703 | 18.449444 | 81.830002 | 1 | 12 | 4 | 1 | 0 | 0 | 0 | 0 | 0 |  | 115.1342868 | 1.38082669 | 98.95345666 | -0.168314618 | 1 | 2 | 2 | 0 | 0 | 1 |  | 3 | 27 | 0 | 7 | 0 |  |
| 8612 | 1 | 17.704 | 5.787 | 32.687527 | 67.312469 | 1 | 14.551677 | 0 | 0.5818758 | 2 | 104.4204 | 23.360001 | 76.469 | 1 | 0 | 3 | 1 | 1 | 1 | 1 | 1 | 0 | 1 | 109.6093878 | 0.829844891 | 102.1123255 | 0.376204606 | 2 | 2 |  | 1 | 0 | 0 | 0 | 3 |  | | | | |
| 8612 | 0 | 20.575001 | 6.25 | 30.376669 | 69.623329 | 1 | 15.293634 | 0 | 0.5818758 | 2 | 104.4204 | 21.383886 | 77.839996 | 1 | 1 | 3 | 1 | 0 | 0 | 0 | 0 | 0 | 0 | 106.3827764 | 0.550228865 | 102.2404046 | 0.396037858 | 2 | 2 | 2 | 1 | 0 | 0 |  | 3 | 20 | 0 | 7 | 0 |  |
| 8612 | 1 | 14.47 | 4.8899999 | 33.794056 | 66.20594 | 1 | 15.482546 | 0 | 0.5818758 | 2 | 104.4204 | 18.255556 | 83.709999 | 1 | 6 | 3 | 1 | 1 | 1 | 1 | 1 | 1 | 1 | 107.3632471 | 0.63353119 | 103.7670938 | 0.67835674 | 2 | 2 | 2 | 1 | 0 | 0 | 0 | 3 | 22 | 0 | 4.1999998 | 1 |  |
| 8612 | 1 | 15.644 | 4.0999999 | 26.20813 | 73.79187 | 1 | 15.802875 | 0 | 0.5818758 | 2 | 104.4204 | 18.398333 | 82.239998 | 1 | 12 | 3 | 1 | 1 | 1 | 1 | 1 | 1 | 1 | 98.55791579 | -0.124811527 | 101.3535171 | 0.234879016 | 2 | 2 | 2 | 1 | 0 | 0 |  | 3 | 24 | 0 | 7 | 0 |  |
| 8614 | 1 | 18.095 | 5.705 | 31.528046 | 68.471954 | 0 | 11.288158 | 0 | 0.34508947 | 2 | 111.00523 | 22.519444 | 74.235 | 1 | 0 | 5 | 0 | 1 | 1 | 1 | 0 | 0 |  | 113.9409533 | 1.224960641 | 101.9082481 | 0.351911595 | 2 | 2 |  | 0 | 0 | 0 | 0 | 2 |  | | | | |
| 8614 | 0 | 22.704 | 6.9099998 | 30.435165 | 69.564835 | 0 | 11.928816 | 0 | 0.34508947 | 2 | 111.00523 | 21.177223 | 78.669998 | 1 | 1 | 5 | 0 | 0 | 0 | 0 | 0 | 0 |  | 117.0505529 | 1.489715659 | 103.8659642 | 0.729561981 | 2 | 2 | 2 | 0 | 0 | 0 |  | 2 | 25 | 0 | 6.9000001 | 1 |  |
| 8614 | 0 | 17.138 | 5.6399999 | 32.909325 | 67.090675 | 1 | 12.013689 | 0 | 0.34508947 | 2 | 111.00523 | 20.353334 | 80.800003 | 1 | 3 | 5 | 0 | 0 | 0 | 0 | 0 | 0 | 0 |  | NA |  | NA | 2 | 2 | 2 | 0 | 0 | 0 |  | 2 | 24 | 0 | 6.9000001 | 1 |  |
| 8614 | 0 | 19.011 | 5.4499998 | 28.667612 | 71.33239 | 1 | 12.090349 | 0 | 0.34508947 | 2 | 111.00523 | 19.286108 | 81.599998 | 1 | 5 | 5 | 0 | 0 | 0 | 0 | 0 | 0 | 0 |  | NA |  | NA | 2 | 2 | 2 | 0 | 0 | 0 |  | 2 | 24 | 0 | 7 | 0 |  |
| 8614 | 1 | 20.93 | 5.29 | 25.274725 | 74.725273 | 1 | 12.13963 | 0 | 0.34508947 | 2 | 111.00523 | 16.866667 | 86.32 | 1 | 6 | 5 | 0 | 1 | 1 | 1 | 1 | 1 | 0 | 114.8980468 | 1.302180857 | 108.7916924 | 1.782276702 | 2 | 2 | 2 | 0 | 0 | 0 | 0 | 2 | 24 | 0 | 7 | 0 |  |
| 8614 | 0 | 18.129 | 4.4699998 | 24.656626 | 75.343376 | 1 | 12.424367 | 0 | 0.34508947 | 2 | 111.00523 | 18.281666 | 80.199997 | 1 | 12 | 5 | 0 | 0 | 0 | 0 | 0 | 0 | 0 | 114.7162244 | 1.284087487 | 109.1667768 | 1.860879683 | 2 | 2 | 2 | 0 | 0 | 0 |  | 2 | 24 | 0 | 7 | 0 |  |
| 8615 | 1 | 17.665 | 5.83 | 33.003113 | 66.996887 | 1 | 13.631759 | 1 | -2.3150556 | 2 | 111.21327 | 21.525555 | 76.393 | 1 | 0 | 5 | 1 | 1 | 1 | 1 | 1 | 0 | 1 | 109.5052287 | 0.869386013 | 109.2283183 | 1.566068403 | 2 | 2 |  | 1 |  | 1 | 0 | 3 |  | | | | |
| 8615 | 0 | 22.575001 | 6.8499999 | 30.343298 | 69.6567 | 1 | 14.349076 | 1 | -2.3150556 | 2 | 111.21327 | 21.198332 | 78.629997 | 1 | 1 | 5 | 1 | 0 | 0 | 0 | 0 | 0 | 0 | 115.9404603 | 1.45386348 | 111.2188831 | 1.90344541 | 2 | 2 | 2 | 1 |  | 1 |  | 3 | 24 | 0 | 7 | 0 |  |
| 8615 | 0 | 17.193001 | 5.6599998 | 32.920372 | 67.079628 | 1 | 14.433949 | 1 | -2.3150556 | 2 | 111.21327 | 20.372776 | 80.709999 | 1 | 3 | 5 | 1 | 0 | 0 | 0 | 0 | 0 | 0 |  | NA |  | NA | 2 | 2 | 2 | 1 |  | 1 |  | 3 | 24 | 0 | 7 | 0 |  |
| 8615 | 0 | 18.48 | 5.5700002 | 30.140694 | 69.859306 | 1 | 14.51061 | 1 | -2.3150556 | 2 | 111.21327 | 19.298334 | 82.559998 | 1 | 5 | 5 | 1 | 0 | 0 | 0 | 0 | 0 | 0 |  | NA |  | NA | 2 | 2 | 2 | 1 |  | 1 |  | 3 | 24 | 0 | 7 | 0 |  |
| 8615 | 1 | 20.532 | 5.27 | 25.667252 | 74.332748 | 1 | 14.568104 | 1 | -2.3150556 | 2 | 111.21327 | 16.868887 | 86.349998 | 1 | 6 | 5 | 1 | 1 | 0 | 1 | 1 | 1 | 0 | 114.9123724 | 1.359861705 | 109.1918325 | 1.527019707 | 2 | 2 | 2 | 1 |  | 1 | 0 | 3 | 24 | 0 | 7 | 0 |  |
| 8615 | 0 | 18.285 | 4.4699998 | 24.446266 | 75.553734 | 1 | 14.850102 | 1 | -2.3150556 | 2 | 111.21327 | 18.231667 | 80.43 | 1 | 12 | 5 | 1 | 0 | 0 | 0 | 0 | 0 | 0 | 111.1770176 | 1.01958548 | 109.535819 | 1.578667615 | 2 | 2 | 2 | 1 |  | 1 |  | 3 | 19 | 1 | 6.0999999 | 1 |  |
| 8616 | 1 | 17.525 | 5.749 | 32.804565 | 67.195435 | 1 | 13.585216 | 0 | -3.3649483 | 4 | 116.36588 | 22.240555 | 75.82 | 1 | 0 | 5 | 1 | 1 | 1 | 1 | 1 | 1 | 1 | 127.6467795 | 2.369342237 | 98.98866527 | -0.174160166 | 2 | 2 |  | 0 | 0 | 0 | 0 | 3 |  | | | | |
| 8616 | 0 | 23.07 | 7.2600002 | 31.469442 | 68.530556 | 1 | 14.275154 | 0 | -3.3649483 | 4 | 116.36588 | 21.571114 | 77.019997 | 1 | 1 | 5 | 1 | 0 | 0 | 0 | 0 | 0 | 0 | 129.8338532 | 2.540734403 | 95.09157189 | -0.793435092 | 2 | 2 | 2 | 0 | 0 | 0 |  | 3 | 16 | 1 | 6.3000002 | 1 |  |
| 8616 | 0 | 17.370001 | 5.6399999 | 32.469772 | 67.530228 | 1 | 14.351814 | 0 | -3.3649483 | 4 | 116.36588 | 20.575558 | 81.449997 | 1 | 3 | 5 | 1 | 0 | 0 | 0 | 0 | 0 | 0 |  | NA |  | NA | 2 | 2 | 2 | 0 | 0 | 0 |  | 3 | 21 | 0 | 7 | 0 |  |
| 8616 | 0 | 17.33 | 5.3800001 | 31.044432 | 68.955566 | 1 | 14.428473 | 0 | -3.3649483 | 4 | 116.36588 | 19.876112 | 80.519997 | 1 | 5 | 5 | 1 | 0 | 0 | 0 | 0 | 0 | 0 |  | NA |  | NA | 2 | 2 | 2 | 0 | 0 | 0 |  | 3 | 23 | 0 | 7 | 0 |  |
| 8616 | 1 | 18.385 | 5.4099998 | 29.426161 | 70.573837 | 1 | 14.466804 | 0 | -3.3649483 | 4 | 116.36588 | 18.139444 | 84.540001 | 1 | 6 | 5 | 1 | 1 | 1 | 1 | 0 | 1 | 0 | 127.0398838 | 2.304600655 | 99.03882141 | -0.164068234 | 2 | 2 | 2 | 0 | 0 | 0 | 0 | 3 | 19 | 1 | 6.9000001 | 1 |  |
| 8616 | 0 | 17.697001 | 4.3699999 | 24.693449 | 75.306549 | 1 | 14.792608 | 0 | -3.3649483 | 4 | 116.36588 | 18.232224 | 80.93 | 1 | 12 | 5 | 1 | 0 | 0 | 0 | 0 | 0 | 0 | 139.1796656 | 3.30352862 | 95.85071524 | -0.674627796 | 2 | 2 | 2 | 0 | 0 | 0 |  | 3 | 18 | 1 | 5.0999999 | 1 |  |
| 8617 | 1 | 17.957 | 5.884 | 32.767166 | 67.232834 | 0 | 10.970568 | 0 | -1.5718457 | 2 | 102.05005 | 21.405001 | 77.596 | 1 | 0 | 5 |  | 1 | 1 | 1 | 0 | 0 |  | 111.8202441 | 1.041440789 | 96.47254392 | -0.598745896 | 2 | 2 |  | 1 | 0 | 0 | 0 | 3 |  | | | | |
| 8617 | 0 | 22.826 | 6.5300002 | 28.607729 | 71.392273 | 0 | 11.712525 | 0 | -1.5718457 | 2 | 102.05005 | 20.941111 | 80.410004 | 1 | 1 | 5 |  | 0 | 0 | 0 | 0 | 0 |  | 120.7282539 | 1.808058853 | 86.85786354 | -1.909630152 | 2 | 2 | 2 | 1 | 0 | 0 |  | 3 | 27 | 0 | 7 | 0 |  |
| 8617 | 0 | 17.562 | 5.6799998 | 32.342556 | 67.65744 | 0 | 11.789186 | 0 | -1.5718457 | 2 | 102.05005 | 20.375002 | 80.68 | 1 | 3 | 5 |  | 0 | 0 | 0 | 0 | 0 |  | | NA |  | NA | 2 | 2 | 2 | 1 | 0 | 0 |  | 3 | 27 | 0 | 7 | 0 |  |
| 8617 | 1 | 17.514999 | 5.25 | 29.97431 | 70.025688 | 0 | 11.865846 | 0 | -1.5718457 | 2 | 102.05005 | 19.435556 | 81.620003 | 1 | 5 | 5 |  | 1 | 0 | 0 | 0 | 0 |  | | NA |  | NA | 2 | 2 | 2 | 1 | 0 | 0 |  | 3 | 22 | 0 | 7 | 0 |  |
| 8617 | 1 | 20.125999 | 5.1599998 | 25.638477 | 74.361519 | 0 | 11.901438 | 0 | -1.5718457 | 2 | 102.05005 | 17.091665 | 85.809998 | 1 | 6 | 5 |  | 1 | 0 | 0 | 0 | 0 |  | 102.688242 | 0.23771469 | 90.13526622 | -1.507340979 | 2 | 2 | 2 | 1 | 0 | 0 | 0 | 3 | 24 | 0 | 7 | 0 |  |
| 8617 | 0 | 17.983 | 4.3200002 | 24.02269 | 75.97731 | 1 | 12.210814 | 0 | -1.5718457 | 2 | 102.05005 | 18.424444 | 80.699997 | 1 | 12 | 5 |  | 0 | 0 | 0 | 0 | 0 | 0 | 81.75060137 | -1.642510025 | 93.90930516 | -0.984579176 | 2 | 2 | 2 | 1 | 0 | 0 |  | 3 | 25 | 0 | 7 | 0 |  |
| 8618 | 1 | 17.89 | 5.856 | 32.733372 | 67.266632 | 0 | 9.7467489 | 0 | 2.4412034 | 2 | 109.4411 | 20.790556 | 78.591 | 1 | 0 | 4 |  | 1 | 1 | 1 | 0 | 0 |  | 114.4481862 | 1.26658462 | 101.9135594 | 0.357728945 |  | | | | | 0 | 0 | 1 |  | | | | |
| 8618 | 1 | 21.941999 | 6.23 | 28.393038 | 71.606964 | 0 | 10.499658 | 0 | 2.4412034 | 2 | 109.4411 | 20.809444 | 81.269997 | 1 | 1 | 4 |  | 1 | 1 | 1 | 1 | 1 |  | 117.107526 | 1.501477666 | 102.1023728 | 0.391828094 |  | | 2 |  | | 0 |  | 1 | 25 | 0 | 7 | 0 |  |
| 8618 | 0 | 16.813 | 5.54 | 32.950691 | 67.049309 | 0 | 10.570842 | 0 | 2.4412034 | 2 | 109.4411 | 20.28389 | 81.040001 | 1 | 3 | 4 |  | 0 | 0 | 0 | 0 | 0 |  | | NA |  | NA |  | | 2 |  | | 0 |  | 1 | 25 | 0 | 7 | 0 |  |
| 8618 | 1 | 18.681 | 5.1900001 | 27.78224 | 72.217758 | 0 | 10.71321 | 0 | 2.4412034 | 2 | 109.4411 | 17.813335 | 84.919998 | 1 | 6 | 4 |  | 1 | 1 | 1 | 0 | 1 |  | 100.9206185 | 0.081894652 | 87.82618315 | -1.800725859 |  | | 2 |  | | 0 | 0 | 1 | 9 | 1 | 5 | 1 |  |
| 8618 | 1 | 17.660999 | 4.2600002 | 24.120947 | 75.879051 | 0 | 10.997947 | 0 | 2.4412034 | 2 | 109.4411 | 18.401667 | 81.190002 | 1 | 12 | 4 |  | 1 | 1 | 1 | 1 | 1 |  | 109.8944131 | 0.873101511 | 100.0967969 | 0.017398446 |  | | 2 |  | | 0 |  | 1 | 27 | 0 | 7 | 0 |  |
| 8620 | 0 | 17.561 | 5.765 | 32.828426 | 67.17157 | 1 | 12.788501 | 1 | 0.59089935 | 3 | 96.878723 | 22.107779 | 75.909 | 1 | 0 | 4 | 1 | 0 | 0 | 0 | 0 | 0 | 0 | 101.1420742 | 0.104787565 | 104.2432459 | 0.702819659 | 2 | 2 |  | 0 | 0 | 0 | 0 |  | | | | | |
| 8620 | 0 | 21.836 | 6.5799999 | 30.133724 | 69.86628 | 1 | 13.50308 | 1 | 0.59089935 | 3 | 96.878723 | 21.142221 | 78.980003 | 1 | 1 | 4 | 1 | 0 | 0 | 0 | 0 | 0 | 0 | 99.89497438 | -0.009631912 | 104.0501531 | 0.660306581 | 2 | 2 | 2 | 0 | 0 | 0 |  | | 24 | 0 | 7 | 0 |  |
| 8620 | 0 | 16.841999 | 5.6199999 | 33.368961 | 66.631042 | 1 | 13.582478 | 1 | 0.59089935 | 3 | 96.878723 | 20.38389 | 80.5 | 1 | 3 | 4 | 1 | 0 | 0 | 0 | 0 | 0 | 0 |  | NA |  | NA | 2 | 2 | 2 | 0 | 0 | 0 |  | | 24 | 0 | 7 | 0 |  |
| 8620 | 0 | 18.438999 | 5.4699998 | 29.665384 | 70.334618 | 1 | 13.659138 | 1 | 0.59089935 | 3 | 96.878723 | 19.31889 | 81.519997 | 1 | 5 | 4 | 1 | 0 | 0 | 0 | 0 | 0 | 0 |  | NA |  | NA | 2 | 2 | 2 | 0 | 0 | 0 |  | | 24 | 0 | 7 | 0 |  |
| 8620 | 0 | 19.018 | 5.0599999 | 26.606373 | 73.393631 | 1 | 13.711157 | 1 | 0.59089935 | 3 | 96.878723 | 16.881668 | 86.349998 | 1 | 6 | 4 | 1 | 0 | 0 | 0 | 0 | 0 | 0 | 78.43167945 | -1.989134435 | 105.6407946 | 0.927826824 | 2 | 2 | 2 | 0 | 0 | 0 | 1 |  | 24 | 0 | 7 | 0 |  |
| 8622 |  | 18.158 | 5.547 | 30.548519 | 69.451485 | 1 | 12.763861 | 1 | 2.3033004 | 2 | 111.63896 | 23.63611 | 73.695 | 1 | 0 | 5 | 1 |  | | | | | | 106.5394554 | 0.599201323 | 112.1468522 | 2.152241508 | 2 | 2 |  | 0 | 0 | 0 | 0 | 4 |  | | | | |
| 8622 | 0 | 24.983 | 6.98 | 27.938999 | 72.061005 | 1 | 13.42642 | 1 | 2.3033004 | 2 | 111.63896 | 21.552221 | 77.220001 | 1 | 1 | 5 | 1 | 0 | 0 | 0 | 0 | 0 | 0 | 99.3894306 | -0.056008615 | 113.1824123 | 2.321098998 | 2 | 2 | 2 | 0 | 0 | 0 |  | 4 | 24 | 0 | 7 | 0 |  |
| 8622 | 0 | 17.049999 | 5.3800001 | 31.554255 | 68.445747 | 1 | 13.500342 | 1 | 2.3033004 | 2 | 111.63896 | 20.606113 | 80.660004 | 1 | 3 | 5 | 1 | 0 | 0 | 0 | 0 | 0 | 0 |  | NA |  | NA | 2 | 2 | 2 | 0 | 0 | 0 |  | 4 | 24 | 0 | 7 | 0 |  |
| 8622 | 0 | 32.581001 | 4.9499998 | 15.192903 | 84.807098 | 1 | 13.590692 | 1 | 2.3033004 | 2 | 111.63896 | 19.668333 | 80.800003 | 1 | 5 | 5 | 1 | 0 | 0 | 0 | 0 | 0 | 0 |  | NA |  | NA | 2 | 2 | 2 | 0 | 0 | 0 |  | 4 | 23 | 0 | 6.8000002 | 1 |  |
| 8622 | 0 | 19.885 | 5.3499999 | 26.904701 | 73.095299 | 1 | 13.61807 | 1 | 2.3033004 | 2 | 111.63896 | 17.235554 | 85.110001 | 1 | 6 | 5 | 1 | 0 | 0 | 0 | 0 | 0 | 0 | 107.4836904 | 0.684847293 | 113.3189453 | 2.337297383 | 2 | 2 | 2 | 0 | 0 | 0 | 0 | 4 | 25 | 0 | 6.9000001 | 1 |  |
| 8622 | 0 | 17.451 | 4.3299999 | 24.81233 | 75.187668 | 1 | 13.946612 | 1 | 2.3033004 | 2 | 111.63896 | 17.9 | 81.230003 | 1 | 12 | 5 | 1 | 0 | 0 | 0 | 0 | 0 | 0 | 107.5426521 | 0.689831579 | 111.7892242 | 2.027988401 | 2 | 2 | 2 | 0 | 0 | 0 |  | 4 | 17 | 1 | 6 | 1 |  |
| 8623 | 1 | 18.452 | 5.953 | 32.262085 | 67.737915 | 1 | 15.066393 | 1 | 2.3033004 | 4 | 104.29568 | 20.884445 | 77.4 | 1 | 0 | 5 | 1 | 1 | 1 | 1 | 1 | 1 | 1 | 128.8292129 | 2.618250048 | 87.15474983 | -1.761502454 | 2 | 2 |  | 0 | 0 | 0 | 0 | 1 |  | | | | |
| 8623 | 0 | 24.059999 | 6.8699999 | 28.553617 | 71.446381 | 1 | 15.808351 | 1 | 2.3033004 | 4 | 104.29568 | 21.735556 | 75.599998 | 1 | 1 | 5 | 1 | 0 | 0 | 0 | 0 | 0 | 0 | 124.0887674 | 2.187447612 | 80.24774972 | -2.522757789 | 2 | 2 | 2 | 0 | 0 | 0 |  | 1 | 21 | 0 | 6.5 | 1 |  |
| 8623 | 0 | 18.416 | 5.2600002 | 28.56212 | 71.437881 | 1 | 15.882273 | 1 | 2.3033004 | 4 | 104.29568 | 20.48222 | 80.57 | 1 | 3 | 5 | 1 | 0 | 0 | 0 | 0 | 0 | 0 |  | NA |  | NA | 2 | 2 | 2 | 0 | 0 | 0 |  | 1 | 22 | 0 | 7 | 0 |  |
| 8623 | 1 | 16.266001 | 5.2600002 | 32.337391 | 67.662613 | 1 | 15.958932 | 1 | 2.3033004 | 4 | 104.29568 | 19.718891 | 80.489998 | 1 | 5 | 5 | 1 | 1 | 1 | 1 | 0 | 1 | 0 |  | NA |  | NA | 2 | 2 | 2 | 0 | 0 | 0 |  | 1 | 23 | 0 | 6.9000001 | 1 |  |
| 8623 | 1 | 17.863001 | 5.0900002 | 28.494654 | 71.505348 | 1 | 15.991786 | 1 | 2.3033004 | 4 | 104.29568 | 17.973886 | 84.139999 | 1 | 6 | 5 | 1 | 1 | 0 | 0 | 0 | 1 | 0 | 126.5014464 | 2.404490654 | 84.23593244 | -2.078259125 | 2 | 2 | 2 | 0 | 0 | 0 | 0 | 1 | 23 | 0 | 6.8000002 | 1 |  |
| 8623 | 1 | 18.658001 | 4.3400002 | 23.260799 | 76.739197 | 1 | 16.284737 | 1 | 2.3033004 | 4 | 104.29568 | 18.483332 | 80.029999 | 1 | 12 | 5 | 1 | 1 | 0 | 1 | 1 | 1 | 1 | 134.6620303 | 3.138208811 | 83.03960399 | -2.20464034 | 2 | 2 | 2 | 0 | 0 | 0 |  | 1 | 22 | 0 | 6.5999999 | 1 |  |
| 8624 | 0 | 18.367 | 6.15 | 33.483967 | 66.516037 | 0 | 11.789186 | 1 | 2.4412034 | 2 | 106.26424 | 19.605556 | 78.784 | 1 | 0 | 5 | 0 | 0 | 0 | 0 | 0 | 0 |  | 127.0224903 | 2.462608577 | 89.54070926 | -1.536493305 |  | | | | | 0 | 0 | 2 |  | | | | |
| 8624 | 1 | 24.134001 | 7.4699998 | 30.952181 | 69.047821 | 1 | 12.522929 | 1 | 2.4412034 | 2 | 106.26424 | 21.77722 | 75.75 | 1 | 1 | 5 | 0 | 1 | 0 | 1 | 1 | 1 | 0 | 115.2544545 | 1.394969627 | 96.80277635 | -0.498376279 |  | | 1 |  | | 0 |  | 2 | 17 | 1 | 5.6999998 | 1 |  |
| 8624 | 1 | 18.125 | 5.6199999 | 31.006895 | 68.993103 | 1 | 12.599589 | 1 | 2.4412034 | 2 | 106.26424 | 20.464443 | 81.169998 | 1 | 3 | 5 | 0 | 1 | 0 | 1 | 1 | 1 | 0 |  | NA |  | NA |  | | 1 |  | | 0 |  | 2 | 17 | 1 | 4.9000001 | 1 |  |
| 8624 | 0 | 16.766001 | 5.4099998 | 32.267681 | 67.732315 | 1 | 12.678987 | 1 | 2.4412034 | 2 | 106.26424 | 19.59 | 81.32 | 1 | 5 | 5 | 0 | 0 | 0 | 0 | 0 | 0 | 0 |  | NA |  | NA |  | | 2 |  | | 0 |  | 2 | 24 | 0 | 7 | 0 |  |
| 8624 | 1 | 19.108 | 5.23 | 27.370735 | 72.629265 | 1 | 12.714579 | 1 | 2.4412034 | 2 | 106.26424 | 17.860003 | 84.690002 | 1 | 6 | 5 | 0 | 1 | 1 | 1 | 0 | 1 | 0 | 113.6939453 | 1.252611061 | 94.00317207 | -0.909096759 |  | | 2 |  | | 0 | 0 | 2 | 24 | 0 | 7 | 0 |  |
| 8624 | 0 | 19.544001 | 4.6500001 | 23.792467 | 76.207535 | 1 | 13.037645 | 1 | 2.4412034 | 2 | 106.26424 | 18.108889 | 80.93 | 1 | 12 | 5 | 0 | 0 | 0 | 0 | 0 | 0 | 0 | 114.7399721 | 1.347619579 | 93.19747972 | -1.018711678 |  | | 2 |  | | 0 |  | 2 | 24 | 0 | 7 | 0 |  |
| 8627 | 1 | 18.469 | 6.009 | 32.535599 | 67.464401 | 1 | 12.473648 | 0 | -0.46975747 | 2 | 127.18694 | 20.516111 | 77.819 | 1 | 0 | 5 | 1 | 1 | 1 | 1 | 1 | 1 |  | 136.2681431 | 3.112728661 | 101.1430121 | 0.205708415 | 2 | 2 |  | 0 | 0 | 0 | 0 | 3 |  | | | | |
| 8627 | 1 | 24.062 | 6.9699998 | 28.966835 | 71.033165 | 1 | 13.215606 | 0 | -0.46975747 | 2 | 127.18694 | 21.705 | 75.800003 | 1 | 1 | 5 | 1 | 1 | 0 | 0 | 0 | 1 | 0 | 139.4491397 | 3.361130529 | 96.61999898 | -0.56426717 | 2 | 2 | 2 | 0 | 0 | 0 |  | 3 | 18 | 1 | 5.6999998 | 1 |  |
| 8627 | 1 | 18.250999 | 5.3699999 | 29.423046 | 70.57695 | 1 | 13.289528 | 0 | -0.46975747 | 2 | 127.18694 | 20.462778 | 80.75 | 1 | 3 | 5 | 1 | 1 | 0 | 0 | 0 | 1 | 0 |  | NA |  | NA | 2 | 2 | 2 | 0 | 0 | 0 |  | 3 | 23 | 0 | 7 | 0 |  |
| 8627 | 1 | 16.358 | 5.2800002 | 32.277786 | 67.722214 | 1 | 13.366187 | 0 | -0.46975747 | 2 | 127.18694 | 19.678331 | 80.720001 | 1 | 5 | 5 | 1 | 1 | 0 | 0 | 0 | 1 | 0 |  | NA |  | NA | 2 | 2 | 2 | 0 | 0 | 0 |  | 3 | 23 | 0 | 7 | 0 |  |
| 8627 | 1 | 18.024 | 5.1300001 | 28.462051 | 71.537949 | 1 | 13.399042 | 0 | -0.46975747 | 2 | 127.18694 | 17.973886 | 84.199997 | 1 | 6 | 5 | 1 | 1 | 1 | 1 | 1 | 1 | 0 | 141.1945863 | 3.500995967 | 94.84525784 | -0.836876171 | 2 | 2 | 2 | 0 | 0 | 0 | 0 | 3 | 23 | 0 | 7 | 0 |  |
| 8627 | 1 | 18.767 | 4.3600001 | 23.232269 | 76.767731 | 1 | 13.691992 | 0 | -0.46975747 | 2 | 127.18694 | 18.449444 | 80.220001 | 1 | 12 | 5 | 1 | 1 | 1 | 1 | 1 | 1 | 1 | 102.2873453 | 0.199901052 | 102.3964974 | 0.433075298 | 2 | 2 | 2 | 0 | 0 | 0 |  | 3 | 14 | 1 | 3.4000001 | 1 |  |
| 8628 | 1 | 17.671 | 6.092 | 34.474564 | 65.525436 | 0 | 10.313484 | 0 | 0.44824421 | 2 | 67.578728 | 23.296667 | 73.737 | 1 | 0 | 5 |  | 1 | 0 | 0 | 0 | 0 |  | 93.85055541 | -0.55028721 | 77.04016557 | -2.847742629 | 2 | 2 |  | 0 | 0 | 0 | 1 | 1 |  | | | | |
| 8628 | 1 | 23.52 | 7.1300001 | 30.314627 | 69.685371 | 0 | 11.014374 | 0 | 0.44824421 | 2 | 67.578728 | 21.465555 | 77.559998 | 1 | 1 | 5 |  | 1 | 1 | 0 | 0 | 1 | 0 | 121.848463 | 1.909603729 | 76.74571111 | -2.879079784 | 2 | 2 | 2 | 0 | 0 | 0 |  | 1 | 19 | 1 | 6.4000001 | 1 |  |
| 8628 | 0 | 17.555 | 6.3600001 | 36.228996 | 63.771004 | 0 | 11.088296 | 0 | 0.44824421 | 2 | 67.578728 | 20.713892 | 79.059998 | 1 | 3 | 5 |  | 0 | 0 | 0 | 0 | 0 | 0 |  | NA |  | NA | 2 | 2 | 2 | 0 | 0 | 0 |  | 1 | 24 | 0 | 7 | 0 |  |
| 8628 | 1 | 20.712999 | 6.4000001 | 30.898472 | 69.101532 | 0 | 11.162218 | 0 | 0.44824421 | 2 | 67.578728 | 19.910553 | 79.849998 | 1 | 5 | 5 |  | 1 | 0 | 1 | 0 | 1 | 0 |  | NA |  | NA | 2 | 2 | 2 | 0 | 0 | 0 |  | 1 | 18 | 1 | 5.5999999 | 1 |  |
| 8628 | 1 | 23.534 | 5.7399998 | 24.390244 | 75.609756 | 0 | 11.206023 | 0 | 0.44824421 | 2 | 67.578728 | 17.475555 | 84.089996 | 1 | 6 | 5 |  | 1 | 1 | 1 | 1 | 0 |  | 108.9589303 | 0.790647841 | 80.67266712 | -2.548573065 | 2 | 2 | 2 | 0 | 0 | 0 | 1 | 1 | 21 | 0 | 5.8000002 | 1 |  |
| 8628 | 0 | 18.261 | 5.6599998 | 30.995016 | 69.004982 | 0 | 11.507187 | 0 | 0.44824421 | 2 | 67.578728 | 17.164444 | 83.089996 | 1 | 12 | 5 |  | 0 | 0 | 0 | 0 | 0 |  | 115.1997213 | 1.333002905 | 81.57852325 | -2.464563022 | 2 | 2 | 1 | 0 | 0 | 0 |  | 1 | 17 | 1 | 5.5 | 1 |  |
| 8629 | 0 | 17.857 | 6.217 | 34.815479 | 65.184525 | 1 | 14.425735 | 1 | 2.3033004 | 2 | 99.598564 | 23.784445 | 73.57 | 1 | 0 | 5 | 0 | 0 | 0 | 0 | 0 | 0 | 0 | 108.9556317 | 0.818061973 | 97.70783906 | -0.347789238 | 2 | 2 |  | 0 | 0 | 0 | 0 | 4 |  | | | | |
| 8629 | 0 | 23.548 | 7.1999998 | 30.575844 | 69.424156 | 1 | 15.154004 | 1 | 2.3033004 | 2 | 99.598564 | 21.613888 | 76.709999 | 1 | 1 | 5 | 0 | 0 | 0 | 0 | 0 | 0 | 0 | 109.5806933 | 0.87382961 | 98.17375914 | -0.27425945 | 2 | 2 | 2 | 0 | 0 | 0 |  | 4 | 24 | 0 | 7 | 0 |  |
| 8629 | 0 | 17.649 | 6.5999999 | 37.395885 | 62.604115 | 1 | 15.225188 | 1 | 2.3033004 | 2 | 99.598564 | 20.866665 | 78.330002 | 1 | 3 | 5 | 0 | 0 | 0 | 0 | 0 | 0 | 0 |  | NA |  | NA | 2 | 2 | 2 | 0 | 0 | 0 |  | 4 | 24 | 0 | 7 | 0 |  |
| 8629 | 0 | 21.267 | 6.79 | 31.927399 | 68.072601 | 1 | 15.301848 | 1 | 2.3033004 | 2 | 99.598564 | 19.978888 | 79.309998 | 1 | 5 | 5 | 0 | 0 | 0 | 0 | 0 | 0 | 0 |  | NA |  | NA | 2 | 2 | 2 | 0 | 0 | 0 |  | 4 | 24 | 0 | 7 | 0 |  |
| 8629 | 0 | 24.375 | 5.6999998 | 23.384615 | 76.615387 | 1 | 15.342916 | 1 | 2.3033004 | 2 | 99.598564 | 17.637779 | 83.120003 | 1 | 6 | 5 | 0 | 0 | 0 | 0 | 0 | 0 | 0 | 107.7842785 | 0.710066538 | 98.31420911 | -0.252574876 | 2 | 2 | 2 | 0 | 0 | 0 | 0 | 4 | 24 | 0 | 7 | 0 |  |
| 8629 | 0 | 16.601999 | 5.4400001 | 32.767139 | 67.232864 | 1 | 15.635866 | 1 | 2.3033004 | 2 | 99.598564 | 18.336664 | 77.779999 | 1 | 12 | 5 | 0 | 0 | 0 | 0 | 0 | 0 | 0 | 104.1735394 | 0.380884927 | 103.1380842 | 0.486197519 | 2 | 2 | 2 | 0 | 0 | 0 |  | 4 | 24 | 0 | 7 | 0 |  |
| 8630 | 1 | 17.666 | 6.135 | 34.727726 | 65.272278 | 1 | 12.487337 | 0 | -1.2354536 | 3 | 125.67046 | 23.68 | 73.365 | 1 | 0 | 5 | 1 | 0 | 0 | 0 | 0 | 1 | 0 | 131.7893412 | 2.736858251 | 101.7786304 | 0.323121492 | 2 | 2 |  | 0 | 0 | 0 | 0 | 4 |  | | | | |
| 8630 | 1 | 24.065001 | 7.3400002 | 30.500727 | 69.499275 | 1 | 13.182752 | 0 | -1.2354536 | 3 | 125.67046 | 21.772223 | 76.120003 | 1 | 1 | 5 | 1 | 1 | 0 | 0 | 0 | 1 | 0 | 128.0834549 | 2.412902993 | 103.0269226 | 0.555499952 | 2 | 2 | 2 | 0 | 0 | 0 |  | 4 | 23 | 0 | 7 | 0 |  |
| 8630 | 1 | 17.958 | 6.5300002 | 36.362625 | 63.637375 | 1 | 13.264887 | 0 | -1.2354536 | 3 | 125.67046 | 20.798891 | 79.089996 | 1 | 3 | 5 | 1 | 1 | 0 | 0 | 0 | 1 | 0 |  | NA |  | NA | 2 | 2 | 2 | 0 | 0 | 0 |  | 4 | 23 | 0 | 6.9000001 | 1 |  |
| 8630 | 0 | 20.954 | 6.5700002 | 31.354397 | 68.645607 | 1 | 13.341547 | 0 | -1.2354536 | 3 | 125.67046 | 19.941666 | 79.610001 | 1 | 5 | 5 | 1 | 0 | 0 | 0 | 0 | 0 | 0 |  | NA |  | NA | 2 | 2 | 2 | 0 | 0 | 0 |  | 4 | 23 | 0 | 7 | 0 |  |
| 8630 | 1 | 23.943001 | 5.7399998 | 23.973602 | 76.026398 | 1 | 13.385352 | 0 | -1.2354536 | 3 | 125.67046 | 17.530556 | 83.68 | 1 | 6 | 5 | 1 | 1 | 1 | 1 | 0 | 1 | 0 | 142.0264153 | 3.569997111 | 100.0454273 | 0.007961899 | 2 | 2 | 2 | 0 | 0 | 0 | 0 | 4 | 18 | 1 | 5.0999999 | 1 |  |
| 8630 | 0 | 16.943001 | 5.3099999 | 31.340374 | 68.659622 | 1 | 13.675565 | 0 | -1.2354536 | 3 | 125.67046 | 18.322779 | 78.269997 | 1 | 12 | 5 | 1 | 0 | 0 | 0 | 0 | 0 | 0 | 138.1113603 | 3.239537429 | 102.1005504 | 0.378050571 | 2 | 2 | 2 | 0 | 0 | 0 |  | 4 | 25 | 0 | 7 | 0 |  |
| 8631 | 1 | 18.271 | 6.19 | 33.878826 | 66.121178 | 1 | 14.179329 | 1 | 2.2397017 | 4 | 115.20515 | 21.984444 | 76.197 | 1 | 0 | 5 | 1 | 1 | 1 | 1 | 1 | 1 | 1 | 121.5264518 | 1.961419985 | 101.4144158 | 0.222441007 | 2 | 2 |  | 1 | 0 | 0 | 0 | 3 |  | | | | |
| 8631 | 0 | 23.927999 | 7.2600002 | 30.341024 | 69.658974 | 1 | 14.918549 | 1 | 2.2397017 | 4 | 115.20515 | 21.587778 | 77.110001 | 1 | 1 | 5 | 1 | 0 | 0 | 0 | 0 | 0 | 0 | 125.8411983 | 2.349036292 | 99.92305942 | -0.011773956 | 2 | 2 | 2 | 1 | 0 | 0 |  | 3 | 10 | 1 | 6.3000002 | 1 |  |
| 8631 | 0 | 18.472 | 6.2800002 | 33.997402 | 66.002594 | 1 | 15.000685 | 1 | 2.2397017 | 4 | 115.20515 | 20.639999 | 79.779999 | 1 | 3 | 5 | 1 | 0 | 0 | 0 | 0 | 0 | 0 |  | NA |  | NA | 2 | 2 | 2 | 1 | 0 | 0 |  | 3 | 23 | 0 | 7 | 0 |  |
| 8631 | 1 | 20.024 | 6.0900002 | 30.413506 | 69.586494 | 1 | 15.077344 | 1 | 2.2397017 | 4 | 115.20515 | 19.860556 | 80.110001 | 1 | 5 | 5 | 1 | 1 | 0 | 0 | 0 | 0 | 0 |  | NA |  | NA | 2 | 2 | 2 | 1 | 0 | 0 |  | 3 | 16 | 1 | 6.0999999 | 1 |  |
| 8631 | 0 | 23.190001 | 5.71 | 24.622681 | 75.377319 | 1 | 15.137577 | 1 | 2.2397017 | 4 | 115.20515 | 17.275557 | 84.839996 | 1 | 6 | 5 | 1 | 0 | 0 | 0 | 0 | 0 | 0 | 119.2311876 | 1.750077512 | 101.2728623 | 0.196103269 | 2 | 2 | 2 | 1 | 0 | 0 | 0 | 3 | 17 | 1 | 7 | 0 |  |
| 8631 | 0 | 17.872 | 5.0300002 | 28.144585 | 71.855415 | 1 | 15.422314 | 1 | 2.2397017 | 4 | 115.20515 | 18.293333 | 79.07 | 1 | 12 | 5 | 1 | 0 | 0 | 0 | 0 | 0 | 0 | 118.9073406 | 1.719923408 | 98.37822805 | -0.242754346 | 2 | 2 | 2 | 1 | 0 | 0 |  | 3 | 24 | 0 | 7 | 0 |  |
| 8632 | 0 | 17.95 | 6.059 | 33.754875 | 66.245125 | 1 | 14.893909 | 1 | 2.2397017 | 2 | 123.4588 | 22.501667 | 75.191 | 1 | 0 | 5 | 1 | 0 | 0 | 0 | 0 | 0 | 0 | 144.9220578 | 4.067319131 | 91.24300811 | -1.247194516 | 2 | 2 |  | 0 | 0 | 0 | 0 | 1 |  | | | | |
| 8632 | 1 | 23.938 | 7.2600002 | 30.328348 | 69.671654 | 1 | 15.611225 | 1 | 2.2397017 | 2 | 123.4588 | 21.59333 | 77.080002 | 1 | 1 | 5 | 1 | 1 | 0 | 0 | 0 | 1 | 0 | 120.8344645 | 1.893878278 | 92.83933961 | -1.020813735 | 2 | 2 | 2 | 0 | 0 | 0 |  | 1 | 24 | 0 | 7 | 0 |  |
| 8632 | 1 | 18.517 | 6.3000002 | 34.022793 | 65.977211 | 1 | 15.69336 | 1 | 2.2397017 | 2 | 123.4588 | 20.647223 | 79.739998 | 1 | 3 | 5 | 1 | 1 | 0 | 0 | 0 | 1 | 0 |  | NA |  | NA | 2 | 2 | 2 | 0 | 0 | 0 |  | 1 | 24 | 0 | 7 | 0 |  |
| 8632 | 1 | 20.035999 | 6.1100001 | 30.495111 | 69.50489 | 1 | 15.77002 | 1 | 2.2397017 | 2 | 123.4588 | 19.864443 | 80.089996 | 1 | 5 | 5 | 1 | 1 | 0 | 0 | 0 | 1 | 0 |  | NA |  | NA | 2 | 2 | 2 | 0 | 0 | 0 |  | 1 | 24 | 0 | 7 | 0 |  |
| 8632 | 1 | 23.098 | 5.6500001 | 24.460993 | 75.539009 | 1 | 15.811089 | 1 | 2.2397017 | 2 | 123.4588 | 17.458332 | 84.370003 | 1 | 6 | 5 | 1 | 0 | 0 | 0 | 0 | 1 | 0 | 96.62144655 | -0.308854908 | 95.90077952 | -0.597329257 | 2 | 2 | 2 | 0 | 0 | 0 | 1 | 1 | 23 | 0 | 7 | 0 |  |
| 8632 | 1 | 17.863001 | 5.0500002 | 28.270727 | 71.729271 | 1 | 16.106775 | 1 | 2.2397017 | 2 | 123.4588 | 18.29611 | 79.019997 | 1 | 12 | 5 | 1 | 1 | 0 | 0 | 0 | 1 | 0 | 111.4940542 | 1.046319414 | 83.90248635 | -2.112908586 | 2 | 2 | 2 | 0 | 0 | 0 |  | 1 | 21 | 0 | 6 | 1 |  |
| 8634 | 0 | 17.284 | 5.765 | 33.354549 | 66.645454 | 1 | 12.832307 | 1 | 0.36403459 | 2 | 106.28562 | 20.07 | 77.505 | 1 | 0 | 4 | 1 | 0 | 0 | 0 | 0 | 0 | 0 | 109.4251914 | 0.862955855 | 107.3648881 | 1.251708081 | 2 | 2 |  | 0 | 0 | 0 | 0 | 3 |  | | | | |
| 8634 | 0 | 22.466999 | 6.9099998 | 30.75622 | 69.243782 | 1 | 13.544147 | 1 | 0.36403459 | 2 | 106.28562 | 21.030558 | 78.980003 | 1 | 1 | 4 | 1 | 0 | 0 | 0 | 0 | 0 | 0 | 117.1747505 | 1.568288814 | 93.39653051 | -0.982113763 | 2 | 2 | 2 | 0 | 0 | 0 |  | 3 | 22 | 0 | 7 | 0 |  |
| 8634 | 1 | 16.572001 | 5.4299998 | 32.766109 | 67.233894 | 1 | 13.61807 | 1 | 0.36403459 | 2 | 106.28562 | 20.282225 | 81.470001 | 1 | 3 | 4 | 1 | 1 | 0 | 1 | 1 | 0 | 0 |  | NA |  | NA | 2 | 2 | 2 | 0 | 0 | 0 |  | 3 | 25 | 0 | 7 | 0 |  |
| 8634 | 1 | 19.233 | 5.2600002 | 27.348829 | 72.651169 | 1 | 13.738535 | 1 | 0.36403459 | 2 | 106.28562 | 18.180555 | 83.860001 | 1 | 6 | 4 | 1 | 1 | 1 | 1 | 1 | 1 | 1 | 106.3897238 | 0.584750511 | 106.6167938 | 1.096526591 | 2 | 2 | 2 | 0 | 0 | 0 | 0 | 3 | 24 | 0 | 7 | 0 |  |
| 8634 | 0 | 19.212999 | 4.5500002 | 23.681885 | 76.318115 | 1 | 14.045175 | 1 | 0.36403459 | 2 | 106.28562 | 17.985001 | 80.160004 | 1 | 12 | 4 | 1 | 0 | 0 | 0 | 0 | 0 | 0 | 103.0526478 | 0.279454434 | 113.9728494 | 2.440202927 | 2 | 2 | 2 | 0 | 0 | 0 |  | 3 | 24 | 0 | 7 | 0 |  |
| 8635 | 1 | 17.616 | 5.791 | 32.873524 | 67.126472 | 1 | 16.246407 | 1 | -0.83691978 | 2 | 98.846489 | 21.507223 | 74.75 | 1 | 0 | 4 | 0 | 1 | 0 | 1 | 1 | 1 | 0 | 100.2151499 | 0.019637255 | 105.4953253 | 0.858387728 | 2 | 2 |  | 1 | 0 | 0 | 0 | 3 |  | | | | |
| 8635 | 1 | 22.815001 | 7.0500002 | 30.900723 | 69.099274 | 1 | 16.900753 | 1 | -0.83691978 | 2 | 98.846489 | 21.170557 | 78.449997 | 1 | 1 | 4 | 0 | 1 | 0 | 0 | 0 | 0 | 0 | 112.4389078 | 1.131039279 | 97.13419794 | -0.415146884 | 2 | 2 | 2 | 1 | 0 | 0 |  | 3 | 23 | 0 | 7 | 0 |  |
| 8635 | 1 | 17.389999 | 5.71 | 32.834965 | 67.165039 | 1 | 16.977413 | 1 | -0.83691978 | 2 | 98.846489 | 20.380554 | 81.400002 | 1 | 3 | 4 | 0 | 1 | 0 | 0 | 0 | 0 | 0 |  | NA |  | NA | 2 | 2 | 2 | 1 | 0 | 0 |  | 3 | 23 | 0 | 7 | 0 |  |
| 8635 | 1 | 20.629999 | 5.4299998 | 26.320892 | 73.679108 | 1 | 17.097878 | 1 | -0.83691978 | 2 | 98.846489 | 17.306667 | 85.639999 | 1 | 6 | 4 | 0 | 1 | 1 |  | | 0 | 0 | 111.7432834 | 1.067792508 | 96.12909437 | -0.554919462 | 2 | 2 | 2 | 1 | 0 | 0 | 0 | 3 | 25 | 0 | 7 | 0 |  |
| 8635 | 0 | 18.799999 | 4.5500002 | 24.202129 | 75.797867 | 1 | 17.404518 | 1 | -0.83691978 | 2 | 98.846489 | 18.124445 | 79.769997 | 1 | 12 | 4 | 0 | 0 | 0 | 0 | 0 | 0 | 0 | 108.4753635 | 0.771079059 | 96.16525405 | -0.547986316 | 2 | 2 | 2 | 1 | 0 | 0 |  | 3 | 24 | 0 | 7 | 0 |  |
| 8638 | 1 | 16.878 | 5.66 | 33.534779 | 66.465218 | 0 | 11.926078 | 1 | 2.2397017 | 2 | 115.02298 | 19.012777 | 78.857 | 1 | 0 | 4 | 1 | 1 | 0 | 0 | 0 | 0 |  | 119.7681289 | 1.804924444 | 102.5785873 | 0.426591308 | 2 | 2 |  | 0 | 0 | 0 | 0 | 4 |  | | | | |
| 8638 | 1 | 22.266001 | 6.8899999 | 30.944038 | 69.055962 | 1 | 12.635181 | 1 | 2.2397017 | 2 | 115.02298 | 20.877779 | 79.300003 | 1 | 1 | 4 | 1 | 1 | 1 | 1 | 1 | 1 | 1 | 115.5081963 | 1.418007935 | 105.2821372 | 0.885225325 | 2 | 2 | 2 | 0 | 0 | 0 |  | 4 | 24 | 0 | 7 | 0 |  |
| 8638 | 1 | 16.011999 | 5.1100001 | 31.913567 | 68.086433 | 1 | 12.714579 | 1 | 2.2397017 | 2 | 115.02298 | 20.128336 | 81.760002 | 1 | 3 | 4 | 1 | 0 | 0 | 1 | 0 | 1 | 0 |  | NA |  | NA | 2 | 2 | 2 | 0 | 0 | 0 |  | 4 | 24 | 0 | 7 | 0 |  |
| 8638 | 1 | 18.402 | 5.1399999 | 27.931746 | 72.068253 | 1 | 12.826831 | 1 | 2.2397017 | 2 | 115.02298 | 18.202223 | 84.110001 | 1 | 6 | 4 | 1 | 1 | 1 | 1 | 1 | 1 | 1 | 79.75243338 | -1.868784568 | 88.85985463 | -1.609642539 | 2 | 2 | 2 | 0 | 0 | 0 | 1 | 4 | 14 | 1 | 4.6999998 | 1 |  |
| 8638 | 0 | 19.834999 | 4.5900002 | 23.140915 | 76.859085 | 1 | 13.144422 | 1 | 2.2397017 | 2 | 115.02298 | 17.780554 | 80.18 | 1 | 12 | 4 | 1 | 0 | 0 | 0 | 0 | 0 | 0 | 103.1782494 | 0.291365477 | 110.0597636 | 1.737452877 | 2 | 2 | 2 | 0 | 0 | 0 |  | 4 | 24 | 0 | 7 | 0 |  |
| 8641 | 1 | 18.135 | 6.074 | 33.493244 | 66.506752 | 0 | 9.4976044 | 0 | 2.4412034 | 3 | 143.28046 | 21.263334 | 77.704 | 1 | 0 | 5 | 1 | 1 | 0 | 1 | 0 | 0 |  | 151.0888698 | 4.351561158 | 101.4811356 | 0.275424883 |  | | | | | 0 | 0 | 4 |  | | | | |
| 8641 | 1 | 22.045 | 6.7399998 | 30.573826 | 69.426178 | 0 | 10.250513 | 0 | 2.4412034 | 3 | 143.28046 | 21.252775 | 78.339996 | 1 | 1 | 5 | 1 | 1 | 0 | 0 | 0 | 0 |  | 143.9496735 | 3.780233438 | 100.6553782 | 0.119572755 |  | | 2 |  | | 0 |  | 4 | 24 | 0 | 6.9000001 | 1 |  |
| 8641 | 0 | 16.933001 | 5.6999998 | 33.662075 | 66.337921 | 0 | 10.310746 | 0 | 2.4412034 | 3 | 143.28046 | 20.528332 | 81.449997 | 1 | 3 | 5 | 1 | 0 | 0 | 0 | 0 | 0 |  | | NA |  | NA |  | | 1 |  | | 0 |  | 4 | 19 | 1 | 6.8000002 | 1 |  |
| 8641 | 1 | 17.309999 | 5.3699999 | 31.022532 | 68.97747 | 0 | 10.387406 | 0 | 2.4412034 | 3 | 143.28046 | 19.874445 | 80.510002 | 1 | 5 | 5 | 1 | 1 | 0 | 0 | 0 | 0 |  | | NA |  | NA |  | | 1 |  | | 0 |  | 4 | 24 | 0 | 6.8000002 | 1 |  |
| 8641 | 0 | 18.892 | 5.5500002 | 29.377516 | 70.622482 | 0 | 10.42026 | 0 | 2.4412034 | 3 | 143.28046 | 18.326111 | 84.25 | 1 | 6 | 5 | 1 | 0 | 0 | 0 | 0 | 0 |  | 148.5838072 | 4.167353755 | 100.5698242 | 0.103681489 |  | | 2 |  | | 0 | 0 | 4 | 24 | 0 | 7 | 0 |  |
| 8641 | 0 | 17.774 | 4.3899999 | 24.698997 | 75.301003 | 0 | 10.737851 | 0 | 2.4412034 | 3 | 143.28046 | 18.232224 | 80.839996 | 1 | 12 | 5 | 1 | 0 | 0 | 0 | 0 | 0 |  | 137.0805648 | 3.206383307 | 102.6569149 | 0.498494474 |  | | 2 |  | | 0 |  | 4 | 27 | 0 | 7 | 0 |  |
| 8642 | 0 | 18.333 | 5.394 | 29.422354 | 70.577644 | 1 | 12.5859 | 1 | 2.3033004 | 3 | 76.534172 | 23.171112 | 74.072 | 1 | 0 | 5 | 1 | 0 | 0 | 0 | 0 | 0 | 0 | 84.84262394 | -1.397068931 | 97.28901335 | -0.424005354 | 2 | 2 |  | 1 |  | 0 | 1 | 3 |  | | | | |
| 8642 | 0 | 24.698999 | 5.8899999 | 23.84712 | 76.152878 | 1 | 13.322382 | 1 | 2.3033004 | 3 | 76.534172 | 21.313889 | 77.82 | 1 | 1 | 5 | 1 | 0 | 0 | 0 | 0 | 0 | 0 | 88.59379618 | -1.049522759 | 98.40566654 | -0.248645759 | 2 | 2 | 2 | 1 |  | 0 |  | 3 | 23 | 0 | 7 | 0 |  |
| 8642 | 0 | 14.283 | 4.9299998 | 34.516556 | 65.483444 | 1 | 13.385352 | 1 | 2.3033004 | 3 | 76.534172 | 20.566666 | 79.18 | 1 | 3 | 5 | 1 | 0 | 0 | 0 | 0 | 0 | 0 |  | NA |  | NA | 2 | 2 | 2 | 1 |  | 0 |  | 3 | 23 | 0 | 7 | 0 |  |
| 8642 | 0 | 35.18 | 5.0900002 | 14.468449 | 85.531555 | 1 | 13.462012 | 1 | 2.3033004 | 3 | 76.534172 | 19.845556 | 79.720001 | 1 | 5 | 5 | 1 | 0 | 0 | 0 | 0 | 0 | 0 |  | NA |  | NA | 2 | 2 | 2 | 1 |  | 0 |  | 3 | 24 | 0 | 7 | 0 |  |
| 8642 | 0 | 22.346001 | 5.1399999 | 23.001879 | 76.998123 | 1 | 13.508555 | 1 | 2.3033004 | 3 | 76.534172 | 17.223333 | 84.68 | 1 | 6 | 5 | 1 | 0 | 0 | 0 | 0 | 0 | 0 | 95.87912349 | -0.378299629 | 97.42312814 | -0.396893876 | 2 | 2 | 2 | 1 |  | 0 | 0 | 3 | 24 | 0 | 7 | 0 |  |
| 8642 | 0 | 16.632 | 4.2399998 | 25.493025 | 74.506973 | 1 | 13.837098 | 1 | 2.3033004 | 3 | 76.534172 | 17.203333 | 82.940002 | 1 | 12 | 5 | 1 | 0 | 0 | 0 | 0 | 0 | 0 | 85.89566297 | -1.297617327 | 98.3127571 | -0.260332791 | 2 | 2 | 2 | 1 |  | 0 |  | 3 | 25 | 0 | 7 | 0 |  |
| 8643 | 0 | 18.16 | 5.497 | 30.269823 | 69.730179 | 1 | 14.184805 | 0 | 2.2397017 | 3 | 94.498924 | 22.864445 | 74.529 | 1 | 0 | 5 | 0 | 0 | 0 | 0 | 0 | 0 | 0 | 117.5798498 | 1.51209497 | 85.95925266 | -1.989694639 | 2 | 2 |  | 0 | 0 | 0 | 1 | 2 |  | | | | |
| 8643 | 0 | 24.084 | 6.6999998 | 27.819298 | 72.180702 | 1 | 14.888433 | 0 | 2.2397017 | 3 | 94.498924 | 21.557777 | 76.860001 | 1 | 1 | 5 | 0 | 0 | 0 | 0 | 0 | 0 | 0 | 101.4938322 | 0.129629504 | 84.42453707 | -2.150257193 | 2 | 2 | 2 | 0 | 0 | 0 |  | 2 | 19 | 1 | 6.4000001 | 1 |  |
| 8643 | 0 | 17.879 | 5.1799998 | 28.972536 | 71.027466 | 1 | 14.973306 | 0 | 2.2397017 | 3 | 94.498924 | 20.594999 | 80.029999 | 1 | 3 | 5 | 0 | 0 | 0 | 0 | 0 | 0 | 0 |  | NA |  | NA | 2 | 2 | 2 | 0 | 0 | 0 |  | 2 | 20 | 0 | 6.8000002 | 1 |  |
| 8643 | 0 | 31.379 | 5.3000002 | 16.890278 | 83.109726 | 1 | 15.049966 | 0 | 2.2397017 | 3 | 94.498924 | 19.833332 | 80.089996 | 1 | 5 | 5 | 0 | 0 | 0 | 0 | 0 | 0 | 0 |  | NA |  | NA | 2 | 2 | 2 | 0 | 0 | 0 |  | 2 | 24 | 0 | 7 | 0 |  |
| 8643 | 1 | 22.545 | 5.25 | 23.28676 | 76.713242 | 1 | 15.085558 | 0 | 2.2397017 | 3 | 94.498924 | 17.391111 | 84.68 | 1 | 6 | 5 | 0 | 1 | 0 | 1 | 0 | 1 | 1 | 109.9927969 | 0.859893693 | 81.31210117 | -2.464973184 | 2 | 2 | 2 | 0 | 0 | 0 | 1 | 2 | 17 | 1 | 5.5999999 | 1 |  |
| 8643 | 1 | 17.018999 | 4.46 | 26.206007 | 73.793991 | 1 | 15.397673 | 0 | 2.2397017 | 3 | 94.498924 | 17.278889 | 82.970001 | 1 | 12 | 5 | 0 | 1 | 0 | 0 | 1 | 1 | 1 | 116.0307137 | 1.370224261 | 85.59214353 | -2.016384316 | 2 | 2 | 2 | 0 | 0 | 0 |  | 2 | 19 | 1 | 7 | 0 |  |
| 8644 | 1 | 18.41 | 5.178 | 28.126019 | 71.873985 | 1 | 16.955509 | 0 | 1.9938918 | 3 | 117.93257 | 24.258888 | 72.369 | 1 | 0 | 5 | 0 | 1 | 0 | 0 | 0 | 1 | 0 | 127.559426 | 2.316569223 | 97.76687292 | -0.364267506 | 2 | 2 |  | 0 | 0 | 0 | 0 | 1 |  | | | | |
| 8644 | 0 | 23.785 | 6.23 | 26.192978 | 73.807022 | 1 | 17.650925 | 0 | 1.9938918 | 3 | 117.93257 | 21.627777 | 76.050003 | 1 | 1 | 5 | 0 | 0 | 0 | 0 | 0 | 0 | 0 | 128.1432577 | 2.357318434 | 102.3542548 | 0.404334038 | 2 | 2 | 2 | 0 | 0 | 0 |  | 1 | 24 | 0 | 7 | 0 |  |
| 8644 | 0 | 18.25 | 4.6799998 | 25.643835 | 74.356163 | 1 | 17.735798 | 0 | 1.9938918 | 3 | 117.93257 | 20.661112 | 79.220001 | 1 | 3 | 5 | 0 | 0 | 0 | 0 | 0 | 0 | 0 |  | NA |  | NA | 2 | 2 | 2 | 0 | 0 | 0 |  | 1 | 24 | 0 | 7 | 0 |  |
| 8644 | 0 | 39.018002 | 4.9699998 | 12.73771 | 87.262291 | 1 | 17.812458 | 0 | 1.9938918 | 3 | 117.93257 | 19.87389 | 79.440002 | 1 | 5 | 5 | 0 | 0 | 0 | 0 | 0 | 0 | 0 |  | NA |  | NA | 2 | 2 | 2 | 0 | 0 | 0 |  | 1 | 24 | 0 | 7 | 0 |  |
| 8644 | 0 | 23.48 | 4.9499998 | 21.08177 | 78.918228 | 1 | 17.848049 | 0 | 1.9938918 | 3 | 117.93257 | 17.412222 | 83.839996 | 1 | 6 | 5 | 0 | 0 | 0 | 0 | 0 | 0 | 0 | 123.2734944 | 1.954964826 | 102.2915925 | 0.392247672 | 2 | 2 | 2 | 0 | 0 | 0 | 0 | 1 | 24 | 0 | 7 | 0 |  |
| 8644 | 0 | 16.275 | 4.04 | 24.823349 | 75.176651 | 1 | 18.160164 | 0 | 1.9938918 | 3 | 117.93257 | 17.144445 | 82.970001 | 1 | 12 | 5 | 0 | 0 | 0 | 0 | 0 | 0 | 0 | 129.674375 | 2.477610722 | 98.82629421 | -0.191398025 | 2 | 2 | 2 | 0 | 0 | 0 |  | 1 | 24 | 0 | 7 | 0 |  |
| 8648 | 0 | 17.701 | 5.758 | 32.529236 | 67.470764 | 1 | 15.383984 | 0 | -0.40615869 | 3 | 83.606041 | 22.314444 | 75.417 | 1 | 0 | 3 | 1 | 0 | 0 | 0 | 0 | 0 | 0 | 88.58079681 | -0.999751782 | 100.9538563 | 0.165468719 | 2 | 2 |  | 0 | 0 | 0 | 0 | 4 |  | | | | |
| 8648 | 0 | 23.566999 | 6.71 | 28.472017 | 71.527985 | 1 | 16.082136 | 0 | -0.40615869 | 3 | 83.606041 | 21.266111 | 78.75 | 1 | 1 | 3 | 1 | 0 | 0 | 0 | 0 | 0 | 0 | 86.31568106 | -1.196230282 | 106.2481384 | 1.153831236 | 2 | 2 | 2 | 0 | 0 | 0 |  | 4 | 25 | 0 | 7 | 0 |  |
| 8648 | 1 | 20.257999 | 5.8299999 | 28.778755 | 71.221245 | 1 | 16.21629 | 0 | -0.40615869 | 3 | 83.606041 | 19.781668 | 81.050003 | 1 | 5 | 3 | 1 | 1 | 1 | 1 | 1 | 1 | 1 |  | NA |  | NA | 2 | 2 | 2 | 0 | 0 | 0 |  | 4 | 21 | 0 | 6.6999998 | 1 |  |
| 8648 | 0 | 18.676001 | 4.5900002 | 24.576998 | 75.423004 | 1 | 16.547569 | 0 | -0.40615869 | 3 | 83.606041 | 17.970556 | 81.400002 | 1 | 12 | 3 | 1 | 0 | 0 | 0 | 0 | 0 | 0 | 90.65410373 | -0.8116986 | 97.65164387 | -0.384261696 | 2 | 2 | 2 | 0 | 0 | 0 |  | 4 | 25 | 0 | 7 | 0 |  |
| 8649 | 1 | 18.076 | 5.52 | 30.537729 | 69.462273 | 0 | 11.687885 | 0 | -0.36892569 | 4 | 115.68256 | 22.373333 | 74.95 | 1 | 0 | 2 | 1 | 1 | 1 | 1 | 1 | 1 |  | 113.5172399 | 1.185944473 | 108.867179 | 1.811449872 | 2 | 2 |  | 0 | 0 | 0 | 0 | 2 |  | | | | |
| 8649 | 0 | 24.309 | 6.7600002 | 27.808632 | 72.191368 | 1 | 12.391513 | 0 | -0.36892569 | 4 | 115.68256 | 21.577221 | 76.68 | 1 | 1 | 2 | 1 | 0 | 0 | 0 | 0 | 0 | 0 | 108.5475604 | 0.749760007 | 106.3504987 | 1.236858973 | 2 | 2 | 2 | 0 | 0 | 0 |  | 2 | 21 | 0 | 7 | 0 |  |
| 8649 | 0 | 17.561001 | 5.1500001 | 29.326347 | 70.673653 | 1 | 12.476386 | 0 | -0.36892569 | 4 | 115.68256 | 20.55611 | 80.150002 | 1 | 3 | 2 | 1 | 0 | 0 | 0 | 0 | 0 | 0 |  | NA |  | NA | 2 | 2 | 2 | 0 | 0 | 0 |  | 2 | 22 | 0 | 6.8000002 | 1 |  |
| 8653 | 0 | 18.248 | 6.338 | 34.732574 | 65.267426 | 1 | 12.539356 | 0 | -0.15840714 | 3 | 124.32761 | 21.859444 | 74.406 | 1 | 0 | 5 | 1 | 0 | 0 | 0 | 0 | 0 | 0 | 131.0833259 | 2.676497077 | 101.4246189 | 0.257284968 | 2 | 2 |  | 1 | 0 | 0 | 0 | 4 |  | | | | |
| 8653 | 0 | 23.242001 | 7.29 | 31.365629 | 68.634369 | 1 | 13.188228 | 0 | -0.15840714 | 3 | 124.32761 | 21.319445 | 78.720001 | 1 | 1 | 5 | 1 | 0 | 0 | 0 | 0 | 0 | 0 | 114.5176501 | 1.260395703 | 109.0154596 | 1.805070129 | 2 | 2 | 2 | 1 | 0 | 0 |  | 4 | 24 | 0 | 6.9000001 | 1 |  |
| 8653 | 0 | 17.142 | 6.52 | 38.035236 | 61.964764 | 1 | 13.264887 | 0 | -0.15840714 | 3 | 124.32761 | 20.617777 | 80.029999 | 1 | 3 | 5 | 1 | 0 | 0 | 0 | 0 | 0 | 0 |  | NA |  | NA | 2 | 2 | 2 | 1 | 0 | 0 |  | 4 | 24 | 0 | 7 | 0 |  |
| 8653 | 0 | 22.295 | 6.3600001 | 28.526575 | 71.473427 | 1 | 13.341547 | 0 | -0.15840714 | 3 | 124.32761 | 19.71389 | 80.900002 | 1 | 5 | 5 | 1 | 0 | 0 | 0 | 0 | 0 | 0 |  | NA |  | NA | 2 | 2 | 2 | 1 | 0 | 0 |  | 4 | 23 | 0 | 7 | 0 |  |
| 8653 | 1 | 23.378 | 6.3299999 | 27.076738 | 72.923264 | 1 | 13.399042 | 0 | -0.15840714 | 3 | 124.32761 | 17.132778 | 85.629997 | 1 | 6 | 5 | 1 | 1 | 0 | 1 | 0 | 1 | 0 | 119.0449759 | 1.645064257 | 108.8808828 | 1.769047704 | 2 | 2 | 2 | 1 | 0 | 0 | 0 | 4 | 24 | 0 | 7 | 0 |  |
| 8653 | 0 | 16.514 | 5.3099999 | 32.154533 | 67.845467 | 1 | 13.689254 | 0 | -0.15840714 | 3 | 124.32761 | 18.40889 | 79.529999 | 1 | 12 | 5 | 1 | 0 | 0 | 0 | 0 | 0 | 0 | 109.5643972 | 0.830751586 | 108.8576346 | 1.756125933 | 2 | 2 | 2 | 1 | 0 | 0 |  | 4 | 24 | 0 | 7 | 0 |  |
| 8654 | 1 | 17.834 | 6.062 | 33.991253 | 66.008751 | 0 | 9.6700888 | 0 | 2.1953936 | 3 | 122.78076 | 22.745556 | 74.729 | 1 | 0 | 5 | 0 | 1 | 1 | 1 | 1 | 0 |  | 127.8088199 | 2.411160549 | 102.5413452 | 0.480321118 | 2 | 2 |  | 0 | 0 | 0 | 0 |  | | | | | |
| 8654 | 0 | 22.684999 | 6.8600001 | 30.240248 | 69.75975 | 0 | 10.398357 | 0 | 2.1953936 | 3 | 122.78076 | 21.318335 | 78.360001 | 1 | 1 | 5 | 0 | 0 | 0 | 0 | 0 | 0 |  | 133.1388523 | 2.872920996 | 98.11573951 | -0.329574047 | 2 | 2 | 2 | 0 | 0 | 0 |  | | 26 | 0 | 7 | 0 |  |
| 8654 | 0 | 17.825001 | 6.3299999 | 35.511917 | 64.488083 | 0 | 10.461328 | 0 | 2.1953936 | 3 | 122.78076 | 20.646112 | 79.239998 | 1 | 3 | 5 | 0 | 0 | 0 | 0 | 0 | 0 |  | | NA |  | NA | 2 | 2 | 2 | 0 | 0 | 0 |  | | 26 | 0 | 7 | 0 |  |
| 8654 | 1 | 20.368 | 6.2399998 | 30.636292 | 69.363708 | 0 | 10.537988 | 0 | 2.1953936 | 3 | 122.78076 | 19.883888 | 79.989998 | 1 | 5 | 5 | 0 | 1 | 0 | 0 | 0 | 0 |  | | NA |  | NA | 2 | 2 | 2 | 0 | 0 | 0 |  | | 24 | 0 | 7 | 0 |  |
| 8654 | 1 | 23.243999 | 5.6799998 | 24.436415 | 75.563583 | 0 | 10.579056 | 0 | 2.1953936 | 3 | 122.78076 | 17.479445 | 84.239998 | 1 | 6 | 5 | 0 | 1 | 1 | 1 | 1 | 0 |  | 130.58474 | 2.657012469 | 100.5634719 | 0.102386011 | 2 | 2 | 2 | 0 | 0 | 0 | 0 |  | 27 | 0 | 7 | 0 |  |
| 8654 | 0 | 17.534 | 5.1199999 | 29.200411 | 70.799591 | 0 | 10.872005 | 0 | 2.1953936 | 3 | 122.78076 | 18.300556 | 78.879997 | 1 | 12 | 5 | 0 | 0 | 0 | 0 | 0 | 0 |  | 126.080197 | 2.272767397 | 97.084659 | -0.500005824 | 2 | 2 | 2 | 0 | 0 | 0 |  | | 22 | 0 | 7 | 0 |  |
| 8655 | 1 | 17.816 | 6.096 | 34.216434 | 65.783562 | 0 | 10.997947 | 0 | -0.59363079 | 4 | 106.59646 | 22.866667 | 74.544 | 1 | 0 | 5 | 1 | 1 | 0 | 0 | 0 | 0 |  | 126.0485675 | 2.269578679 | 90.39886502 | -1.479130428 | 2 | 2 |  | 0 | 0 | 0 | 1 | 3 |  | | | | |
| 8655 | 0 | 23.989 | 7.3200002 | 30.513987 | 69.486015 | 0 | 11.70705 | 0 | -0.59363079 | 4 | 106.59646 | 21.688889 | 76.57 | 1 | 1 | 5 | 1 | 0 | 0 | 0 | 0 | 0 |  | 124.6795849 | 2.146295961 | 91.77860056 | -1.290156124 | 2 | 2 | 2 | 0 | 0 | 0 |  | 3 | 27 | 0 | 7 | 0 |  |
| 8655 | 0 | 18.017 | 6.4099998 | 35.577511 | 64.422493 | 0 | 11.789186 | 0 | -0.59363079 | 4 | 106.59646 | 20.72611 | 79.480003 | 1 | 3 | 5 | 1 | 0 | 0 | 0 | 0 | 0 |  | | NA |  | NA | 2 | 2 | 2 | 0 | 0 | 0 |  | 3 | 26 | 0 | 6.9000001 | 1 |  |
| 8655 | 0 | 20.674 | 6.3800001 | 30.860018 | 69.139984 | 0 | 11.865846 | 0 | -0.59363079 | 4 | 106.59646 | 19.906668 | 79.879997 | 1 | 5 | 5 | 1 | 0 | 0 | 0 | 0 | 0 |  | | NA |  | NA | 2 | 2 | 2 | 0 | 0 | 0 |  | 3 | 27 | 0 | 7 | 0 |  |
| 8655 | 0 | 23.450001 | 5.71 | 24.34968 | 75.650322 | 0 | 11.906913 | 0 | -0.59363079 | 4 | 106.59646 | 17.505554 | 84.080002 | 1 | 6 | 5 | 1 | 0 | 0 | 0 | 0 | 0 |  | 130.7255962 | 2.657137831 | 93.87684116 | -0.991447054 | 2 | 2 | 2 | 0 | 0 | 0 | 0 | 3 | 25 | 0 | 7 | 0 |  |
| 8655 | 0 | 17.385 | 5.3699999 | 30.888697 | 69.111305 | 1 | 12.202601 | 0 | -0.59363079 | 4 | 106.59646 | 17.875002 | 81 | 1 | 12 | 5 | 1 | 0 | 0 | 0 | 0 | 0 | 0 | 127.6733944 | 2.394182223 | 76.59675369 | -2.89646747 | 2 | 2 | 2 | 0 | 0 | 0 |  | 3 | 25 | 0 | 7 | 0 |  |
| 8656 | 1 | 18.25 | 6.263 | 34.31781 | 65.68219 | 0 | 10.773443 | 0 | -0.59363079 | 3 | 114.8511 | 22.407223 | 75.616 | 1 | 0 | 5 | 1 | 1 | 0 | 0 | 1 | 0 |  | 121.1086001 | 1.846802667 | 101.3877078 | 0.255079054 | 2 | 2 |  | 1 | 0 | 0 | 0 | 4 |  | | | | |
| 8656 | 1 | 24.013 | 7.3299999 | 30.525131 | 69.474869 | 0 | 11.512663 | 0 | -0.59363079 | 3 | 114.8511 | 21.719444 | 76.410004 | 1 | 1 | 5 | 1 | 1 | 1 | 0 | 0 | 0 |  | 114.400819 | 1.263711543 | 104.231325 | 0.807028306 | 2 | 2 | 2 | 1 | 0 | 0 |  | 4 | 27 | 0 | 7 | 0 |  |
| 8656 | 1 | 17.974001 | 6.4499998 | 35.885166 | 64.114838 | 0 | 11.594798 | 0 | -0.59363079 | 3 | 114.8511 | 20.752779 | 79.349998 | 1 | 3 | 5 | 1 | 1 | 0 | 0 | 0 | 0 |  | | NA |  | NA | 2 | 2 | 2 | 1 | 0 | 0 |  | 4 | 27 | 0 | 7 | 0 |  |
| 8656 | 0 | 20.798 | 6.4499998 | 31.012596 | 68.987404 | 0 | 11.671458 | 0 | -0.59363079 | 3 | 114.8511 | 19.920557 | 79.779999 | 1 | 5 | 5 | 1 | 0 | 0 | 0 | 0 | 0 |  | | NA |  | NA | 2 | 2 | 2 | 1 | 0 | 0 |  | 4 | 27 | 0 | 7 | 0 |  |
| 8656 | 1 | 23.652 | 5.75 | 24.310841 | 75.689163 | 0 | 11.715263 | 0 | -0.59363079 | 3 | 114.8511 | 17.490557 | 83.980003 | 1 | 6 | 5 | 1 | 1 | 0 | 0 | 0 | 0 |  | 121.3365853 | 1.860223092 | 99.28011196 | -0.126917459 | 2 | 2 | 2 | 1 | 0 | 0 | 0 | 4 | 27 | 0 | 7 | 0 |  |
| 8656 | 0 | 17.492001 | 5.3400002 | 30.52824 | 69.471756 | 1 | 12.024641 | 0 | -0.59363079 | 3 | 114.8511 | 18.030001 | 80.419998 | 1 | 12 | 5 | 1 | 0 | 0 | 0 | 0 | 0 | 0 | 116.3225237 | 1.426123047 | 106.6726603 | 1.312383206 | 2 | 2 | 2 | 1 | 0 | 0 |  | 4 | 25 | 0 | 7 | 0 |  |
| 8657 | 0 | 17.785 | 6.147 | 34.562836 | 65.437164 | 1 | 17.612595 | 1 | 2.0574906 | 3 | 126.63857 | 23.934999 | 72.574 | 1 | 0 | 3 | 1 | 0 | 0 | 0 | 0 | 0 | 0 | 136.4007283 | 3.290210267 | 99.25901512 | -0.108271177 | 2 | 2 |  | 1 | 0 | 0 | 0 |  | | | | | |
| 8657 | 0 | 20.639 | 6.3600001 | 30.815447 | 69.184555 | 1 | 18.422998 | 1 | 2.0574906 | 3 | 126.63857 | 19.902777 | 79.900002 | 1 | 5 | 3 | 1 | 0 | 0 | 0 | 0 | 0 | 0 |  | NA |  | NA | 2 | 2 | 2 | 1 | 0 | 0 |  | | 24 | 0 | 7 | 0 |  |
| 8657 | 0 | 23.403 | 5.71 | 24.398581 | 75.601418 | 1 | 18.464066 | 1 | 2.0574906 | 3 | 126.63857 | 17.49889 | 84.120003 | 1 | 6 | 3 | 1 | 0 | 0 | 0 | 0 | 0 | 0 | 140.0157496 | 3.611852576 | 95.59659797 | -0.620560156 | 2 | 2 | 2 | 1 | 0 | 0 | 0 |  | 24 | 0 | 7 | 0 |  |
| 8657 | 0 | 17.167 | 5.1599998 | 30.057667 | 69.942329 | 1 | 18.767967 | 1 | 2.0574906 | 3 | 126.63857 | 18.332779 | 78.720001 | 1 | 12 | 3 | 1 | 0 | 0 | 0 | 0 | 0 | 0 | 127.0530856 | 2.448083003 | 98.16953108 | -0.262688075 | 2 | 2 | 2 | 1 | 0 | 0 |  | | 24 | 0 | 7 | 0 |  |
| 8658 | 1 | 17.642 | 6.11 | 34.633263 | 65.366737 | 1 | 13.549623 | 1 | 2.6434023 | 3 | 124.77712 | 23.530001 | 73.347 | 1 | 0 | 5 |  | 1 | 0 | 0 | 0 | 1 | 0 | 119.5041874 | 1.780018483 | 111.2520998 | 1.944884739 | 2 | 2 |  | 0 | 0 | 0 | 0 | 4 |  | | | | |
| 8658 | 0 | 23.521999 | 7.1300001 | 30.31205 | 69.68795 | 1 | 14.239562 | 1 | 2.6434023 | 3 | 124.77712 | 21.47611 | 77.5 | 1 | 1 | 5 |  | 0 | 0 | 0 | 0 | 0 | 0 | 105.6649712 | 0.518066287 | 114.0470351 | 2.443268442 | 2 | 2 | 2 | 0 | 0 | 0 |  | 4 | 22 | 0 | 7 | 0 |  |
| 8658 | 0 | 17.615999 | 6.4099998 | 36.387375 | 63.612625 | 1 | 14.310746 | 1 | 2.6434023 | 3 | 124.77712 | 20.731112 | 79.099998 | 1 | 3 | 5 |  | 0 | 0 | 0 | 0 | 0 | 0 |  | NA |  | NA | 2 | 2 | 2 | 0 | 0 | 0 |  | 4 | 24 | 0 | 7 | 0 |  |
| 8658 | 0 | 20.761999 | 6.4299998 | 30.970041 | 69.029961 | 1 | 14.387406 | 1 | 2.6434023 | 3 | 124.77712 | 19.916111 | 79.809998 | 1 | 5 | 5 |  | 0 | 0 | 0 | 0 | 0 | 0 |  | NA |  | NA | 2 | 2 | 2 | 0 | 0 | 0 |  | 4 | 22 | 0 | 7 | 0 |  |
[truncated: 141,758 more chars]
